# Ni-catalyzed asymmetric hydrogenation of *N*-aryl imino esters for the efficient synthesis of chiral α-aryl glycines

**DOI:** 10.1038/s41467-020-19807-5

**Published:** 2020-11-23

**Authors:** Dan Liu, Bowen Li, Jianzhong Chen, Ilya D. Gridnev, Deyue Yan, Wanbin Zhang

**Affiliations:** 1 Shanghai Key Laboratory for Molecular Engineering of Chiral Drugs, Frontiers Science Center for Transformative Molecules, School of Chemistry and Chemical Engineering, Shanghai Jiao Tong University, 800 Dongchuan Road, 200240 Shanghai, China; 2 Department of Chemistry, Graduate School of Science, Tohoku University, Aramaki 3-6, Aoba-ku, Sendai 980-8578 Japan

**Keywords:** Asymmetric catalysis, Catalytic mechanisms, Homogeneous catalysis, Stereochemistry

## Abstract

Chiral α-aryl glycines play a key role in the preparation of some bioactive products, however, their catalytic asymmetric synthesis is far from being satisfactory. Herein, we report an efficient nickel-catalyzed asymmetric hydrogenation of *N*-aryl imino esters, affording chiral α-aryl glycines in high yields and enantioselectivities (up to 98% ee). The hydrogenation can be conducted on a gram scale with a substrate/catalyst ratio of up to 2000. The obtained chiral *N*-*p*-methoxyphenyl α-aryl glycine derivatives are not only directly useful chiral secondary amino acid esters but can also be easily deprotected by treatment with cerium ammonium nitrate for further transformations to several widely used molecules including drug intermediates and chiral ligands. Formation of a chiral Ni-H species in hydrogenation is detected by ^1^H NMR. Computational results indicate that the stereo selection is determined during the approach of the substrate to the catalyst.

## Abstract (web summary)

Chiral α-amino acids find application in the fields of pharmaceutical, biological and synthetic chemistry. Here, the authors report a nickel-catalyzed asymmetric hydrogenation of *N*-aryl imino esters affording chiral α-aryl glycines in high yields and enantioselectivities.

## Introduction

**Fig. 1:**
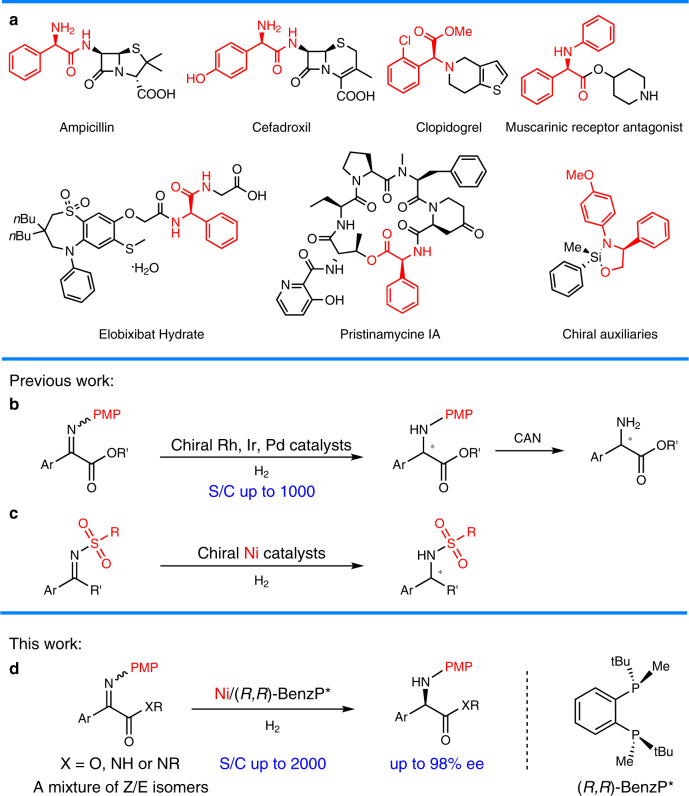
Asymmetric hydrogenation of *N*-aryl imino esters for the synthesis of chiral α-aryl glycines. **a** Representative chiral products bearing chiral α-aryl glycine skeletons. **b** Previous work about asymmetric hydrogenation of *N*-aryl imino esters. **c** Previous work about Ni-catalyzed asymmetric hydrogenation of activated imines. **d** This work: Ni-catalyzed asymmetric hydrogenation of imino esters.

Chiral α-amino acids have found broad applications in the fields of pharmaceutical, biological, and synthetic chemistry^1,2^. Therefore, the synthesis of these compounds is of considerable importance. In the past decades, the synthesis of chiral α-alkyl amino acids, mainly via the asymmetric hydrogenation of the corresponding α-dehydroamino acid derivatives, has been widely investigated affording excellent results^3–7^. A prominent example is the industrial preparation of L-DOPA developed by Knowles, which has received high praise and won the Nobel Prize in Chemistry in 2001^8^. As another category of α-amino acids, chiral α-aryl glycines play a key role in synthetic drugs as well as bioactive natural products (Fig. 1a)^9–12^.

In order to obtain these building blocks, many methodologies have been developed^13–17^. Among various approaches, the transition-metal-catalyzed asymmetric hydrogenation of α-aryl imino esters provides a promising route in terms of both efficiency and practicality. However, due to the coexistence of *Z/E* isomers and the intrinsically low activity of the substrates, studies in this area are still far from satisfactory^18–30^. Although several strategies have been developed to overcome these drawbacks, including introducing activated groups like *N*-sulfonyl imino esters or using cyclic imino esters, the corresponding products either contain groups which cannot be easily removed or are limited to cyclic derivatives^18–27^. As an alternative, *N*-*p*-methoxyphenyl (PMP) substrates have the advantages of possessing a readily removable PMP group and greater substrate diversity. Some asymmetric hydrogenations of *N*-PMP α-aryl imino esters based on noble transition-metal catalysts of Pd, Rh, and Ir have been reported with good results (S/C up to 1000, Fig. 1b)^28–30^.

In view of current interest in the development of earth-abundant metal catalysts in asymmetric hydrogenation^31–41^, which are inexpensive and environmentally friendly, the use of nickel has drawn increasing attention^24,31–41^. Although Ni-catalyzed asymmetric reduction has seen rapid development, the asymmetric hydrogenation of imines is still in its infancy^24,42–44^. To date, only a few Ni-catalyzed asymmetric hydrogenations of imines have been reported by Zhang^24,43,44^ and our group^42^, and the imines substrates are all activated by *N*-sulfonyl groups (Fig. 1c). The electronic-withdrawing sulfonyl substituents at the nitrogen atom can increase the electrophilicity of the imine carbon, which is more vulnerable to hydride attack. To the best of our knowledge, there is still no Ni-catalyzed asymmetric hydrogenation of unactivated *N*-aryl imines. Continuing our pursuit of earth-abundant metal-catalyzed asymmetric hydrogenation^38,39,42^, herein we disclose an efficient Ni-catalyzed asymmetric hydrogenation of *N*-PMP imino esters for the synthesis of useful chiral α-aryl glycines (Fig. 1d).

## Results

### Investigation of reaction conditions

Initially, our investigations were performed using (*Z*/*E*)-methyl-2-((4-methoxyphenyl)imino)-2-phenylacetate (**1a**) as a model substrate with 1 mol% catalyst under 30 bar H_2_ at 50 °C for 24 h. As listed in Table 1, a variety of chiral diphosphine ligands (entries 1–7) and commonly used solvents (entries 8–14) were explored.

**Table 1:** Reaction optimization^a^.


entry	Ligand	Solvent	Conv %^b^	ee %^c^
1	(*S*)-BINAP	TFE	7	82
2	(*S*)-SegPhos	TFE	50	84
3	(*R,Sp*)-JosiPhos	TFE	95	65
4	(*Rc,Sp*)-DuanPhos	TFE	99	83
5	(*S,S*)-Ph-BPE	TFE	99	91
6	(*R,R*)-QuinoxP*	TFE	99	92
7	(*R,R*)-BenzP*	TFE	99	96
8	(*R,R*)-BenzP*	MeOH	99	43
9	(*R,R*)-BenzP*	EtOH	58	50
10	(*R,R*)-BenzP*	(CF_3_)_2_CHOH	98	76
11	(*R,R*)-BenzP*	Toluene	0	–
12	(*R,R*)-BenzP*	CH_2_Cl_2_	0	–
13	(*R,R*)-BenzP*	EtOAc	0	–
14	(*R,R*)-BenzP*	THF	trace	–


^a^Reaction conditions: **1a** (0.3 mmol), Ni(OAc)_2_·4H_2_O (0.003 mmol), Ligand (0.003 mmol), H_2_ (30 bar), TFE (1 mL), 50 ^o^C, 24 h.

^b^The conversions were calculated by ^1^H NMR spectra.

^c^The ee values were determined by HPLC using chiral stationary phase.

When (*S*)-BINAP was used, only a trace amount of product was obtained (entry 1). By replacing (*S*)-BINAP with the electron-rich ligand (*S*)-SegPhos, the conversion of **1a** was improved from 7% to 50% (entry 2). To our delight, introducing a more electron-rich alkyl phosphine to the ligand could dramatically increase the reaction activity (entries 3-7), and full conversion with the best enantioselectivity (99% conv, 96% ee) was obtained by using the chiral dialkyl phosphine ligand (*R,R*)-BenzP* (entry 7). In solvent screening experiments, 2,2,2-trifluoroethanol (TFE) provided the best result for this reaction. Other protic solvents, such as MeOH, EtOH and (CF_3_)_2_CHOH, were viable but gave the desired product with low ee values (entries 8-10), whereas the use of aprotic solvents, such as toluene, CH_2_Cl_2_, EtOAc, and THF resulted in no or little reactivity (entries 11-14).

### Scope of asymmetric catalysis of *N*-PMP α-aryl imino esters

With the optimized reaction conditions in hand (Table 1, entry 7), we next investigated the substrate scope of this asymmetric hydrogenation (Table 2). Substrates with both electron-donating (Me, MeO, etc.) and electron-withdrawing (F, Cl, Br, etc.) groups could be hydrogenated to the corresponding products with good to high yields and excellent ees (**2b-2t**, 90–98% ee) regardless of the positions on the aryl moieties. To our delight, the 3-NO_2_ substituted *N*-aryl imino ester **1k** was also amenable to the reaction conditions (83% yield, 90% ee) with no reduction of the NO_2_ group being observed, even at a higher temperature. Disubstituted and naphthyl substrates were also evaluated in the catalytic reactions, affording excellent results (**2u-2ab**, 92–98% ee). Altering the ester groups showed no obvious influence on the reactivity and enantioselectivity (**2ac-2ae**). Changing the esters to amide groups also afforded good results with only slightly lower yields of the corresponding products (**2af** and **2ag**). The reaction was also applicable to heteroaryl substituents (**1ah** and **1ai**) affording the desired products in 90%/23% yield and 73%/39% ee, respectively. Excellent results were also obtained when replacing the PMP with other aryl groups (**2aj**-**2am**, 94–98% ee). It should be noted that methyl substituted substrate **1am** was smoothly hydrogenated to product **2am** with 94% yield and 98% ee, which is a key intermediate for the synthesis of the chiral fungicide (*R*)-metalaxyl^45^. The absolute configuration of product **2a** was assigned to be *R* by X-ray crystallographic analysis.

**Table 2:**
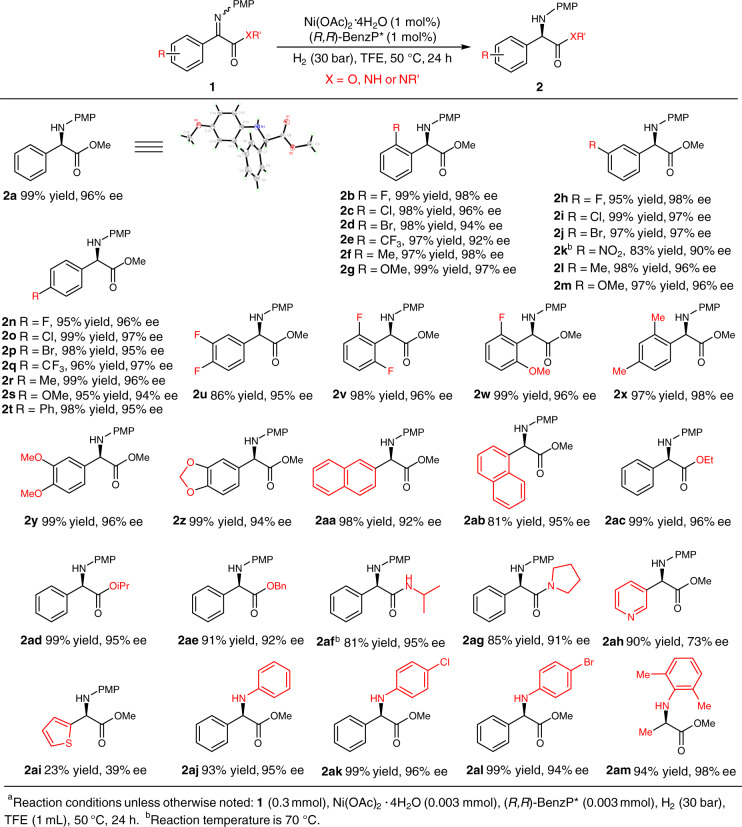
Substrate scope^a^.

### Synthetic utility of chiral α-aryl glycine products

**Fig. 2:**
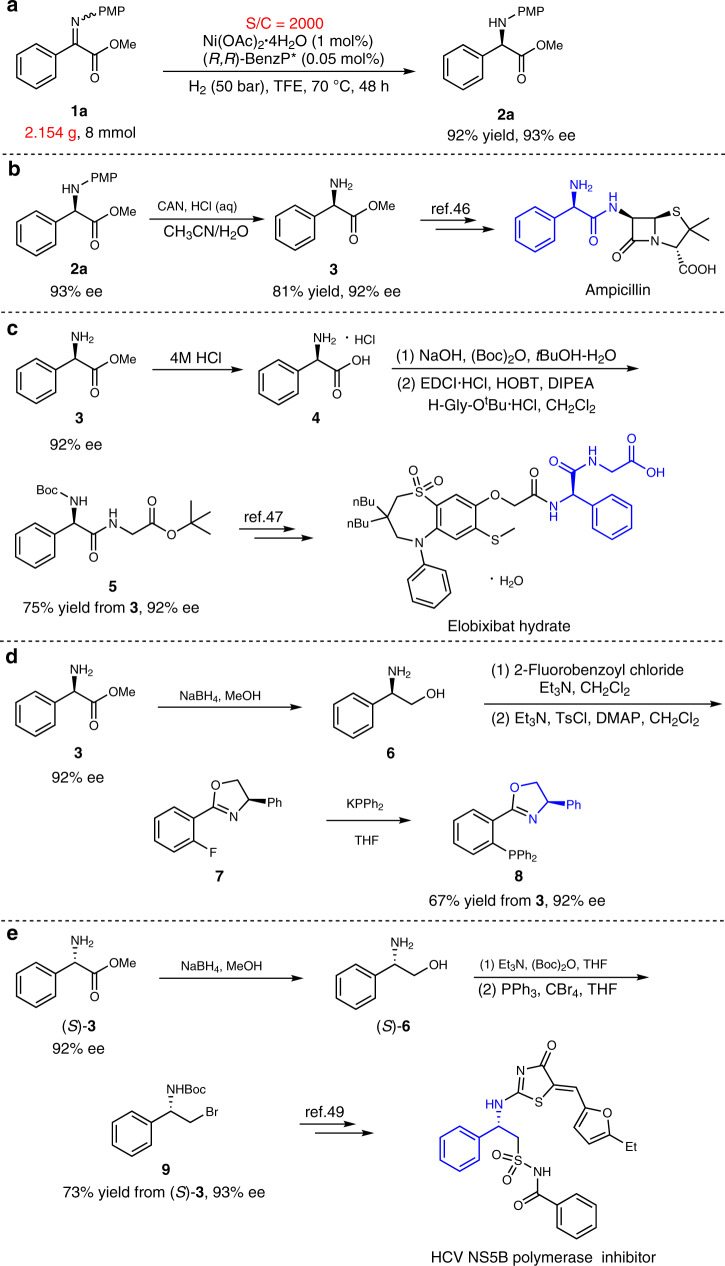
Practical applications. **a** A gram scale experiment. **b** The removal of PMP. **c** The synthesis of elobixibat hydrate intermediate. **d** The synthesis of ligand **8**. **e** The synthesis of HCV NS5B polymerase inhibitor intermediate.

To demonstrate the synthetic utility of this method, a gram scale experiment with a high substrate/catalyst ratio (S/C = 2000) was carried out (Fig. 2a). As a result, **1a** was hydrogenated to produce **2a** with 92% yield and 93% ee. It should be noted that a high Ni/ligand ratio was vital for the reaction efficiency (see SI for details). Next, several transformations of the chiral α-aryl glycine derivatives were conducted. The PMP group of product **2a** could be smoothly removed by the use of cerium ammonium nitrate (CAN) to give the deprotected product **3** in high yield and without an obvious loss of enantioselectivity (Fig. 2b). The chiral primary amine **3** is an intermediate for the synthesis of the marketed drug Ampicillin (Fig. 2b)^46^. The hydrolysis of **3** gave the chiral α-aryl glycine hydrochloride **4**, which could be further transformed to chiral compound **5**, an intermediate for the synthesis of the commercial drug Elobixibat hydrate (Fig. 2c)^47^. The compound **3** was also reduced with NaBH_4_ to afford the corresponding amino alcohol **6**, which was further transformed to chiral compound **8** via intermediate **7** (Fig. 2d). Chiral compound **8** has been widely used as a ligand for numerous asymmetric catalytic reactions^48^. Chiral (*S*)-**6** derived from (*S*)-**3** could be also transformed to **9**, which is an intermediate for the synthesis of the HCV NS5B polymerase inhibitor (Fig. 2e)^49^.

### The studies of coordination and Ni-H species

**Fig. 3:**
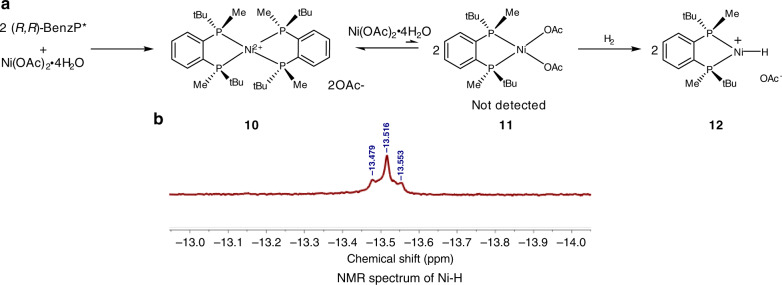
The experimental studies of coordination and Ni-H species. **a** Formation of the catalyst **12**. **b** ^1^H NMR Spectrum of Ni−H.

Active chiral metal hydride species are the crucial intermediates in the transition-metal-catalyzed asymmetric hydrogenation process, but they are very difficult to detect and isolate due to their high-activities and generation in only trace amounts^50^. Thus, the active chiral nickel hydride species has remained undetected, albeit several chiral diphosphine ligand nickel complexes have been well studies. In this study, at first, based on our previous studies of Ni catalysts^39,42^, the coordination behaviors of Ni salt and the ligand BenzP* were studied. Unlike previous investigations^39,42^, (*R,R*)-BenzP* (L) coordinates with Ni(OAc)_2_·4H_2_O (M) to form a dual-ligand coordinated complex **10** with different L/M ratios in CF_3_CD_2_OD, with none of the mono-ligand coordinated complex **11** being detected by ^1^H NMR and HRMS, probably because it exists in trace amounts in an equilibrium with the dominating complex **10** (Fig. 3a). Afterwards, a sample analyzed upon hydrogenation of the solution containing a 1:1 ratio of L/M exhibited a weak hydride signal at δ = −13.52 ppm (t, ^2^*J*_P-H_ = 14.8 Hz) (Fig. 3b). The triplet structure of this signal is evidence of its coupling with two equivalent *cis*-phosphorus atoms that strongly supports the presence of the structure **12**. Similar species were previously detected for the analogous Pd complex^50^, and it is commonly accepted that these species are real catalysts in Pd-catalyzed hydrogenations. Hence, our experimental observation supports previous studies, in that a nickel hydride complex most likely acts as the catalyst^39,42^.

### *Z/E* isomers interconversion and deuterium labeling experiments

The ^1^H NMR spectrum of a crystal of (*Z*)-**1a** dissolved in the solvent showed a mixture of *Z*/*E* isomers, indicating that there is rapid interconversion between the two isomers (Supplementary Fig. 5). Furthermore, complete deuteration occurred at the prochiral carbon atom in a deuterium labeling experiment using D_2_ (Supplementary Fig. 6).

### Mechanistic considerations

**Fig. 4:**
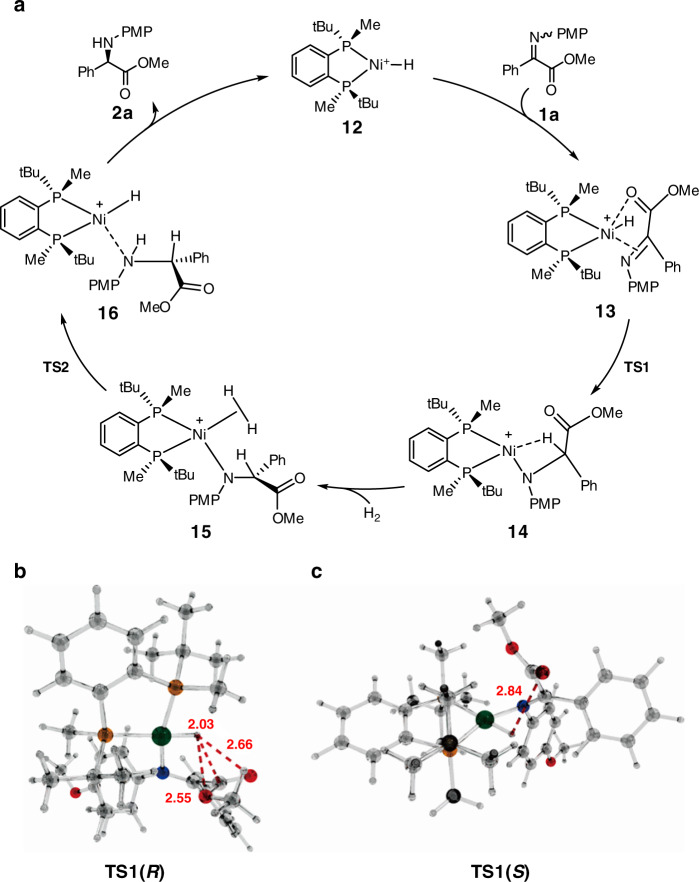
Computed mechanism of catalytic cycle and important stabilizing interactions (Angstrom). **a** Computed mechanism of catalytic cycle. **b** Optimized structures of **TS1**(*R*). **c** Optimized structures of **TS1**(*S*).

Based on the above experimental results, we have computed a catalytic cycle for the reaction under study considering four competing reaction pathways (*R*- and *S*-pathways for (*Z*)-**1a** and (*E*)-**1a**, e.g. Fig. 4a). On approach of (*Z*)-**1a** or (*E*)-**1a** to the catalyst **12**, we have located four diastereomers of the chelate complex **13**. Further approach along the same coordinate results in the hydride transfer producing intermediate **14**. Coordination of H_2_ to **14** forms complex **15**, which undergoes subsequent sigma-bond metathesis to give complex **16**. Finally, **2a** is released to regenerate the Ni-H species necessary for the next catalytic turnover.

Enantioselectivity is generated during hydride transfer via **TS1**. Among four computed transition states, the two most stable originate from (*E*)-**1a**; **TS1(*R*)** is 1.8 kcal/mol more stable than **TS1(*S*)** (see SI for details). The main structural difference leading to the notable alteration in their energies is the interaction of the carboxymethyl substituent with the hydride being transferred from Ni to carbon. This is illustrated in Fig. 4b: in the ***S*** transition state only one quite long H^…^O contact of this type can be found, whereas in the ***R*** transition state the carboxymethyl group is completely involved in stabilization of the **TS1(*R*)** transition state via appropriately distanced intramolecular interactions (the distance C(carbonyl)-H(hydride) is 2.03 Å). This is further illustrated in Fig. 4c.

Usually, migratory insertion of a metal hydride proceeds via gradual elongation of the M–H bond supported by gradual shortening of the H–C distance and formation of an M–C (or M–N) bond. These processes are characterized by high absolute values of the imaginary vibrations in the corresponding transition states.

**Fig. 5:**
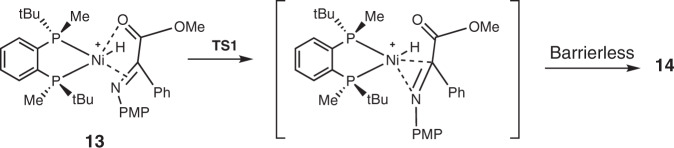
Intrinsic mechanism of the hydride transfer. The configuration with coplanar orientation of Ni–C and Ni–H bonds.

In all four computed reaction pathways, the hydride insertion proceeds differently: the very low barrier (1-2 kcal/mol) or even barrierless hydride transfer takes place after the formation of a configuration with coplanar orientation of Ni-C and Ni-H bonds that requires initial elongation of the C-N bond resulting in the appearance of the activation barrier (Fig. 5, see SI for details)^51^. Hence, stereoselection actually occurs at the stage of achieving a proper mode of the substrate coordination for the subsequent hydride transfer^52^, which parallels that of Rh-catalyzed asymmetric hydrogenation^53,54^.

Experimentally observed enantioselection (96% ee) attests to a higher energy difference (about 2.3 kcal/mol). Our computations suggest that the alternative pathways, avoiding formation of chelating complexes **13**, can be more stereoselective, and their interference may improve the optical yields.

**Fig. 6:**
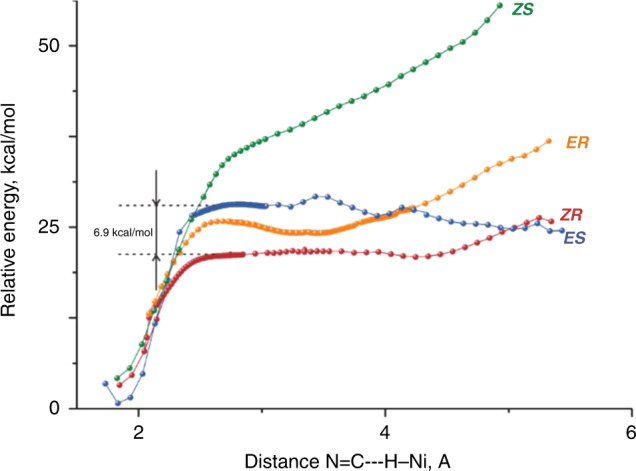
Energy scans simulating the approach of 1a to the catalytic hydride 12. ***ZS***, ***ER***, ***ZR***, ***ES***: the approach of (*Z*)-**1a** or (*E*)-**1a** to the catalyst to form the products (*S*)-**2a** or (*R*)-**2a**, respectively.

To gain further insight into the asymmetric pathways, detailed computational studies of the approach of the substrate to the catalyst were conducted. From Fig. 6, it is clear that stereo differentiation could be effectively achieved at this stage. Thus, both pathways leading to the experimentally observed *R*-product are continuously lower in energy than both alternative pathways starting from the separation distance of 4 Å. The energy gap between the approaching curves for ***ES*** and ***ZR*** is continuously maintained at approximately 6.9 kcal/mol which is enough to secure perfect enantioselection. Of course, thermodynamics should favor the formation of the complexes **13**, but the interference of the pathways shown in Fig. 6 cannot be excluded.

It should be noted that the steep drop in the molecular relative energy starting at each of the four pathways at approximately 2.4 Å corresponds to the building of Ni–C bonding, since at that stage the length of the Ni–H bond is not yet increasing. Really, barrierless migratory insertion begins at approximately 2.1 Å in each case, i.e. in the area when the difference between the relative energies of the four pathways can be neglected (Fig. 6). Hence, the stereodiscrimination in this case is also achieved on the stage of the approach of the substrate to the catalyst.

## Discussion

In summary, we have developed an efficient Ni-catalyzed asymmetric hydrogenation of *N*-aryl imino esters to synthesize chiral α-aryl glycines in high yields and with excellent enantioselectivities (up to 98% ee). The reaction proceeded smoothly on a gram scale at a low catalyst loading (S/C up to 2000). The obtained products were directly useful chiral secondary amino acid ester or further applied to the synthesis of several widely used molecules including drug intermediates and chiral ligand. A chiral active Ni–H species has been discovered first using NMR spectroscopy. Computational results indicate that the stereo selection is determined during the approach of the substrate to the catalyst.

## Methods

### General procedure for asymmetric hydrogenation of *N*-aryl imino esters/amides

To a hydrogenation tube, Ni(OAc)_2_·4H_2_O (0.75 mg, 0.003 mmol), (*R,R*)-BenzP* (0.85 mg, 0.003 mmol) and the substrate (S/C = 100) were added, and then the mixture was transferred to a nitrogen-filled glovebox. The degassed and anhydrous trifluoroethanol (TFE, 1.0 mL) was added. The reaction was performed with H_2_ (30 bar) at 50 °C for 24 h. After carefully releasing hydrogen gas, the pure product is obtained by column chromatography (PE/EtOAc). The enantiomeric excess was determined by chiral HPLC.

## Supplementary information

Supplementary Information

Description of Additional Supplementary Files

Supplementary Data 1

## Data Availability

The authors declare that the data supporting the findings of this study are available within the article and its Supplementary Information file. For the experimental procedures, data of NMR and HPLC analysis and Cartesian coordinates of the optimized structures, see Supplementary Methods in Supplementary Information file. The X-ray crystallographic coordinates for structures reported in this article have been deposited at the Cambridge Crystallographic Data Centre (***Z*****-1a**: CCDC 2011640 [https://www.ccdc.cam.ac.uk/structures/Search?access=referee&ccdc=2011640&Author=Liu+Dan], ***R*****-2a**: CCDC 2011648 [https://www.ccdc.cam.ac.uk/structures/Search?access=referee&ccdc=2011648&Author=Dan+Liu]). These data could be obtained free of charge from The Cambridge Crystallographic Data Centre (https://www.ccdc.cam.ac.uk/structures/).
