# Supplementary material for: Ni-catalyzed asymmetric hydrogenation of N-aryl imino esters for the efficient synthesis of chiral α-aryl glycines
Source: Nat Commun. 2020 Nov 23;11:5935. doi: 10.1038/s41467-020-19807-5 (PMC7683563; doi:10.1038/s41467-020-19807-5)
Supplement: Supplementary file 1 — Supplementary Information [file 41467_2020_19807_MOESM1_ESM.pdf]

Supplementary Information

Ni-Catalyzed Asymmetric Hydrogenation of *N*-Aryl Imino Esters for the Efficient Synthesis of Chiral α-Aryl Glycines

Liu et al

Supplementary Methods

General information

All reactions were performed in flame-dried glassware or in an autoclave under an atmosphere of hydrogen unless otherwise specifically stated. Hydrogenation solvents were used after being dried and degassed by standard procedures. Commercially available reagents were applied without further purification. $Ni(OAc)_2 \cdot 4H_2O$ was purchased from Adamas-bfeta and (*R,R*)-BenzP* was from Tokyo Chemical Industry (TCI).

^{1}H NMR, ^{13}C NMR, ^{19}F NMR spectra were recorded on Varian MERCURY plus-400, Bruker AVIII400 spectrometer. HRMS was performed on Waters Micromass Q-TOF Premier Mass Spectrometer at the Instrumental Analysis Center of Shanghai Jiao Tong University. Reagent weighing is made using a Mettler-Toledo analytical balance. Column chromatography was performed with 100-200 mesh silica gel. Melting points were measured with SGW X-4 micro melting point apparatus at a rate of 5 ℃/min. Optical rotations were measured on a Rudolph Research Analytical Autopol VI automatic polarimeter using a 100 mm path-length cell at 589 nm. Enantiomeric excesses were measured by high performance liquid chromatography using Daicel Chiralcel columns with hexane/2-propanol as eluent.

Supplementary Note 1

Preparation of α-aryl ketone esters/amides

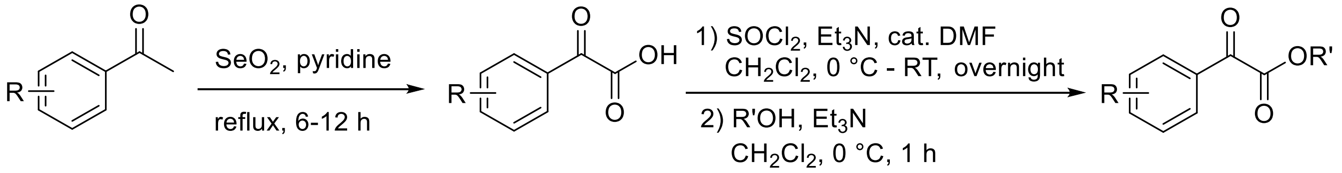

Procedure A[1-2]: To a 100 mL flame-dried round bottom flask equipped with a stirrer bar in 30 mL of pyridine, were added the corresponding substituted ketones (20 mmol) and selenium dioxide (3.33 g, 30 mmol). The mixture was stirred at reflux and monitored by thin layer chromatography (TLC). After completion of the reaction, the dark solution was filtered and the residue was washed with EtOAc three times. The collected filtrate was then treated with concentrated HCl until pH = 1-2 and extracted with EtOAc (3 × 60 mL). The combined organic layer was dried over anhydrous Na_2SO_4 and concentrated in vacuum. The desired α-aryl ketone acids were isolated by silica gel column chromatography (PE/EtOAc = 10:1 - 5:1).

The obtained α-aryl ketone acids (14 mmol) and triethylamine (1.95 mL, 14 mmol) were dissolved in CH_2Cl_2 (15 mL), and then thionyl chloride (2.03 mL, 28 mmol) was added dropwise at 0 ℃. The mixture was stirred overnight at room temperature in the presence of a catalytic amount of DMF (two drops). Afterwards, the above prepared mixture was added dropwise over 0.5 h to a solution of corresponding alcohol (3.4 mL, 84 mmol) and triethylamine (5.87 mL, 42 mmol) in CH_2Cl_2 (25 mL) at 0 ℃. After 0.5 h, saturated aqueous $NaHCO_3$ solution (20 mL) was added to quench the reaction and the mixture was extracted with CH_2Cl_2 (3 × 30 mL). After being dried and concentrated, the crude product was purified by column chromatography using PE/EtOAc (40:1) as eluent to give the α-aryl imino ester product (62-85% yield).

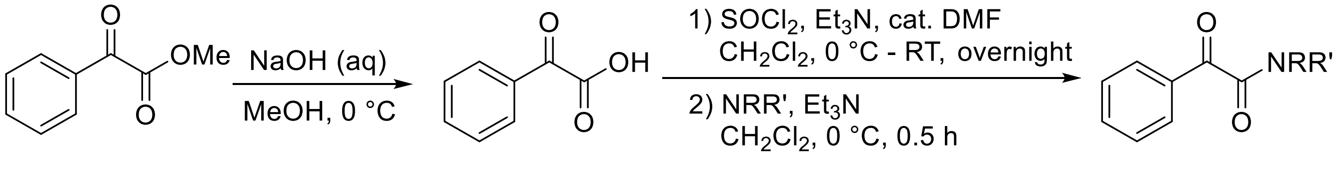

Procedure B[2]: To a methanol solution (25 mL) of methyl phenylglyoxylate (1.69 mL, 12 mmol) at 0 ℃ was added dropwise aqueous NaOH solution (quantitative), and the resulting mixture was stirred and monitored by thin layer chromatography (TLC). When the reaction was complete,

the system was acidified to pH = 1-2 with concentrated HCl and extracted with EtOAc. The combined organic phase was washed with brine, dried over anhydrous Na_2SO_4, and concentrated to give phenylglyoxylic acid which was used without further purification in the next step.

A mixture of phenylglyoxylic acid (1.71 g, 11.4 mmol) and triethylamine (1.58 mL, 11.4 mmol) in CH_2Cl_2 (15 mL) was stirred at 0 ℃ for 15 min followed by the addition of thionyl chloride (1.65 mL, 22.8 mmol) dropwise under the ice bath. The reaction was allowed to warm to room temperature and stirred overnight with a catalytic amount of DMF (two drops). Then the reaction mixture above was directly added dropwise over 0.5 h to a solution of the corresponding amine (17.1 mmol) and triethylamine (6.33 mL, 45.6 mmol) in CH_2Cl_2 (20 mL) at 0 ℃ before being diluted with saturated aqueous $NaHCO_3$ solution (20 mL) and extracted with CH_2Cl_2 (3 × 40 mL) . The combined organic layer was dried over anhydrous Na_2SO_4, concentrated in vacuo, and purified by column chromatography (PE/EtOAc = 5:1) to afford the desired α-aryl imine amide product (55-87% yield).

Preparation of *N*-aryl imino esters/amides

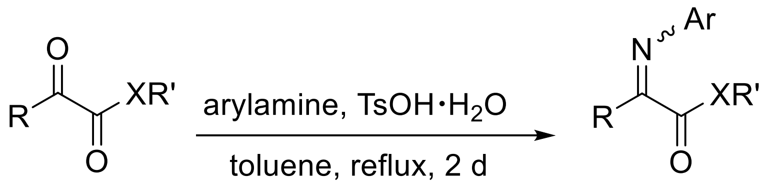

1a-1am

Procedure C[3]: A mixture of the corresponding imino ester/amide (10 mmol), arylamine (15 mmol), and *p*-toluenesulfonic acid monohydrate (95.11 mg, 0.5 mmol) in toluene (20 mL) was heated to reflux with azeotropic removal of water. After the completion of the reaction as monitored by TLC detection, solvent was removed in vacuo, and the residue was purified by column chromatography (PE/EtOAc = 40:1 - 5:1) to give the corresponding *N*-aryl α-aryl imino ester/amide. The product was further purified by recrystallization if necessary. The ratio of geometric isomers was determined by ^1H NMR spectrometry.

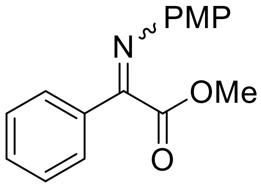

Methyl 2-((4-methoxyphenyl)imino)-2-phenylacetate (1a)[3]

76% yield, yellow solid, 90:10 mixture of geometric isomers; ^{1}H NMR (400 MHz, Chloroform-*d*) ***Z*** isomer (major) *δ* 7.89 (d, *J* = 6.4 Hz, 2H), 7.55-7.45 (m, 3H), 7.00 (d, *J* = 8.8 Hz, 2H), 6.91 (d, *J* = 8.8 Hz, 2H), 3.83 (s, 3H), 3.72 (s, 3H); minor isomer *δ* 7.38-7.29 (m, 3H), 7.23 (d, *J* = 7.6 Hz, 2H), 6.78 (d, *J* = 8.8 Hz, 2H), 6.74 (d, *J* = 8.8 Hz, 2H), 3.98 (s, 3H), 3.76 (s, 3H); ^{13}C NMR (101 MHz, Chloroform-*d*) *δ* 166.1, 159.1, 157.4, 143.1, 134.1, 131.6, 128.7, 127.8, 121.2, 114.2, 55.4, 52.0, resonances of the minor isomer are obscured.

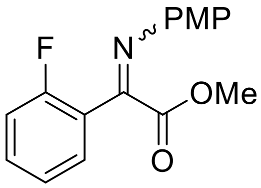

Methyl 2-(2-fluorophenyl)-2-((4-methoxyphenyl)imino)acetate (1b)[3]

64% yield, yellow solid after being laid aside for a long time, 54:46 mixture of geometric isomers; ^{1}H NMR (400 MHz, Chloroform-*d*) ***Z*** isomer (major) *δ* 8.03 (td, *J* = 7.6 Hz, 2.0 Hz, 1H), 7.53-7.47 (m, 1H), 7.30-7.26 (m, 1H), 7.15-7.10 (m, 1H), 6.97 (d, *J* = 8.8 Hz, 2H), 6.90 (d, *J* = 8.8 Hz, 2H), 3.83 (s, 3H), 3.70 (s, 3H); minor isomer *δ* 7.39-7.33 (m, 1H), 7.08-7.04 (m, 3H), 6.80 (d, *J* = 9.2 Hz, 2H), 6.73 (d, *J* = 9.2 Hz, 2H), 3.97 (s, 3H), 3.75 (s, 3H); ^{13}C NMR (101 MHz, Chloroform-*d*) *δ* 165.6, 164.6, 161.5 (d, *J* = 253.5 Hz), 159.3 (d, *J* = 140.4 Hz),158.0, 157.6, 154.3 (d, *J* = 83.8 Hz), 142.8, 141.2, 133.3 (d, *J* = 9.1 Hz), 131.5 (d, *J* = 8.1 Hz), 130.2 (d, *J* = 4.0 Hz), 130.0 (d, *J* = 3.0 Hz), 124.7 (d, *J* = 4.0 Hz), 124.1 (d, *J* = 4.0 Hz), 123.4 (d, *J* = 10.1 Hz), 122.9, 122.0 (d, *J* = 17.2 Hz), 121.2, 116.2, 116.0, 115.8, 114.2, 113.9, 55.4, 55.3, 53.4, 52.2.

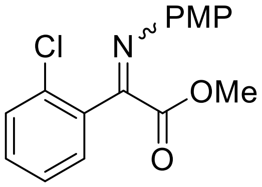

Methyl 2-(2-chlorophenyl)-2-((4-methoxyphenyl)imino)acetate (1c)

62% yield, yellow solid after being laid aside for a long time, melting point: 60.1-64.1 ^{o}C; 88:12 mixture of geometric isomers; ^{1}H NMR (400 MHz, Chloroform-*d*) ***Z*** isomer (major) *δ* 7.40-7.37

(m, 1H), 7.30-7.26 (m, 1H), 7.16 (td, J = 8.0 Hz, 1.2 Hz, 1H), 6.97 (d, J = 8.0 Hz, 1H), 6.78 (d, J = 8.0 Hz, 2H), 6.70 (d, J = 8.0 Hz, 2H), 3.94 (s, 3H), 3.72 (s, 3H); minor isomer δ 6.93-6.88 (m, 4H), 3.81 (s, 3H), 3.65 (s, 3H), other aromatic resonances are obscured; ^{13}C NMR (101 MHz, Chloroform-*d*) δ 164.5, 164.4, 158.1, 157.6, 157.2, 156.3, 143.5, 140.8, 135.1, 134.1, 133.3, 132.9, 131.9, 131.7, 130.5, 130.1, 129.9, 129.5, 127.2, 126.9, 123.4, 120.8, 114.2, 113.8, 55.4, 55.3, 53.4, 52.3; HRMS (ESI-MS) Calcd. For $C_{16}H_{14}ClNO_3$ [M+H]$^+$ 304.0735, found: 304.0737.

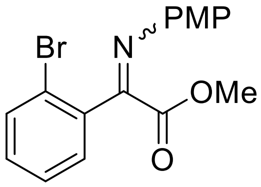

Methyl 2-(2-bromophenyl)-2-((4-methoxyphenyl)imino)acetate (1d)

67% yield, yellow solid after being laid aside for a long time, melting point: 80.3-81.1 ^{o}C; 92:8 mixture of geometric isomers; ^{1}H NMR (400 MHz, Chloroform-*d*) ***Z*** isomer (major) δ 7.58-7.56 (m, 1H), 7.23-7.19 (m, 2H), 6.96-6.93 (m, 1H), 6.80 (d, J = 9.2 Hz, 2H), 6.70 (d, J = 9.2 Hz, 2H), 3.94 (s, 3H), 3.72 (s, 3H); minor isomer δ 7.70 (dd, J = 7.6 Hz, 1.6 Hz, 1H), 7.61 (dd, J = 8.0 Hz, 1.2 Hz, 1H), 7.41 (td, J = 7.6 Hz, 1.6 Hz, 1H), 7.32 (td, J = 7.2 Hz, 1.6 Hz, 1H), 6.92-6.89 (m, 4H), 3.81 (s, 3H), 3.65 (s, 3H); ^{13}C NMR (101 MHz, Chloroform-*d*) δ 164.3, 163.9, 158.7, 158.1, 157.6, 157.3, 143.0, 140.7, 136.9, 135.9, 133.4, 132.6, 131.9, 131.7, 130.6, 130.1, 127.7, 127.5, 123.5, 122.2, 122.0, 120.7, 114.2, 113.8, 55.4, 55.3, 53.4, 52.3; HRMS (ESI-MS) Calcd. For $C_{16}H_{14}BrNO_3$ [M+H]$^+$ 348.0230, found: 348.0233.

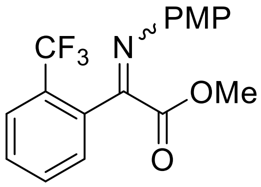

Methyl 2-((4-methoxyphenyl)imino)-2-(2-(trifluoromethyl)phenyl)acetate (1e)

62% yield, yellow solid after being laid aside for a long time, melting point: 74.8-75.4^{o}C; 91:9 mixture of geometric isomers; ^{1}H NMR (400 MHz, Chloroform-*d*) ***Z*** isomer (major) δ 7.69-7.67 (m, 1H), 7.47-7.43 (m, 2H), 7.11-7.05 (m, 1H), 6.75 (d, J = 9.2 Hz, 2H), 6.68 (d, J = 9.2 Hz, 2H), 3.90 (s, 3H), 3.69 (s, 3H); minor isomer δ 6.94-6.87 (m, 4H), 3.80 (s, 3H), 3.61 (s, 3H), other aromatic resonances are obscured; ^{13}C NMR (101 MHz, Chloroform-*d*) δ 164.6, 158.2, 156.0, 140.3, 133.3 (q, J = 2.0 Hz), 132.0, 129.9, 129.4, 129.0 (q, J = 31.3 Hz), 126.8 (q, J = 4.0 Hz) , 124.0, 123.8 (q, J = 274.7 Hz), 112.8, 55.4, 55.3, 53.3, 52.2, other resonances of the minor

isomer are obscured; ^{19}F NMR (376 MHz, Chloroform-*d*) *δ* -58.04, -59.87; HRMS (ESI-MS) Calcd. For $C_{17}H_{14}F_3NO_3$ [M+Na]$^+$ 360.0818, found: 360.0822.

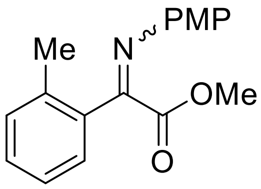

Methyl 2-((4-methoxyphenyl)imino)-2-(*o*-tolyl)acetate (1f)

56% yield, yellow solid after being laid aside for a long time, melting point: 59.3-60.5 °C; 80:20 mixture of geometric isomers; ^{1}H NMR (400 MHz, Chloroform-d) ***Z*** isomer (major) *δ* 7.26-7.23 (m, 1H), 7.18-7.14 (m, 2H), 7.08 (d, *J* = 7.6 Hz, 1H), 6.77 (d, *J* = 6.8 Hz, 2H), 6.68 (d, *J* = 6.8 Hz, 2H), 3.92 (s, 3H), 3.71 (s, 3H), 2.10 (s, 3H); minor isomer *δ* 7.52 (d, *J* = 8.0 Hz, 1H), 7.36-7.31 (m, 1H), 6.95-6.87 (m, 4H), 3.80 (s, 3H), 3.63 (s, 3H), 2.52 (s, 3H), other aromatic resonances are obscured; ^{13}C NMR (101 MHz, Chloroform-*d*) *δ* 165.3, 159.6, 158.3, 143.2, 141.3, 137.2, 135.7, 134.5, 131.8, 130.6, 129.5, 128.6, 126.2, 126.1, 124.3, 121.0, 114.5, 113.9, 55.7, 55.5, 53.0, 52.2, 20.9, 19.9, other resonances of the minor isomer are obscured; HRMS (ESI-MS) Calcd. For $C_{17}H_{17}NO_3$ [M+H]$^+$ 284.1281, found: 284.1282.

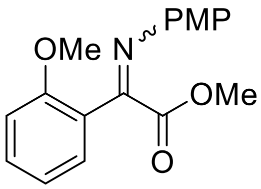

Methyl 2-(2-methoxyphenyl)-2-((4-methoxyphenyl)imino)acetate (1g)[3]

79% yield, yellow solid, 53:47 mixture of geometric isomers; ^{1}H NMR (400 MHz, Chloroform-*d*) ***Z*** isomer (major) *δ* 7.89 (dd, *J* = 7.6 Hz, 1.6 Hz, 1H), 7.29 (ddd, *J* = 8.4 Hz, 7.2 Hz, 2.0 Hz, 1H), 7.06 (td, *J* = 7.6 Hz, 1.2 Hz, 1H), 6.95-6.80 (m, 1H), 6.75 (d, *J* = 9.2 Hz, 2H), 6.69 (d, *J* = 9.2 Hz, 2H), 3.91 (s, 3H), 3.72 (s, 3H), 3.71 (s, 3H); minor isomer *δ* 7.45 (ddd, *J* = 8.4 Hz, 7.2 Hz, 1.6 Hz, 1H), 3.81 (s, 3H), 3.80 (s, 3H), 3.61 (s, 3H), other aromatic resonances are obscured; ^{13}C NMR (101 MHz, Chloroform-*d*) *δ* 166.0, 165.6, 158.4, 157.5, 157.49, 157.48, 157.2, 143.5, 141.8, 132.9, 130.9, 130.2, 129.7, 125.0, 123.3, 122.7, 121.4, 121.0, 120.6, 114.0, 113.6, 111.7, 111.1, 56.1, 55.6, 55.4, 55.2, 53.1, 51.7, other resonances of the minor isomer are obscured.

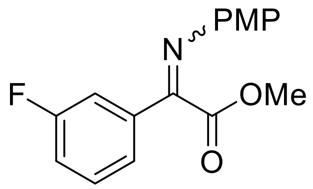

Methyl 2-(3-fluorophenyl)-2-((4-methoxyphenyl)imino)acetate (1h)[3]

56% yield, yellow liquid, 93:7 mixture of geometric isomers; ^{1}H NMR (400 MHz, Chloroform-*d*) ***Z*** isomer (major) δ 7.65-7.62 (m, 1H), 7.58 (d, J = 7.6 Hz, 1H), 7.44-7.38 (m, 1H), 7.21-7.16 (m, 1H), 6.97 (d, J = 8.8 Hz, 2H), 6.88 (d, J = 8.8 Hz, 2H), 3.80 (s, 3H), 3.70 (s, 3H); minor isomer δ 3.95 (s, 3H), 3.74 (s, 3H), aromatic resonances are obscured; ^{13}C NMR (101 MHz, Chloroform-*d*) δ 165.7, 162.9 (d, J = 247.5 Hz), 157.7, 157.6 (d, J = 3.0 Hz), 142.6, 136.4 (d, J = 7.1 Hz), 130.3 (d, J = 8.1 Hz), 123.7 (d, J = 3.0 Hz), 121.3, 118.5 (d, J = 21.2 Hz), 114.4 (d, J = 24.2 Hz), 114.3, 55.4, 52.1; resonances of the minor isomer are obscured.

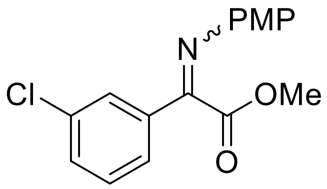

Methyl 2-(3-chlorophenyl)-2-((4-methoxyphenyl)imino)acetate (1i)

69% yield, yellow solid after being laid aside for a long time, melting point: 61.7-63.4 ^{o}C, 93:7 mixture of geometric isomers; ^{1}H NMR (400 MHz, Chloroform-*d*) ***Z*** isomer (major) δ 7.90 (t, J = 1.6 Hz, 1H), 7.70-7.67 (m, 1H), 7.47-7.44 (m, 1H), 7.37 (t, J = 8.0 Hz, 1H), 6.96 (d, J = 8.8 Hz, 2H), 6.88 (d, J = 8.8 Hz, 2H), 3.80 (s, 3H), 3.69 (s, 3H); minor isomer δ 7.30-7.29 (m, 1H), 7.20 (t, J = 8.0 Hz, 1H), 6.77-6.71 (m, 4H), 3.94 (s, 3H), 3.73 (s, 3H), other aromatic resonances are obscured; ^{13}C NMR (101 MHz, Chloroform-*d*) δ 165.9, 157.9, 157.6, 142.9, 136.1, 135.2, 131.7, 130.1, 129.3, 127.9, 127.4, 126.3, 123.6, 121.5, 114.5, 114.3, 55.6, 53.6, 52.4, other resonances of the minor isomer are obscured; HRMS (ESI-MS) Calcd. For $C_{16}H_{14}ClNO_3$ $[M+H]^+$ 304.0735, found: 304.0735.

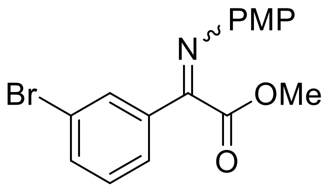

Methyl 2-(3-bromophenyl)-2-((4-methoxyphenyl)imino)acetate (1j)

65% yield, yellow solid after being laid aside for a long time, melting point: 79.0-80.4 ^{o}C, 93:7 mixture of geometric isomers; ^{1}H NMR (400 MHz, Chloroform-*d*) ***Z*** isomer (major) δ 8.06 (t, J =

2.0 Hz, 1H), 7.74-7.71 (m, 1H), 7.63-7.60 (m, 1H), 7.32 (t, *J* = 8.0 Hz, 1H), 6.96 (d, *J* = 8.8 Hz, 2H), 6.88 (d, *J* = 8.8 Hz, 2H), 3.81 (s, 3H), 3.70 (s, 3H); minor isomer *δ* 6.76-6.71 (m, 4H), 3.95 (s, 3H), 3.74 (s, 3H), other aromatic resonances are obscured; ^{13}C NMR (101 MHz, Chloroform-*d*) *δ* 165.6, 157.7, 157.3, 142.6, 136.1, 134.4, 132.6, 131.9, 130.6, 130.2, 129.9, 127.6, 126.5, 123.4, 123.0, 121.3, 114.3, 114.1, 55.4, 55.3, 53.4, 52.2, other resonances of the minor isomer are obscured; HRMS (ESI-MS) Calcd. For $C_{16}H_{14}BrNO_3$ $[M+H]^+$ 348.0230, found: 348.0230.

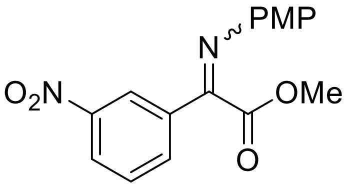

Methyl 2-((4-methoxyphenyl)imino)-2-(3-nitrophenyl)acetate (1k)[3]

75% yield, yellow solid after being laid aside for a long time, 94:6 mixture of geometric isomers; ^{1}H NMR (400 MHz, Chloroform-*d*) ***Z*** isomer (major) *δ* 8.72 (s, 1H), 8.33 (d, *J* = 6.8 Hz, 1H), 8.18 (d, *J* = 6.8 Hz, 1H), 7.73-7.54 (m, 1H), 7.00 (d, *J* = 8.8 Hz, 2H), 6.90 (d, *J* = 8.8 Hz, 2H), 3.82 (s, 3H), 3.76 (s, 3H); minor isomer *δ* 4.03 (s, 3H), 3.98 (s, 3H), aromatic resonances are obscured; ^{13}C NMR (101 MHz, Chloroform-*d*) *δ* 165.6, 158.3, 156.1, 148.7, 143.3, 136.2, 133.7, 130.0, 126.0, 122.9, 121.7, 114.6, 55.7, 52.7; resonances of minor isomer are obscured.

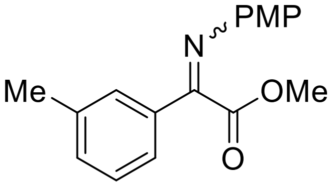

Methyl 2-((4-methoxyphenyl)imino)-2-(*m*-tolyl)acetate (1l)

68% yield, yellow solid after being laid aside for a long time, melting point: 62.8-64.2 ^{o}C, 94:6 mixture of geometric isomers; ^{1}H NMR (400 MHz, Chloroform-*d*) ***Z*** isomer (major) *δ* 7.69 (s, 1H), 7.61 (d, *J* = 7.2 Hz, 1H), 7.35-7.29 (m, 2H), 6.96 (d, *J* = 8.8 Hz, 2H), 6.87 (d, *J* = 8.8 Hz, 2H), 3.79 (s, 3H), 3.67 (s, 3H), 2.40 (s, 3H); minor isomer *δ* 6.76-6.69 (m, 4H), 3.94 (s, 3H), 3.72 (s, 3H), 2.27 (s, 3H), other aromatic resonances are obscured; ^{13}C NMR (101 MHz, Chloroform-*d*) *δ* 166.1, 165.7, 159.4, 158.8, 157.5, 157.3, 143.2, 141.3, 138.5, 138.1, 134.0, 133.0, 132.4, 130.4, 129.4, 128.6, 128.1, 126.1, 125.1, 123.2, 121.2, 114.2, 113.9, 55.4, 55.3, 53.2, 51.9, 21.4, other resonances of minor isomer are obscured; HRMS (ESI-MS) Calcd. For $C_{17}H_{17}NO_3$ $[M+H]^+$ 284.1281, found: 284.1282.

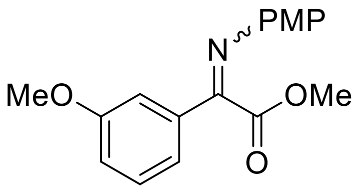

Methyl 2-(3-methoxyphenyl)-2-((4-methoxyphenyl)imino)acetate (1m)[3]

72% yield, yellow solid, 92:8 mixture of geometric isomers; ^{1}H NMR (400 MHz, Chloroform-*d*) ***Z*** isomer (major) *δ* 7.50 (s, 1H), 7.37-7.31 (m, 2H), 7.06-7.03 (m, 1H), 6.96 (d, *J* = 8.8 Hz, 2H), 6.87 (d, *J* = 8.8 Hz, 2H), 3.86 (s, 3H), 3.80 (s, 3H), 3.68 (s, 3H); minor isomer *δ* 7.20 (t, *J* = 8.0 Hz, 1H), 6.78-6.71 (m, 4H), 3.94 (s, 3H), 3.74 (s, 3H), 3.67 (s, 3H), other aromatic resonances are obscured; ^{13}C NMR (101 MHz, Chloroform-*d*) *δ* 166.2, 160.1, 159.3, 157.6, 143.3, 135.7, 129.9, 123.4, 121.4, 121.0, 118.5, 114.4, 114.2, 112.0, 55.7, 55.6, 52.2, other resonances of minor isomer are obscured.

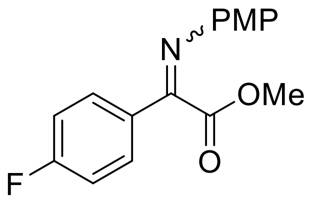

Methyl 2-(4-fluorophenyl)-2-((4-methoxyphenyl)imino)acetate (1n)[3]

63% yield, yellow liquid, 94:6 mixture of geometric isomers; ^{1}H NMR (400 MHz, Chloroform-*d*) ***Z*** isomer (major) *δ* 7.89-7.84 (m, 2H), 7.16-7.09 (m, 2H), 6.95 (d, *J* = 8.8 Hz, 2H), 6.88 (d, *J* = 8.8 Hz, 2H), 3.80 (s, 3H), 3.69 (s, 3H); minor isomer *δ* 8.13-8.05 (m, 1H), 3.95 (s, 3H), 3.74 (s, 3H), other aromatic resonances are obscured by the major isomer; ^{13}C NMR (101 MHz, Chloroform-*d*) *δ* 165.9, 164.8 (d, *J* = 254.5 Hz), 157.7, 157.4, 142.9, 133.1 (d, *J* = 10.1 Hz), 130.4 (d, *J* = 3.0 Hz), 130.1 (d, *J* = 9.1 Hz), 123.1, 121.2, 116.3 (d, *J* = 22.2 Hz), 115.9 (d, *J* = 22.2 Hz), 114.2, 114.0, 55.4, 55.3, 52.1, other resonances of minor isomer are obscured.

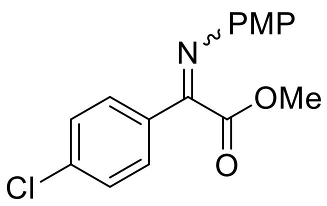

Methyl 2-(4-chlorophenyl)-2-((4-methoxyphenyl)imino)acetate (1o)[3]

85% yield, yellow solid, 93:7 mixture of geometric isomers; ^{1}H NMR (400 MHz, Chloroform-*d*) ***Z*** isomer (major) *δ* 7.79 (d, *J* = 8.4 Hz, 2H), 7.42 (d, *J* = 8.4 Hz, 2H), 6.96 (d, *J* = 8.8 Hz, 2H), 6.88 (d, *J* = 8.8 Hz, 2H), 3.80 (s, 3H), 3.69 (s, 3H); minor isomer *δ* 7.14 (d, *J* = 8.4 Hz, 2H), 6.73 (s,

4H), 3.95 (s, 3H), 3.74 (s, 3H), other aromatic resonances are obscured; ^{13}C NMR (101 MHz, Chloroform-*d*) *δ* 165.8, 165.2, 157.7, 157.6, 157.57, 157.2, 142.8, 141.0, 137.8, 135.7, 132.6, 131.4, 130.6, 129.1, 129.0, 128.7, 123.2, 121.2, 114.3, 114.1, 55.4, 55.3, 53.3, 52.1.

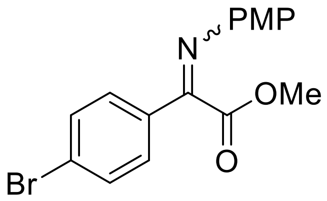

Methyl 2-(4-bromophenyl)-2-((4-methoxyphenyl)imino)acetate (1p)[3]

76% yield, yellow solid, 92:8 mixture of geometric isomers; ^{1}H NMR (400 MHz, Chloroform-*d*) ***Z*** isomer (major) *δ* 7.72 (d, *J* = 8.4 Hz, 2H), 7.58 (d, *J* = 8.4 Hz, 2H), 6.95 (d, *J* = 8.8 Hz, 2H), 6.87 (d, *J* = 8.8 Hz, 2H), 3.81 (s, 3H), 3.68 (s, 3H); minor isomer *δ* 7.43 (d, *J* = 8.4 Hz, 2H), 7.07 (d, *J* = 8.4 Hz, 2H), 6.73 (s, 4H), 3.94 (s, 3H), 3.74 (s, 3H); ^{13}C NMR (101 MHz, Chloroform-*d*) *δ* 165.7, 165.1, 157.7, 157.6, 157.3, 142.8, 140.9, 133.1, 131.9, 131.6, 130.8, 129.3, 126.3, 124.1, 123.2, 121.2, 114.2, 114.1, 55.4, 55.3, 53.3, 52.1, other resonances of minor isomer are obscured.

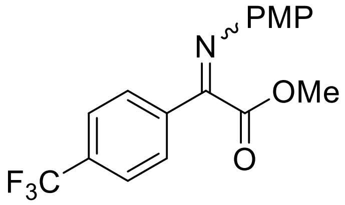

Methyl 2-((4-methoxyphenyl)imino)-2-(4-(trifluoromethyl)phenyl)acetate (1q)

81% yield, yellow solid, melting point: 89.4-91.0 °C, 92:8 mixture of geometric isomers; ^{1}H NMR (400 MHz, Chloroform-*d*) ***Z*** isomer (major) *δ* 7.98 (d, *J* = 8.8 Hz, 2H), 7.71 (d, *J* = 8.8 Hz, 2H), 6.99 (d, *J* = 9.2 Hz, 2H), 6.89 (d, *J* = 9.2 Hz, 2H), 3.81 (s, 3H), 3.72 (s, 3H); minor isomer *δ* 7.56 (d, *J* = 8.4 Hz, 2H), 7.32 (d, *J* = 8.4 Hz, 2H), 6.72 (s, 4H), 3.96 (s, 3H), 3.74 (s, 3H); ^{13}C NMR (101 MHz, Chloroform-*d*) *δ* 165.7, 165.0, 157.9, 156.86, 157.3, 142.6, 140.6, 137.4, 136.8, 132.9 (q, *J* = 32.3 Hz), 129.6, 128.2, 125.6 (q, *J* = 4.0 Hz), 125.3 (q, *J* = 4.0 Hz), 123.8 (q, *J* = 273.7 Hz),123.4, 121.3, 114.3, 114.1, 55.4, 55.3, 53.4, 52.2, other resonances of minor isomer are obscured; ^{19}F NMR (376 MHz, Chloroform-*d*) *δ* -62.94, -62.96; HRMS (ESI-MS) Calcd. For $C_{17}H_{14}F_3NO_3$ $[M+H]^+$ 338.0999, found: 338.0998.

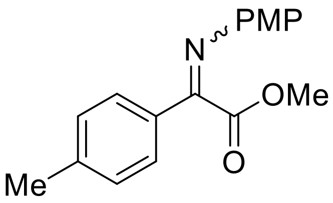

Methyl 2-((4-methoxyphenyl)imino)-2-(*p*-tolyl)acetate (1r)[3]

76% yield, yellow solid, 93:7 mixture of geometric isomers; ^{1}H NMR (400 MHz, Chloroform-*d*) ***Z*** isomer (major) *δ* 7.73 (d, *J* = 8.4 Hz, 2H), 7.24 (d, *J* = 8.4 Hz, 2H), 6.95 (d, *J* = 8.8 Hz, 2H), 6.86 (d, *J* = 8.8 Hz, 2H), 3.78 (s, 3H), 3.67 (s, 3H), 2.39 (s, 3H); minor isomer *δ* 6.76-7.70 (m, 4H), 3.93 (s, 3H), 3.72 (s, 3H), 2.30 (s, 3H), other aromatic resonances are obscured; ^{13}C NMR (101 MHz, Chloroform-*d*) *δ* 166.1, 165.7, 159.0, 158.7, 157.2, 157.1, 143.2, 142.0, 141.4, 139.7, 131.4, 129.8, 129.3, 129.0, 128.9, 127.7, 122.9, 121.1, 114.0, 113.8, 55.3, 55.2, 53.0, 51.8, 21.4, 21.3.

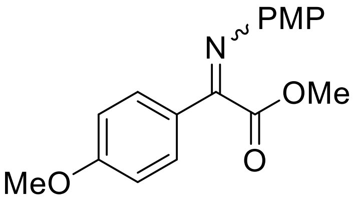

Methyl 2-(4-methoxyphenyl)-2-((4-methoxyphenyl)imino)acetate (1s)[3]

82% yield, yellow solid, 93:7 mixture of geometric isomers; ^{1}H NMR (400 MHz, Chloroform-*d*) ***Z*** isomer (major) *δ* 7.80 (d, *J* = 7.2 Hz, 2H), 6.96-6.93 (m, 4H), 6.86 (d, *J* = 7.2 Hz, 2H), 3.85 (s, 3H), 3.79 (s, 3H), 3.67 (s, 3H); minor isomer *δ* 7.15 (d, *J* = 7.2 Hz, 2H), 6.79-7.72 (m, 6H), 3.95 (s, 3H), 3.77 (s, 3H), 3.74 (s, 3H); ^{13}C NMR (101 MHz, Chloroform-*d*) *δ* 166.3, 165.9, 162.4, 160.5, 158.6, 158.3, 157.2, 157.1, 143.4, 141.8, 131.1, 129.6, 126.9, 124.7, 122.8, 121.2, 114.2, 114.1, 114.0, 113.7, 55.4, 55.38, 55.3, 55.2, 53.2, 51.9.

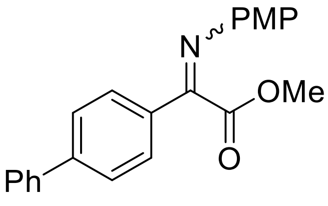

Methyl 2-([1,1'-biphenyl]-4-yl)-2-((4-methoxyphenyl)imino)acetate (1t)

76% yield, yellow solid, melting point: 116.9-118.2 ^{o}C, 93:7 mixture of geometric isomers; ^{1}H NMR (400 MHz, Chloroform-*d*) ***Z*** isomer (major) *δ* 7.92 (d, *J* = 8.4 Hz, 2H), 7.67 (d, *J* = 8.4 Hz, 2H), 7.62 (d, *J* = 7.2 Hz, 2H), 7.49-7.42 (m, 2H), 7.40-7.34 (m, 1H), 6.99 (d, *J* = 8.8 Hz, 2H), 6.88 (d, *J* = 8.8 Hz, 2H), 3.79 (s, 3H), 3.70 (s, 3H); minor isomer *δ* 7.27 (d, *J* = 8.4 Hz, 2H), 6.79

(d, J = 8.8 Hz, 2H), 6.73 (d, J = 8.8 Hz, 2H), 3.96 (s, 3H), 3.72 (s, 3H), other resonances are obscured by the major isomer; ^{13}C NMR (101 MHz, Chloroform-*d*) δ 166.4, 159.0, 157.7, 144.5, 143.5, 140.3, 133.3, 130.0, 129.2, 128.6, 128.3, 127.6, 127.4, 127.1, 123.4, 121.5, 114.5, 114.3, 55.7, 55.5, 53.6, 52.3, other resonances of minor isomer are obscured; HRMS (ESI-MS) Calcd. For $C_{22}H_{19}NO_3$ $[M+H]^+$ 346.1438, found: 346.1436.

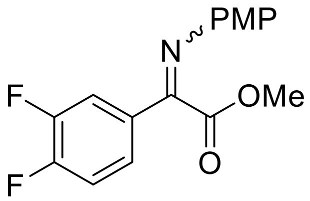

Methyl 2-(3,4-difluorophenyl)-2-((4-methoxyphenyl)imino)acetate (1u)

57% yield, yellow liquid, 93:7 mixture of geometric isomers; ^{1}H NMR (400 MHz, Chloroform-*d*) ***Z*** isomer (major) δ 7.82-7.76 (m, 1H), 7.56-7.53 (m, 1H), 7.24-7.19 (m, 1H), 6.95 (d, J = 8.8 Hz, 2H), 6.88 (d, J = 8.8 Hz, 2H), 3.81 (s, 3H), 3.70 (s, 3H); minor isomer δ 6.76-6.71 (m, 4H), 3.98 (s, 3H), 3.76 (s, 3H), other resonances are obscured; ^{13}C NMR (101 MHz, Chloroform-*d*) δ 165.5, 157.7, 156.4 (t, J = 2.0 Hz), 152.5 (dd, J = 255.5 Hz, 13.0 Hz), 150.6 (dd, J = 251.5 Hz, 13.2 Hz), 142.5, 131.4 (dd, J = 6.1 Hz, 4.0 Hz), 124.6 (q, J = 4.0 Hz), 121.2, 117.5 (d, J = 18.3 Hz), 116.7 (d, J = 19.2 Hz), 114.3, 55.4, 52.3; resonances of the minor isomer are obscured; ^{19}F NMR (376 MHz, Chloroform-*d*) δ -132.37 (d, J = 21.1 Hz), -134.56 (d, J = 20.7 Hz), -134.95 (d, J = 20.7 Hz), -136.21 (d, J = 21.1 Hz); HRMS (ESI-MS) Calcd. For $C_{16}H_{13}$ F_2NO_3 $[M+H]^+$ 306.0936, found: 306.0938.

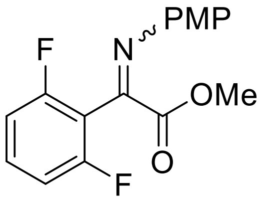

Methyl 2-(2,6-difluorophenyl)-2-((4-methoxyphenyl)imino)acetate (1v)

79% yield, yellow solid after being laid aside for a long time, melting point: 38.0-39.9 ^{o}C, 97:3 mixture of geometric isomers; ^{1}H NMR (400 MHz, Chloroform-*d*) ***Z*** isomer (major) δ 7.36-7.28 (m, 1H), 6.87-6.79 (m, 4H), 6.73 (d, J = 8.8 Hz, 2H), 3.96 (s, 3H), 3.73 (s, 3H); minor isomer δ 3.81 (s, 3H), 3.65 (s, 3H), other resonances are obscured; ^{13}C NMR (101 MHz, Chloroform-*d*) δ 163.7, 159.5 (dd, J = 250.5 Hz, 8.1 Hz), 158.3, 148.8, 141.4, 131.8 (t, J = 10.1 Hz), 122.0, 113.9, 111.9 (t, J = 22.2 Hz), 111.5 (m), 55.3, 53.5; resonances of the minor isomer are obscured;

^{19}F NMR (376 MHz, Chloroform-*d*) δ -109.77, -111.87; HRMS (ESI-MS) Calcd. For $C_{16}H_{13}F_2NO_3$ $[M+H]^+$ 306.0936, found: 306.0939.

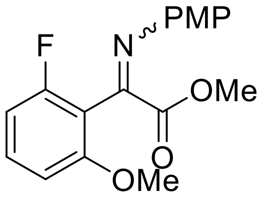

Methyl 2-(2-fluoro-6-methoxyphenyl)-2-((4-methoxyphenyl)imino)acetate (1w)

83% yield, yellow solid after being laid aside for a long time, melting point: 107.5-109.0 °C, 98:2 mixture of geometric isomers; ^{1}H NMR (400 MHz, Chloroform-*d*) ***Z*** isomer (major) δ 7.29-7.23 (m, 1H), 6.79 (d, *J* = 8.8 Hz, 2H), 6.70 (d, *J* = 8.8 Hz, 2H), 6.63-6.57 (m, 2H), 3.92 (s, 3H), 3.72 (s, 3H), 3.69 (s, 3H); minor isomer 3.82 (s, 3H), 3.80 (s, 3H), 3.60 (s, 3H), other resonances are obscured; ^{13}C NMR (101 MHz, Chloroform-*d*) δ 164.5, 159.5 (d, *J* = 248.5 Hz), 158.1 (d, *J* = 8.1 Hz), 157.8, 152.0, 142.1, 131.6 (d, *J* = 10.1 Hz), 121.8, 113.5, 112.0 (d, *J* = 21.2 Hz), 108.1 (d, *J* = 22.2 Hz), 106.5 (d, *J* = 3.0 Hz), 56.0, 55.2, 53.3; resonances of the minor isomer are obscure; ^{19}F NMR (376 MHz, Chloroform-*d*) δ -111.54, -114.19; HRMS (ESI-MS) Calcd. For $C_{17}H_{16}FNO_4$ $[M+H]^+$ 318.1136, found: 318.1137.

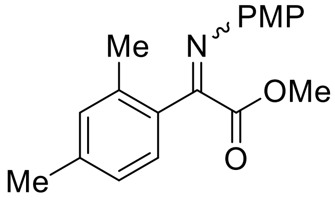

Methyl 2-(2,4-dimethylphenyl)-2-((4-methoxyphenyl)imino)acetate (1x)

69% yield, yellow solid after being laid aside for a long time, melting point: 68.8-71.4 °C, 81:19 mixture of geometric isomers; ^{1}H NMR (400 MHz, Chloroform-*d*) ***Z*** isomer (major) δ 7.26 (s, 1H), 6.99-6.93 (m, 2H), 6.77 (d, *J* = 9.2 Hz, 2H), 6.68 (d, *J* = 9.2 Hz, 2H), 3.92 (s, 3H), 3.73 (s, 3H), 2.30 (s, 3H), 2.02 (s, 3H); minor isomer δ 3.81 (s, 3H), 3.62 (s, 3H), 2.49 (s, 3H), 2.35 (s, 3H), other resonances are obscured; ^{13}C NMR (101 MHz, Chloroform-*d*) δ 166.1, 165.7, 160.5, 159.2, 157.9, 157.2, 143.5, 141.0, 140.7, 139.2, 137.7, 135.3, 132.5, 131.6, 131.2, 131.2, 129.5, 128.3, 126.6, 124.4, 124.0, 120.8, 114.2, 113.7, 55.4, 55.2, 53.2, 51.9, 21.3, 20.8, 19.7, 19.6; HRMS (ESI-MS) Calcd. For $C_{18}H_{19}NO_3$ $[M+H]^+$ 298.1438, found: 298.1439.

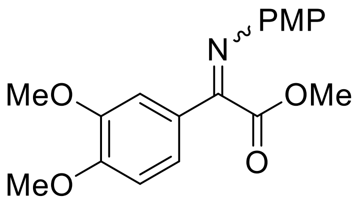

Methyl 2-(3,4-dimethoxyphenyl)-2-((4-methoxyphenyl)imino)acetate (1y)

72% yield, yellow solid, melting point: 143.6-145.0 °C, 96:4 mixture of geometric isomers; ^{1}H NMR (400 MHz, Chloroform-*d*) ***Z*** isomer (major) *δ* 7.62 (d, *J* = 2.4 Hz, 1H), 7.22 (dd, *J* = 8.4 Hz, 2.4 Hz, 1H), 6.95 (d, *J* = 8.8 Hz, 2H), 6.90-6.85 (m, 3H), 3.96 (s, 3H), 3.94 (s, 3H), 3.80 (s, 3H), 3.67 (s, 3H); minor isomer 3.86 (s, 3H), 3.75 (s, 3H), 3.61 (s, 3H), other resonances are obscured; ^{13}C NMR (101 MHz, Chloroform-*d*) *δ* 166.2, 158.8, 157.1, 152.2, 149.4, 143.3, 127.1, 122.5, 121.1, 114.2, 110.3, 109.2, 56.0, 55.4, 51.9, resonances of the minor isomer are obscured; HRMS (ESI-MS) Calcd. For $C_{18}H_{19}NO_5$ $[M+H]^+$ 330.1336, found: 330.1332.

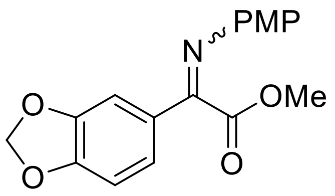

Methyl 2-(benzo[*d*][1,3]dioxol-5-yl)-2-((4-methoxyphenyl)imino)acetate (1z)

77% yield, yellow solid, melting point: 118.1-120.7 °C, 96:4 mixture of geometric isomers; ^{1}H NMR (400 MHz, Chloroform-*d*) ***Z*** isomer (major) *δ* 7.48 (d, *J* = 1.6 Hz, 1H), 7.23 (dd, *J* = 8.4 Hz, 1.6 Hz, 1H), 6.93 (d, *J* = 9.2 Hz, 1H), 6.88-6.82 (m, 4H), 6.01 (s, 2H), 3.79 (s, 3H), 3.66 (s, 3H); minor isomer *δ* 5.93 (s, 2H), 3.93 (s, 3H), 3.74 (s, 3H), other resonances are obscured; ^{13}C NMR (101 MHz, Chloroform-*d*) *δ* 166.3, 158.5, 157.4, 150.9, 148.6, 143.4, 129.0, 124.0, 121.4, 114.4, 108.3, 107.4, 102.0, 55.6, 52.2, resonances of the minor isomer are obscured; HRMS (ESI-MS) Calcd. For $C_{17}H_{15}NO_5$ $[M+H]^+$ 314.1023, found: 314.1021.

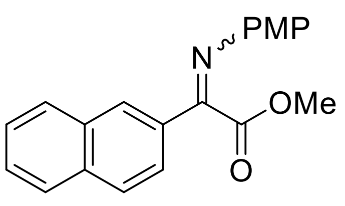

Methyl 2-((4-methoxyphenyl)imino)-2-(naphthalen-2-yl)acetate (1aa)[3]

83% yield, yellow solid, 92:8 mixture of geometric isomers; ^{1}H NMR (400 MHz, Chloroform-*d*) ***Z*** isomer (major) *δ* 8.23-8.03 (m, 2H), 7.96-7.78 (m, 3H), 7.53-7.49 (m, 2H), 7.02 (d, *J* = 6.4 Hz, 2H), 6.89 (d, *J* = 10.4 Hz, 2H), 3.78 (s, 3H), 3.72 (s, 3H); minor isomer 3.95 (s, 3H), 3.66 (s, 3H), other resonances are obscured; ^{13}C NMR (101 MHz, Chloroform-*d*) *δ* 166.5, 159.3, 157.7, 143.5,

135.2, 133.1, 131.9, 129.6, 129.3, 128.9, 128.1, 127.0, 124.0, 121.5, 114.5, 55.6, 52.3, resonances of the minor isomer are obscured.

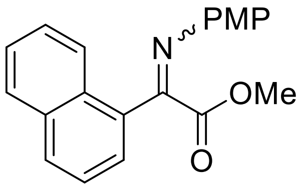

Methyl 2-((4-methoxyphenyl)imino)-2-(naphthalen-1-yl)acetate (1ab)[3]

76% yield, yellow solid, 78:22 mixture of geometric isomers; ^{1}H NMR (400 MHz, Chloroform-*d*) ***Z*** isomer (major) δ 7.85-7.81 (m, 2H), 7.67-7.62 (m, 1H), 7.46-7.42 (m, 2H), 7.41-7.37 (m, 1H), 7.26-7.24 (m, 1H), 6.74 (d, J = 8.8 Hz, 2H), 6.54 (d, J = 8.8 Hz, 2H), 3.88 (s, 3H), 3.60 (s, 3H); minor isomer δ 8.73 (d, J = 8.4 Hz, 1H), 7.94 (d, J = 8.4 Hz, 1H), 7.89-7.86 (m, 1H), 7.72 (dd, J = 7.2 Hz, 1.2 Hz, 1H), 7.05 (d, J = 8.8 Hz, 2H), 6.92 (d, J = 8.8 Hz, 2H), 3.81 (s, 3H), 3.65 (s, 3H), other resonances are obscured by the major isomer; ^{13}C NMR (101 MHz, Chloroform-*d*) δ 165.9, 165.6, 160.3, 158.5, 158.0, 157.5, 143.3, 141.0, 134.1, 133.3, 132.6, 132.0, 131.6, 130.9, 130.5, 129.6, 128.7, 128.6, 128.4, 127.6, 127.1, 126.7, 126.4, 126.37, 125.4, 125.2, 124.8, 124.6, 123.7, 121.0, 114.3, 113.7, 55.5, 55.2, 53.3, 52.1.

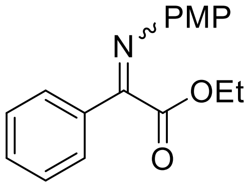

Ethyl 2-((4-methoxyphenyl)imino)-2-phenylacetate (1ac)[3]

83% yield, yellow solid, 95:5 mixture of geometric isomers; ^{1}H NMR (400 MHz, Chloroform-*d*) ***Z*** isomer (major) δ 7.87 (d, J = 6.8 Hz, 2H), 7.52-7.41 (m, 3H), 6.96 (d, J = 8.8 Hz, 2H), 6.87 (d, J = 8.8 Hz, 2H), 4.18 (q, J = 7.2 Hz, 2H), 3.80 (s, 3H), 1.08 (t, J = 7.2 Hz, 3H); minor isomer δ 7.32-7.26 (m, 3H), 7.21-7.17 (m, 2H), 6.75-6.69 (m, 4H), 4.42 (q, J = 7.2 Hz, 2H), 3.73 (s, 3H), 1.40 (t, J = 7.2 Hz, 3H); ^{13}C NMR (101 MHz, Chloroform-*d*) δ 165.5, 159.7, 157.3, 143.3, 134.1, 131.5, 129.5, 129.1, 128.7, 128.3, 127.8, 123.1, 121.2, 114.1, 113.9, 62.4, 61.4, 55.5, 55.3, 14.2, 13.9, other resonances of minor isomer are obscured.

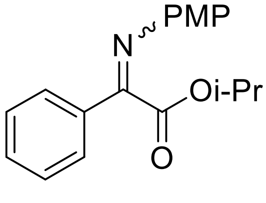

Isopropyl 2-((4-methoxyphenyl)imino)-2-phenylacetate (1ad)[4]

79% yield, yellow solid, 97:3 mixture of geometric isomers; ^{1}H NMR (400 MHz, Chloroform-*d*) ***Z*** isomer (major) *δ* 7.87 (d, *J* = 6.4 Hz, 2H), 7.51-7.43 (m, 3H), 6.96 (d, *J* = 8.8 Hz, 2H), 6.86 (d, *J* = 8.8 Hz, 2H), 5.13-5.04 (m, 1H), 3.79 (s, 3H), 1.07 (d, *J* = 6.4 Hz, 6H); minor isomer *δ* 3.72 (s, 3H), 1.36 (d, *J* = 6.4 Hz, 6H), other resonances are obscured; ^{13}C NMR (101 MHz, Chloroform-*d*) *δ* 165.0, 160.0, 157.3, 143.4, 134.2, 131.5, 128.7, 127.8, 121.2, 114.1, 69.6, 55.5, 21.5, resonances of the minor isomer are obscured.

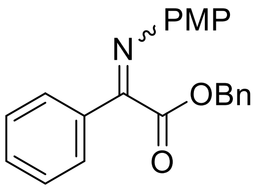

Benzyl 2-((4-methoxyphenyl)imino)-2-phenylacetate (1ae)[4]

75% yield, yellow solid, 97:3 mixture of geometric isomers; ^{1}H NMR (400 MHz, Chloroform-*d*) ***Z*** isomer (major) *δ* 7.84 (d, *J* = 6.8 Hz, 2H), 7.50-7.39 (m, 3H), 7.30-7.23 (m, 3H), 7.08 (dd, *J* = 7.6 Hz, 1.6 Hz, 2H), 6.91 (d, *J* = 8.8 Hz, 2H), 6.76 (d, *J* = 8.8 Hz, 2H), 5.13 (s, 2H), 3.76 (s, 3H); minor isomer *δ* 5.38 (s, 2H), 3.70 (s, 3H), other resonances are obscured; ^{13}C NMR (101 MHz, Chloroform-*d*) *δ* 165.4, 159.1, 157.4, 143.1, 134.5, 134.2, 131.6, 128.7, 128.5, 127.9, 121.3, 114.2, 67.2, 55.4.

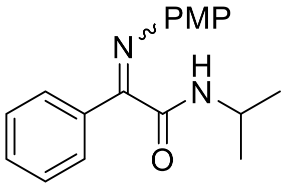

***N*-isopropyl-2-((4-methoxyphenyl)imino)-2-phenylacetamide (1af)**

56% yield, yellow solid, melting point: 184.3-185.8 ^{o}C; 57:43 mixture of geometric isomers; ^{1}H NMR (400 MHz, Chloroform-*d*) ***Z*** isomer (major) *δ* 7.54 (d, *J* = 8.0 Hz, 1H), 7.31-7.24 (m, 3H), 7.19 (d, *J* = 6.0 Hz, 2H), 6.73-6.67 (m, 4H), 4.22-4.12 (m, 1H), 3.74 (s, 3H), 1.28 (s, 3H), 1.27 (s, 3H); minor isomer *δ* 7.94 (d, *J* = 6.4 Hz, 2H), 7.49-7.41 (m, 3H), 7.02 (d, *J* = 8.8 Hz, 2H), 6.87 (d, *J* = 8.8 Hz, 2H), 5.23 (d, *J* = 8.4 Hz, 1H), 4,12-4.03 (m, 1H), 3.80 (s, 3H), 0.92 (s, 3H), 0.90 (s, 3H); ^{13}C NMR (101 MHz, Chloroform-*d*) *δ* 165.0, 163.1, 163.05, 160.6, 157.4, 157.3, 143.0, 141.1, 134.8, 132.4, 131.4, 129.6, 129.1, 128.6, 128.2, 127.9, 123.3, 121.6, 114.2, 114.0, 55.5, 55.3, 41.7, 41.3, 22.7, 22.3. HRMS (ESI-MS) Calcd. For $C_{18}H_{20}N_{2}O_{2}$ $[M+H]^{+}$ 297.1598, found: 297.1597.

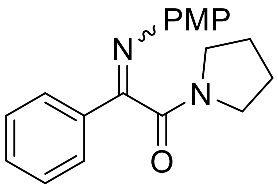

2-((4-methoxyphenyl)imino)-2-phenyl-1-(pyrrolidin-1-yl)ethan-1-one (1ag)

73% yield, yellow solid, melting point: 96.7-97.6 ^{o}C; ^{1}H NMR (400 MHz, Chloroform-*d*) *δ* 7.91 (d, *J* = 7.6 Hz, 2H), 7.49-7.41 (m, 3H), 7.15 (d, *J* = 8.8 Hz, 2H), 6.87 (d, *J* = 8.8 Hz, 2H), 3.80 (s, 3H), 3.57-2.85 (m, 4H), 1.79-1.49 (m, 4H); ^{13}C NMR (101 MHz, Chloroform-*d*) *δ* 165.9, 162.3, 157.7, 142.4, 135.0, 131.5, 129.0, 128.1, 122.7, 114.3, 55.6, 46.5, 44.9, 25.7, 24.3; HRMS (ESI-MS) Calcd. For $C_{19}H_{20}N_2O_2$ $[M+H]^+$ 309.1598, found: 309.1595.

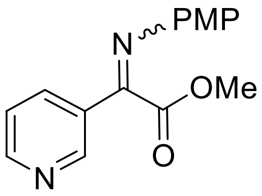

Methyl 2-((4-methoxyphenyl)imino)-2-(pyridin-3-yl)acetate (1ah)

62% yield, yellow liquid, 90:10 mixture of geometric isomers; ^{1}H NMR (400 MHz, Chloroform-*d*) ***Z*** isomer (major) *δ* 8.99 (dd, *J* = 2.0 Hz, 0.8 Hz, 1H), 8.68 (dd, *J* = 4.8 Hz, 2.0 Hz, 1H), 8.22-8.11 (m, 1H), 7.36 (ddd, *J* = 8.0 Hz, 4.8 Hz, 0.8 Hz, 1H), 6.95 (d, *J* = 8.8 Hz, 2H), 6.86 (d, *J* = 8.8 Hz, 2H), 3.78 (s, 3H), 3.69 (s, 3H); minor isomer *δ* 6.70 (s, 4H), 3.94 (s, 3H), 3.70 (s, 3H), other resonances are obscured; ^{13}C NMR (101 MHz, Chloroform-*d*) *δ* 165.3, 157.8, 156.3, 152.0, 149.3, 142.6, 134.9, 130.1, 123.5, 121.3, 114.3, 55.4, 52.3, resonances of the minor isomer are obscured. HRMS (ESI-MS) Calcd. For $C_{15}H_{14}N_2O_3$ $[M+H]^+$ 271.1077, found: 271.1088.

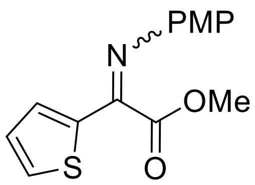

Methyl 2-((4-methoxyphenyl)imino)-2-(thiophen-2-yl)acetate (1ai)[5]

74% yield, yellow solid, 94:6 mixture of geometric isomers; ^{1}H NMR (400 MHz, Chloroform-*d*) ***Z*** isomer (major) *δ* 7.52 (d, *J* = 5.6 Hz, 1H), 7.40 (d, *J* = 2.4 Hz, 1H), 7.12-7.08 (m, 1H), 6.98 (d, *J* = 8.8 Hz, 2H), 6.88 (d, *J* = 8.8 Hz, 2H), 3.79 (s, 3H), 3.71 (s, 3H); minor isomer *δ* 4.02 (s, 3H), 4.00 (s, 3H), other resonances are obscured; ^{13}C NMR (101 MHz, Chloroform-*d*) *δ* 165.7, 160.2, 150.3, 134.0, 132.0, 129.1, 129.0, 128.2, 125.2, 119.7, 55.6, 52.5, resonances of the minor isomer are obscured.

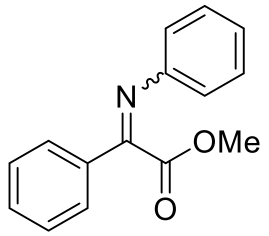

Methyl 2-phenyl-2-(phenylimino)acetate (1aj)[6]

64% yield, yellow liquid, 96:4 mixture of geometric isomers; ^{1}H NMR (400 MHz, Chloroform-*d*) ***Z*** isomer (major) *δ* 7.88-7.85 (m, 2H), 7.54-7.46 (m, 3H), 7.33 (t, *J* = 7.6 Hz, 2H), 7.15 (t, *J* = 7.6 Hz, 1H), 6.97-6.94 (m, 2H), 3.64 (s, 3H); minor isomer *δ* 3.99 (s, 3H), other resonances are obscured; ^{13}C NMR (101 MHz, Chloroform-*d*) *δ* 165.7, 160.2, 150.3, 134.0, 132.0, 129.1, 129.0, 128.2, 125.2, 119.7, 52.1, resonances of the minor isomer are obscured.

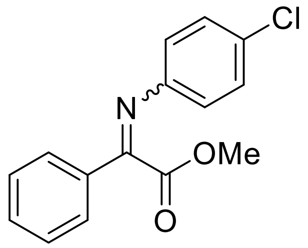

Methyl 2-((4-chlorophenyl)imino)-2-phenylacetate (1ak)

69% yield, yellow liquid, 90:10 mixture of geometric isomers; ^{1}H NMR (400 MHz, Chloroform-*d*) ***Z*** isomer (major) *δ* 7.84 (d, *J* = 8.4 Hz, 2H), 7.55-7.41 (m, 3H), 7.29 (d, *J* = 8.8 Hz, 2H), 6.89 (d, *J* = 8.8 Hz, 2H), 3.66 (s, 3H); minor isomer *δ* 3.97 (s, 3H), other resonances are obscured; ^{13}C NMR (101 MHz, Chloroform-*d*) *δ* 165.2, 160.5, 148.6, 135.0, 133.6, 132.1, 130.4, 130.1, 129.1, 129.0, 128.9, 128.8, 128.3, 128.1, 122.0, 121.0, 116.3, 52.8, 52.0; other resonances of the minor isomer are obscured; HRMS (ESI-MS) Calcd. For $C_{15}H_{12}ClNO_2$ $[M+H]^+$ 274.0629, found: 274.0641.

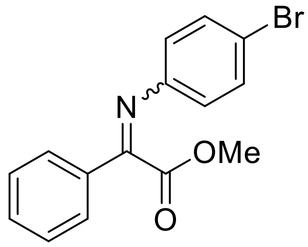

Methyl 2-((4-bromophenyl)imino)-2-phenylacetate (1al)

73% yield, yellow liquid, 90:10 mixture of geometric isomers; ^{1}H NMR (400 MHz, Chloroform-*d*) ***Z*** isomer (major) *δ* 7.84 (d, *J* = 6.8 Hz, 2H), 7.54-7.47 (m, 1H), 7.47-7.42 (m, 4H), 6.83 (d, *J* = 8.8 Hz, 2H), 3.66 (s, 3H); minor isomer *δ* 3.97 (s, 3H), other resonances are obscured; ^{13}C NMR

(101 MHz, Chloroform-*d*) δ 165.1, 160.5, 149.0, 135.0, 133.5, 132.1, 131.9, 130.1, 128.9, 128.8, 128.1, 122.3, 121.4, 118.2, 116.8, 52.8, 52.1, other resonances of the minor isomer are obscured; HRMS (ESI-MS) Calcd. For $C_{15}H_{12}BrNO_2$ $[M+H]^+$ 318.0124, found: 318.0127.

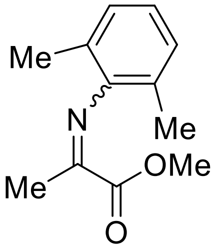

Methyl 2-((2,6-dimethylphenyl)imino)propanoate (1am)[6]

68% yield, yellow liquid, 94:6 mixture of geometric isomers; ^{1}H NMR (400 MHz, Chloroform-*d*) ***Z*** isomer (major) δ 7.00 (d, J = 6.8 Hz, 2H), 6.93-6.89 (m, 1H), 3.93 (s, 3H), 1.97 (s, 6H), 1.90 (s, 3H); minor isomer 3.84 (s, 3H), 2.44 (s, 3H), 2.15 (s, 6H) other resonances are obscured; ^{13}C NMR (101 MHz, Chloroform-*d*) δ 164.9, 161.0, 147.0, 128.0, 124.5, 123.9, 53.1, 17.7, 17.2, resonances of the minor isomer are obscured.

Asymmetric hydrogenation of *N*-aryl imino esters/amides

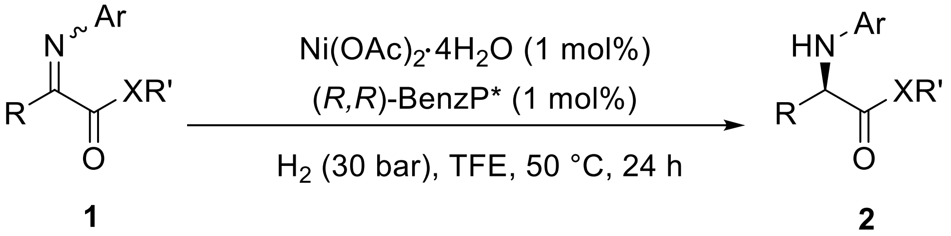

Procedure D: To a hydrogenation tube, $Ni(OAc)_2 \cdot 4H_2O$ (0.75 mg, 0.003 mmol), (*R,R*)-BenzP* (0.85 mg, 0.003 mmol) and the substrate (S/C = 100) were added, and then the mixture was transferred to a nitrogen-filled glovebox. The degassed and anhydrous trifluoroethanol (TFE, 1.0 mL) was added. The reaction was performed with H_2 (30 bar) at 50 ℃ for 24 h. After carefully releasing hydrogen gas, the pure product is obtained by column chromatography (PE/EtOAc). The enantiomeric excess was determined by chiral HPLC.

Note: The reaction temperature in the above procedure for substrates **1k** and **1af** was 70 ^{o}C.

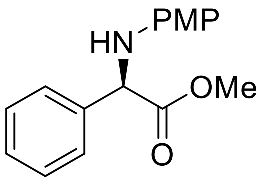

Methyl (*R*)-2-((4-methoxyphenyl)amino)-2-phenylacetate (2a)[3]

White solid, 99% yield, 96% ee, $[\alpha]_D^{20}$ = -95.2 (c = 1.08, CH_2Cl_2); ^{1}H NMR (400 MHz, Chloroform-*d*) δ 7.48 (d, *J* = 7.2 Hz, 2H), 7.40-7.26 (m, 3H), 6.71 (d, *J* = 8.8 Hz, 2H), 6.52 (d, *J* = 8.8 Hz, 2H), 5.01 (s, 1H), 4.67 (s, 1H), 3.70 (s, 3H), 3.69 (s, 3H); ^{13}C NMR (101 MHz, Chloroform-*d*) δ 172.8, 152.8, 140.5, 138.1, 129.1, 128.5, 127.5, 115.1, 115.0, 61.9, 55.9, 53.0; HPLC conditions: DAICEL Chiralpak IE column, *n*-Hexane/*i*-PrOH = 95/5, 254 nm, 0.8 mL/min, t_{major} = 20.944 min, t_{minor} = 17.205 min.

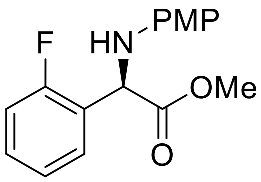

Methyl (*R*)-2-(2-fluorophenyl)-2-((4-methoxyphenyl)amino)acetate (2b)[3]

Colorless liquid, 99% yield, 98% ee, $[\alpha]_D^{20}$ = -98.7 (c = 0.76, CH_2Cl_2); ^{1}H NMR (400 MHz, Chloroform-*d*) δ 7.42 (td, *J* = 7.6 Hz, 2.0 Hz, 1H), 7.31-7.24 (m, 1H), 7.14-7.05 (m, 2H), 6.72 (d,

J = 8.8 Hz, 2H), 6.56 (d, J = 8.8 Hz, 2H), 5.38 (s, 1H), 4.76 (s, 1H), 3.71 (s, 3H), 3.69 (s, 3H); ^{13}C NMR (101 MHz, Chloroform-*d*) δ 172.0, 160.8 (d, J = 248.5 Hz), 152.7, 139.8, 129.9 (d, J = 8.3 Hz), 128.3 (d, J = 3.5 Hz), 125.4 (d, J = 13.8 Hz), 124.7 (d, J = 3.4 Hz), 115.8 (d, J = 21.8 Hz), 114.9, 55.7, 54.7 (d, J = 3.0 Hz), 52.9; ^{19}F NMR (376 MHz, Chloroform-*d*) δ -118.48; HPLC conditions: DAICEL Chiralpak IE column, *n*-Hexane/*i*-PrOH = 95/5, 254 nm, 0.8 mL/min, t_{major} = 18.760 min, t_{minor} = 16.437 min.

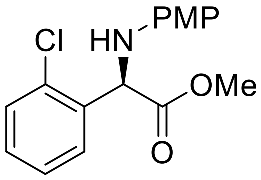

Methyl (*R*)-2-(2-chlorophenyl)-2-((4-methoxyphenyl)amino)acetate (2c)

Colorless liquid, 98% yield, 96% ee, $[\alpha]_D^{20}$ = -102.8 (c = 1.39, CH_2Cl_2); ^{1}H NMR (400 MHz, Chloroform-*d*) δ 7.46-7.44 (m, 1H), 7.41-7.39 (m, 1H), 7.23-7.20 (m, 2H), 6.71 (d, J = 8.8 Hz, 2H), 6.53 (d, J = 8.8 Hz, 2H), 5.54 (s, 1H), 4.79 (s, 1H), 3.70 (s, 3H), 3.68 (s, 3H); ^{13}C NMR (101 MHz, Chloroform-*d*) δ 172.3, 152.9, 140.1, 136.1, 134.4, 130.2, 129.6, 128.5, 127.7, 115.1, 115.0, 58.2, 55.9, 53.1; HPLC conditions: DAICEL Chiralpak IE column, *n*-Hexane/*i*-PrOH = 95/5, 254 nm, 0.8 mL/min, t_{major} = 17.356 min, t_{minor} = 13.386 min; HRMS (ESI-MS) Calcd. For $C_{16}H_{16}ClNO_3$ $[M+H]^+$ 306.0891, found: 306.0890.

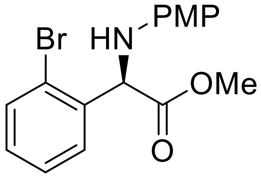

Methyl (*R*)-2-(2-bromophenyl)-2-((4-methoxyphenyl)amino)acetate (2d)

Colorless liquid, 98% yield, 94% ee, $[\alpha]_D^{20}$ = -35.2 (c = 1.45, CH_2Cl_2); ^{1}H NMR (400 MHz, Chloroform-*d*) δ 7.59 (dd, J = 8.0 Hz, 1.6 Hz, 1H), 7.45 (dd, J = 8.0 Hz, 1.6 Hz, 1H), 7.28-7.24 (m, 1H), 7.16-7.12 (m, 1H), 6.71 (d, J = 8.8 Hz, 2H), 6.53 (d, J = 8.8 Hz, 2H), 5.53 (s, 1H), 4.81 (s, 1H), 3.71 (s, 3H), 3.68 (s, 3H); ^{13}C NMR (101 MHz, Chloroform-*d*) δ 172.0, 152.7, 139.8, 137.5, 133.3, 129.7, 128.4, 128.1, 124.6, 114.9, 60.4, 55.7, 52.9; HPLC conditions: DAICEL Chiralpak IE column, *n*-Hexane/*i*-PrOH = 90/10, 254 nm, 0.8 mL/min, t_{major} = 12.954 min, t_{minor} = 10.477 min; HRMS (ESI-MS) Calcd. For $C_{16}H_{16}BrNO_3$ $[M+H]^+$ 350.0386, found: 350.0385.

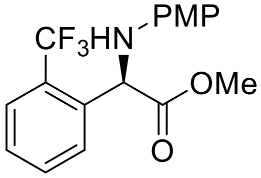

Methyl (*R*)-2-((4-methoxyphenyl)amino)-2-(2-(trifluoromethyl)phenyl)acetate (2e)

Colorless liquid, 97% yield, 92% ee, $[\alpha]_D^{20}$ = -77.7 (c =1.31, CH_2Cl_2); ^{1}H NMR (400 MHz, Chloroform-*d*) δ 7.70 (t, *J* = 8.8 Hz, 2H), 7.52 (t, *J* = 7.2 Hz, 1H), 7.41 (t, *J* = 8.0 Hz, 1H), 6.72 (d, *J* = 8.8 Hz, 2H), 6.60 (d, *J* = 8.8 Hz, 2H), 5.46 (s, 1H), 4.54 (s, 1H), 3.69 (s, 6H); ^{13}C NMR (101 MHz, Chloroform-*d*) δ 172.3, 153.1, 140.0, 136.7 (d, *J* = 2.0 Hz), 132.6, 128.8 (q, *J* = 30.3 Hz), 128.5, 128.4, 126.6 (q, *J* = 5.1 Hz), 124.3 (q, *J* = 275.7 Hz), 115.5, 114.8, 57.8 (q, *J* = 4.0 Hz), 55.6, 52.9; ^{19}F NMR (376 MHz, Chloroform-d) δ -58.06; HPLC conditions: DAICEL Chiralpak IC-3 column, *n*-Hexane/*i*-PrOH = 95/5, 254 nm, 0.8 mL/min, t_{major} = 13.071 min, t_{minor} = 16.722 min; HRMS (ESI-MS) Calcd. For $C_{17}H_{16}F_3NO_3$ $[M+H]^+$ 340.1155, found: 340.1152.

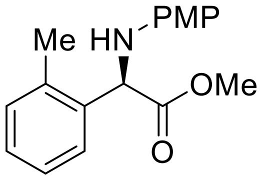

Methyl (*R*)-2-((4-methoxyphenyl)amino)-2-(*o*-tolyl)acetate (2f)

Colorless liquid, 97% yield, 98% ee, $[\alpha]_D^{20}$ = -84.8 (c = 1.21, CH_2Cl_2); ^{1}H NMR (400 MHz, Chloroform-*d*) δ 7.38 (d, *J* = 6.8 Hz, 1H), 7.21-7.13 (m, 3H), 6.71 (d, *J* = 8.8 Hz, 2H), 6.50 (d, *J* = 8.8 Hz, 2H), 5.22 (s, 1H), 4.54 (s, 1H), 3.69 (s, 3H), 3.68 (s, 3H), 2.51 (s, 3H); ^{13}C NMR (101 MHz, Chloroform-*d*) δ 173.0, 152.6, 140.5, 136.7, 136.1, 131.0, 128.2, 126.7, 126.5, 114.9, 114.6, 58.3, 55.7, 52.6, 19.6; HPLC conditions: DAICEL Chiralpak IE column, *n*-Hexane/*i*-PrOH = 95/5, 254 nm, 0.8 mL/min, t_{major} = 20.640 min, t_{minor} = 15.016 min; HRMS (ESI-MS) Calcd. For $C_{17}H_{19}NO_3$ $[M+H]^+$ 286.1438, found: 286.1434.

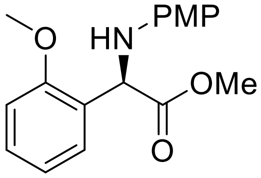

Methyl (*R*)-2-(2-methoxyphenyl)-2-((4-methoxyphenyl)amino)acetate (2g)[3]

Colorless liquid, 99% yield, 97% ee; $[\alpha]_D^{20}$ = -132.4 (c = 0.84, CH_2Cl_2); ^{1}H NMR (400 MHz, Chloroform-*d*) δ 7.33 (d, *J* = 9.2 Hz, 1H), 7.28-7.21 (m, 1H), 6.93-6.89 (m, 2H), 6.71 (d, *J* = 8.8 Hz, 2H), 6.59 (d, *J* = 8.8 Hz, 2H), 5.44 (s, 1H), 4.61 (s, 1H), 3.88 (s, 3H), 3.68 (s, 3H), 3.67 (s,

3H); ^{13}C NMR (101 MHz, Chloroform-*d*) δ 173.3, 157.4, 152.8, 140.9, 129.7, 128.4, 126.7, 121.3, 115.3, 115.0, 111.4, 56.1, 56.0, 55.9, 52.8; HPLC conditions: DAICEL Chiralpak IE column, *n*-Hexane/*i*-PrOH = 95/5, 254 nm, 0.8 mL/min, t_{major} = 32.553 min, t_{minor} = 29.835 min.

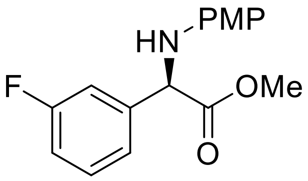

Methyl (*R*)-2-(3-fluorophenyl)-2-((4-methoxyphenyl)amino)acetate (2h)[3]

Colorless liquid, 95% yield, 98% ee, $[\alpha]_D^{20}$ = -80.8 (c = 1.15, CH_2Cl_2); ^{1}H NMR (400 MHz, Chloroform-*d*) δ 7.48-7.30 (m, 2H), 7.28-7.26 (m, 1H), 7.05-7.00 (m, 1H), 6.77 (d, *J* = 8.8 Hz, 2H), 6.56 (d, *J* = 8.8 Hz, 2H), 5.06 (s, 1H), 4.77 (s, 1H), 3.76 (s, 3H), 3.74 (s, 3H); ^{13}C NMR (101 MHz, Chloroform-*d*) δ 172.2, 163.4 (d, *J* = 247.5 Hz), 153.0, 140.8 (d, *J* = 6.7 Hz), 140.2, 130.6 (d, *J* = 8.9 Hz), 123.2, 115.5 (d, *J* = 21.2 Hz), 115.2, 115.1, 114.5 (d, *J* = 22.2 Hz), 61.5, 55.9, 53.0; ^{19}F NMR (376 MHz, Chloroform-*d*) δ -112.64; HPLC conditions: DAICEL Chiralpak IE column, *n*-Hexane/*i*-PrOH = 95/5, 254 nm, 0.8 mL/min, t_{major} = 24.444 min, t_{minor} = 14.223 min.

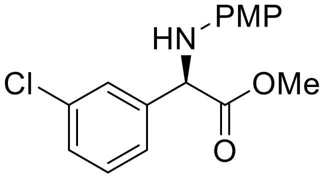

Methyl (*R*)-2-(3-chlorophenyl)-2-((4-methoxyphenyl)amino)acetate (2i)

Colorless liquid, 99% yield, 97% ee, $[\alpha]_D^{20}$ = -83.6 (c = 1.47, CH_2Cl_2); ^{1}H NMR (400 MHz, Chloroform-*d*) δ 7.50 (s, 1H), 7.40-7.35 (m, 1H), 7.29-7.27 (m, 2H), 6.72 (d, *J* = 9.2 Hz, 2H), 6.50 (d, *J* = 9.2 Hz, 2H), 4.97 (s, 1H), 4.72 (s, 1H), 3.73 (s, 3H), 3.70 (s, 3H); ^{13}C NMR (101 MHz, Chloroform-*d*) δ 172.1, 152.9, 140.3, 140.0, 135.0, 130.3, 128.7, 127.7, 125.7, 115.1, 115.0, 61.4, 55.9, 53.2; HPLC conditions: DAICEL Chiralpak IE column, *n*-Hexane/*i*-PrOH = 95/5, 254 nm, 0.8 mL/min, t_{major} = 17.912 min, t_{minor} = 13.519 min; HRMS (ESI-MS) Calcd. For $C_{16}H_{16}ClNO_3$ $[M+H]^+$ 306.0891, found: 306.0891.

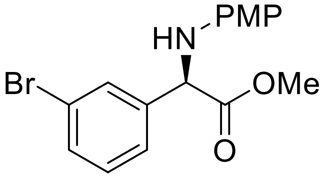

Methyl (*R*)-2-(3-bromophenyl)-2-((4-methoxyphenyl)amino)acetate (2j)

Colorless liquid, 97% yield, 97% ee, $[\alpha]_D^{20}$ = -82.8 (c =1.47, CH_2Cl_2); ^{1}H NMR (400 MHz, Chloroform-*d*) δ 7.65 (t, *J* = 2.0 Hz, 1H), 7.42-7.41 (m, 2H), 7.20 (t, *J* = 8.0 Hz, 1H), 6.71 (d, *J* = 9.2 Hz, 2H), 6.50 (d, *J* = 9.2 Hz, 2H), 4.96 (s, 1H), 4.73 (s, 1H), 3.72 (s, 3H), 3.69 (s, 3H); ^{13}C NMR (101 MHz, Chloroform-*d*) δ 171.9, 152.7, 140.4, 139.8, 131.5, 130.4, 130.4, 126.0, 123.0, 114.9, 114.8, 61.1, 55.7, 53.0; HPLC conditions: DAICEL Chiralpak IE column, *n*-Hexane/*i*-PrOH = 95/5, 254 nm, 0.8 mL/min, t_{major} = 16.794 min, t_{minor} = 13.547 min; HRMS (ESI-MS) Calcd. For $C_{16}H_{16}BrNO_3$ $[M+H]^+$ 350.0386, found: 350.0386.

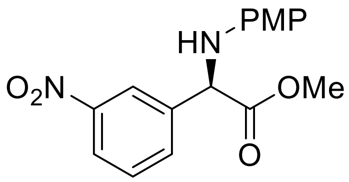

Methyl (*R*)-2-((4-methoxyphenyl)amino)-2-(3-nitrophenyl)acetate (2k)[3]

Colorless liquid, 83% yield, 90% ee, $[\alpha]_D^{20}$ = -63.9 (c = 0.33, CH_2Cl_2); ^{1}H NMR (400 MHz, Chloroform-*d*) δ 8.38 (t, *J* = 2.0 Hz, 1H), 8.15 (d, *J* = 8.4 Hz, 1H), 7.85 (d, *J* = 8.0 Hz, 1H), 7.52 (t, *J* = 8.0 Hz, 1H), 6.71 (d, *J* = 8.8 Hz, 2H), 6.50 (d, *J* = 8.8 Hz, 2H), 5.12 (s, 1H), 4.86 (s, 1H), 3.75 (s, 3H), 3.69 (s, 3H); ^{13}C NMR (101 MHz, Chloroform-*d*) δ 171.4, 153.2, 148.9, 140.7, 139.6, 133.5, 130.0, 123.5, 122.7, 115.2, 115.15, 61.3, 55.9, 53.4; HPLC conditions: DAICEL Chiralpak IE column, *n*-Hexane/*i*-PrOH = 90/10, 254 nm, 0.8 mL/min, t_{major} = 39.927 min, t_{minor} = 26.133 min.

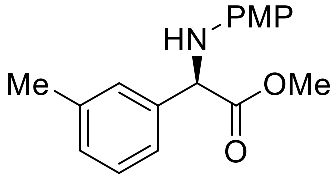

Methyl (*R*)-2-((4-methoxyphenyl)amino)-2-(*m*-tolyl)acetate (2l)

Colorless liquid, 98% yield, 96% ee, $[\alpha]_D^{20}$ = -89.8 (c = 1.30, CH_2Cl_2); ^{1}H NMR (400 MHz, Chloroform-*d*) δ 7.28-7.21 (m, 3H), 7.11 (d, *J* = 6.8 Hz, 1H), 6.72 (d, *J* = 8.8 Hz, 2H), 6.53 (d, *J* = 8.8 Hz, 2H), 4.97 (s, 1H), 4.60 (s, 1H), 3.71 (s, 3H), 3.70 (s, 3H), 2.34 (s, 3H); ^{13}C NMR (101 MHz, Chloroform-*d*) δ 172.7, 152.5, 140.4, 138.6, 137.8, 129.1, 128.8, 127.9, 124.5, 114.9, 114.8, 61.7, 55.7, 52.7, 21.5; HPLC conditions: DAICEL Chiralpak IE column, *n*-Hexane/*i*-PrOH = 95/5, 254 nm, 0.8 mL/min, t_{major} = 18.768 min, t_{minor} = 14.484 min; HRMS (ESI-MS) Calcd. For $C_{17}H_{19}NO_3$ $[M+H]^+$ 286.1438, found: 286.1435.

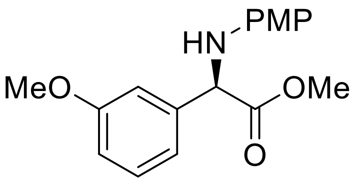

Methyl (*R*)-2-(3-methoxyphenyl)-2-((4-methoxyphenyl)amino)acetate (2m)[3]

Colorless liquid, 97% yield, 96% ee, $[\alpha]_D^{20}$ = -97.5 (c = 1.18, CH_2Cl_2); ^{1}H NMR (400 MHz, Chloroform-*d*) *δ* 7.26-7.22 (m, 1H), 7.07-7.03 (m, 2H), 6.82 (dd, *J* = 8.4 Hz, 1.6 Hz, 1H), 6.70 (d, *J* = 8.8 Hz, 2H), 6.52 (d, *J* = 8.8 Hz, 2H), 4.97 (s, 1H), 4.67 (s, 1H), 3.76 (s, 3H), 3.69 (s, 3H), 3.68 (s, 3H); ^{13}C NMR (101 MHz, Chloroform-*d*) *δ* 172.5, 160.0, 152.6, 140.2, 139.5, 129.9, 119.7, 114.9, 114.8, 113.7, 112.9, 61.7, 55.7, 55.3, 52.8; HPLC conditions: DAICEL Chiralpak IE column, *n*-Hexane/*i*-PrOH = 90/10, 254 nm, 0.8 mL/min, t_{major} = 28.527 min, t_{minor} = 18.349 min.

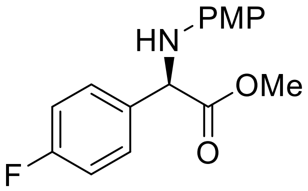

Methyl (*R*)-2-(4-fluorophenyl)-2-((4-methoxyphenyl)amino)acetate (2n)[3]

Colorless liquid, 95% yield, 96% ee, $[\alpha]_D^{20}$ = -72.4 (c = 0.83, CH_2Cl_2); ^{1}H NMR (400 MHz, Chloroform-*d*) *δ* 7.47-7.44 (m, 2H), 7.03 (t, *J* = 8.8 Hz, 2H), 6.71 (d, *J* = 8.8 Hz, 2H), 6.50 (d, *J* = 8.8 Hz, 2H), 4.99 (d, *J* = 3.6 Hz, 1H), 4.69 (s, 1H), 3.71 (s, 3H), 3.69 (s, 3H); ^{13}C NMR (101 MHz, Chloroform-*d*) *δ* 172.3, 162.6 (d, *J* = 247.5 Hz), 152.6, 139.9, 133.6 (d, *J* = 3.0 Hz), 129.0 (d, *J* = 9.1 Hz), 115.8 (d, *J* = 21.2 Hz), 114.9, 114.8, 60.9, 55.7, 52.8; ^{19}F NMR (376 MHz, Chloroform-*d*) *δ* -113.93; HPLC conditions: DAICEL Chiralpak IE column, *n*-Hexane/*i*-PrOH = 95/5, 254 nm, 0.8 mL/min, t_{major} = 17.385 min, t_{minor} = 15.893 min.

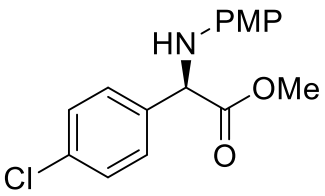

Methyl (*R*)-2-(4-chlorophenyl)-2-((4-methoxyphenyl)amino)acetate (2o)[3]

Colorless liquid, 99% yield, 97% ee, $[\alpha]_D^{20}$ = -100.6 (c = 0.95, CH_2Cl_2); ^{1}H NMR (400 MHz, Chloroform-*d*) *δ* 7.42 (d, *J* = 8.4 Hz, 2H), 7.31 (d, *J* = 8.4 Hz, 2H), 6.71 (d, *J* = 8.8 Hz, 2H), 6.49 (d, *J* = 8.8 Hz, 2H), 4.98 (s, 1H), 4.70 (s, 1H), 3.71 (s, 3H), 3.69 (s, 3H); ^{13}C NMR (101 MHz, Chloroform-*d*) *δ* 172.1, 152.6, 139.8, 136.4, 134.1, 129.0, 128.7, 114.9, 114.8, 61.0, 55.7, 52.9;

HPLC conditions: DAICEL Chiralpak IC-3 column, *n*-Hexane/*i*-PrOH = 95/5, 254 nm, 0.8 mL/min, t_{major} = 17.291 min, t_{minor} = 19.911 min.

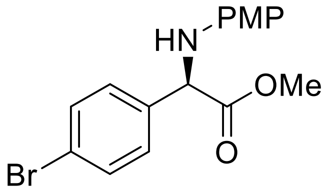

Methyl (*R*)-2-(4-bromophenyl)-2-((4-methoxyphenyl)amino)acetate (2p)[3]

Colorless liquid, 98% yield, 95% ee, $[\alpha]_D^{20}$ = -57.9 (c = 1.68, CH_2Cl_2); ^1H NMR (400 MHz, Chloroform-*d*) δ 7.46 (d, *J* = 8.8 Hz, 2H), 7.36 (d, *J* = 8.8 Hz, 2H), 6.70 (d, *J* = 8.8 Hz, 2H), 6.48 (d, *J* = 8.8 Hz, 2H), 4.96 (s, 1H), 4.72 (s, 1H), 3.70 (s, 3H), 3.68 (s, 3H); ^{13}C NMR (101 MHz, Chloroform-*d*) δ 172.0, 152.7, 139.8, 137.0, 132.0, 129.0, 122.3, 114.9, 114.8, 61.0, 55.7, 52.9; HPLC conditions: DAICEL Chiralpak IC-3 column, *n*-Hexane/*i*-PrOH = 95/5, 254 nm, 0.8 mL/min, t_{major} = 18.308 min, t_{minor} = 20.893 min.

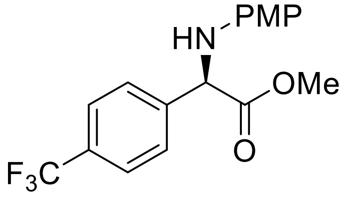

Methyl (*R*)-2-((4-methoxyphenyl)amino)-2-(4-(trifluoromethyl)phenyl)acetate (2q)

Colorless liquid, 96% yield, 97% ee, $[\alpha]_D^{20}$ = -102.7 (c = 0.35, CH_2Cl_2); ^1H NMR (400 MHz, Chloroform-*d*) δ 7.64-7.59 (m, 4H), 6.72 (d, *J* = 8.8 Hz, 2H), 6.49 (d, *J* = 8.8 Hz, 2H), 5.07 (s, 1H), 4.78 (s, 1H), 3.73 (s, 3H), 3.69 (s, 3H); ^{13}C NMR (101 MHz, Chloroform-*d*) δ 171.7, 152.7, 142.0 (d, *J* = 1.0 Hz), 139.7, 130.5 (q, *J* = 33.3 Hz), 127.7, 125.8 (q, *J* = 4.0 Hz), 124.0 (q, *J* = 272.7 Hz),114.9, 114.8, 61.3, 55.6, 53.0; ^{19}F NMR (376 MHz, Chloroform-*d*) δ -62.73; HPLC conditions: DAICEL Chiralpak IC-3 column, *n*-Hexane/*i*-PrOH = 95/5, 254 nm, 0.8 mL/min, t_{major} = 12.291 min, t_{minor} = 14.295 min; HRMS (ESI-MS) Calcd. For $C_{17}H_{16}F_3NO_3[M+Na]^+$ 340.1155, found: 340.1153.

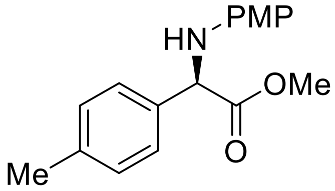

Methyl (*R*)-2-((4-methoxyphenyl)amino)-2-(*p*-tolyl)acetate (2r)[3]

Colorless liquid, 99% yield, 96% ee, $[\alpha]_D^{20}$ = -105.6 (c = 1.10, CH_2Cl_2); ^{1}H NMR (400 MHz, Chloroform-*d*) *δ* 7.35 (d, *J* = 8.0 Hz, 2H), 7.14 (d, *J* = 8.0 Hz, 2H), 6.70 (d, *J* = 8.8 Hz, 2H), 6.52 (d, *J* = 8.8 Hz, 2H), 4.98 (s, 1H), 4.64 (s, 1H), 3.69 (s, 3H), 3.68 (s, 3H), 2.31 (s, 3H); ^{13}C NMR (101 MHz, Chloroform-*d*) *δ* 172.8, 152.5, 140.3, 138.1, 134.8, 129.6, 127.2, 114.9, 114.8, 61.4, 55.7, 52.7, 21.2; HPLC conditions: DAICEL Chiralpak IE column, *n*-Hexane/*i*-PrOH = 95/5, 254 nm, 0.8 mL/min, t_{major} = 24.215 min, t_{minor} = 19.510 min.

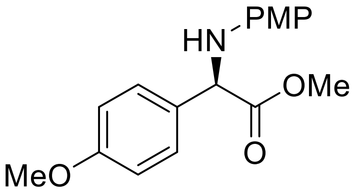

Methyl (*R*)-2-(4-methoxyphenyl)-2-((4-methoxyphenyl)amino)acetate (2s)[3]

Colorless liquid, 95% yield, 94% ee, $[\alpha]_D^{20}$ = -103.2 (c = 1.37, CH_2Cl_2); ^{1}H NMR (400 MHz, Chloroform-*d*) *δ* 7.38 (d, *J* = 8.8 Hz, 2H), 6.86 (d, *J* = 8.8 Hz, 2H), 6.71 (d, *J* = 8.8 Hz, 2H), 6.52 (d, *J* = 8.8 Hz, 2H), 4.96 (s, 1H), 4.62 (s, 1H), 3.75 (s, 3H), 3.69 (s, 3H), 3.68 (s, 3H); ^{13}C NMR (101 MHz, Chloroform-*d*) *δ* 172.8, 159.6, 152.5, 140.3, 129.8, 128.5, 114.9, 114.8, 114.3, 61.0, 55.7, 55.3, 52.7; HPLC conditions: DAICEL Chiralpak IE column, *n*-Hexane/*i*-PrOH = 90/10, 254 nm, 0.8 mL/min, t_{major} = 32.183 min, t_{minor} = 21.198 min.

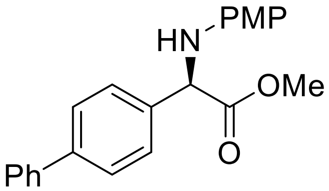

Methyl (*R*)-2-([1,1'-biphenyl]-4-yl)-2-((4-methoxyphenyl)amino)acetate (2t)

Colorless liquid, 98% yield, 95% ee, $[\alpha]_D^{20}$ = -128.8 (c = 0.84, CH_2Cl_2); ^{1}H NMR (400 MHz, Chloroform-*d*) *δ* 7.66-7.60 (m, 6H), 7.50-7.46 (m, 2H), 7.42-7.37 (m, 1H), 6.80 (d, *J* = 8.8 Hz, 2H), 6.63 (d, *J* = 8.8 Hz, 2H), 5.13 (s, 1H), 3.79 (s, 3H), 3.75 (s, 3H); ^{13}C NMR (101 MHz, Chloroform-*d*) *δ* 172.6, 152.6, 141.2, 140.6, 140.2, 136.8, 128.8, 127.8, 127.6, 127.5, 127.1, 114.9, 114.8, 61.4, 55.7, 52.8; HPLC conditions: DAICEL Chiralpak IE column, *n*-Hexane/*i*-PrOH = 90/10, 254 nm, 0.8 mL/min, t_{major} = 35.929 min, t_{minor} = 19.924 min; HRMS (ESI-MS) Calcd. For $C_{22}H_{21}NO_3[M+H]^+$ 348.1594, found: 348.1591.

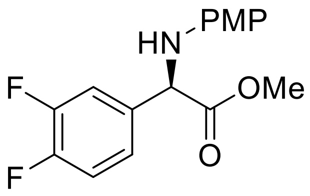

Methyl (*R*)-2-(3,4-difluorophenyl)-2-((4-methoxyphenyl)amino)acetate (2u)

Colorless liquid, 86% yield, 95% ee, $[\alpha]_D^{20}$ = -64.9 (c = 0.82, CH_2Cl_2); ^1H NMR (400 MHz, Chloroform-*d*) δ 7.36-7.30 (m, 1H), 7.26-7.22 (m, 1H), 7.16-7.09 (m, 1H), 6.72 (d, *J* = 9.2 Hz, 2H), 6.48 (d, *J* = 9.2 Hz, 2H), 4.96 (s, 1H), 4.74 (s, 1H), 3.73 (s, 3H), 3.70 (s, 3H); ^{13}C NMR (101 MHz, Chloroform-*d*) δ 171.9, 153.0, 150.8 (dd, *J* = 250.5 Hz, 13.1 Hz), 150.5 (dd, *J* = 250.5 Hz, 12.1 Hz), 139.8, 135.2 (t, *J* = 3.0 Hz), 123.5 (t, *J* = 5.1 Hz), 117.8 (d, *J* = 17.2 Hz), 116.5 (d, *J* = 17.2 Hz), 115.1, 115.0, 60.9, 55.9, 53.2; ^{19}F NMR (376 MHz, Chloroform-*d*) δ -137.04, -138.67; HPLC conditions: DAICEL Chiralpak IE column, *n*-Hexane/*i*-PrOH = 98/2, 254 nm, 0.8 mL/min, t_{major} = 39.634 min, t_{minor} = 21.782 min; HRMS (ESI-MS) Calcd. For $C_{16}H_{15}F_2NO_3$ $[M+Na]^+$ 308.1093, found: 308.1092.

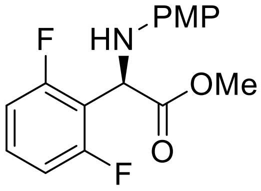

Methyl (*R*)-2-(2,6-difluorophenyl)-2-((4-methoxyphenyl)amino)acetate (2v)

Colorless liquid, 98% yield, 96% ee, $[\alpha]_D^{20}$ = -201.9 (c = 1.04, CH_2Cl_2); ^1H NMR (400 MHz, Chloroform-*d*) δ 7.26-7.17 (m, 1H), 6.87 (t, *J* = 8.0 Hz, 2H), 6.73 (d, *J* = 8.8 Hz, 2H), 6.67 (d, *J* = 8.8 Hz, 2H), 5.50 (s, 1H), 4.65 (s, 1H), 3.73 (s, 3H), 3.69 (s, 3H); ^{13}C NMR (101 MHz, Chloroform-*d*) δ 171.1, 161.2 (dd, *J* = 249.5 Hz, 8.1 Hz), 153.0, 139.9, 129.9 (t, *J* = 10.1 Hz), 115.4, 115.2 (t, *J* = 15.2 Hz), 114.9, 111.8 (dd, *J* = 20.2 Hz, 7.1 Hz), 55.6, 53.0, 52.0 (t, *J* = 2.0 Hz); ^{19}F NMR (376 MHz, Chloroform-*d*) δ -115.44; HPLC conditions: DAICEL Chiralpak IE column, *n*-Hexane/*i*-PrOH = 95/5, 254 nm, 0.8 mL/min, t_{major} = 25.203 min, t_{minor} = 22.758 min; HRMS (ESI-MS) Calcd. For $C_{16}H_{15}$ F_2NO_3 $[M+H]^+$ 308.1093, found: 308.1091.

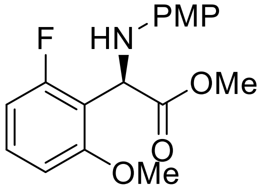

Methyl (*R*)-2-(2-fluoro-6-methoxyphenyl)-2-((4-methoxyphenyl)amino)acetate (2w)

Colorless liquid, 99% yield, 96% ee, $[\alpha]_D^{20}$ = -217.2 (c = 1.13, CH_2Cl_2); ^{1}H NMR (400 MHz, Chloroform-*d*) δ 7.21-7.15 (m, 1H), 6.74-6.64 (m, 6H), 5.56 (s, 1H), 4.70 (s, 1H), 3.85 (s, 3H), 3.69 (s, 6H); ^{13}C NMR (101 MHz, Chloroform-*d*) δ 172.3 (d, *J* = 1.0 Hz), 161.4 (d, *J* = 246.4 Hz), 158.4 (d, *J* = 8.1 Hz), 152.8, 140.7, 129.7 (d, *J* =11.1 Hz), 115.5, 115.1 (d, *J* = 16.2 Hz), 114.7, 108.4 (d, *J* = 23.2 Hz), 106.9 (d, *J* = 3.0 Hz), 56.2, 55.6, 52.6, 52.2 (d, *J* = 3.0 Hz); ^{19}F NMR (376 MHz, Chloroform-*d*) δ -116.99; HPLC conditions: DAICEL Chiralpak IE column, *n*-Hexane/*i*-PrOH = 90/10, 254 nm, 0.8 mL/min, t_{major} = 26.262 min, t_{minor} = 28.215 min; HRMS (ESI-MS) Calcd. For $C_{17}H_{18}$ F NO_4 $[M+Na]^+$ 320.1293, found: 320.1285.

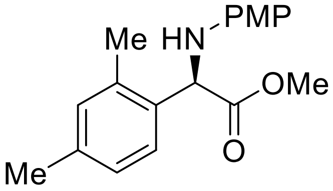

Methyl (*R*)-2-(2,4-dimethylphenyl)-2-((4-methoxyphenyl)amino)acetate (2x)

Colorless liquid, 97% yield, 98% ee, $[\alpha]_D^{20}$ = -102.0 (c = 1.24, CH_2Cl_2); ^{1}H NMR (400 MHz, Chloroform-*d*) δ 7.25 (d, *J* = 8.0 Hz, 1H), 7.01-6.97 (m, 2H), 6.71 (d, *J* = 9.2 Hz, 2H), 6.50 (d, *J* = 9.2 Hz, 2H), 5.18 (s, 1H), 4.49 (s, 1H), 3.68 (s, 3H), 3.678 (s, 3H), 2.47 (s, 3H), 2.28 (s, 3H); ^{13}C NMR (101 MHz, Chloroform-*d*) δ 173.2, 152.6, 140.6, 137.9, 136.4, 133.2, 131.8, 127.3, 126.5, 114.9, 114.6, 58.1, 55.7, 52.6, 21.1, 19.4; HPLC conditions: DAICEL Chiralpak IC-3 column, *n*-Hexane/*i*-PrOH = 90/10, 254 nm, 0.8 mL/min, t_{major} = 14.820 min, t_{minor} = 17.046 min; HRMS (ESI-MS) Calcd. For $C_{18}H_{21}NO_3$ $[M+Na]^+$ 300.1594, found: 300.1592.

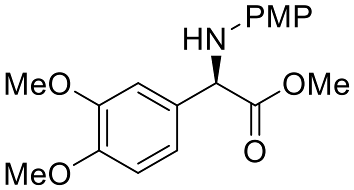

Methyl (*R*)-2-(3,4-dimethoxyphenyl)-2-((4-methoxyphenyl)amino)acetate (2y)

Colorless liquid, 99% yield, 96% ee, $[\alpha]_D^{25}$ = -96.3 (c =1.29, CH_2Cl_2); ^{1}H NMR (400 MHz, Chloroform-*d*) δ 7.04-7.00 (m, 2H), 6.83 (d, *J* = 8.0 Hz, 1H), 6.73 (d, *J* = 8.8 Hz, 2H), 6.54 (d, *J* = 8.8 Hz, 2H), 4.94 (s, 1H), 4.62 (s, 1H), 3.86 (s, 3H), 3.85 (s, 3H), 3.71 (s, 3H), 3.69 (s, 3H); ^{13}C NMR (101 MHz, Chloroform-*d*) δ 172.8, 152.6, 149.3, 149.0, 140.3, 130.2, 119.7, 114.84, 114.82, 111.3, 110.1, 61.4, 55.9, 55.89, 55.7, 52.7; HPLC conditions: DAICEL Chiralpak IE column, *n*-Hexane/*i*-PrOH = 80/20, 254 nm, 0.8 mL/min, t_{major} = 28.426 min, t_{minor} = 24.648 min; HRMS (ESI-MS) Calcd. For $C_{18}H_{21}NO_5$ $[M+H]^+$ 332.1492, found: 332.1489.

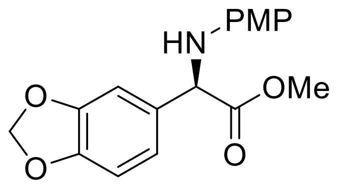

Methyl (*R*)-2-(benzo[*d*][1,3]dioxol-5-yl)-2-((4-methoxyphenyl)amino)acetate (2z)

Colorless liquid, 99% yield, 94% ee, $[\alpha]_D^{20}$ = -108.8 (c = 1.31, CH_2Cl_2); ^{1}H NMR (400 MHz, Chloroform-*d*) δ 6.96-6.94 (m, 2H), 6.76 (d, *J* = 8.4 Hz, 1H), 6.71 (d, *J* = 8.8 Hz, 2H), 6.51 (d, *J* = 8.8 Hz, 2H), 5.92-5.91 (m, 2H), 4.91 (s, 1H), 4.65 (s, 1H), 3.71 (s, 3H), 3.69 (s, 3H); ^{13}C NMR (101 MHz, Chloroform-*d*) δ 172.5, 152.5, 148.1, 147.6, 140.1, 131.7, 120.9, 114.9, 114.8, 108.5, 107.6, 101.2, 61.2, 55.7, 52.8; HPLC conditions: DAICEL Chiralpak IE column, *n*-Hexane/*i*-PrOH = 80/20, 254 nm, 0.8 mL/min, t_{major} = 28.090 min, t_{minor} = 16.268 min; HRMS (ESI-MS) Calcd. For $C_{17}H_{17}NO_5$ $[M+H]^+$ 316.1179, found: 316.1177.

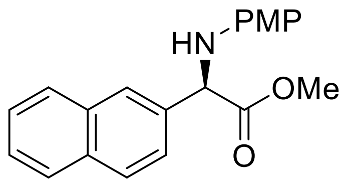

Methyl (*R*)-2-((4-methoxyphenyl)amino)-2-(naphthalen-2-yl)acetate (2aa)[3]

Colorless liquid, 98% yield, 92% ee, $[\alpha]_D^{20}$ = -104.9 (c = 0.70, CH_2Cl_2); ^{1}H NMR (400 MHz, Chloroform-*d*) δ 7.95 (s, 1H), 7.83-7.79 (m, 3H), 7.59 (d, *J* = 8.4 Hz, 1H), 7.48-7.44 (m, 2H), 6.70 (d, *J* = 8.0 Hz, 2H), 6.56 (d, *J* = 8.0 Hz, 2H), 5.17 (s, 1H), 4.80 (s, 1H), 3.70 (s, 3H), 3.66 (s, 3H); ^{13}C NMR (101 MHz, Chloroform-*d*) δ 172.5, 152.6, 140.2, 135.4, 133.4, 133.3, 128.8, 128.1, 127.7, 126.5, 126.4, 126.3, 125.0, 114.9, 114.86, 61.8, 55.7, 52.8; HPLC conditions: DAICEL Chiralpak IE column, *n*-Hexane/*i*-PrOH = 90/10, 254 nm, 0.8 mL/min, t_{major} = 24.698 min, t_{minor} = 15.563 min.

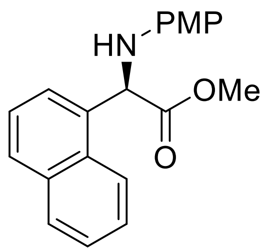

Methyl (*R*)-2-((4-methoxyphenyl)amino)-2-(naphthalen-1-yl)acetate (2ab)[3]

Colorless liquid, 81% yield, 95% ee, $[\alpha]_D^{20}$ = -52.5 (c = 1.05, CH_2Cl_2); ^{1}H NMR (400 MHz, Chloroform-*d*) δ 8.28 (d, *J* = 9.2 Hz, 1H), 7.88 (d, *J* = 8.0 Hz, 1H), 7.81 (d, *J* = 9.2 Hz, 1H), 7.63-7.49 (m, 3H), 7.44-7.40 (m, 1H), 6.69 (d, *J* = 8.8 Hz, 2H), 6.53 (d, *J* = 8.8 Hz, 2H), 5.76 (s, 1H),

4.61 (s, 1H), 3.67 (s, 3H), 3.66 (s, 3H); ^{13}C NMR (101 MHz, Chloroform-*d*) *δ* 173.0, 152.6, 140.5, 134.2, 133.6, 131.4, 129.1, 129.0, 126.7, 126.0, 125.6, 125.1, 123.4, 114.9, 114.6, 58.4, 55.7, 52.8; HPLC conditions: DAICEL Chiralpak IE column, *n*-Hexane/*i*-PrOH = 95/5, 254 nm, 0.8 mL/min, t_{major} = 25.272 min, t_{minor} = 19.838 min.

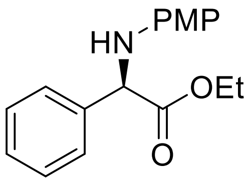

Ethyl (*R*)-2-((4-methoxyphenyl)amino)-2-phenylacetate (2ac)[3]

Colorless liquid, 99% yield, 96% ee, $[\alpha]_D^{20}$ = -53.6 (c = 1.38, CH_2Cl_2); ^{1}H NMR (400 MHz, Chloroform-*d*) *δ* 7.48 (d, *J* = 6.8 Hz, 2H), 7.34-7.25 (m, 3H), 6.70 (d, *J* = 8.8 Hz, 2H), 6.52 (d, *J* = 8.8 Hz, 2H), 5.00 (s, 1H), 4.67 (s, 1H), 4.23-4.06 (m, 2H), 3.66 (s, 3H), 1.17 (t, *J* = 7.2 Hz, 3H); ^{13}C NMR (101 MHz, Chloroform-*d*) *δ* 172.1, 152.5, 140.3, 138.0, 128.8, 128.2, 127.3, 114.9, 114.8, 61.8, 61.7, 55.7, 14.1; HPLC conditions: DAICEL Chiralpak IE column, *n*-Hexane/*i*-PrOH = 95/5, 254 nm, 0.8 mL/min, t_{major} = 20.575 min, t_{minor} = 17.438 min.

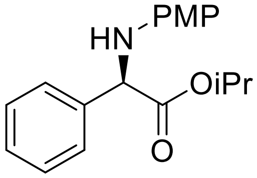

Isopropyl (*R*)-2-((4-methoxyphenyl)amino)-2-phenylacetate (2ad)[4]

Colorless liquid, 99% yield, 95% ee, $[\alpha]_D^{20}$ = -211.5 (c = 1.42, CH_2Cl_2); ^{1}H NMR (400 MHz, Chloroform-*d*) *δ* 7.47 (d, *J* = 7.2 Hz, 2H), 7.34-7.24 (m, 3H), 6.70 (d, *J* = 8.8 Hz, 2H), 6.52 (d, *J* = 8.8 Hz, 2H), 5.06-4.98 (m, 1H), 4.97 (d, *J* = 3.9 Hz, 1H), 4.67 (s, 1H), 3.67 (s, 3H), 1.24 (d, *J* = 6.4 Hz, 3H), 1.06 (d, *J* = 6.0 Hz, 3H); ^{13}C NMR (101 MHz, Chloroform-*d*) *δ* 171.6, 152.5, 140.4, 138.0, 128.7, 128.1, 127.2, 114.9, 114.8, 69.4, 61.8, 55.7, 21.8, 21.4; HPLC conditions: DAICEL Chiralpak AS-H column, *n*-Hexane/*i*-PrOH = 95/5, 254 nm, 0.8 mL/min, t_{major} = 14.978 min, t_{minor} = 12.037 min.

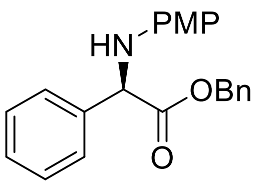

Benzyl (*R*)-2-((4-methoxyphenyl)amino)-2-phenylacetate (2ae)[4]

Colorless liquid, 91% yield, 92% ee, $[\alpha]_D^{20}$ = -45.1 (c = 1.22, CH_2Cl_2); ^1H NMR (400 MHz, Chloroform-*d*) δ 7.47 (d, *J* = 6.0 Hz, 2H), 7.34-7.26 (m, 6H), 7.15-7.13 (m, 2H), 6.69 (d, *J* = 8.8 Hz, 2H), 6.51 (d, *J* = 8.8 Hz, 2H), 5.18-5.06 (m, 3H), 4.67 (s, 1H), 3.66 (s, 3H); ^{13}C NMR (101 MHz, Chloroform-*d*) δ 172.0, 152.6, 140.2, 137.7, 135.4, 128.9, 128.6, 128.4, 128.3, 127.9, 127.4, 114.9, 114.87, 67.3, 61.8, 55.7; HPLC conditions: DAICEL Chiralpak IC-3 column, *n*-Hexane/*i*-PrOH = 90/10, 254 nm, 0.8 mL/min, t_{major} = 14.094 min, t_{minor} = 18.201 min.

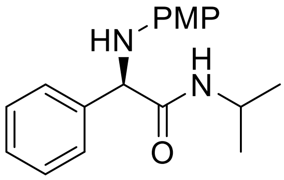

(*R*)-*N*-isopropyl-2-((4-methoxyphenyl)amino)-2-phenylacetamide (2af)

Colorless liquid, 81% yield, 95% ee, $[\alpha]_D^{20}$ = -133.0 (c = 1.17, CH_2Cl_2); ^1H NMR (400 MHz, Chloroform-*d*) δ 7.42-7.30 (m, 5H), 6.77 (d, *J* = 8.8 Hz, 2H), 6.72 (d, *J* = 8.8 Hz, 1H), 6.58 (d, *J* = 8.8 Hz, 2H), 4.61 (s, 1H), 4.21 (s, 1H), 4.14-4.06 (m, 1H), 3.73 (s, 3H), 1.14 (d, *J* = 6.8 Hz, 3H), 1.06 (d, *J* = 6.8 Hz, 3H); ^{13}C NMR (101 MHz, Chloroform-*d*) δ 170.4, 153.2, 140.9, 139.1, 129.1, 128.5, 127.4, 115.0, 114.8, 65.2, 55.7, 41.3, 22.7, 22.5; HPLC conditions: DAICEL Chiralpak OJ-H column, *n*-Hexane/*i*-PrOH = 90/10, 254 nm, 0.8 mL/min, t_{major} = 28.647 min, t_{minor} = 35.398 min; HRMS (ESI-MS) Calcd. For $C_{18}H_{22}N_2O_2$ $[M+H]^+$ 299.1754, found: 299.1752.

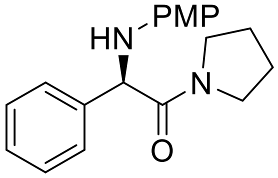

(*R*)-2-((4-methoxyphenyl)amino)-2-phenyl-1-(pyrrolidin-1-yl)ethan-1-one (2ag)

Colorless liquid, 85% yield, 91% ee, $[\alpha]_D^{20}$ = -112.3 (c = 0.77, CH_2Cl_2); ^1H NMR (400 MHz, Chloroform-*d*) δ 7.45 (d, *J* = 8.0 Hz, 2H), 7.31 (t, *J* = 7.2 Hz, 2H), 7.26-7.23 (m, 1H), 6.69 (d, *J* = 8.8 Hz, 2H), 6.58 (d, *J* = 8.8 Hz, 2H), 5.00 (s, 1H), 3.67 (s, 3H), 3.63-3.51 (m, 2H), 3.43-3.36 (m, 1H), 3.28-3.23 (m, 1H), 1.93-1.67 (m, 4H); ^{13}C NMR (101 MHz, Chloroform-*d*) δ 169.4, 152.2, 140.8, 138.4, 128.8, 128.0, 128.0, 115.2, 114.8, 60.9, 55.7, 46.4, 46.3, 26.0, 23.9; HPLC conditions: DAICEL Chiralpak OJ-H column, *n*-Hexane/*i*-PrOH = 80/20, 254 nm, 0.8 mL/min, t_{major} = 27.700 min, t_{minor} = 36.316 min; HRMS (ESI-MS) Calcd. For $C_{19}H_{22}N_2O_2$ $[M+H]^+$ 311.1754, found: 311.1756.

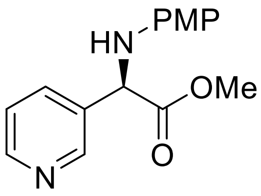

Methyl (*R*)-2-((4-methoxyphenyl)amino)-2-(pyridin-3-yl)acetate (2ah)

White solid, 90% yield, 73% ee, $[\alpha]_D^{25}$ = -63.2 (c = 0.41, CH_2Cl_2); melting point: 90.1-93.4 ^oC; ^1H NMR (400 MHz, Chloroform-*d*) δ 8.79 (d, *J* = 2.4 Hz, 1H), 8.57 (dd, *J* = 4.8, 2.0 Hz, 1H), 7.81 (dt, *J* = 8.0, 2.0 Hz, 1H), 7.32-7.26 (m, 1H), 6.74 (d, *J* = 8.8 Hz, 2H), 6.53 (d, *J* = 8.8 Hz, 2H), 5.08 (s, 1H), 4.73 (s, 1H), 3.76 (s, 3H), 3.72 (s, 3H); ^{13}C NMR (101 MHz, Chloroform-*d*) δ 171.7, 152.8, 149.6, 149.4, 139.6, 134.6, 133.7, 123.8, 114.9, 59.5, 55.7, 53.0; HPLC conditions: DAICEL Chiralpak OD-H column, *n*-Hexane/*i*-PrOH = 80/20, 254 nm, 0.8 mL/min, t_{major} = 12.962 min, t_{minor} = 18.073 min; HRMS (ESI-MS) Calcd. For $C_{15}H_{16}N_2O_3$ $[M+H]^+$ 273.1234, found: 273.1238.

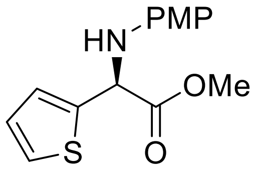

Methyl (*S*)-2-((4-methoxyphenyl)amino)-2-(thiophen-2-yl)acetate (2ai)

Colorless liquid, 23% yield, 39% ee, $[\alpha]_D^{25}$ = 21.9 (c = 0.13, CH_2Cl_2); ^1H NMR (400 MHz, Chloroform-*d*) δ 7.31-7.27 (m, 1H), 7.20-7.15 (m, 1H), 7.04-7.02 (m, 1H), 6.87-6.73 (m, 2H), 6.74-6.55 (m, 2H), 5.36 (s, 1H), 4.71 (s, 1H), 3.81 (s, 3H), 3.76 (s, 3H); ^{13}C NMR (101 MHz, Chloroform-*d*) δ 171.7, 153.0, 141.5, 140.0, 127.1, 125.7, 125.6, 115.3, 114.9, 57.8, 55.7, 53.0; HPLC conditions: DAICEL Chiralpak IC-3 column, *n*-Hexane/*i*-PrOH = 95/5, 254 nm, 0.8 mL/min, t_{major} = 20.352 min, t_{minor} = 18.426 min; HRMS (ESI-MS) Calcd. For $C_{14}H_{15}NO_3S$ $[M+H]^+$ 278.0845, found: 278.0851.

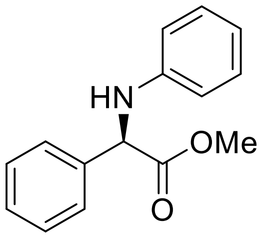

Methyl (*R*)-2-phenyl-2-(phenylamino)acetate (2aj)[6]

Colorless liquid, 93% yield, 95% ee, $[\alpha]_D^{25}$ = -85.9 (c = 0.46, CH_2Cl_2); ^1H NMR (400 MHz, Chloroform-*d*) δ 7.51-7.49 (m, 2H), 7.38-7.31 (m, 3H), 7.14-7.11 (m, 2H), 6.71 (t, *J* = 7.6 Hz,

1H), 6.57 (d, J = 7.6 Hz, 2H), 5.09 (s, 1H), 4.92 (s, 1H), 3.77 (s, 3H); ^{13}C NMR (101 MHz, Chloroform-*d*) δ 172.6, 146.2, 137.8, 129.5, 129.1, 128.5, 127.5, 118.4, 113.6, 61.0, 53.0; HPLC conditions: DAICEL Chiralpak IE column, *n*-Hexane/*i*-PrOH = 98/2, 254 nm, 0.8 mL/min, t_{major} = 9.209 min, t_{minor} = 8.735 min.

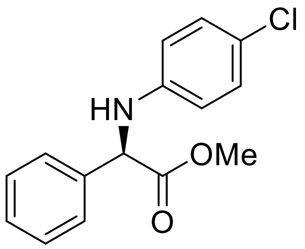

Methyl (*R*)-2-((4-chlorophenyl)amino)-2-phenylacetate (2ak)

White solid, 99% yield, 96% ee, $[\alpha]_D^{25}$ = -100.6 (c = 0.45, CH_2Cl_2); melting point: 96.9-98.6 ^{o}C; ^{1}H NMR (400 MHz, Chloroform-*d*) δ 7.50 (d, J = 6.8 Hz, 2H), 7.43-7.32 (m, 3H), 7.09 (d, J = 8.8 Hz, 2H), 6.50 (d, J = 8.8 Hz, 2H), 5.06 (s, 1H), 3.76 (s, 3H); ^{13}C NMR (101 MHz, Chloroform-*d*) δ172.0, 144.4, 137.1, 129.1, 129.0, 128.5, 127.2, 122.8, 114.5, 60.7, 52.9; HPLC conditions: DAICEL Chiralpak OD-H column, *n*-Hexane/*i*-PrOH = 95/5, 254 nm, 0.8 mL/min, t_{major} = 9.299 min, t_{minor} = 10.523 min; HRMS (ESI-MS) Calcd. For $C_{15}H_{14}ClNO_2$ $[M+H]^+$ 276.0786, found: 276.0800.

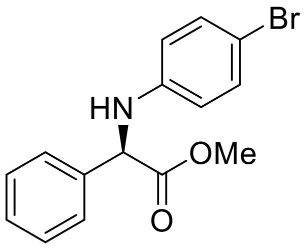

Methyl (*R*)-2-((4-bromophenyl)amino)-2-phenylacetate (2al)

White solid, 99% yield, 94% ee, $[\alpha]_D^{25}$ = -93.9 (c = 0.29, CH_2Cl_2); melting point: 108.1-109.6 ^{o}C; ^{1}H NMR (400 MHz, Chloroform-*d*) δ 7.49 (d, J = 6.8 Hz, 2H), 7.42-7.31 (m, 3H), 7.22 (d, J = 8.8 Hz, 2H), 6.45 (d, J = 8.8 Hz, 2H), 5.05 (s, 1H), 3.76 (s, 3H); ^{13}C NMR (101 MHz, Chloroform-*d*) δ 172.0, 144.8, 137.1, 132.0, 129.0, 128.5, 127.2, 115.0, 109.9, 60.6, 52.9; HPLC conditions: DAICEL Chiralpak OD-H column, *n*-Hexane/*i*-PrOH = 95/5, 254 nm, 0.8 mL/min, t_{major} = 9.874 min, t_{minor} = 11.602 min; HRMS (ESI-MS) Calcd. For $C_{15}H_{14}BrNO_2$ $[M+H]^+$ 320.0281, found: 320.0294.

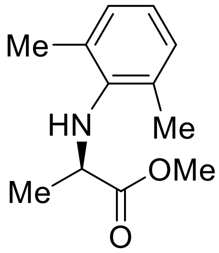

Methyl (2,6-dimethylphenyl)-D-alaninate (2am)[6]

Colorless liquid, 94% yield, 98% ee, $[\alpha]_D^{25}$ = 18.8 (c = 0.38, CH_2Cl_2); ^1H NMR (400 MHz, Chloroform-*d*) δ 6.96 (d, *J* = 7.6 Hz, 2H), 6.79 (t, *J* = 7.6 Hz, 1H), 3.98 (q, *J* = 6.8 Hz, 1H), 3.66 (s, 3H), 2.29 (s, 6H), 1.37 (d, *J* = 6.8 Hz, 3H); ^{13}C NMR (101 MHz, Chloroform-*d*) δ 176.0, 144.0, 129.0, 128.9, 121.9, 55.1, 52.0, 19.7, 18.7; HPLC conditions: DAICEL Chiralpak OD-H column, *n*-Hexane/*i*-PrOH = 99/1, 254 nm, 0.8 mL/min, t_{major} = 7.695 min, t_{minor} = 8.402 min.

Supplementary Note 2

Study of scale up

Supplementary Table 1. The influence of the additives.[a]

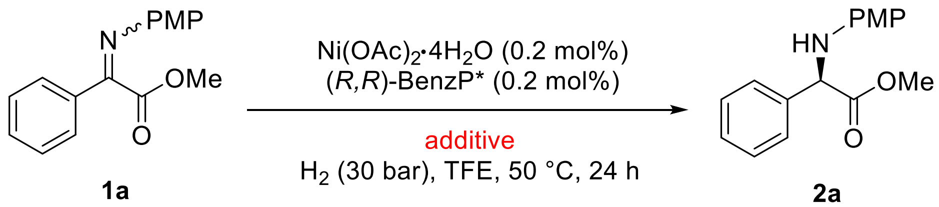

Entry	Additive (0.1 equiv.)	Conv [%][b]	ee [%][c]
1	/	18	95
2	*n*-Bu_4NCl	10	-
3	*n*-Bu_4NBr	13	-
4	*n*-Bu_4NI	9	-
5	*n*-Bu_4NOAc	60	95
6	NaOAc	34	95
7	KOAc	27	95
8	$Zn(OAc)_2$	0	-
9	$Cu(OAc)_2$	0	-
10[d]	$Ni(OAc)_2·4H_2O$	81	95
11[e]	$Ni(OAc)_2·4H_2O$	90	95
12[f]	$Ni(OAc)_2·4H_2O$	93	95

[a] Reaction conditions unless otherwise noted: **1a** (1 mmol), $Ni(OAc)_2·4H_2O$ (0.002 mmol), (*R,R*)-BenzP* (0.002 mmol), additive (0.1 mmol), H_2 (30 bar), TFE (2 mL), 50 ^{o}C, 24 h; [b] The conversions were calculated from ^{1}H NMR spectra; [c] The ee values were determined by HPLC using chiral stationary phase; [d] 0.004 equiv. of $Ni(OAc)_2·4H_2O$ (0.004 mmol); [e] 0.01 equiv. of $Ni(OAc)_2·4H_2O$ (0.01 mmol); [f] 0.02 equiv. of $Ni(OAc)_2·4H_2O$ (0.02 mmol).

Various additives with different anions (entries 2-5, Supplementary Table 1) and cations (entries 6-9) were also examined in the study of the S/C. The OAc^- anions gave the best results compared to other anions (Cl, Br, I, entries 2-5), and the excess $Ni(OAc)_2·4H_2O$ was the best additive of the five acetate salts (Na, K, Zn, Cu, entries 6-10). A small excess of $Ni(OAc)_2·4H_2O$ (0.004 equiv.) can greatly improve the reaction activity (entries 1, 10-12), and the hydrogenation results were similar when 0.01 and 0.02 equiv of $Ni(OAc)_2·4H_2O$ were used.

Supplementary Table 2. The influence of excess $Ni(OAc)_2·4H_2O$ for high catalytic efficiency.[a]

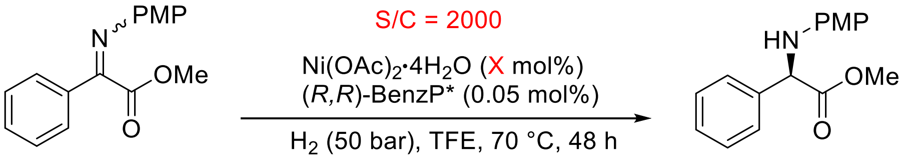

1a **2a**

Entry	X	Conv [%][b]	ee [%][c]
1	0.05 (M/L = 1:1)	6	92
2	0.10 (M/L = 2:1)	32	92
3	0.25 (M/L = 5:1)	69	93
4	0.50(M/L = 10:1)	88	93
5	1.00 (M/L = 20:1)	94	93

[a] Conditions: **1a** (2.154 g, 8 mmol), $Ni(OAc)_2·4H_2O$ (M), (*R*,*R*)-BenzP* (L, 1.13 mg, 0.004 mmol), TFE (9 mL), H_2 (50 bar), 70 °C, 48 h; [b] The conversions were calculated from ^1H NMR spectra; [c] The ee values were determined by HPLC using a chiral column.

As listed in Supplementary Table 2, when the hydrogenation was carried out at M/L ratio of 1:1, only a trace amount of product (6% conv) was detected (entry 1). To our delight, maintaining the quantity of (*R*,*R*)-BenzP* and adding more $Ni(OAc)_2·4H_2O$ could obviously increase the reaction conversion (entries 2-5). When the M/L ratio was 20:1, good results were obtained (entry 5: 94% conv 93% ee).

Supplementary Note 3

Synthesis of 2a on a gram scale.

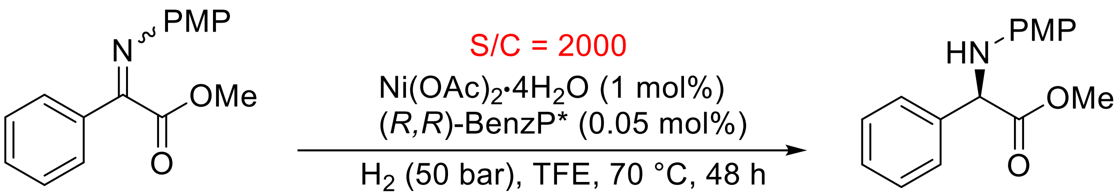

1a
2.154 g, 8 mmol

2a
94% conv, 92% yield, 93% ee

Procedure E: To a hydrogenation tube Ni(OAc)$_2$·4H$_2$O (19.91 mg, 0.08 mmol), (*R,R*)-BenzP* (1.13 mg, 0.004 mmol) and the substrate (2.154 g, 8 mmol) were added, then the mixture was transferred to a nitrogen-filled glovebox. Degassed and anhydrous trifluoroethanol (TFE, 9 mL) was then added. The reaction was carried out under H_2 (50 bar) at 70 ℃ for 48 h. After carefully releasing the hydrogen gas, the pure product **2a** was obtained by column chromatography (PE/EtOAc = 30:1). White solid, 1.996 g, 92% yield, 93% ee. HPLC conditions: DAICEL Chiralpak IE column, *n*-Hexane/*i*-PrOH = 95/5, 254 nm, 0.8 mL/min, t_{major} = 23.299 min, t_{minor} = 18.670 min.

Synthesis of compound 3.

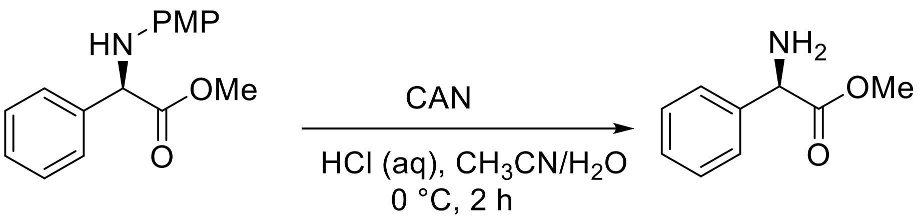

2a
93% ee

3
81% yield, 92 % ee

Procedure F[3]: Methyl (*R*)-2-((4-methoxyphenyl)amino)-2-phenylacetate **2a** (200 mg, 0.737 mmol) was dissolved in acetonitrile/water (6 mL/3 mL), and hydrochloric acid (1 M, 1 mL) was added. Then ceric ammonium nitrate aqueous solution (1.5 g dissolved in 2 mL water, 2.736 mmol) was added in four portions to the reaction mixture in an ice bath. After stirring for 2 h, the resulting dark brown solution was diluted with EtOAc (10 mL) and extracted with water (3 × 5 mL). The combined aqueous layer was treated with saturated $NaHSO_3$ solution (5 mL) and solid $NaHCO_3$ added until pH = 8. EtOAc (3 × 8 mL) was then used to extract the aqueous layer. The

collected organic layer was dried over Na_2SO_4, and concentrated in vacuo. The crude product was purified by silica-gel column chromatography (PE/EtOAc = 3:1, 0.5% Et_3N addition). The obtained product **3** was a yellow liquid: 98 mg, 81% yield, 92% ee; $[\alpha]_D^{20}$ = -146.9 (c = 1.14, CH_2Cl_2); ^{1}H NMR (400 MHz, Chloroform-*d*) *δ* 7.46-7.27 (m, 5H), 4.60 (s, 1H), 3.68 (s, 3H), 2.00 (s, 2H); ^{13}C NMR (101 MHz, Chloroform-*d*) *δ* 174.7, 140.5, 129.0, 128.3, 127.0, 59.0, 52.6; HPLC conditions: DAICEL Chiralpak AS-H column, *n*-Hexane/*i*-PrOH = 90/10, 210 nm, 0.8 mL/min, t_{major} = 16.186 min, t_{minor} = 13.545 min.

Synthesis of compound 5.

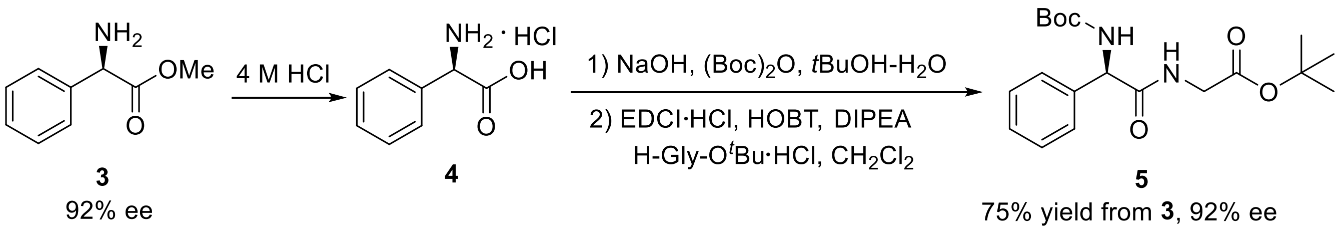

3
92% ee

4

5
75% yield from **3**, 92% ee

Procedure G[7-10]: A mixture of methyl (*R*)-2-amino-2-phenylacetate (**3**, 90 mg, 0.545 mmol) and HCl aq (4 M, 3 mL) was heated to 50 ℃ and reacted overnight. After removing solvent in vacuo, a white solid (**4**) was obtained and used in the next step without further purification.

To a solution of **4** in water (2 mL) and *tert*-butanol (1 mL), NaOH (44.7 mg dissolved in 2 mL water, 1.117 mmol) was added. The mixture was stirred for 2 h at room temperature after which di-*tert*-butyl dicarbonate (0.15 mL, 0.654 mmol) was added. Then HCl (1 M) was added slowly until pH = 3 and the solution was extracted with EtOAc (3 × 10 mL), and washed with brine. The collected organic layer was concentrated to an oil, which was mixed with EDCI·HCl (114.9 mg, 0.599 mmol), HOBT (80.9 mg, 0.599 mmol), and H-Gly-O^{t}Bu·HCl (91.3 mg, 0.545 mmol) in CH_2Cl_2 (10 mL). After stirring for 5 min, DIPEA (0.32 mL, 1.798 mmol) was added. When the reaction was complete, the solution was treated with water (15 mL) and extracted with CH_2Cl_2 (3 × 10 mL). The combined organic phase was washed with brine, dried over Na_2SO_4, filtered, and concentrated. The crude product was purified by column chromatography on silica gel using PE/EtOAc (4:1) to afford target compound **5**: 149 mg, colorless liquid, 75% yield from **3**, 92% ee; $[\alpha]_D^{20}$ = -80.1 (c = 0.28, CH_2Cl_2); ^{1}H NMR (400 MHz, Chloroform-d) *δ* 7.36-7.30 (m, 5H), 6.52 (s, 1H), 5.90 (s, 1H), 5.25 (s, 1H), 3.98-3.92 (m, 1H), 3.85-3.79 (m, 1H), 1.41 (s, 9H), 1.40 (s, 9H); ^{13}C NMR (101 MHz, Chloroform-d) *δ* 170.4, 168.7, 155.4, 138.4, 129.2, 128.6, 127.5, 82.6, 80.3, 58.7, 42.5, 28.5, 28.2; HPLC conditions: DAICEL Chiralpak IC-3 column, *n*-Hexane/*i*-PrOH = 80/20, 210 nm, 0.6 mL/min, t_{major} = 16.604 min, t_{minor} = 24.156 min.

Synthesis of compound 8.

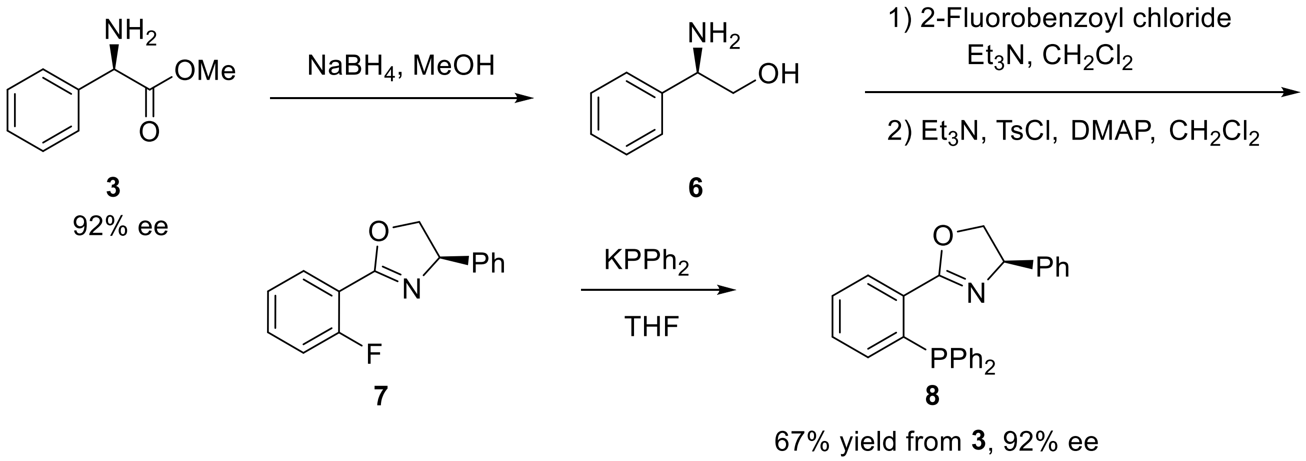

Procedure H[8-10]: Methyl (*R*)-2-amino-2-phenylacetate (**3**, 90 mg, 0.545 mmol) was dissolved in MeOH (5 mL), and $NaBH_4$ (82.4 mg, 2.179 mmol) was added in four portions in an ice bath. The resulting solution was stirred for 2 h at room temperature, then water (5 mL) was added and the mixture was extracted with EtOAc (3 × 15 mL). After being washed and dried, the concentrated liquid was transferred into CH_2Cl_2 (10 mL), then triethylamine (0.23 mL, 1.634 mmol) and pre-prepared 2-fluorobenzoyl chloride (according to literature[10]) were added dropwise to the above solution at 0 ℃. The reaction process was monitored by thin layer chromatograph. When the reaction was complete, it was washed by the addition of HCl (0.2 M, 10 mL), saturated $NaHCO_3$ (10 mL) and brine. The pure intermediate benzamide was gained by column chromatograph (PE/EtOAc = 4:1) as a white solid.

A stirred solution of benzamide, triethylamine (0.23 mL, 1.634 mmol), tosyl chloride (208 mg, 1.090 mmol), and 4-dimethylaminopyridine (7 mg, 0.054 mmol) in CH_2Cl_2 (10 mL) was heated at reflux for 16 h under N_2. Water (0.5 mL) was added to the reaction and the mixture was heated at reflux for 1 h. After being extracted with CH_2Cl_2 (3 × 15 mL) and water, the concentrated residue was purified by column chromatograph using PE/EtOAc (10:1) as eluent to afford compound **7**.

To a solution of **7** in anhydrous tetrahydrofuran (6 mL), potassium diphenylphosphide (0.6 mmol, 0.599 mmol) was added. The resulting solution was heated to 70 ℃ and heated at reflux for 2 h under N_2. Then it was cooled to room temperature, and saturated $NaHCO_3$ (aq. 10 mL) was poured into the mixture. The solution was extracted with CH_2Cl_2 (3 × 10 mL), washed with brine, and dried with anhydrous Na_2SO_4. The desired product **8** was obtained by column chromatograph using PE/EtOAc (10:1): 148 mg, colorless oil, 67% yield from **3**, 92% ee, $[\alpha]_D^{20}$

= -30.2 (c = 0.96, CH_2Cl_2); ^{1}H NMR (400 MHz, Chloroform-*d*) δ 8.02-7.98 (m, 1H), 7.45-7.19 (m, 15H), 6.93-6.87 (s, 3H), 5.22 (t, *J* = 9.6 Hz, 1H), 4.55 (t, *J* = 8.8 Hz, 1H), 3.93 (t, *J* = 8.4 Hz, 1H); ^{13}C NMR (101 MHz, Chloroform-d) δ 165.0, 142.3, 139.2, 138.3, 138.2, 138.1, 138.0, 134.7, 134.5, 134.3, 134.1, 134.0, 131.0, 130.6, 130.56, 129.0, 128.8, 128.77, 128.7, 128.3, 127.4, 126.9, 74.6, 70.4; The enantiomeric excess was deduced from compound **7**, HPLC conditions for compound **7**: DAICEL Chiralpak OC-H column, *n*-Hexane/*i*-PrOH = 90/10, 254 nm, 0.8 mL/min, t_{major} = 18.447 min, t_{minor} = 21.848 min.

Synthesis of compound 9.

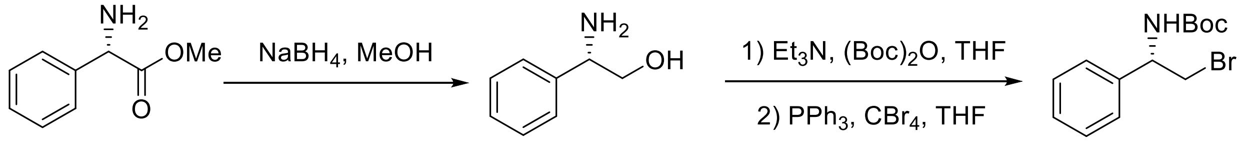

(*S*)-**3** (*S*)-**6** **9**

73% yield from (*S*)-**3**, 93% ee

Procedure I[8-11]: The (*S*)-**3** was further obtained from the hydrogenation of **1a** with (*S,S*)-BenzP* and PMP group removal. Then a 15 mL flask was charged with methyl (*S*)-2-amino-2-phenylacetate ((*S*)-**3**, 85 mg, 0.514 mmol) and MeOH (5 mL), and $NaBH_4$ (77.8 mg, 2.058 mmol) was added in four portions at 0 ℃. The stirring solution was reacted for 2 h at room temperature, then water (5 mL) was added and the mixture was extracted with EtOAc (3 × 15 mL). The collected organic layer was washed with brine, dried over anhydrous Na_2SO_4, and concentrated in vacuo to acquire crude product (*S*)-**6**, which was dissolved in tetrahydrofuran (8 mL). Triethylamine (0.09 mL, 0.617 mmol) and di-*tert*-butyl dicarbonate (146 mg, 0.669 mmol) were then added at 0 ℃. After completion of the reaction, the mixture was diluted with saturated NH_4Cl (aq. 10 mL) and water (10 mL) and extracted with EtOAc (3 × 10 mL). The combined organic phrase was washed with brine, followed by purification with silica gel chromatography using PE/EtOAc (5:1) to afford the *N*-protected intermediate. The corresponding *N*-protected intermediate, triphenylphosphine (203 mg, 0.772 mmol), and carbon tetrabromide (266 mg, 0.772 mmol) were dissolved in anhydrous tetrahydrofuran (8 mL). After 2 h, water (10 mL) was added and the system was extracted with EtOAc (3 × 10 mL). The resulting product **9** was obtained by column chromatograph using PE/EtOAc (5:1) as the eluent: 112 mg, white solid, 73% yield from (*S*)-**3**, 93% ee; $[\alpha]_D^{20}$ = 38.2 (c = 0.26, CH_2Cl_2); ^{1}H NMR (400 MHz, Chloroform-*d*) δ 7.36-7.26 (m, 5H), 5.13 (s, 1H), 5.02(s, 1H), 3.70 (s, 2H), 1.46 (s, 9H); ^{13}C NMR (101 MHz,

Chloroform-*d*) δ 155.2, 139.7, 129.0, 128.3, 126.7, 80.2, 55.0, 37.4, 28.6; HPLC conditions: DAICEL Chiralpak AS-H column, *n*-Hexane/*i*-PrOH = 95/5, 210 nm, 0.8 mL/min, t_{major} = 8.476 min, t_{minor} = 11.593 min.

Supplementary Note 4

Coordination behaviours of (*R,R*)-BenzP*/Ni.

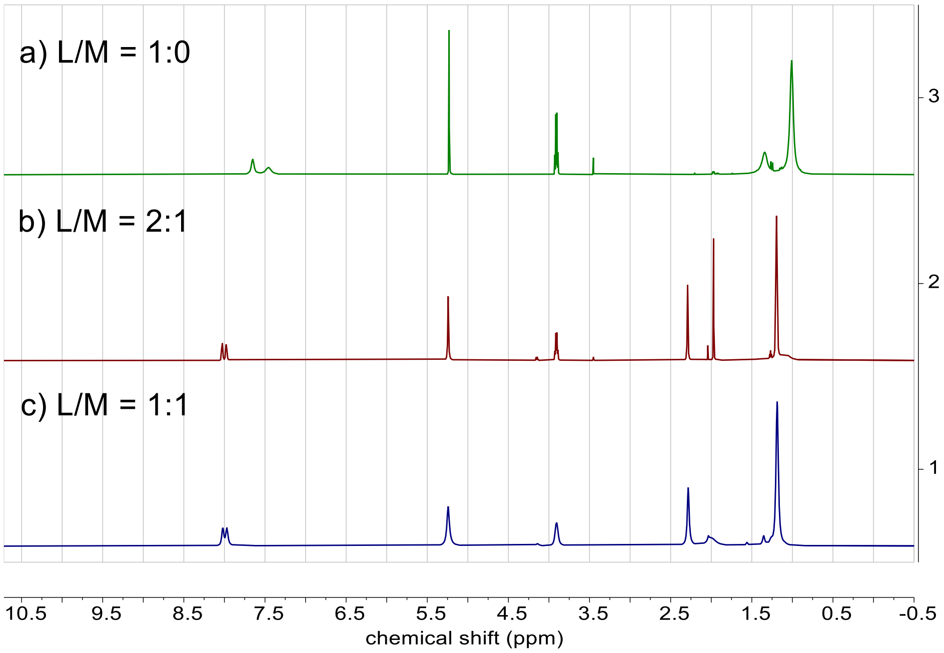

Supplementary Figure 1. The ^1H NMR spectra of the mixture of (*R*,*R*)-BenzP*(L) and $Ni(OAc)_2{\cdot}4H_2O$ (M) in CF_3CD_2OD after stirring for 30 min at 50 °C; a) (*R*,*R*)-BenzP*; b-c) L:M = 2:1; 1:1, respectively.

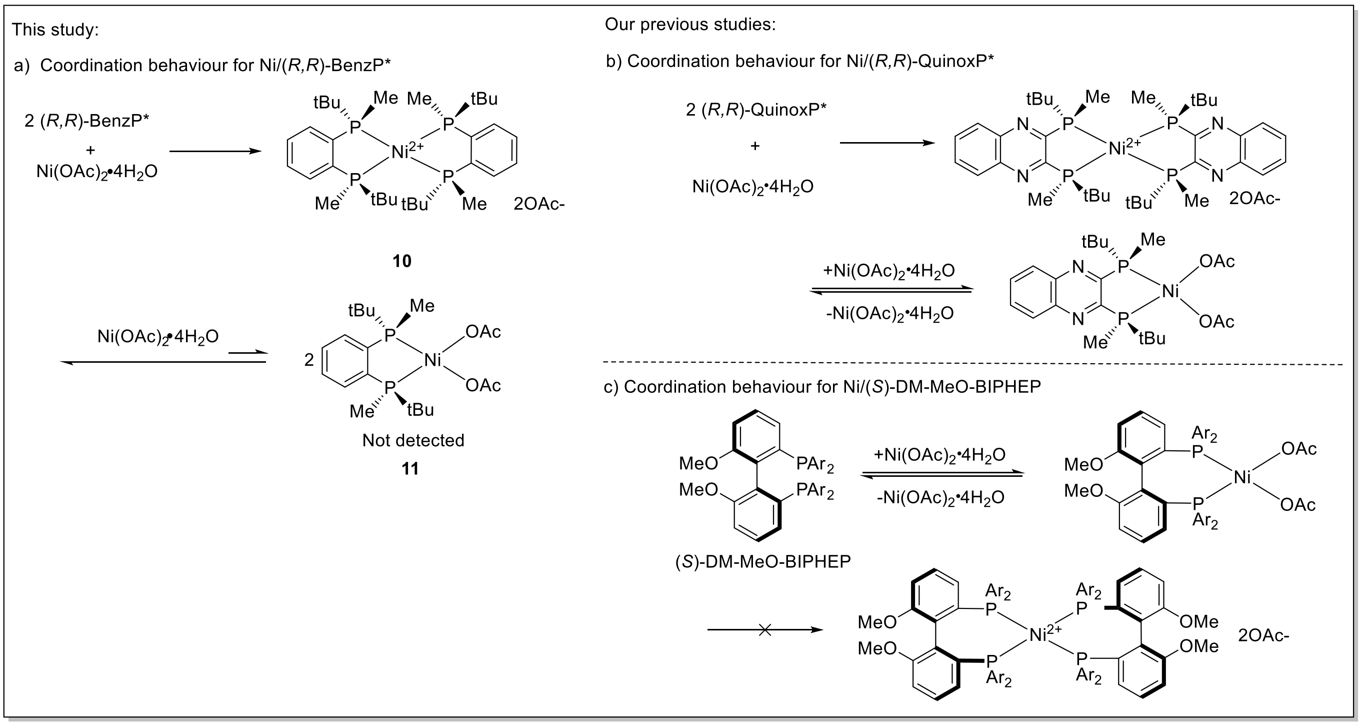

Supplementary Figure 2. Coordination studies for nickel catalysts with different ligands.

To explore the coordination behaviour of (*R,R*)-BenzP*/Ni (L/M), we analyzed the ^{1}H NMR spectra of the catalysts using CF_3CD_2OD as the solvent under different conditions. As illustrated in Supplementary Figure 1, (*R,R*)-BenzP* (L) coordinates with $Ni(OAc)_2 \cdot 4H_2O$ (M) to form a dual-ligand coordinated complex **10** with different L/M ratios in CF_3CD_2OD, with none of the mono-ligand coordinated complex **11** being detected by ^{1}H NMR and HRMS, probably because it exists in trace amounts in an equilibrium with the dominating complex **10** (Supplementary Figure 2). It should be noted that, in our previous studies,[12-13] the coordination behaviour of the nickel catalysts differed with two types of ligands. When using (*R,R*)-QuinoxP* as the ligand, an equilibrium between the dual- and mono-ligand complexes was observed based on an equivalent of nickel salt (Supplementary Figure 2b).[12] Conversely, in the (*S*)-DM-MeO-BIPHEP/Ni system, an equilibrium was seen between the ligand and mono-ligand complex with no dual-ligand complex being observed (Supplementary Figure 2c).[13]

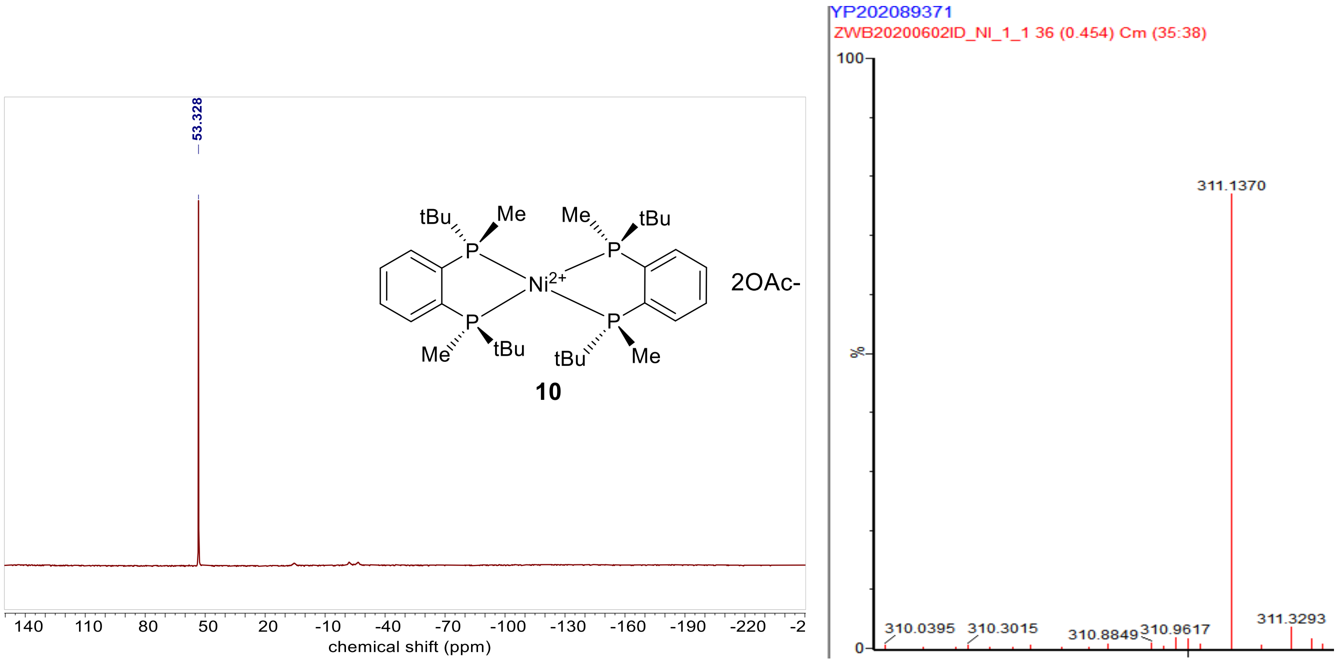

Supplementary Figure 3. The ^{31}P NMR and HRMS spectrum of complex **10**.

Complex **10**: ^{1}H NMR (700 MHz, TFE-*d*3) δ 8.02 (s, 4H), 7.98 (s, 4H), 2.29 (s, 12H), 1.97 (s, 6H), 1.20 (s, 36H); ^{31}P NMR (283 MHz, TFE-*d*3) δ 53.33; HRMS (ESI-MS) Calcd. For $C_{32}H_{56}NiP_4^{2+}$ 311.1338, found: 311.1370.

Determination of complex 12.

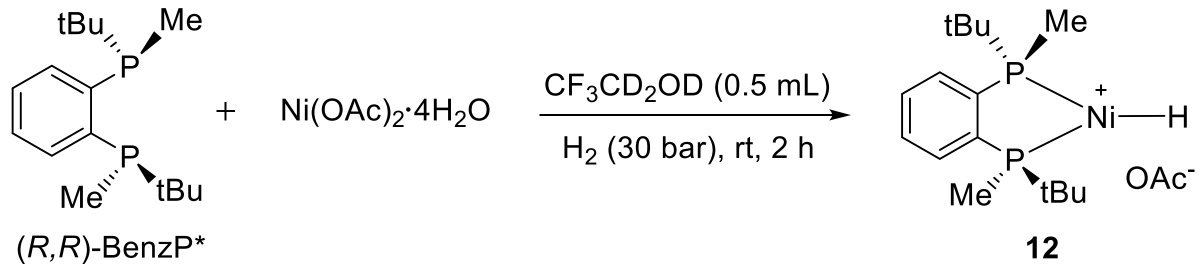

Procedure J: In a glovebox, (*R,R*)-BenzP*(2.82 mg, 0.01 mmol) and $Ni(OAc)_2{\cdot}4H_2O$ (2.48 mg, 0.01 mmol) were dissolved in CF_3CD_2OD (0.5 mL), and then the mixture was stirred for 2 h at room temperature under 30 bar H_2. The obtained orange solution was used for NMR analysis. Complex **12** ^{1}H NMR (400 MHz, TFE-*d*3) δ = -13.52 (t, $^2J_{\text{P-H}}$ = 14.8 Hz; Ni-H), The ratio of complex **12** to other complexes is 1:7 according to the integration of the Ni-H and Ar-H signals; ^{31}P NMR (162 MHz, TFE-*d*3) δ 52.46, Notablely, the peak of complex **12** at the ^{31}P spectrum of the hydrogenated complex could not been found, it appears the same as the spectrum for complex **10**. This may be because complex **12** exists in trace amounts and/or is not stable and decomposes to complex **10**.

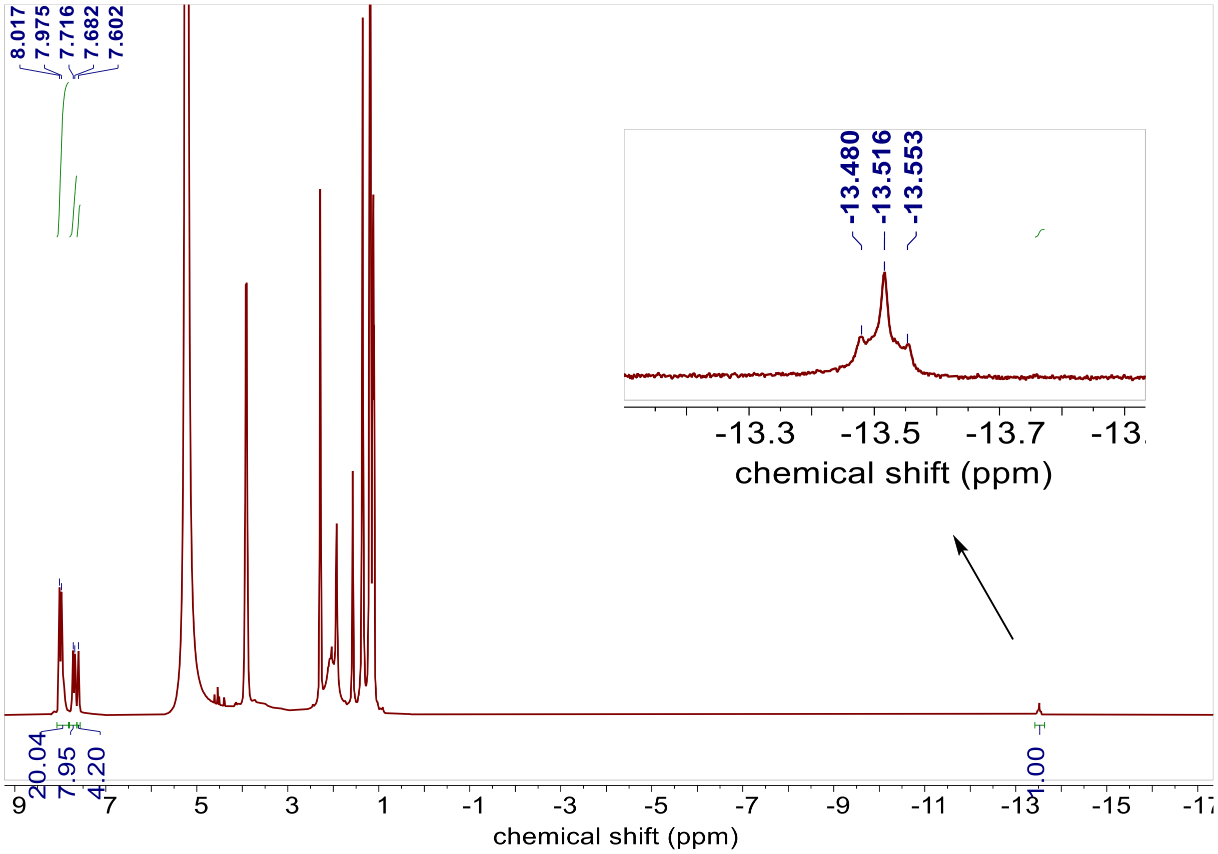

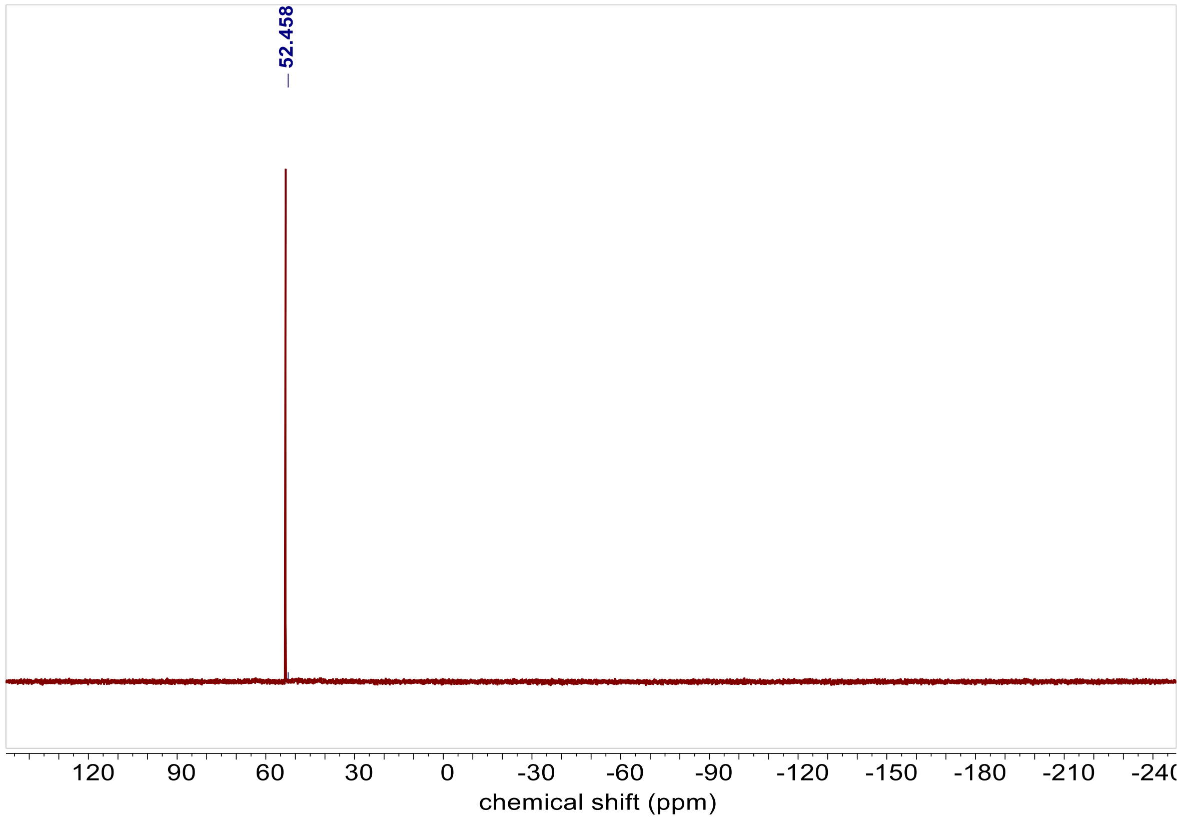

Supplementary Figure 4. ^{1}H NMR and ^{31}P NMR spectrum of the hydrogenation of the mixture of (*R,R*)-BenzP* (L) and $Ni(OAc)_2{\cdot}4H_2O$ (M) (L/M = 1:1).

Study of *Z/E* interconversion of 1a.

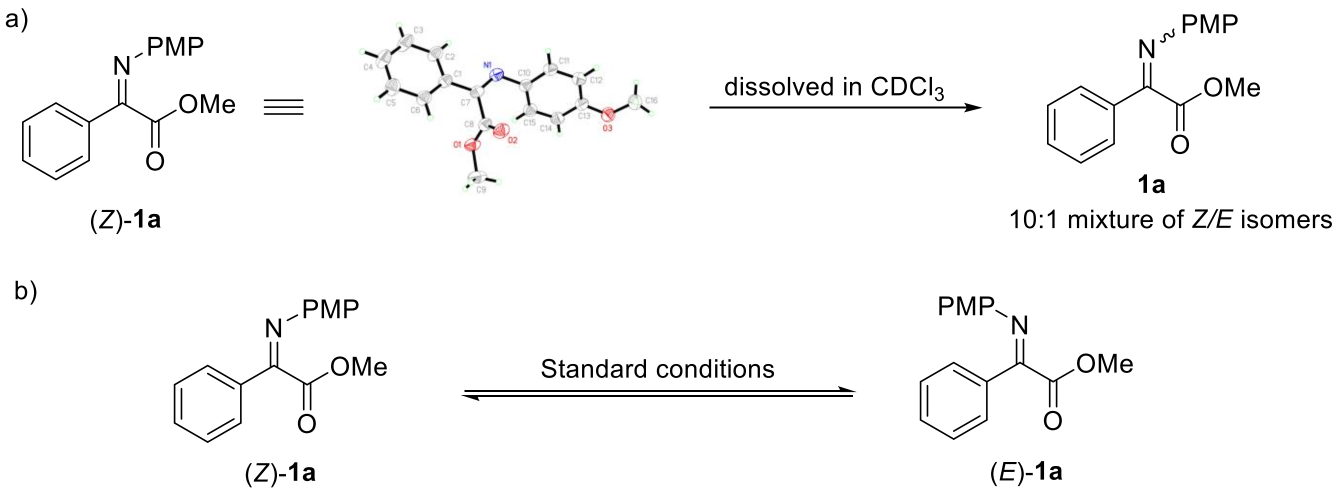

Supplementary Figure 5. Study of *Z/E* interconversion of 1a.

The crystal (*Z*)-**1a** was crystalized from CH_2Cl_2/PE, and its absolute configuration was confirmed by X-ray crystallographic analysis. The crystal was dissolved in $CDCl_3$ and the ^{1}H NMR spectrum of a crystal of (*Z*)-**1a** dissolved in the solvent showed a mixture of *Z*/*E* isomers, indicating that there is rapid interconversion between the two isomers (Supplementary Figure 5).

^{1}H NMR spectrum of the crystal (*Z*)-**1a** in $CDCl_3$: 10:1 mixture of geometric isomers; ^{1}H NMR (400 MHz, Chloroform-*d*) **Z** isomer (major) δ 7.83 (d, J = 8.4 Hz, 2H), 7.54-7.40 (m, 3H), 6.95 (d, J = 8.8 Hz, 2H), 6.86 (d, J = 8.8 Hz, 2H), 3.80 (s, 3H), 3.68 (s, 3H); minor isomer δ 6.74-6.68 (m, 4H), 3.93 (s, 3H), 3.73 (s, 3H), other resonances of the minor isomer are obscured.

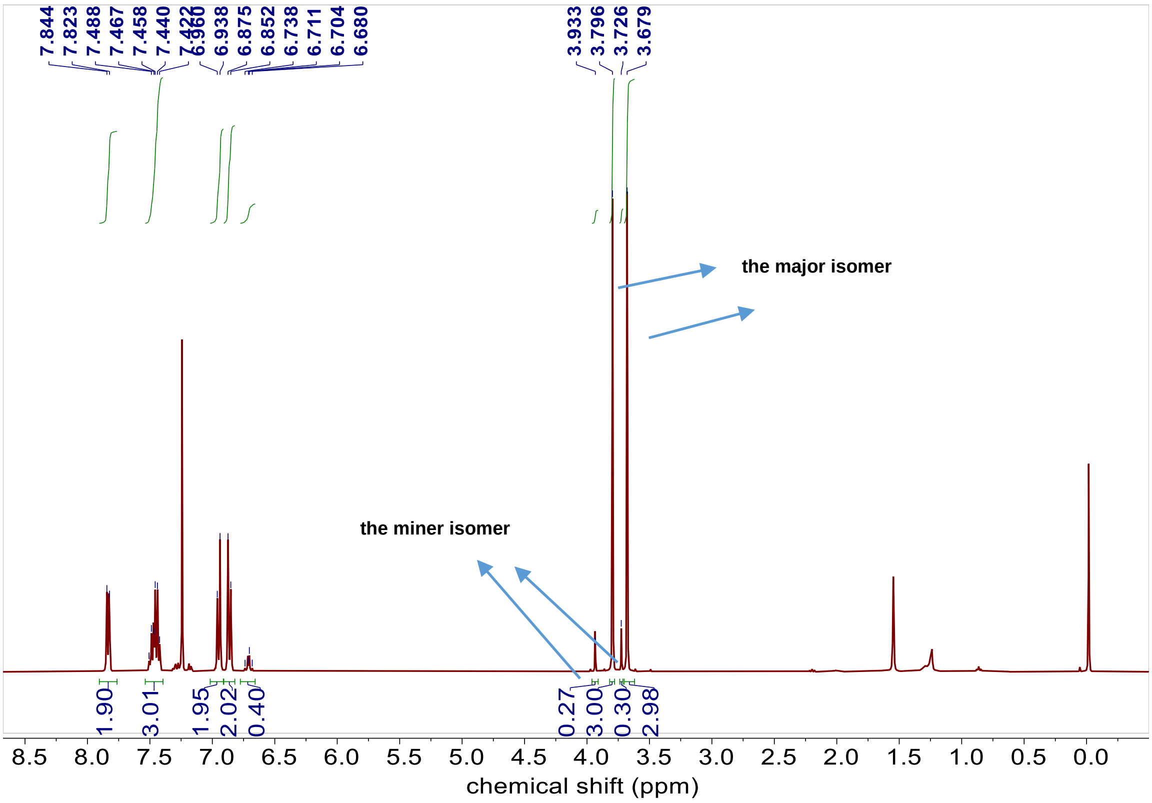

7.844
7.823
7.488
7.467
7.458
7.440
7.422
6.960
6.938
6.875
6.852
6.738
6.711
6.704
6.680
3.933
3.796
3.726
3.679
the major isomer
the miner isomer
1.90
3.01
1.95
2.02
0.40
0.27
3.00
0.30
2.98
8.5 8.0 7.5 7.0 6.5 6.0 5.5 5.0 4.5 4.0 3.5 3.0 2.5 2.0 1.5 1.0 0.5 0.0
chemical shift (ppm)

Deuterium-labeling experiments.

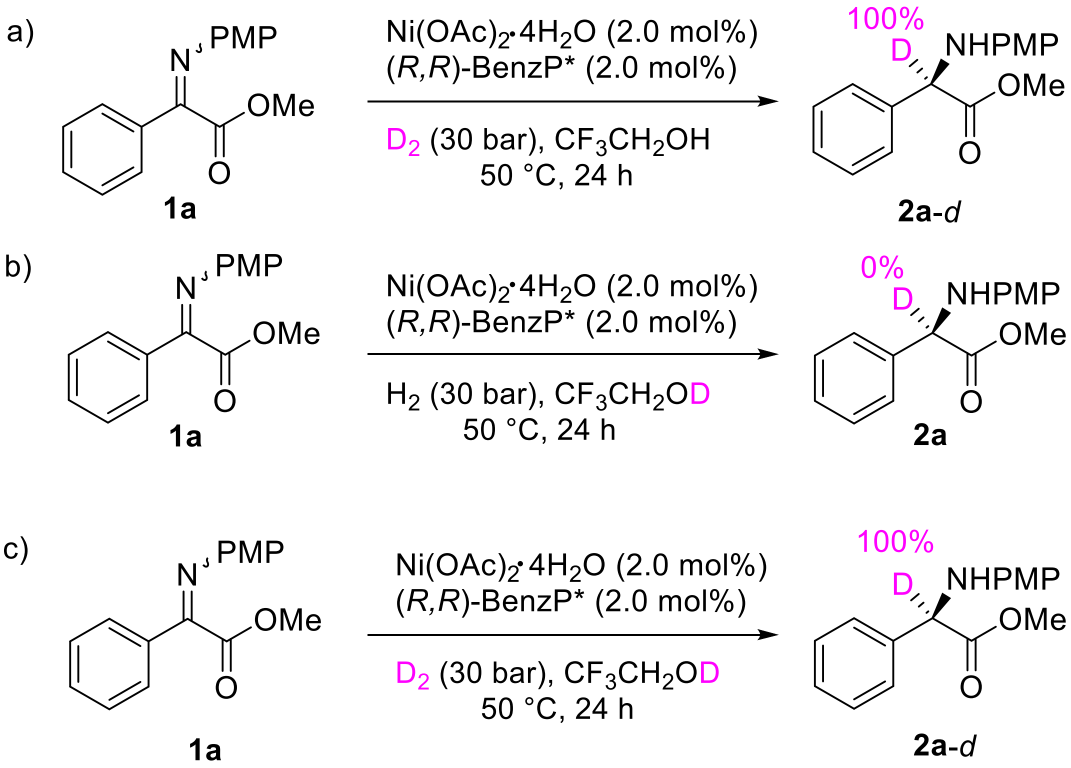

Supplementary Figure 6. Deuterium-labeling experiments.

The deuterium labeling experiments were conducted using D_2 and CF_3CH_2OD to probe the reaction pathway of the asymmetric hydrogenation. Under a D_2 atmosphere, deuterium was completely located at the chiral carbon atom (Supplementary Figure 6a). When H_2 and CF_3CH_2OD were used, no deuterated product was found (Supplementary Figure 6b). The product also showed a deuterium substitution rate of 100% using D_2 and CF_3CH_2OD (Supplementary Figure 6c).

Compound **2a**-*d*: ^{1}H NMR (400 MHz, Chloroform-*d*) δ 7.50 (d, J = 7.2 Hz, 2H), 7.43-7.28 (m, 3H), 6.74 (d, J = 7.2 Hz, 2H), 6.55 (d, J = 7.2 Hz, 2H), 4.67 (s, 1H), 3.72 (s, 3H), 3.71 (s, 3H).

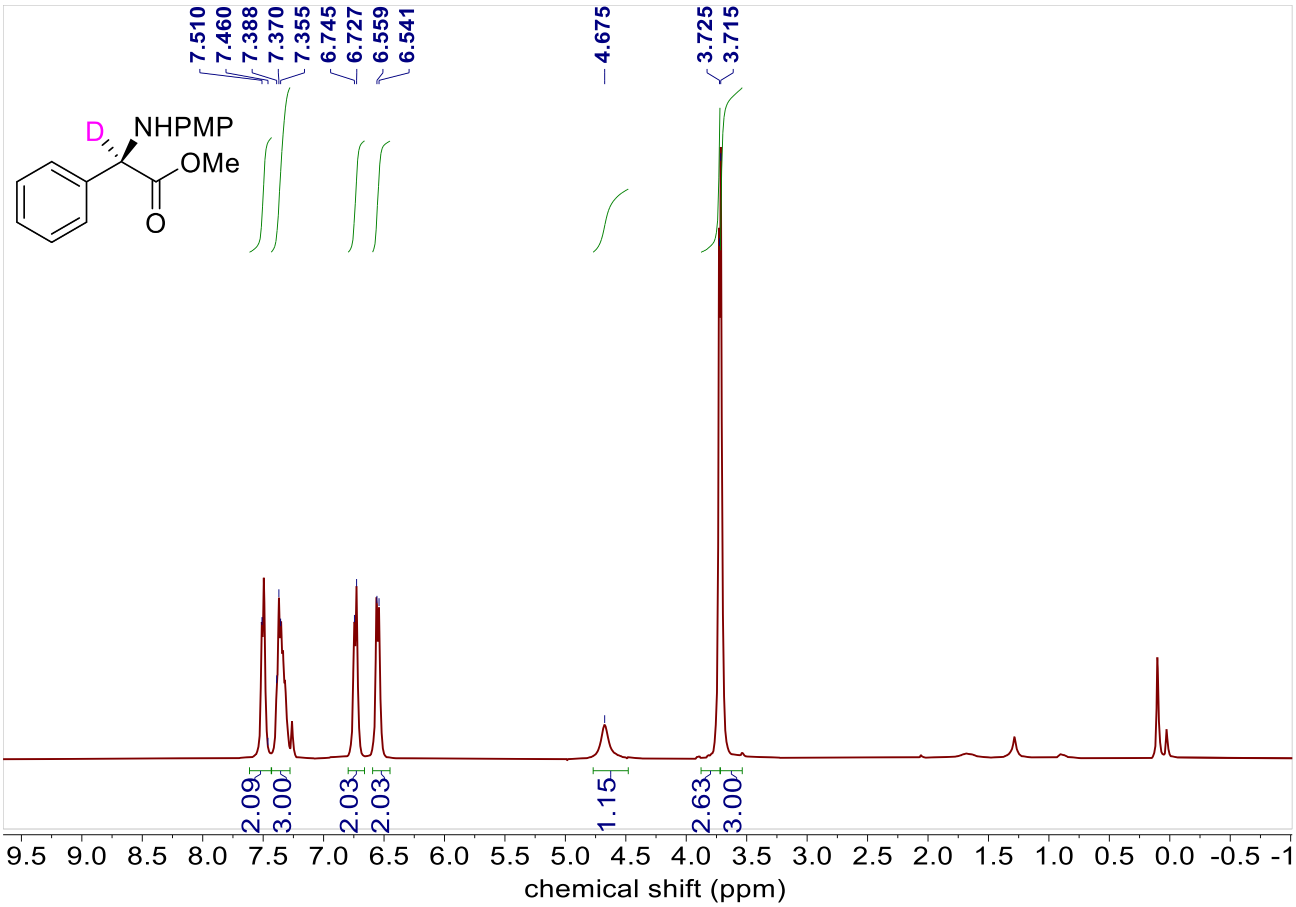
7.510
7.460
7.388
7.370
7.355
6.745
6.727
6.559
6.541
4.675
3.725
3.715
D
NHPMP
OMe
O
2.09
3.00
2.03
2.03
1.15
2.63
3.00
9.5 9.0 8.5 8.0 7.5 7.0 6.5 6.0 5.5 5.0 4.5 4.0 3.5 3.0 2.5 2.0 1.5 1.0 0.5 0.0 -0.5 -1
chemical shift (ppm)

Computed profile of relative free energy for 4 pathways.

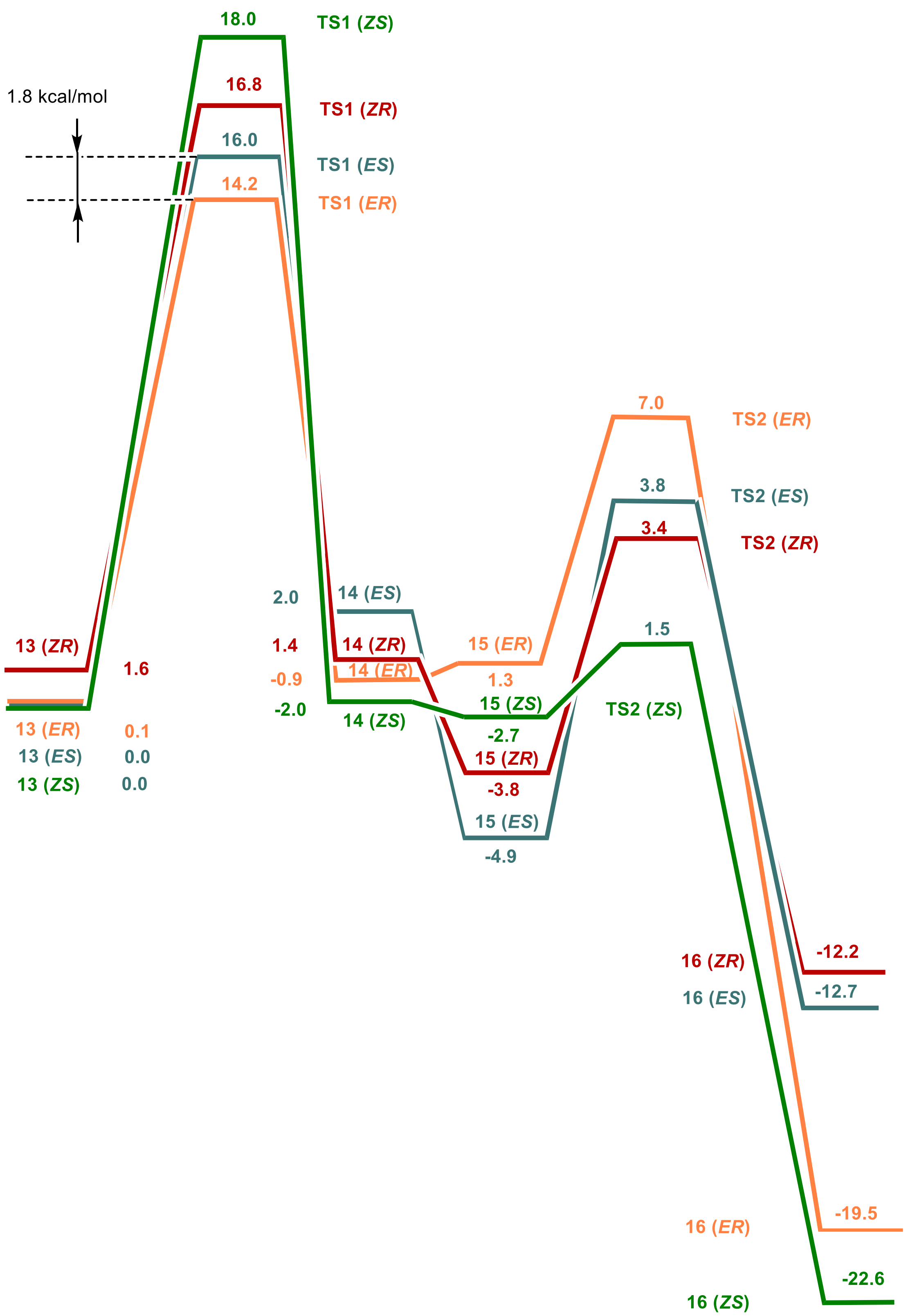

Supplementary Figure 7. Potential energy profile for the computed catalytic pathways.

Detail on the intramolecular interactions in TS1 (*ER*) and TS1 (*ES*)

Analysis of the intramolecular stabilizing interactions in the competing transition states of the most viable cationic mechanism (Supplementary Table 3) suggests that the main difference leading to the notable difference in their energies is the interaction of the carboxymethyl substituent with the hydride being transferred from Ni to carbon. This is further illustrated in Figure 4 – if in the *S* transition state, only one quite long H…O contact of this kind can be found. In the *R* transition state the carboxymethyl group is totally involved in supporting the stability of the transition state via appropriately distanced intramolecular interactions (the distance C(carbonyl)-H)hydride is 2.03Å.

Supplementary Table 3. Structural Reasons for Enantioselection.

Compound	Interatomic distances between catalyst and coordinated substrate falling into range of the corresponding attractive interactions			
	C-H···H-C	C-H···π	C-H···O	C-H···N
R	2.41, 2.52, 2.41, 2.39, 2.58	2.82, 3.20	2.65, 2.76, 2.55*, 2.66*	2.63
S	2.20, 2.63, 2.28, 2.91	2.52, 2.69	2.52, 2.85, 2.86, 2.84*	2.82

*****with the hydride that is being transferred

Details of the configuration with coplanar orientation of Ni-C and Ni-H bonds.

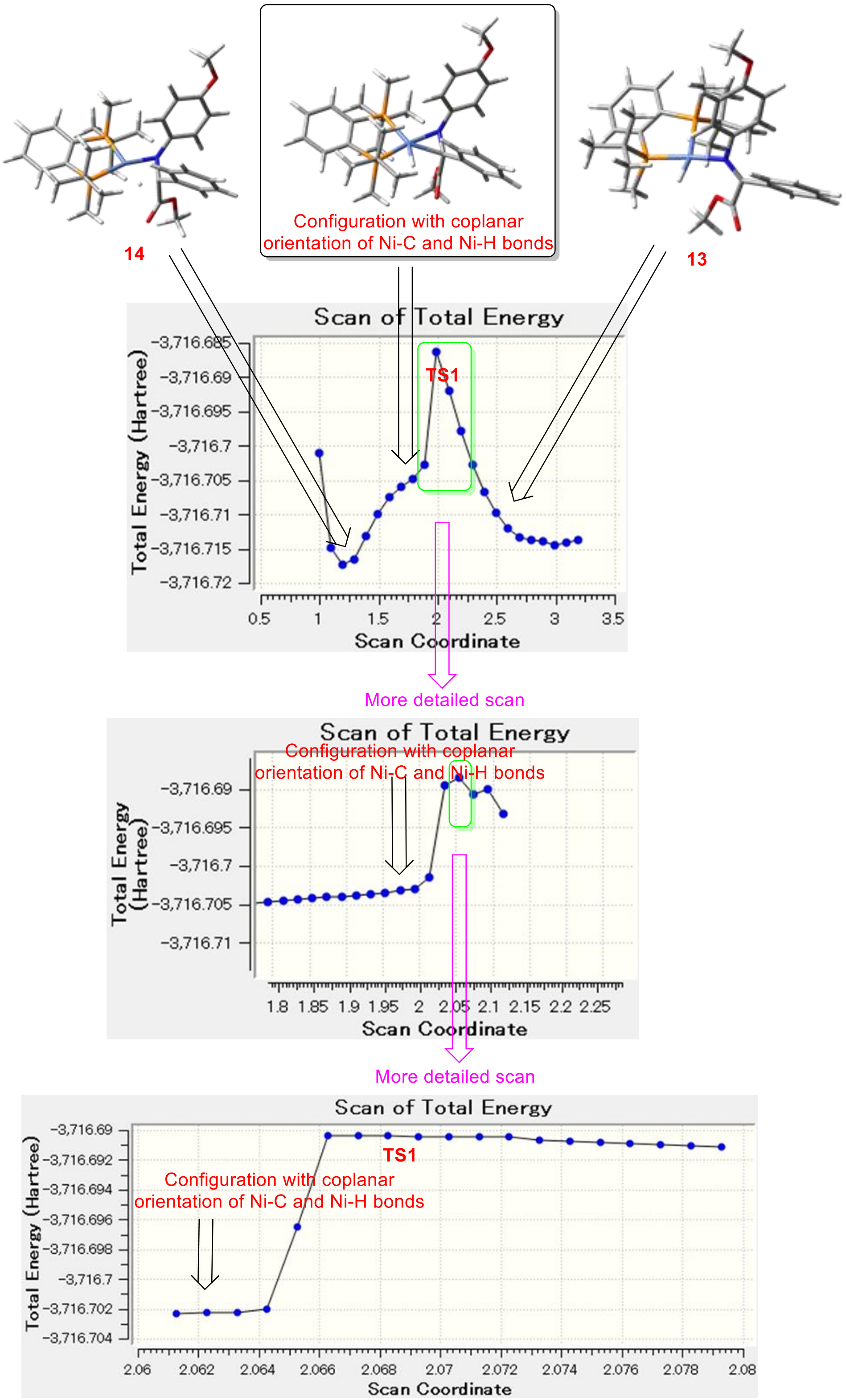

Supplementary Figure 8. The details of the configuration with coplanar orientation of Ni-C and Ni-H bonds.

The configuration with coplanar orientation of Ni-C and Ni-H bonds was found during the calculation studies (Supplementary Figure 8). The details can be seen in the below figure (Supplementary Figure 8). There are numerous examples of very low barrier (1-2 kcal/mol) or even barrierless migratory insertions in transition-metal catalyzed asymmetric hydrogenations.[14]

In addition, scanning via several different methods was also examined (Supplementary Figure 9). All these methods agree that in order for migratory insertion to be favorable, a configuration with an almost equal number of Ni-C and Ni-N bonds must form.

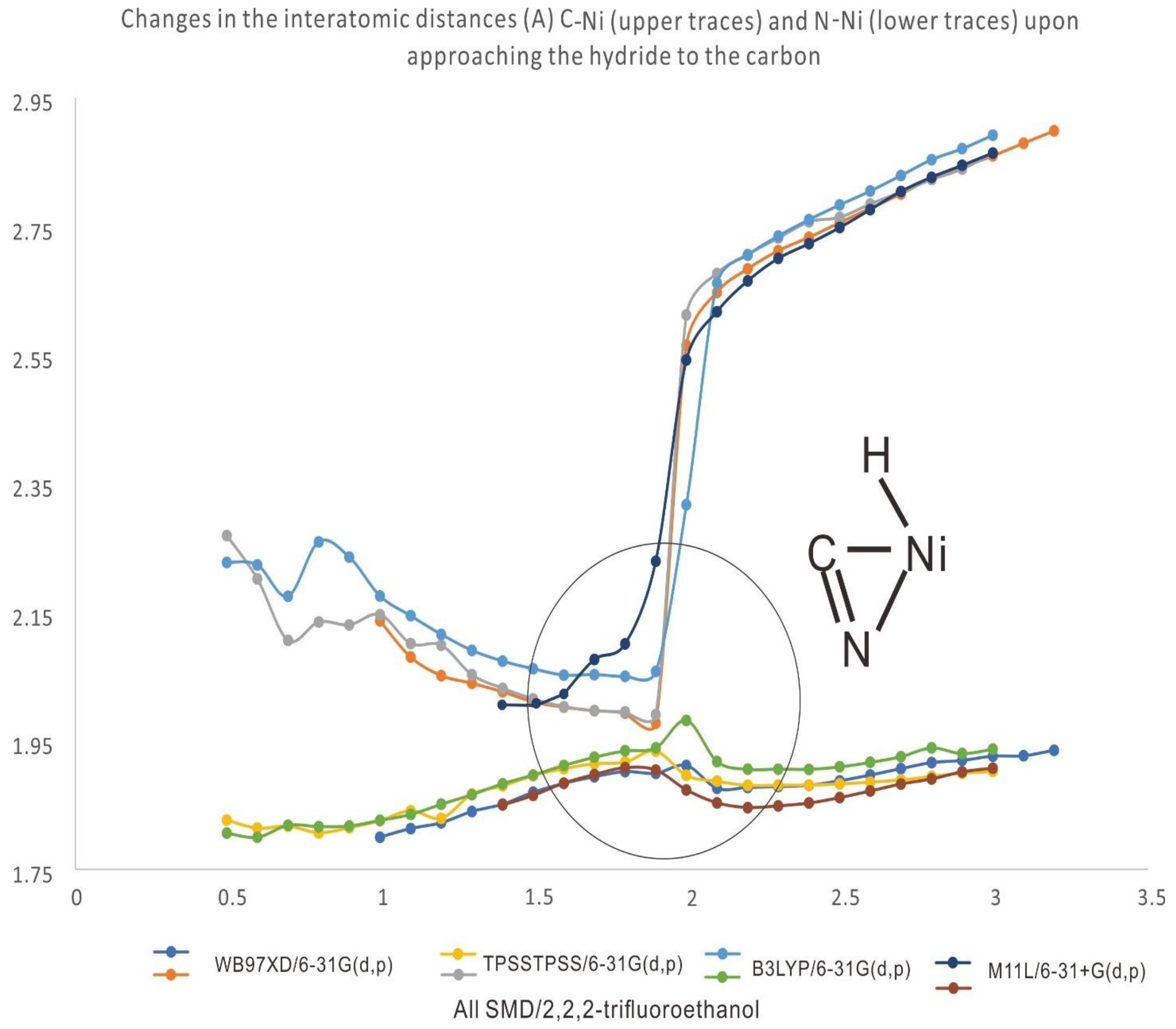

Supplementary Figure 9. The scanning by several different methods.

Computational details.

Computations were carried out using the long-range corrected hybrid functional with damped atom-atom dispersion (WB97XD),[15] as implemented in the GAUSSIAN 09 software package.[16] All atoms were modeled at the 6-31G(d,p) level of theory.[17-21] The solvent effect was accounted for by carrying out optimizations in the SMD force field[22] (2,2,2-trifluoroethanol).

Supplementary Note 5

X-ray analysis data

Methyl (*Z*)-2-((4-methoxyphenyl)imino)-2-phenylacetate ((*Z*)-1a)

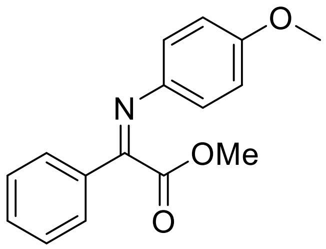

CCDC 2011640

checkCIF/PLATON report

Structure factors have been supplied for datablock(s) t_a

THIS REPORT IS FOR GUIDANCE ONLY. IF USED AS PART OF A REVIEW PROCEDURE FOR PUBLICATION, IT SHOULD NOT REPLACE THE EXPERTISE OF AN EXPERIENCED CRYSTALLOGRAPHIC REFEREE.

No syntax errors found. CIF dictionary Interpreting this report

Datablock: t_a

```
Bond precision:  C-C = 0.0043 A          Wavelength=1.54178

Cell:          a=9.0312(5)       b=9.1371(5)      c=10.3007(6)
               alpha=78.952(3)   beta=70.057(3)   gamma=65.439(2)
Temperature:   297 K

                Calculated                 Reported
Volume          725.50(7)                  725.50(7)
Space group     P -1                       P -1
Hall group      -P 1                       -P 1
Moiety formula  C16 H15 N O3               C16 H15 N O3
Sum formula     C16 H15 N O3               C16 H15 N O3
Mr              269.29                     269.29
Dx,g cm-3       1.233                      1.233
Z               2                          2
Mu (mm-1)       0.698                      0.698
F000            284.0                      284.0
F000'           284.90
h,k,lmax        10,10,12                   10,10,12
Nref            2648                       2610
Tmin,Tmax       0.846,0.870                0.527,0.753
Tmin'           0.846

Correction method= # Reported T Limits: Tmin=0.527 Tmax=0.753
AbsCorr = ?

Data completeness= 0.986          Theta(max)= 68.174

R(reflections)= 0.0681( 1752)     wR2(reflections)= 0.2253( 2610)

S = 1.092                  Npar= 183
```

The following ALERTS were generated. Each ALERT has the format
test-name_ALERT_alert-type_alert-level.
Click on the hyperlinks for more details of the test.

Supplementary Figure 10.

Alert level C

```
PLAT052_ALERT_1_C Info on Absorption Correction Method   Not Given      Please Do !
PLAT340_ALERT_3_C Low Bond Precision on  C-C Bonds ...............    0.00429 Ang.
PLAT906_ALERT_3_C Large K Value in the Analysis of Variance ......      7.229 Check
PLAT911_ALERT_3_C Missing FCF Refl Between Thmin & STh/L=    0.600         30 Report
```

Alert level G

```
PLAT072_ALERT_2_G SHELXL First  Parameter in WGHT  Unusually Large       0.12 Report
PLAT883_ALERT_1_G No Info/Value for _atom_sites_solution_primary .     Please Do !
PLAT912_ALERT_4_G Missing # of FCF Reflections Above STh/L=  0.600          8 Note
PLAT941_ALERT_3_G Average HKL Measurement Multiplicity ...........        3.1 Low
PLAT978_ALERT_2_G Number C-C Bonds with Positive Residual Density.          0 Info
PLAT992_ALERT_5_G Repd & Actual _reflns_number_gt Values Differ by         10 Check
```

```
   0 ALERT level A = Most likely a serious problem - resolve or explain
   0 ALERT level B = A potentially serious problem, consider carefully
   4 ALERT level C = Check. Ensure it is not caused by an omission or oversight
   6 ALERT level G = General information/check it is not something unexpected

   2 ALERT type 1 CIF construction/syntax error, inconsistent or missing data
   2 ALERT type 2 Indicator that the structure model may be wrong or deficient
   4 ALERT type 3 Indicator that the structure quality may be low
   1 ALERT type 4 Improvement, methodology, query or suggestion
   1 ALERT type 5 Informative message, check
```

It is advisable to attempt to resolve as many as possible of the alerts in all categories. Often the minor alerts point to easily fixed oversights, errors and omissions in your CIF or refinement strategy, so attention to these fine details can be worthwhile. In order to resolve some of the more serious problems it may be necessary to carry out additional measurements or structure refinements. However, the purpose of your study may justify the reported deviations and the more serious of these should normally be commented upon in the discussion or experimental section of a paper or in the "special_details" fields of the CIF. checkCIF was carefully designed to identify outliers and unusual parameters, but every test has its limitations and alerts that are not important in a particular case may appear. Conversely, the absence of alerts does not guarantee there are no aspects of the results needing attention. It is up to the individual to critically assess their own results and, if necessary, seek expert advice.

Publication of your CIF in IUCr journals

A basic structural check has been run on your CIF. These basic checks will be run on all CIFs submitted for publication in IUCr journals (*Acta Crystallographica*, *Journal of Applied Crystallography*, *Journal of Synchrotron Radiation*); however, if you intend to submit to *Acta Crystallographica Section C* or *E* or *IUCrData*, you should make sure that full publication checks are run on the final version of your CIF prior to submission.

Publication of your CIF in other journals

Please refer to the *Notes for Authors* of the relevant journal for any special instructions relating to CIF submission.

Supplementary Figure 11.

PLATON version of 04/06/2020; check.def file version of 02/06/2020

Datablock t_a - ellipsoid plot

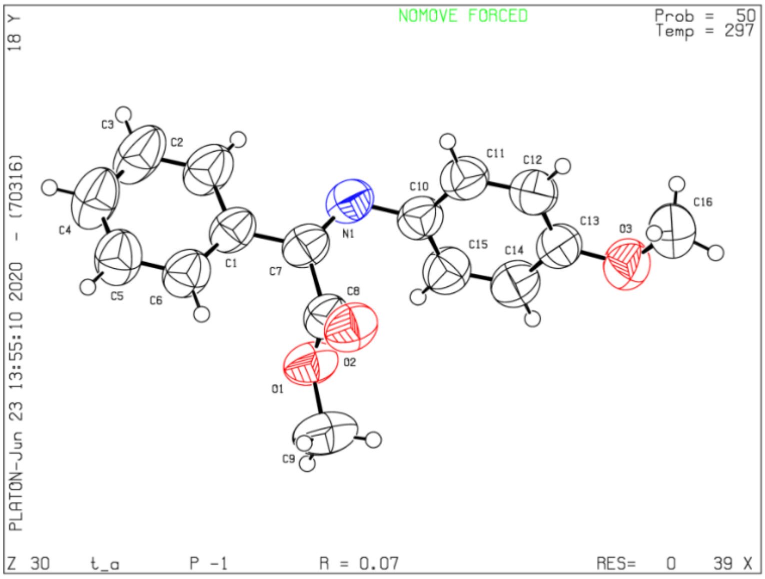

Supplementary Figure 12.

Methyl (*R*)-2-((4-methoxyphenyl)amino)-2-phenylacetate (2a)

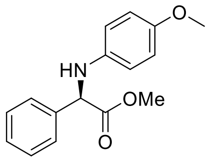

CCDC 2011648

checkCIF/PLATON report

Structure factors have been supplied for datablock(s) t_a

THIS REPORT IS FOR GUIDANCE ONLY. IF USED AS PART OF A REVIEW PROCEDURE FOR PUBLICATION, IT SHOULD NOT REPLACE THE EXPERTISE OF AN EXPERIENCED CRYSTALLOGRAPHIC REFEREE.

No syntax errors found. CIF dictionary Interpreting this report

Datablock: t_a

```
Bond precision:  C-C = 0.0083 A          Wavelength=1.54178

Cell:           a=14.2048(16)    b=9.798(3)          c=10.6592(16)
                alpha=90         beta=108.155(8)     gamma=90
Temperature:    296 K

                Calculated                 Reported
Volume          1409.7(5)                  1409.7(5)
Space group     C c                        C c
Hall group      C -2yc                     C -2yc
Moiety formula  C16 H17 N O3               C16 H17 N O3
Sum formula     C16 H17 N O3               C16 H17 N O3
Mr              271.31                     271.30
Dx,g cm-3       1.278                      1.278
Z               4                          4
Mu (mm-1)       0.719                      0.719
F000            576.0                      576.0
F000'           577.79
h,k,lmax        17,11,12                   17,11,12
Nref            2603[ 1304]                1797
Tmin,Tmax       0.866,0.898                0.681,0.753
Tmin'           0.866

Correction method= # Reported T Limits: Tmin=0.681 Tmax=0.753
AbsCorr = ?

Data completeness= 1.38/0.69       Theta(max)= 68.357

R(reflections)= 0.0390( 1264)      wR2(reflections)= 0.2030( 1797)

S = 1.310                  Npar= 187
```

The following ALERTS were generated. Each ALERT has the format
test-name_ALERT_alert-type_alert-level.
Click on the hyperlinks for more details of the test.

Supplementary Figure 13.

Alert level B

```
PLAT029_ALERT_3_B _diffrn_measured_fraction_theta_full value Low .      0.952 Why?
PLAT915_ALERT_3_B No Flack x Check Done: Low Friedel Pair Coverage        43 %
```

Alert level C

```
DIFMN02_ALERT_2_C  The minimum difference density is < -0.1*ZMAX*0.75
            _refine_diff_density_min given =      -0.689
            Test value =      -0.600
DIFMN03_ALERT_1_C  The minimum difference density is < -0.1*ZMAX*0.75
            The relevant atom site should be identified.
PLAT052_ALERT_1_C Info on Absorption Correction Method   Not Given     Please Do !
PLAT089_ALERT_3_C Poor Data / Parameter Ratio (Zmax < 18) ........       6.61 Note
PLAT098_ALERT_2_C Large Reported Min.  (Negative) Residual Density      -0.69 eA-3
PLAT340_ALERT_3_C Low Bond Precision on  C-C Bonds ..............    0.00829 Ang.
PLAT420_ALERT_2_C D-H Without Acceptor       N1        --H1      .     Please Check
PLAT911_ALERT_3_C Missing FCF Refl Between Thmin & STh/L=    0.600         62 Report
PLAT913_ALERT_3_C Missing # of Very Strong Reflections in FCF ....         15 Note
```

Alert level G

```
PLAT072_ALERT_2_G SHELXL First  Parameter in WGHT  Unusually Large       0.12 Report
PLAT792_ALERT_1_G Model has Chirality at C7           (Polar  SPGR)         R Verify
PLAT883_ALERT_1_G No Info/Value for _atom_sites_solution_primary .     Please Do !
PLAT912_ALERT_4_G Missing # of FCF Reflections Above STh/L=  0.600          6 Note
PLAT941_ALERT_3_G Average HKL Measurement Multiplicity ..........         2.6 Low
PLAT978_ALERT_2_G Number C-C Bonds with Positive Residual Density.          0 Info
PLAT992_ALERT_5_G Repd & Actual _reflns_number_gt Values Differ by          1 Check
```

0 **ALERT level A** = Most likely a serious problem - resolve or explain
2 **ALERT level B** = A potentially serious problem, consider carefully
9 **ALERT level C** = Check. Ensure it is not caused by an omission or oversight
7 **ALERT level G** = General information/check it is not something unexpected

4 ALERT type 1 CIF construction/syntax error, inconsistent or missing data
5 ALERT type 2 Indicator that the structure model may be wrong or deficient
7 ALERT type 3 Indicator that the structure quality may be low
1 ALERT type 4 Improvement, methodology, query or suggestion
1 ALERT type 5 Informative message, check

Supplementary Figure 14.

It is advisable to attempt to resolve as many as possible of the alerts in all categories. Often the minor alerts point to easily fixed oversights, errors and omissions in your CIF or refinement strategy, so attention to these fine details can be worthwhile. In order to resolve some of the more serious problems it may be necessary to carry out additional measurements or structure refinements. However, the purpose of your study may justify the reported deviations and the more serious of these should normally be commented upon in the discussion or experimental section of a paper or in the "special_details" fields of the CIF. checkCIF was carefully designed to identify outliers and unusual parameters, but every test has its limitations and alerts that are not important in a particular case may appear. Conversely, the absence of alerts does not guarantee there are no aspects of the results needing attention. It is up to the individual to critically assess their own results and, if necessary, seek expert advice.

Publication of your CIF in IUCr journals

A basic structural check has been run on your CIF. These basic checks will be run on all CIFs submitted for publication in IUCr journals (*Acta Crystallographica*, *Journal of Applied Crystallography*, *Journal of Synchrotron Radiation*); however, if you intend to submit to *Acta Crystallographica Section C* or *E* or *IUCrData*, you should make sure that full publication checks are run on the final version of your CIF prior to submission.

Publication of your CIF in other journals

Please refer to the *Notes for Authors* of the relevant journal for any special instructions relating to CIF submission.

PLATON version of 04/06/2020; check.def file version of 02/06/2020

Supplementary Figure 15.

Datablock t_a - ellipsoid plot

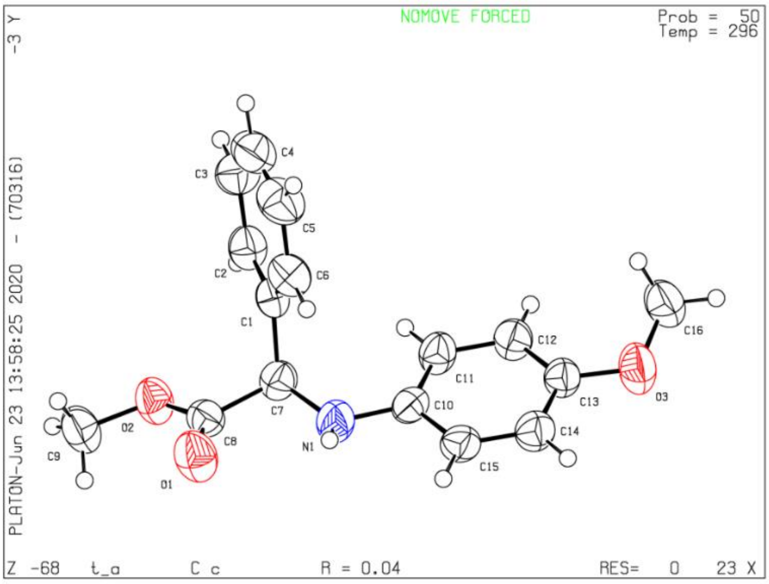

Supplementary Figure 16.

Supplementary Figures

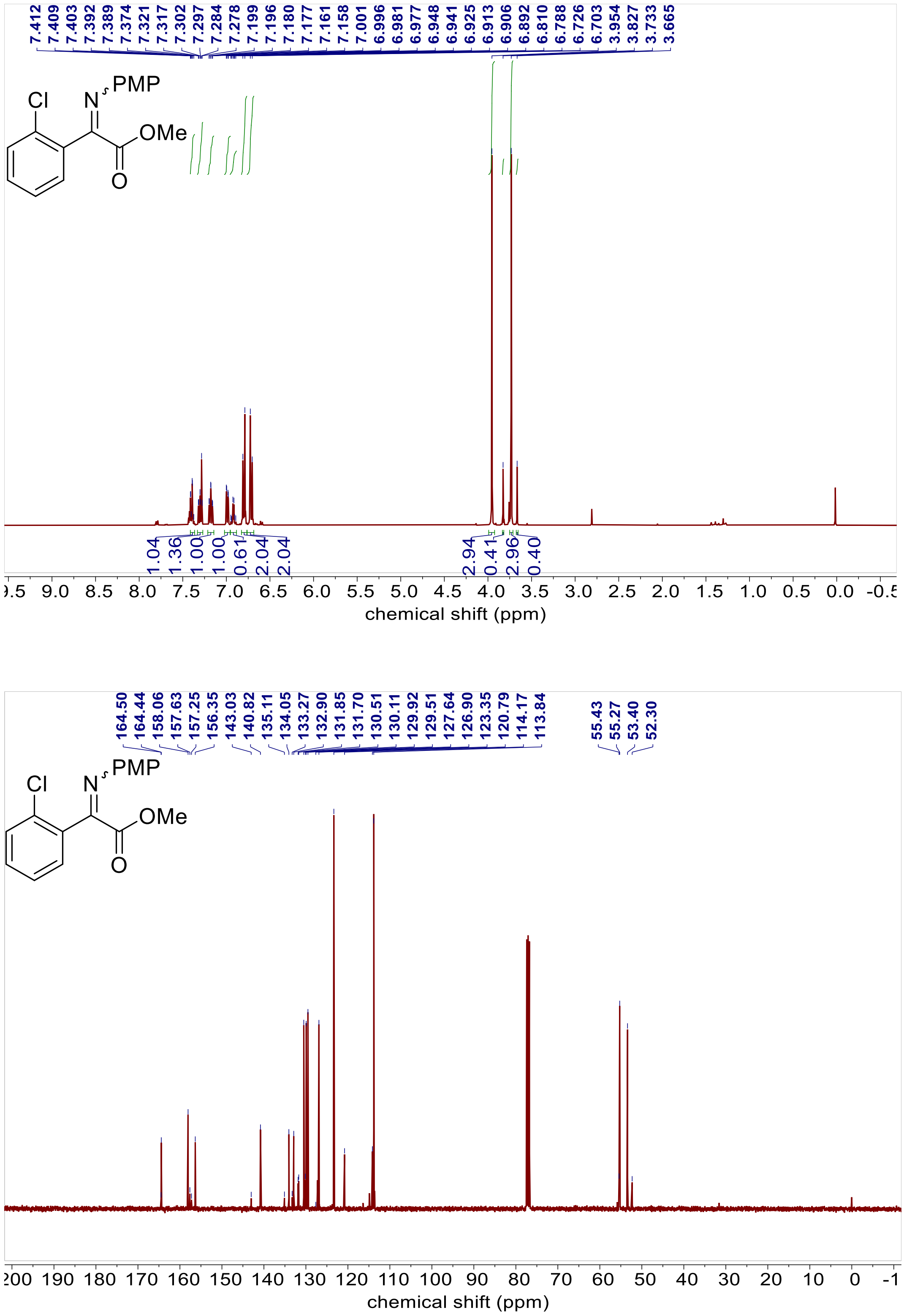

Supplementary Figure 17. ^{1}H NMR & ^{13}C NMR spectra of compound **1c** in $CDCl_3$

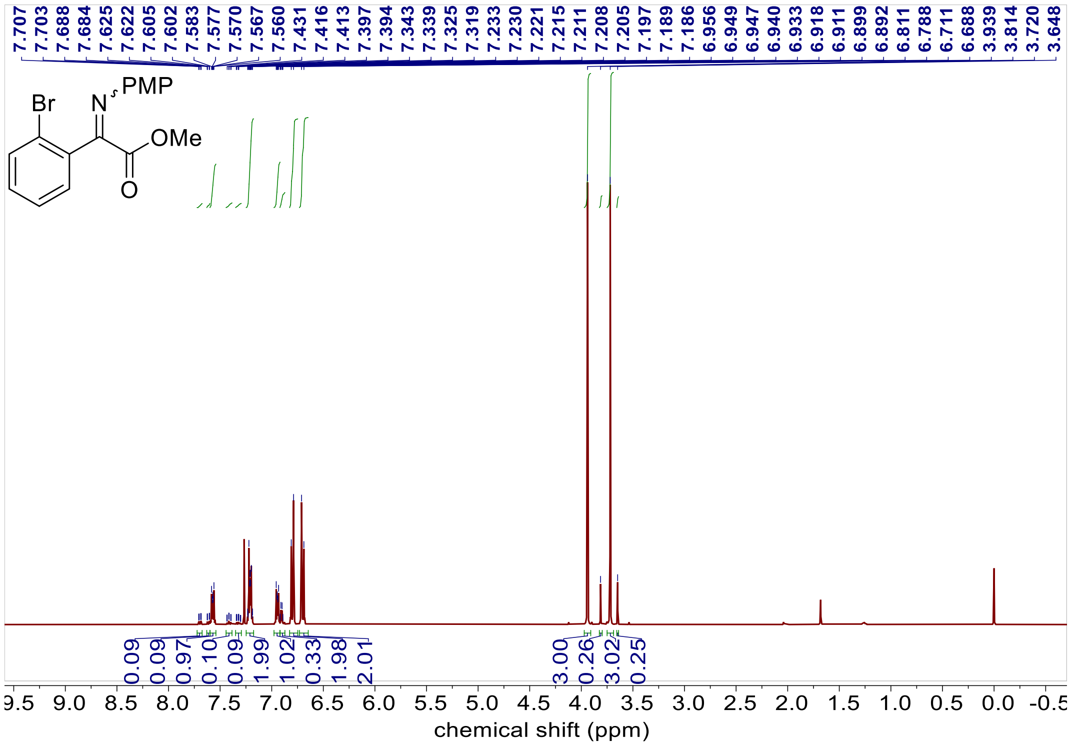

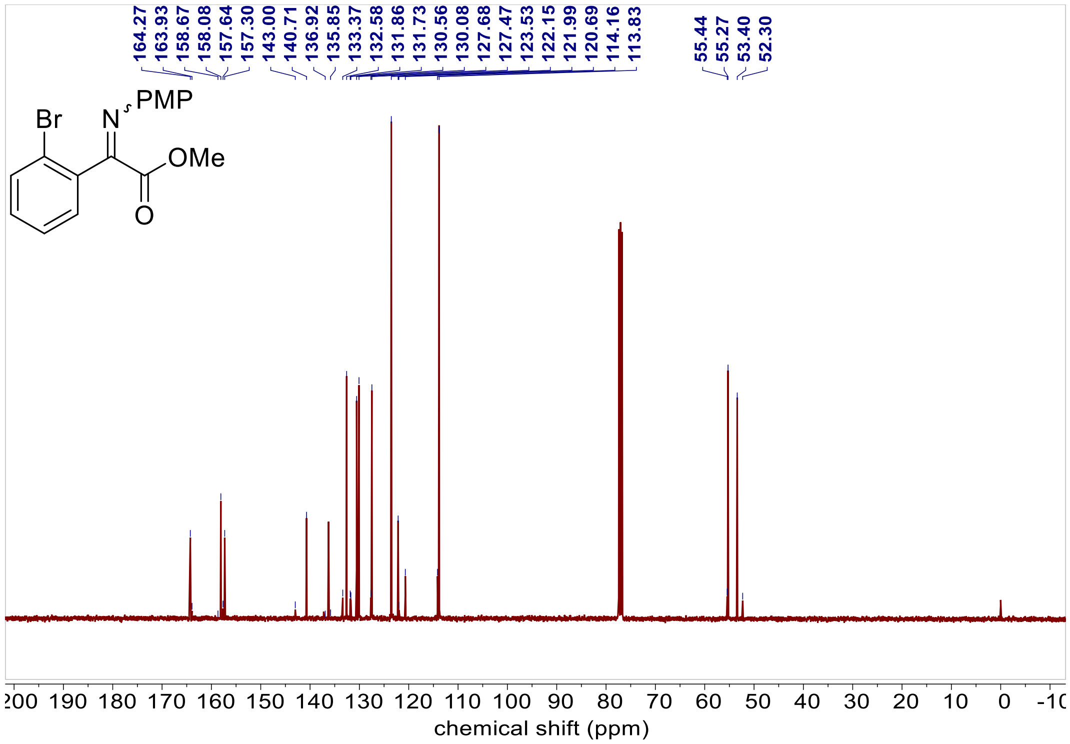

Supplementary Figure 18. ^{1}H NMR & ^{13}C NMR spectra of compound **1d** in $CDCl_3$

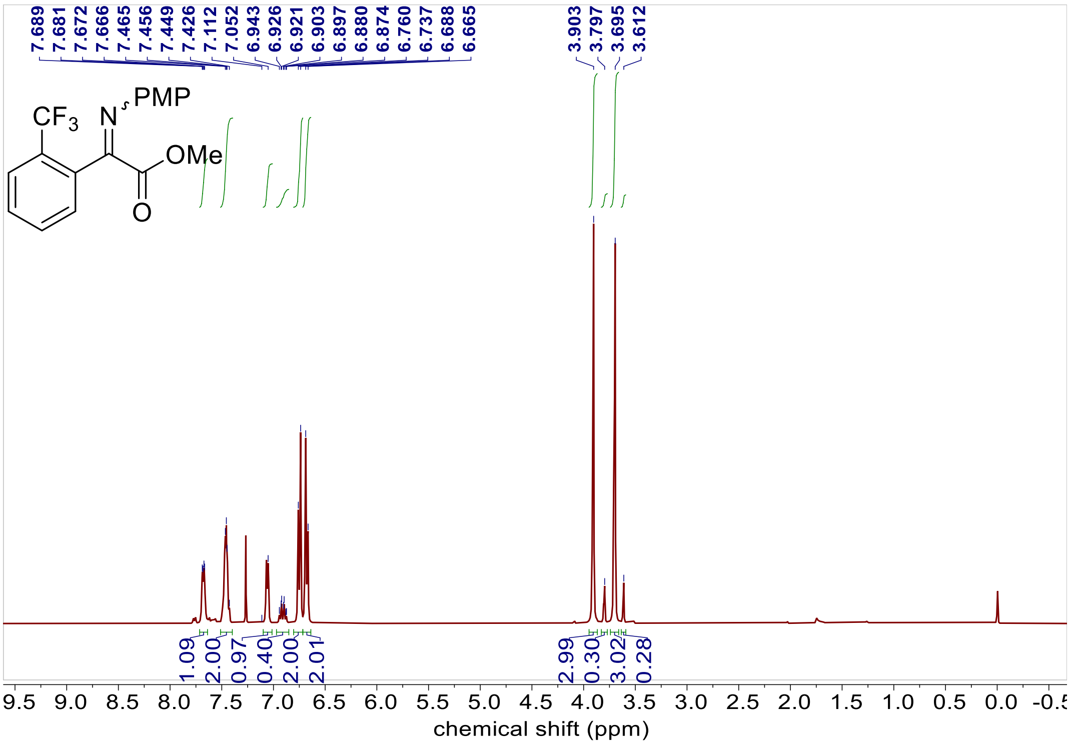
7.689
7.681
7.672
7.666
7.465
7.456
7.449
7.426
7.112
7.052
6.943
6.926
6.921
6.903
6.897
6.880
6.874
6.760
6.737
6.688
6.665
3.903
3.797
3.695
3.612
CF3
N
PMP
OMe
O
1.09
2.00
0.97
0.40
2.00
2.01
2.99
0.30
3.02
0.28
chemical shift (ppm)

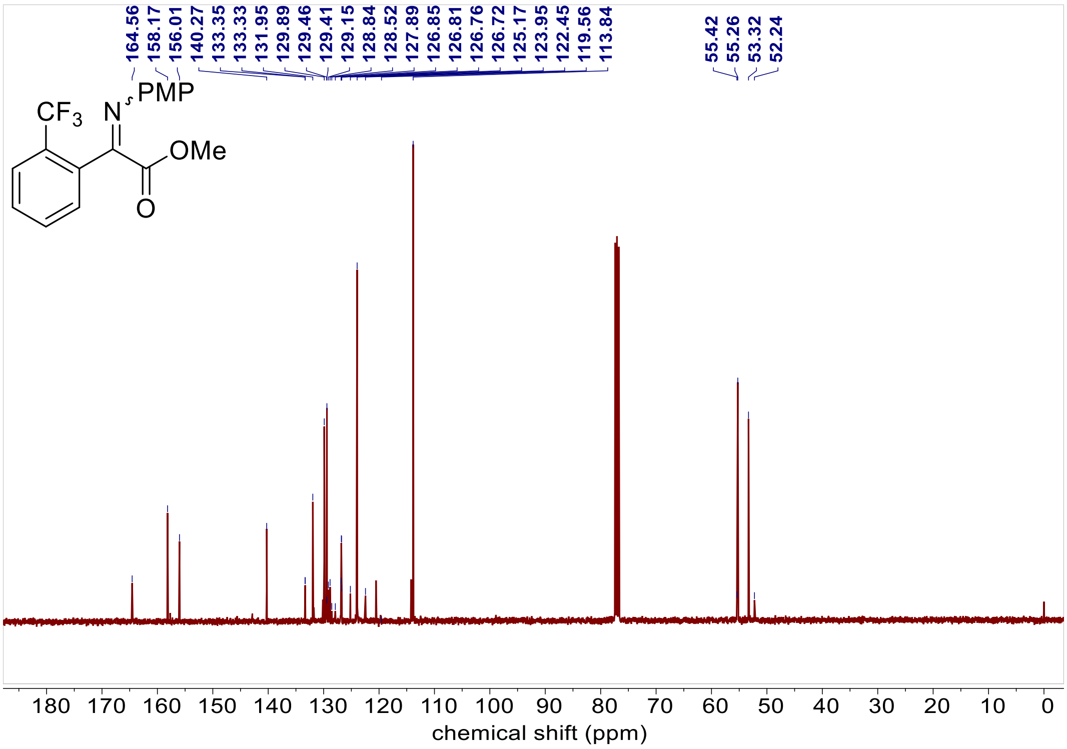
164.56
158.17
156.01
140.27
133.35
133.33
131.95
129.89
129.46
129.41
129.15
128.84
128.52
127.89
126.85
126.81
126.76
126.72
125.17
123.95
122.45
119.56
113.84
55.42
55.26
53.32
52.24
CF3
N
PMP
OMe
O
chemical shift (ppm)

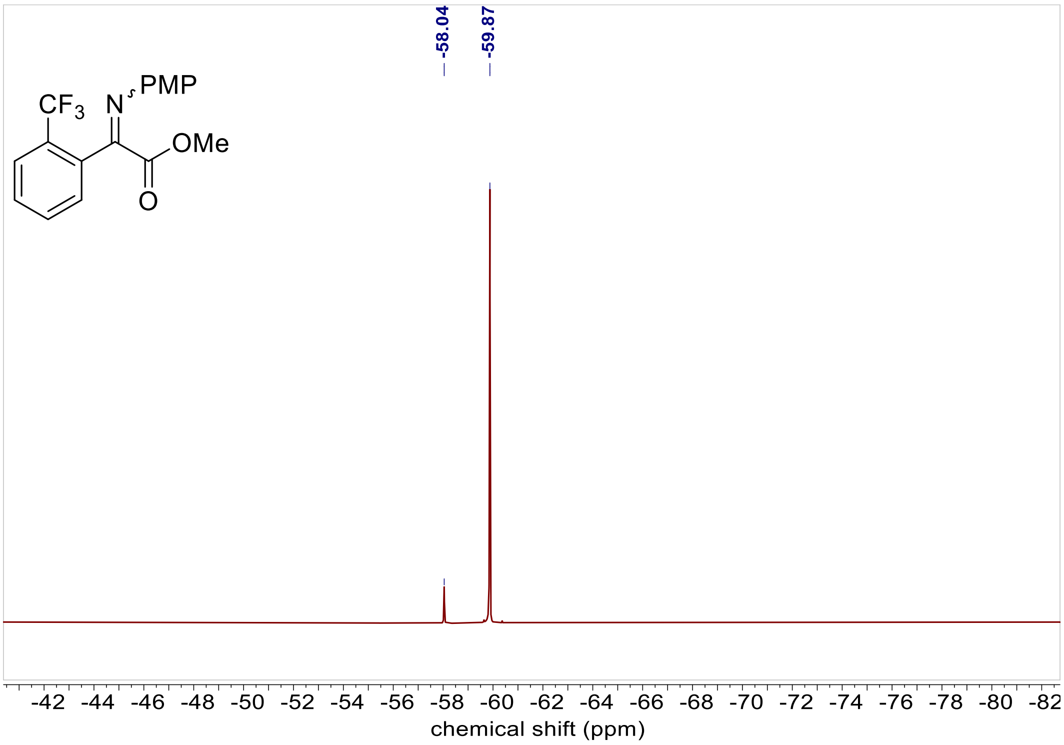

Supplementary Figure 19. ^{1}H NMR & ^{13}C NMR & ^{19}F NMR spectra of compound **1e** in $CDCl_3$

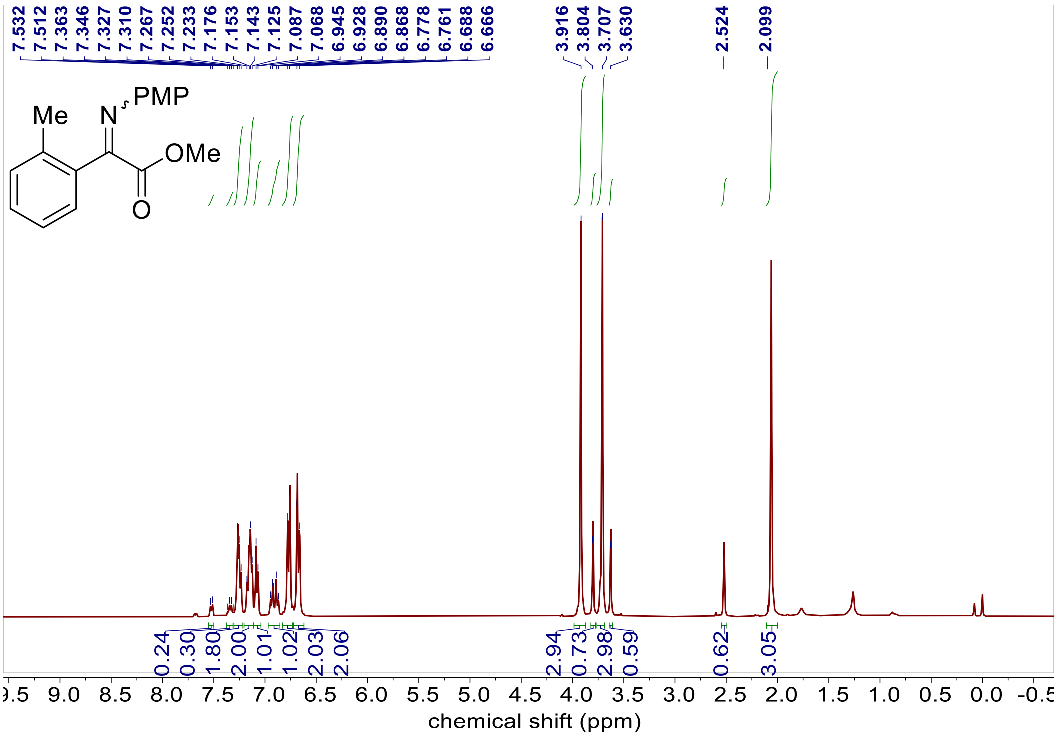

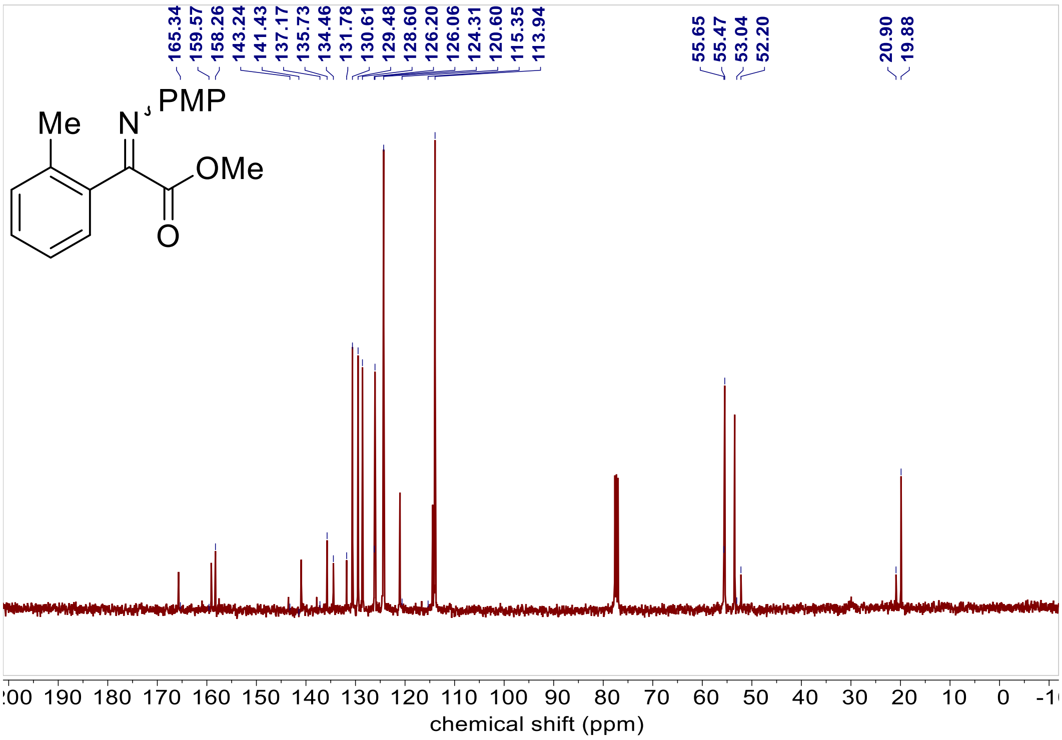

Supplementary Figure 20. ^{1}H NMR & ^{13}C NMR spectra of compound **1f** in $CDCl_3$

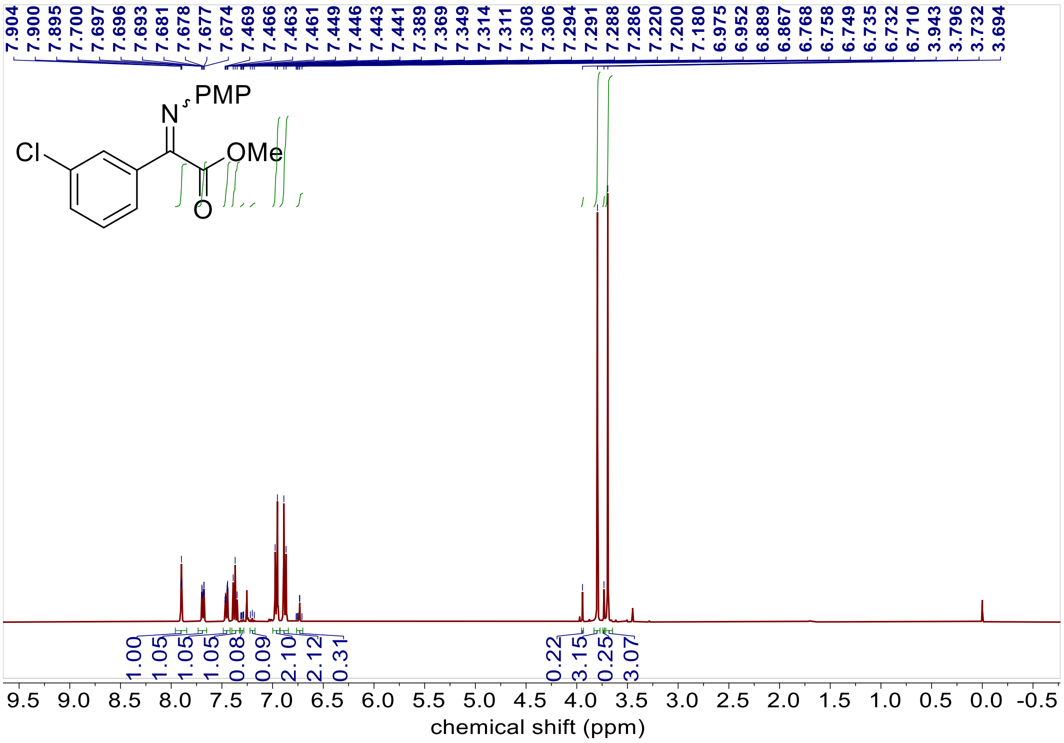

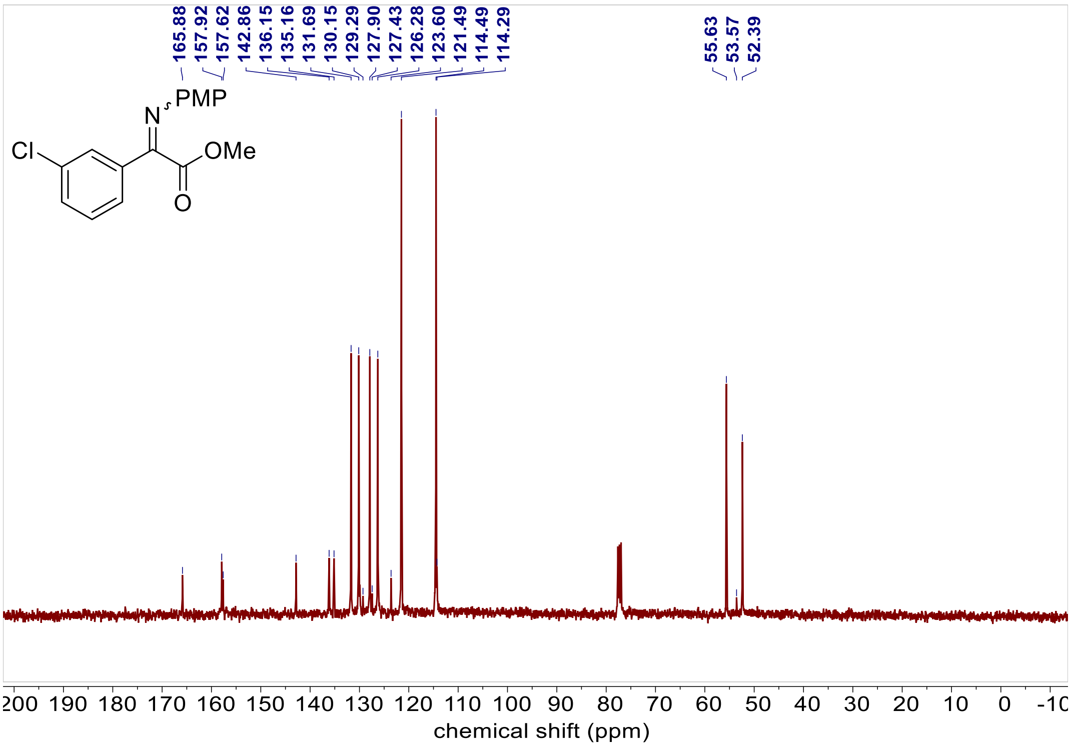

Supplementary Figure 21. ^{1}H NMR & ^{13}C NMR spectra of compound **1i** in $CDCl_3$

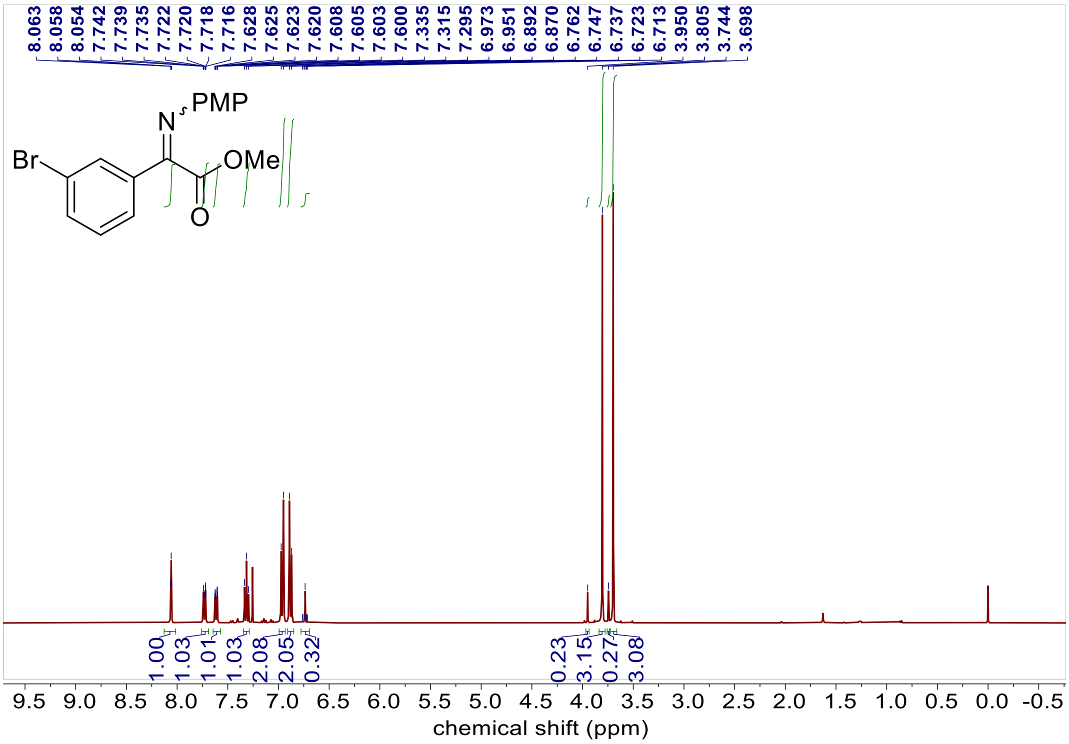

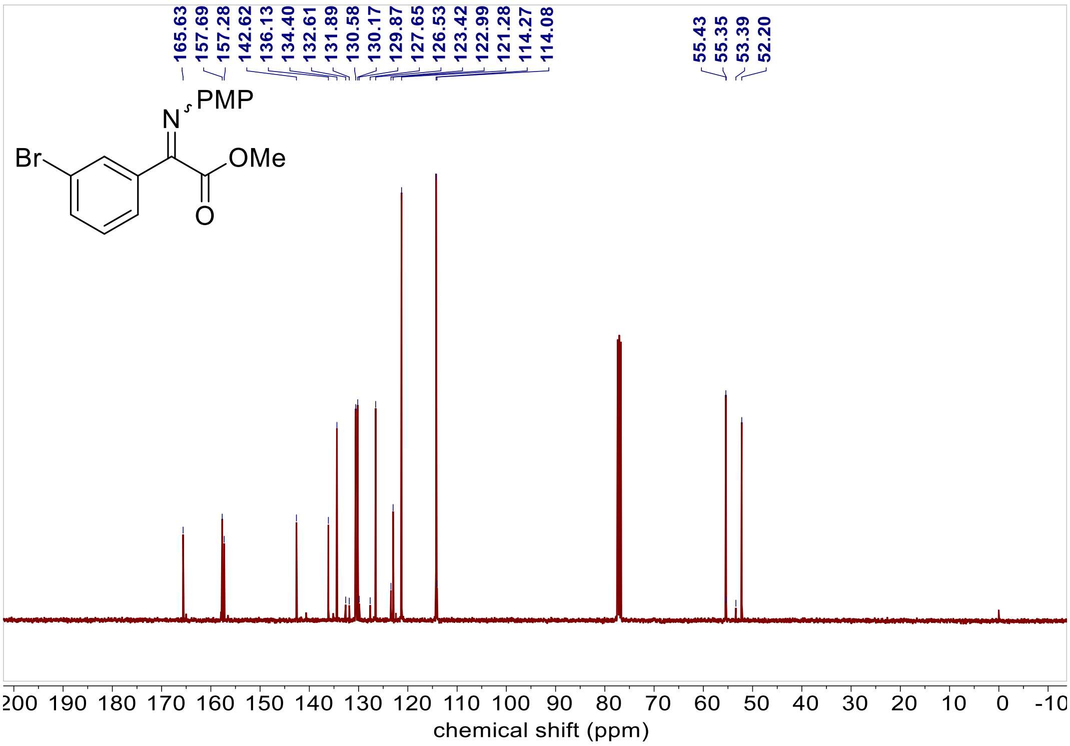

Supplementary Figure 22. ^{1}H NMR & ^{13}C NMR spectra of compound **1j** in $CDCl_3$

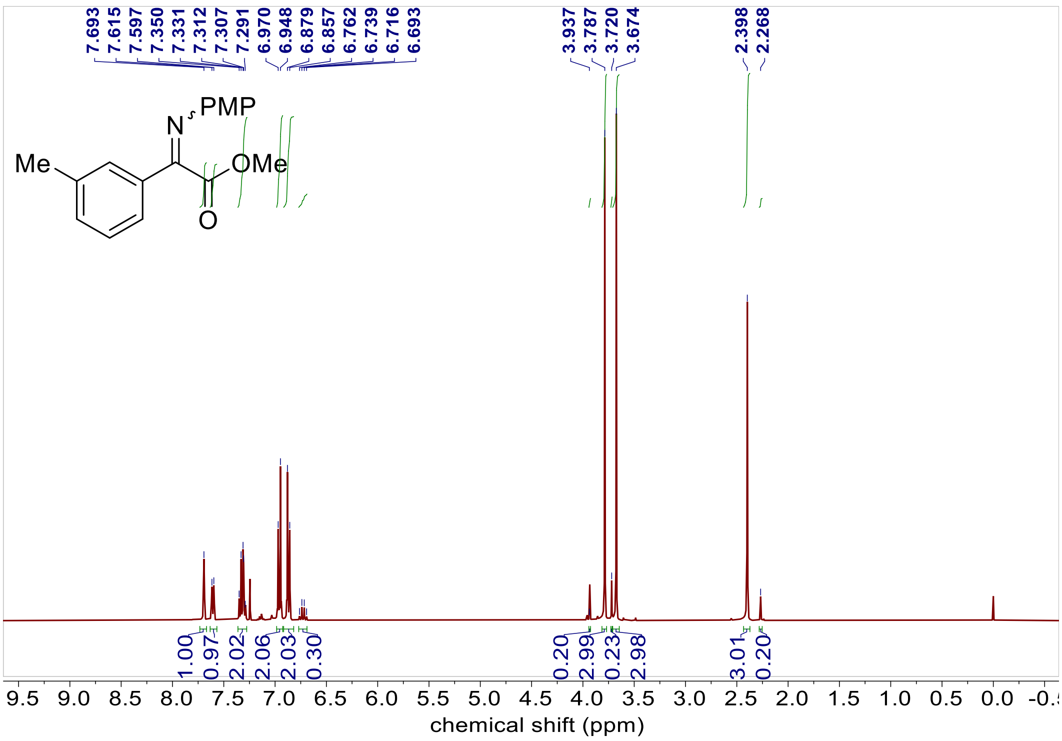

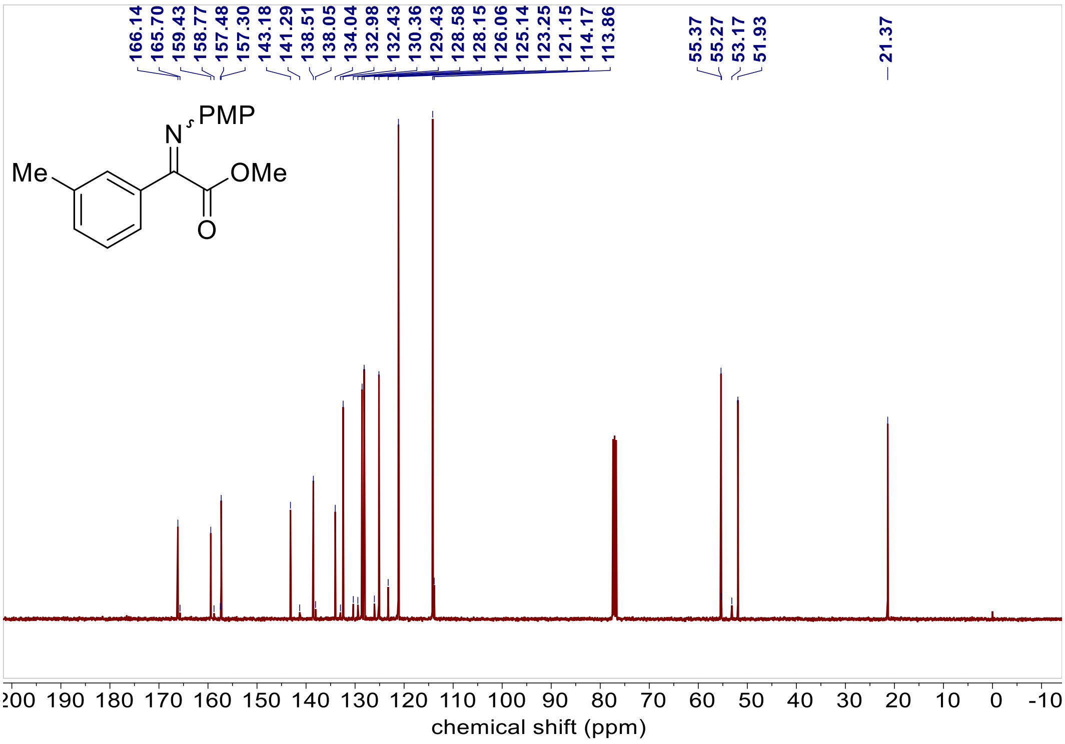

Supplementary Figure 23. ^{1}H NMR & ^{13}C NMR spectra of compound **1l** in $CDCl_3$

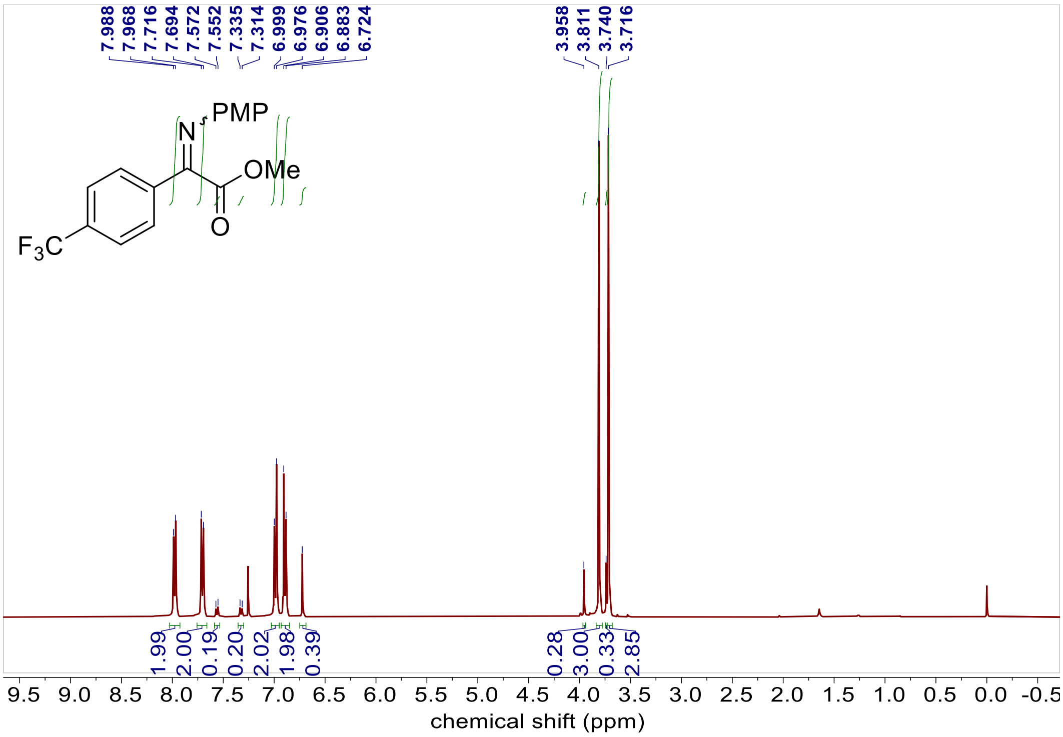
7.988
7.968
7.716
7.694
7.572
7.552
7.335
7.314
6.999
6.976
6.906
6.883
6.724
3.958
3.811
3.740
3.716
N
PMP
OMe
O
F3C
1.99
2.00
0.19
0.20
2.02
1.98
0.39
0.28
3.00
0.33
2.85
9.5 9.0 8.5 8.0 7.5 7.0 6.5 6.0 5.5 5.0 4.5 4.0 3.5 3.0 2.5 2.0 1.5 1.0 0.5 0.0 -0.5
chemical shift (ppm)

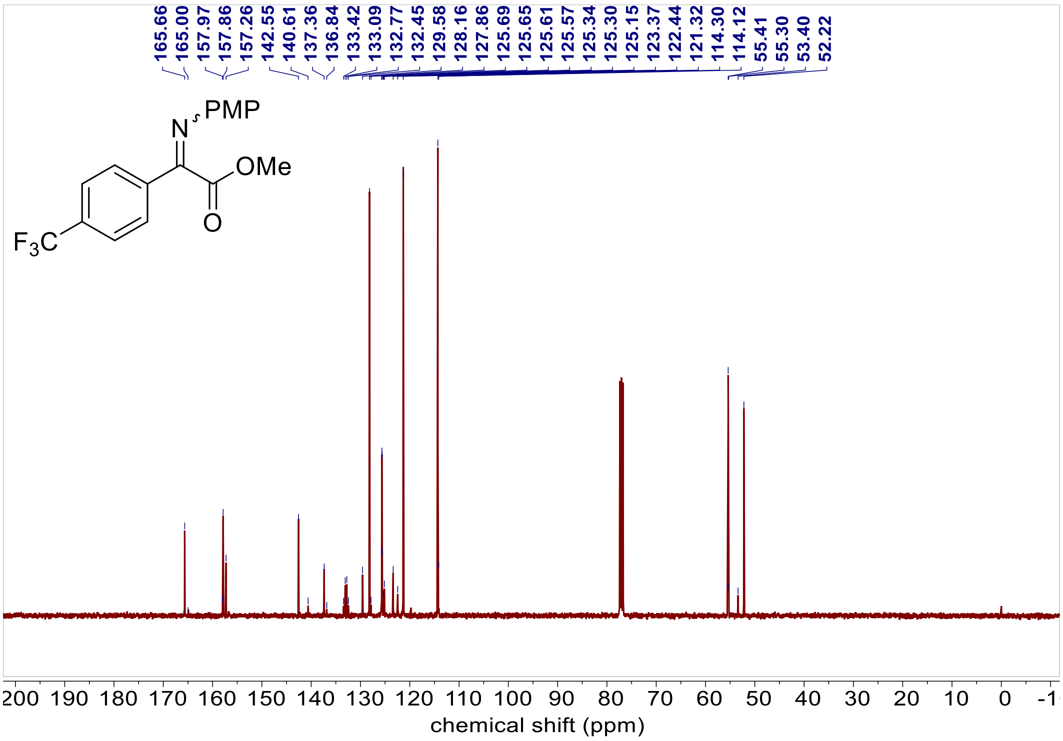
165.66
165.00
157.97
157.86
157.26
142.55
140.61
137.36
136.84
133.42
133.09
132.77
132.45
129.58
128.16
127.86
125.69
125.65
125.61
125.57
125.34
125.30
125.15
123.37
122.44
121.32
114.30
114.12
55.41
55.30
53.40
52.22
N
PMP
OMe
O
F3C
200 190 180 170 160 150 140 130 120 110 100 90 80 70 60 50 40 30 20 10 0 -1
chemical shift (ppm)

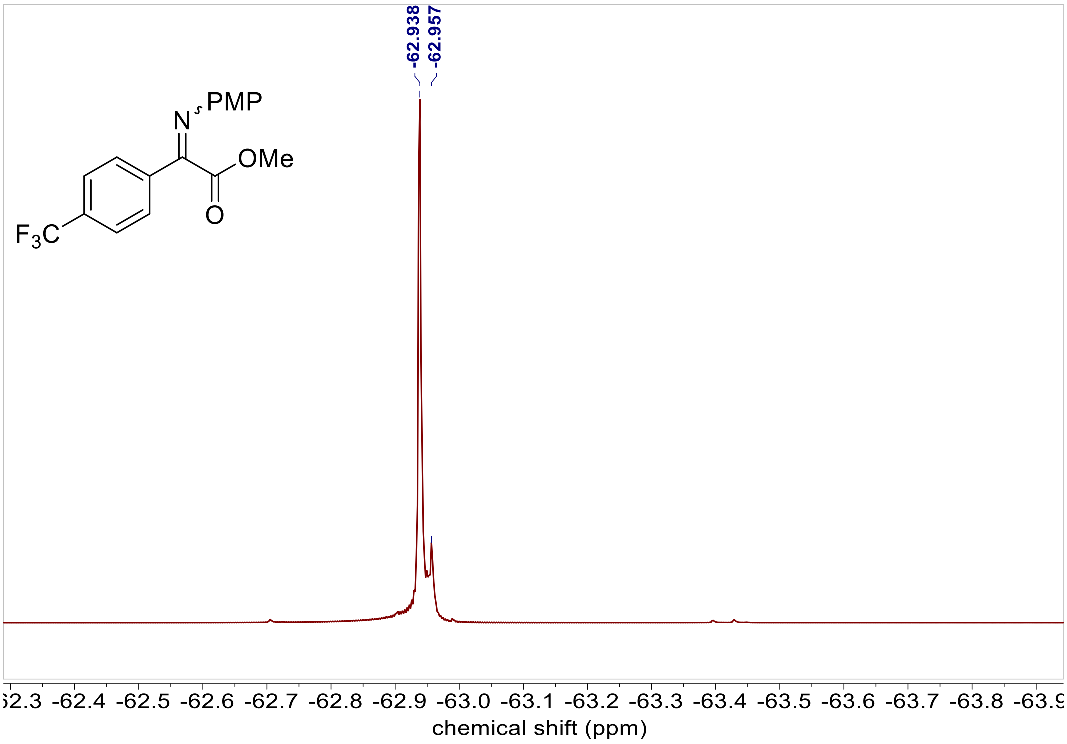

Supplementary Figure 24. ^{1}H NMR & ^{13}C NMR & ^{19}F NMR spectra of compound **1q** in $CDCl_3$

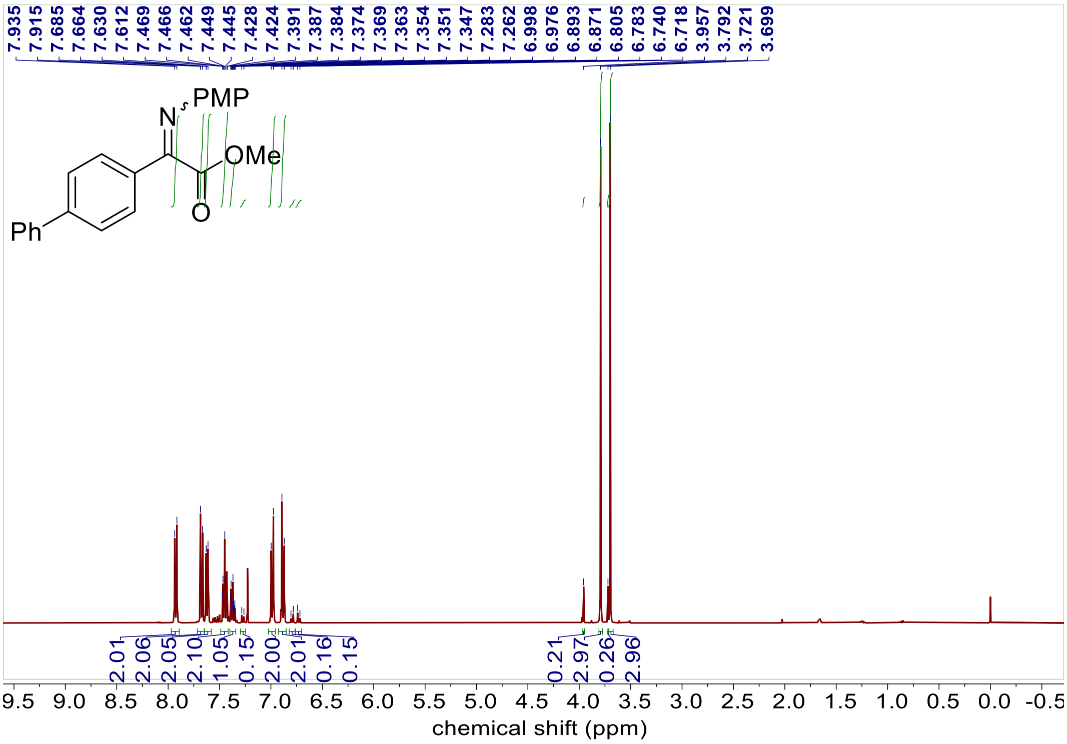

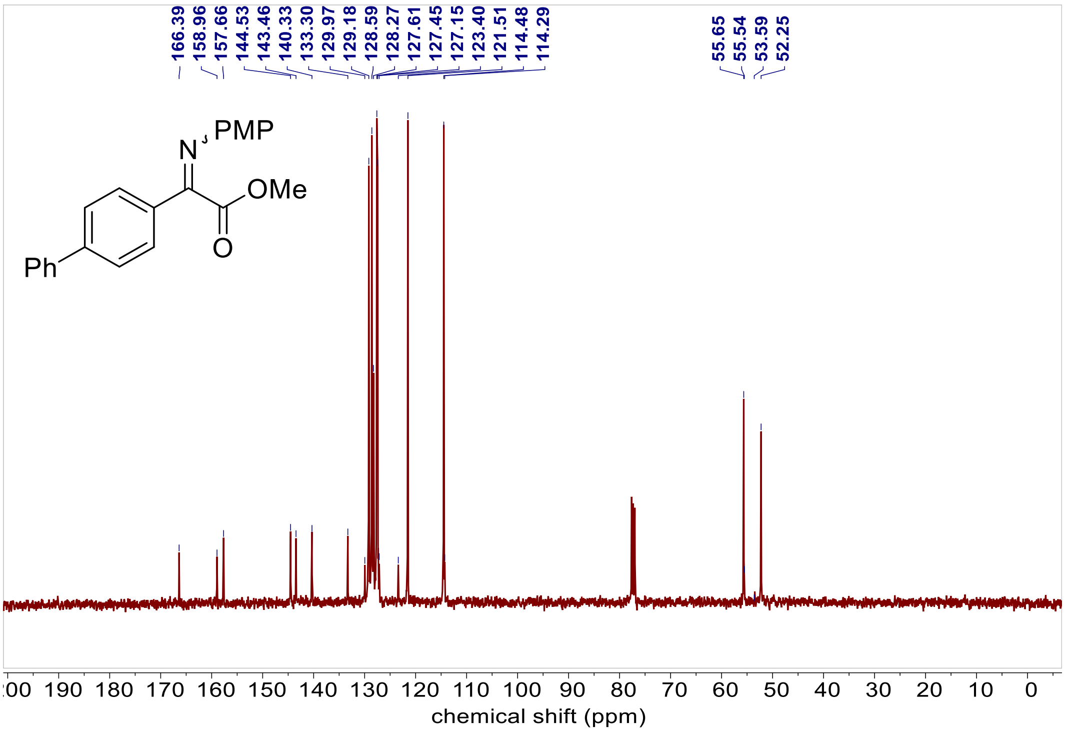

Supplementary Figure 25. ^{1}H NMR & ^{13}C NMR spectra of compound **1t** in $CDCl_3$

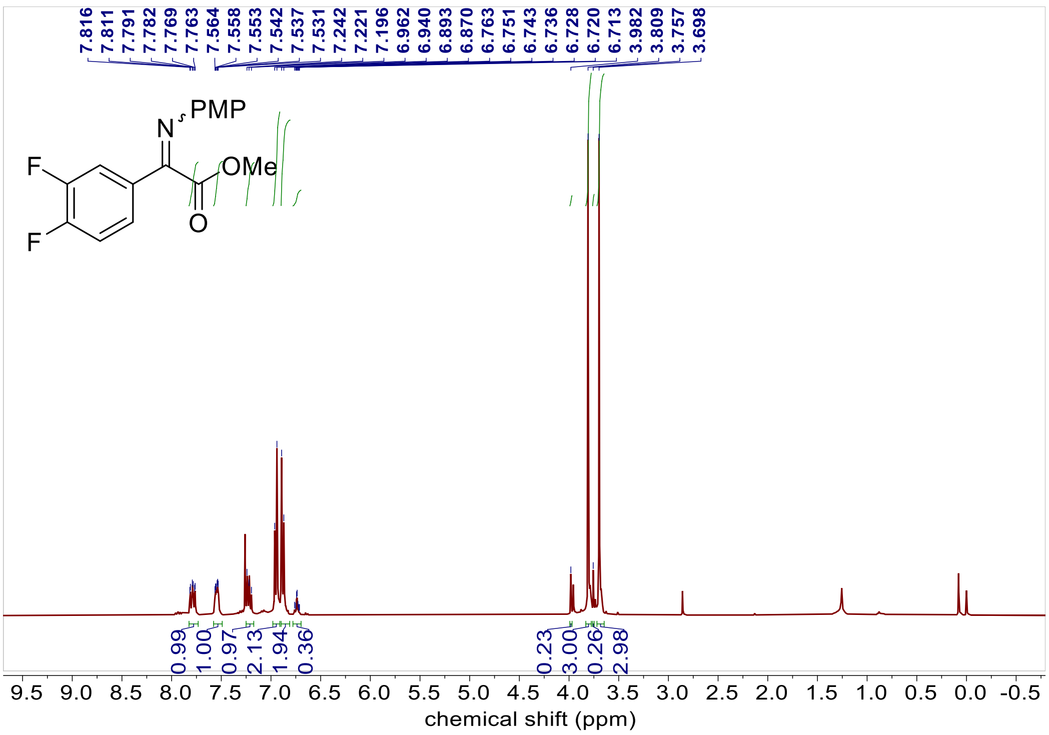
7.816
7.811
7.791
7.782
7.769
7.763
7.564
7.558
7.553
7.542
7.537
7.531
7.242
7.221
7.196
6.962
6.940
6.893
6.870
6.763
6.751
6.743
6.736
6.728
6.720
6.713
3.982
3.809
3.757
3.698
PMP
N
F
OMe
F
O
0.99
1.00
0.97
2.13
1.94
0.36
0.23
3.00
0.26
2.98
9.5 9.0 8.5 8.0 7.5 7.0 6.5 6.0 5.5 5.0 4.5 4.0 3.5 3.0 2.5 2.0 1.5 1.0 0.5 0.0 -0.5
chemical shift (ppm)

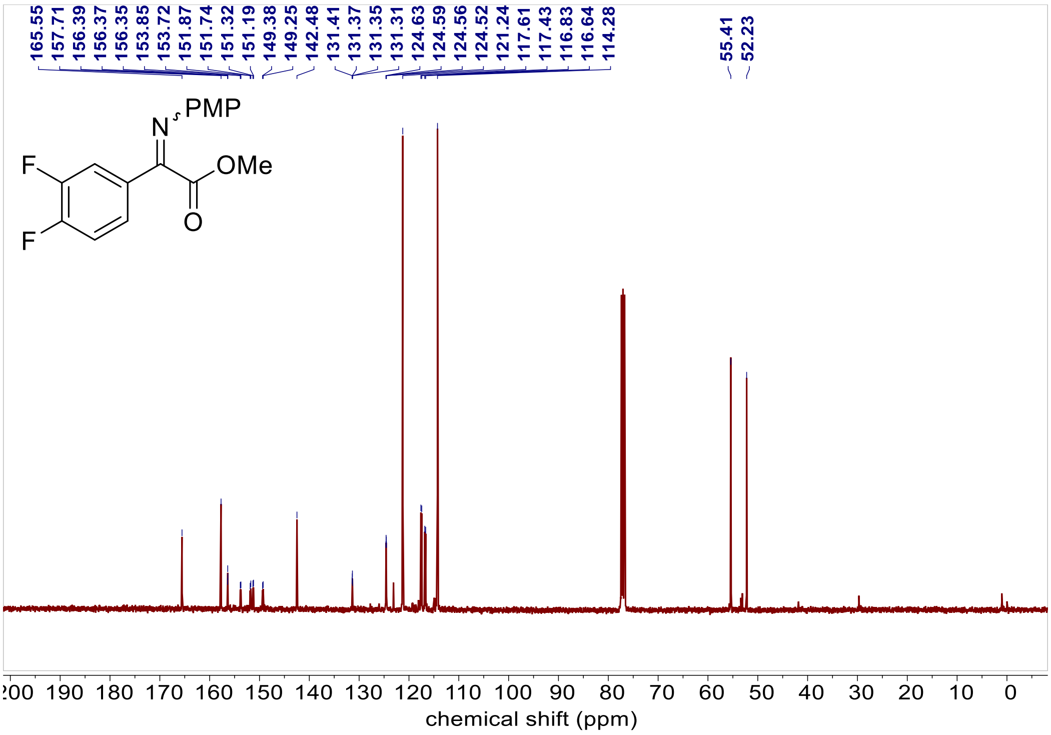
165.55
157.71
156.39
156.37
156.35
153.85
153.72
151.87
151.74
151.32
151.19
149.38
149.25
142.48
131.41
131.37
131.35
131.31
124.63
124.59
124.56
124.52
121.24
117.61
117.43
116.83
116.64
114.28
55.41
52.23
PMP
N
F
OMe
F
O
200 190 180 170 160 150 140 130 120 110 100 90 80 70 60 50 40 30 20 10 0
chemical shift (ppm)

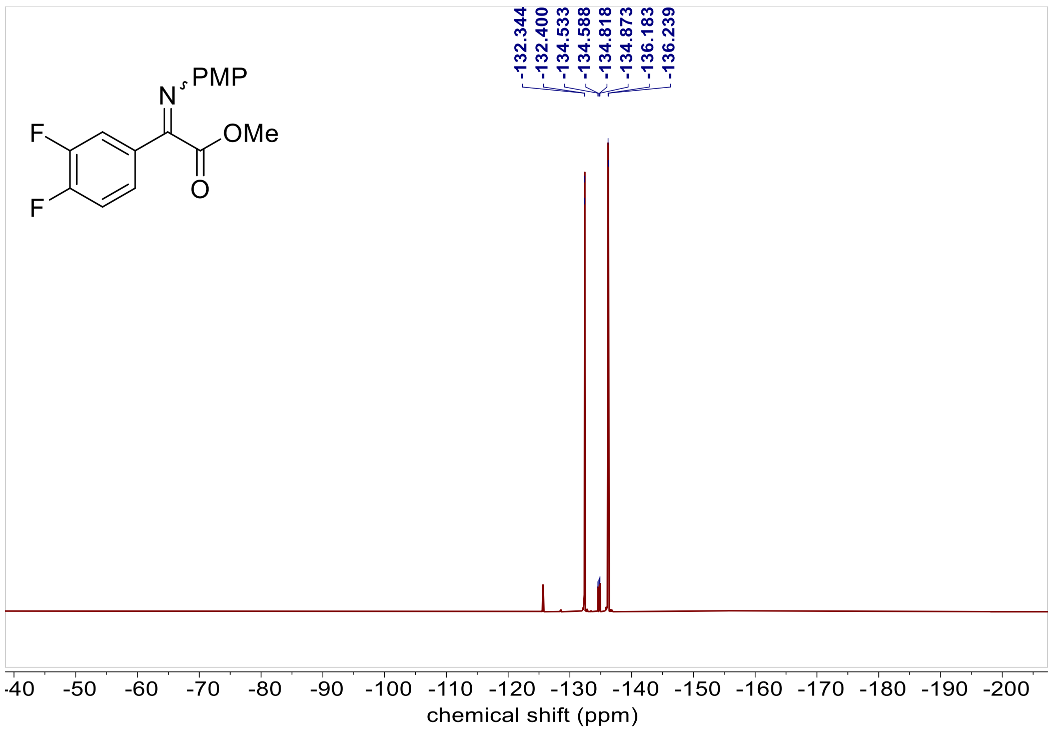

Supplementary Figure 26. ^{1}H NMR & ^{13}C NMR & ^{19}F NMR spectra of compound **1u** in $CDCl_3$

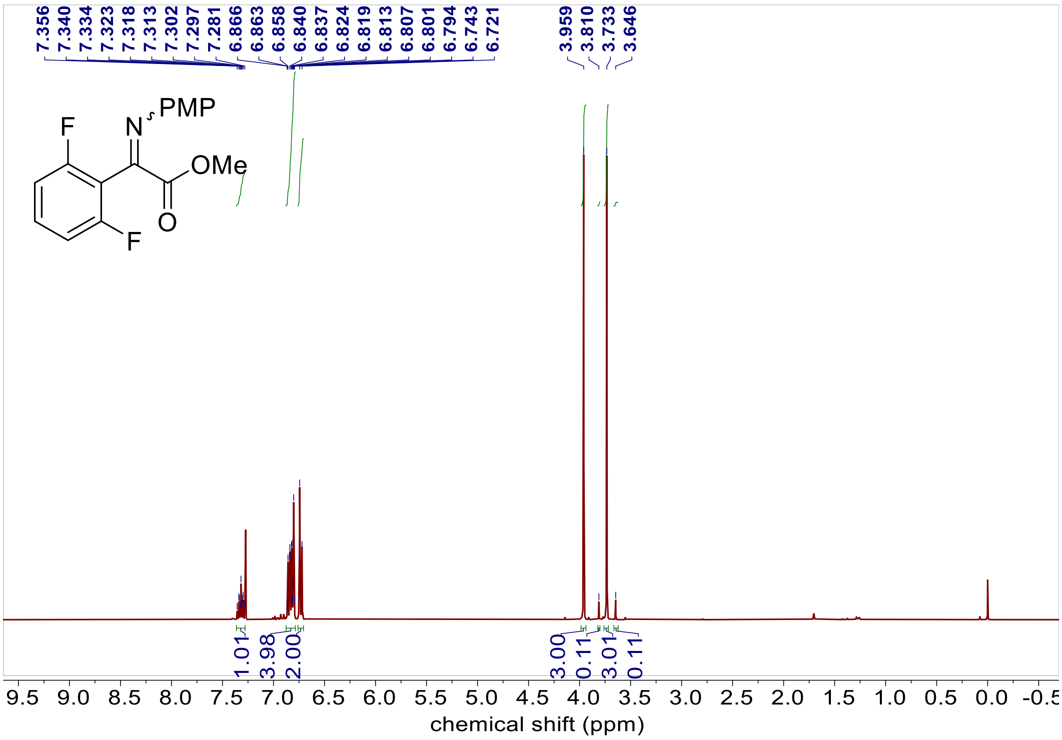
7.356
7.340
7.334
7.323
7.318
7.313
7.302
7.297
7.281
6.866
6.863
6.858
6.840
6.837
6.824
6.819
6.813
6.807
6.801
6.794
6.743
6.721
3.959
3.810
3.733
3.646
F
N
PMP
OMe
O
F
1.01
3.98
2.00
3.00
0.11
3.01
0.11
9.5 9.0 8.5 8.0 7.5 7.0 6.5 6.0 5.5 5.0 4.5 4.0 3.5 3.0 2.5 2.0 1.5 1.0 0.5 0.0 -0.5
chemical shift (ppm)

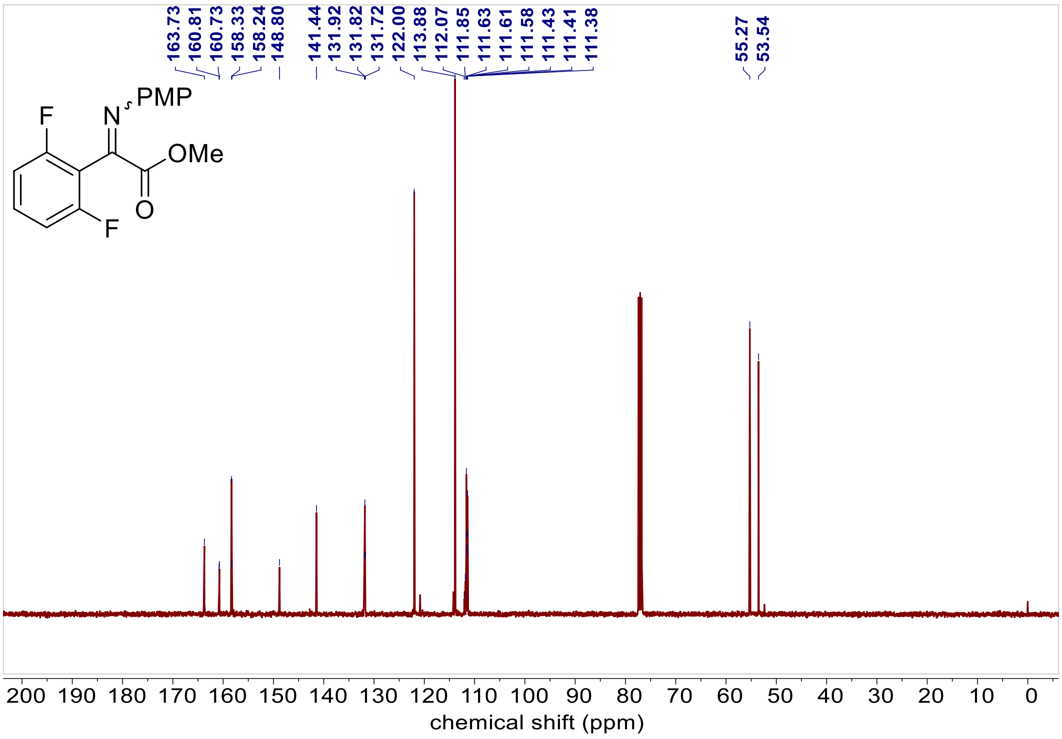
163.73
160.81
160.73
158.33
158.24
148.80
141.44
131.92
131.82
131.72
122.00
113.88
112.07
111.85
111.63
111.61
111.58
111.43
111.41
111.38
55.27
53.54
F
N
PMP
OMe
O
F
200 190 180 170 160 150 140 130 120 110 100 90 80 70 60 50 40 30 20 10 0
chemical shift (ppm)

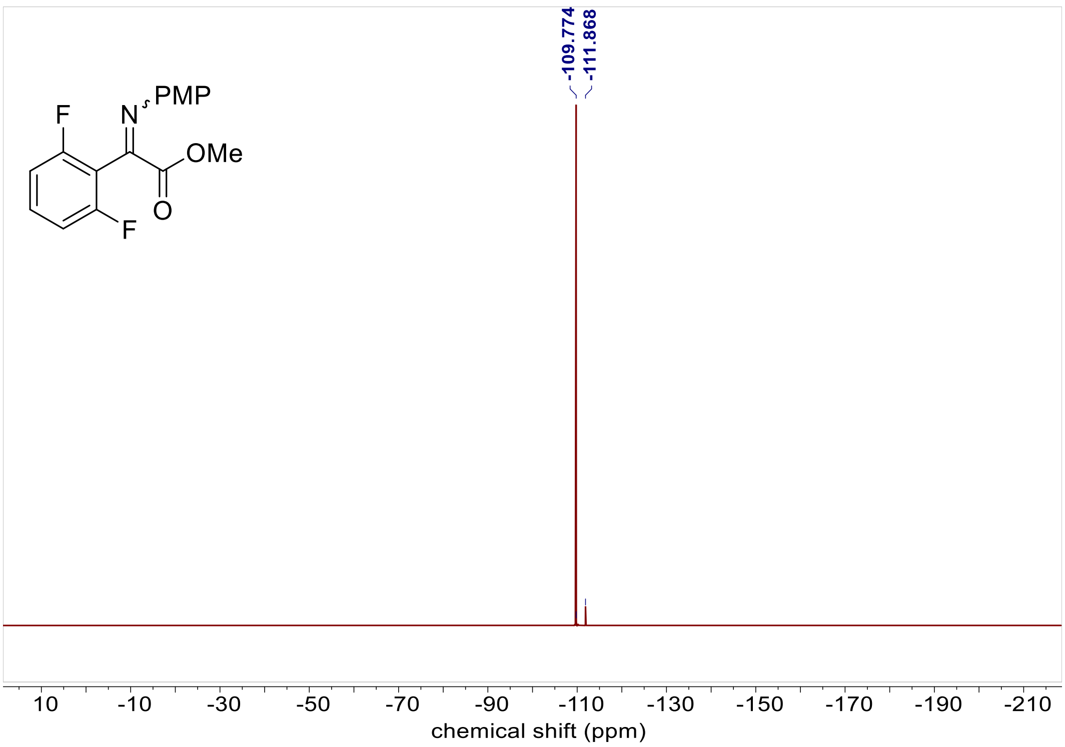

Supplementary Figure 27. ^{1}H NMR & ^{13}C NMR & ^{19}F NMR spectra of compound **1v** in $CDCl_3$

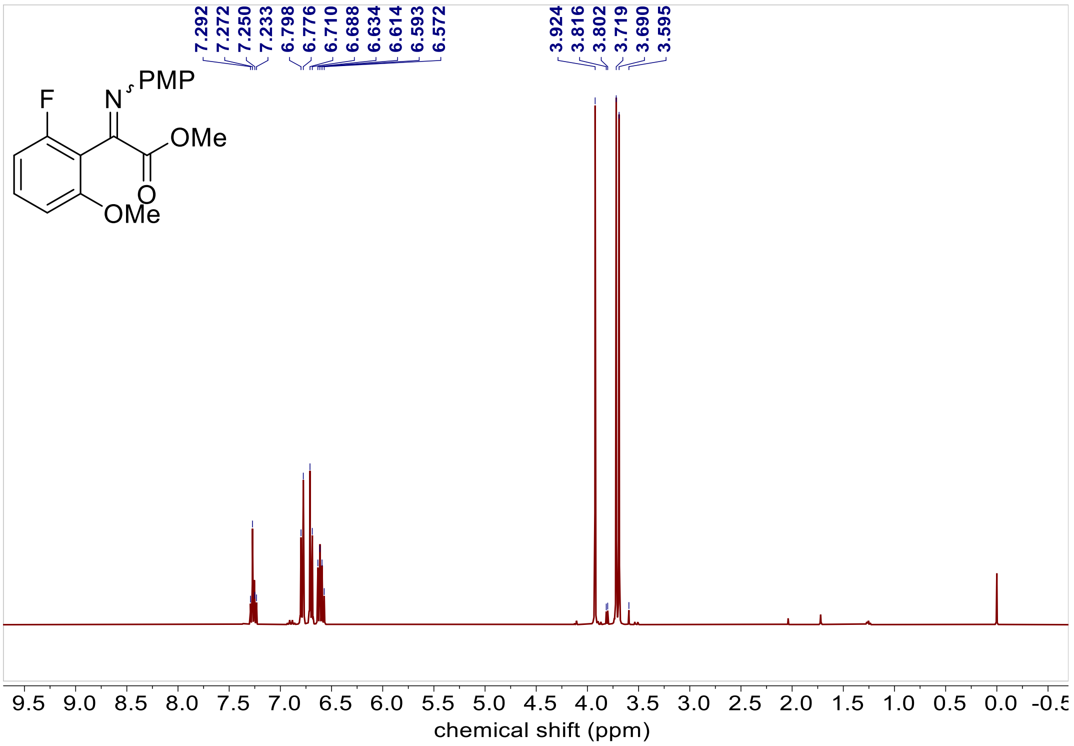
7.292
7.272
7.250
7.233
6.798
6.776
6.710
6.688
6.634
6.614
6.593
6.572
3.924
3.816
3.802
3.719
3.690
3.595
F
N
PMP
OMe
O
OMe
9.5 9.0 8.5 8.0 7.5 7.0 6.5 6.0 5.5 5.0 4.5 4.0 3.5 3.0 2.5 2.0 1.5 1.0 0.5 0.0 -0.5
chemical shift (ppm)

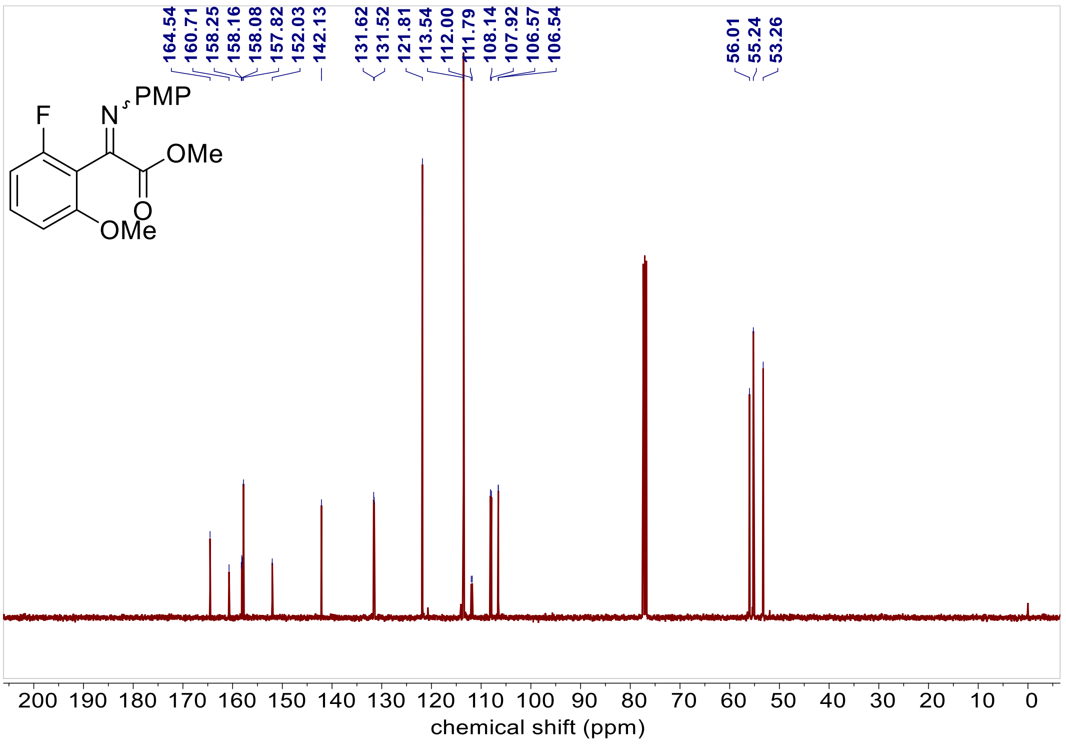
164.54
160.71
158.25
158.16
158.08
157.82
152.03
142.13
131.62
131.52
121.81
113.54
112.00
111.79
108.14
107.92
106.57
106.54
56.01
55.24
53.26
F
N
PMP
OMe
O
OMe
200 190 180 170 160 150 140 130 120 110 100 90 80 70 60 50 40 30 20 10 0
chemical shift (ppm)

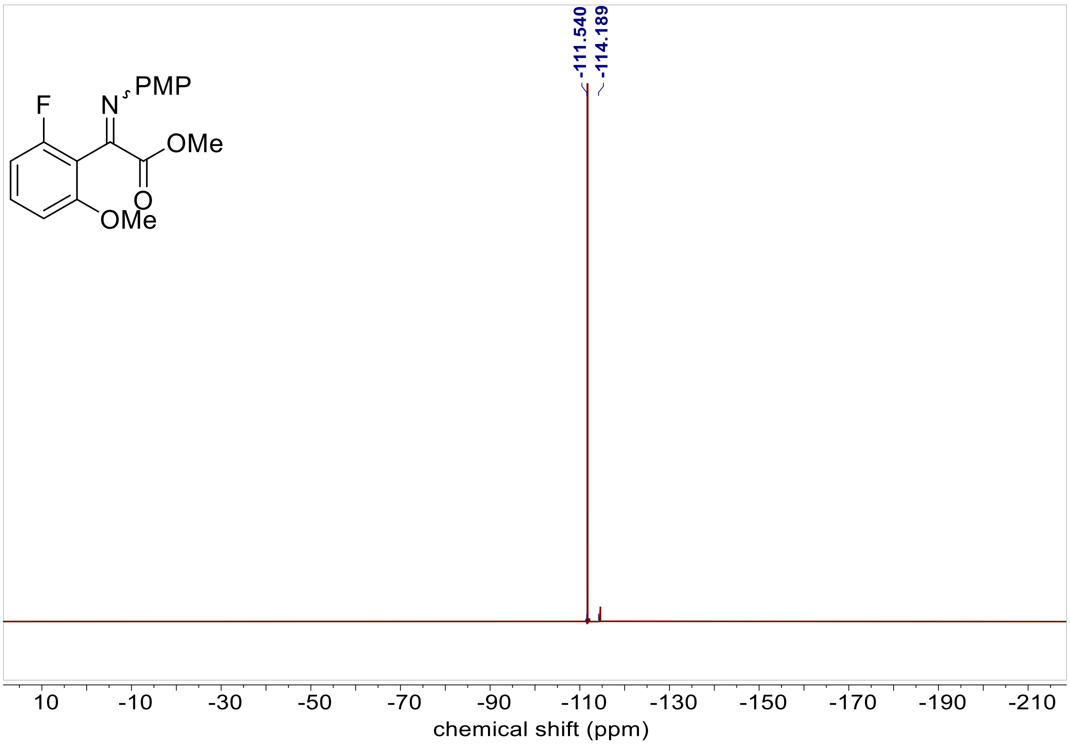

Supplementary Figure 28. ^{1}H NMR & ^{13}C NMR & ^{19}F NMR spectra of compound **1w** in $CDCl_3$

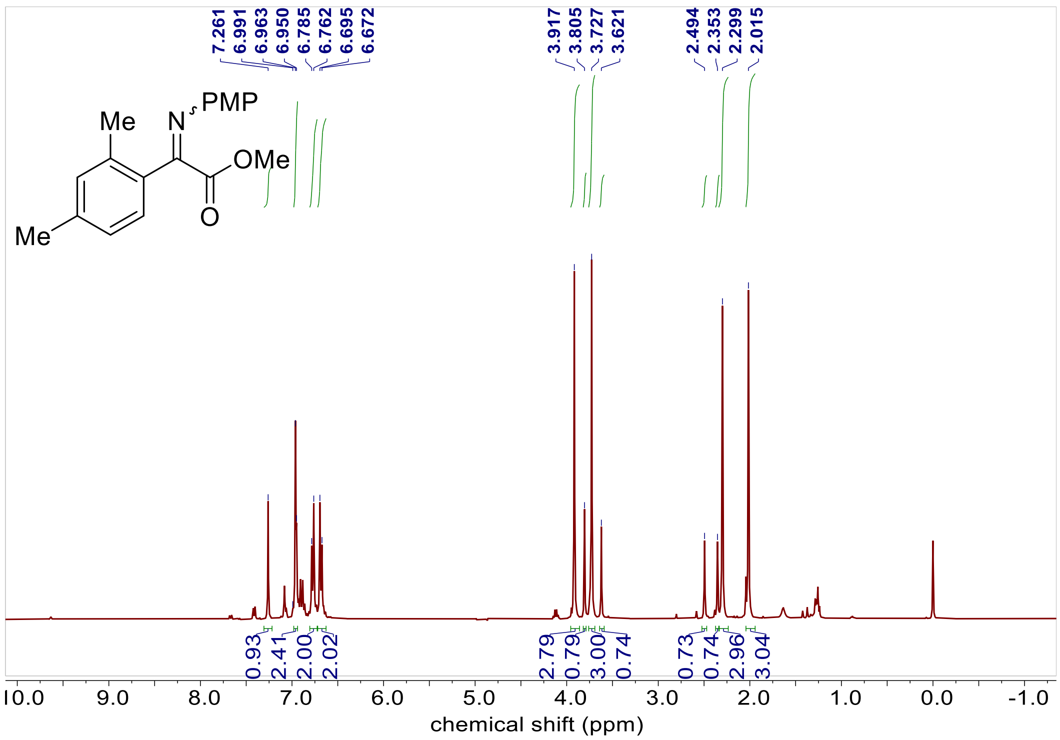

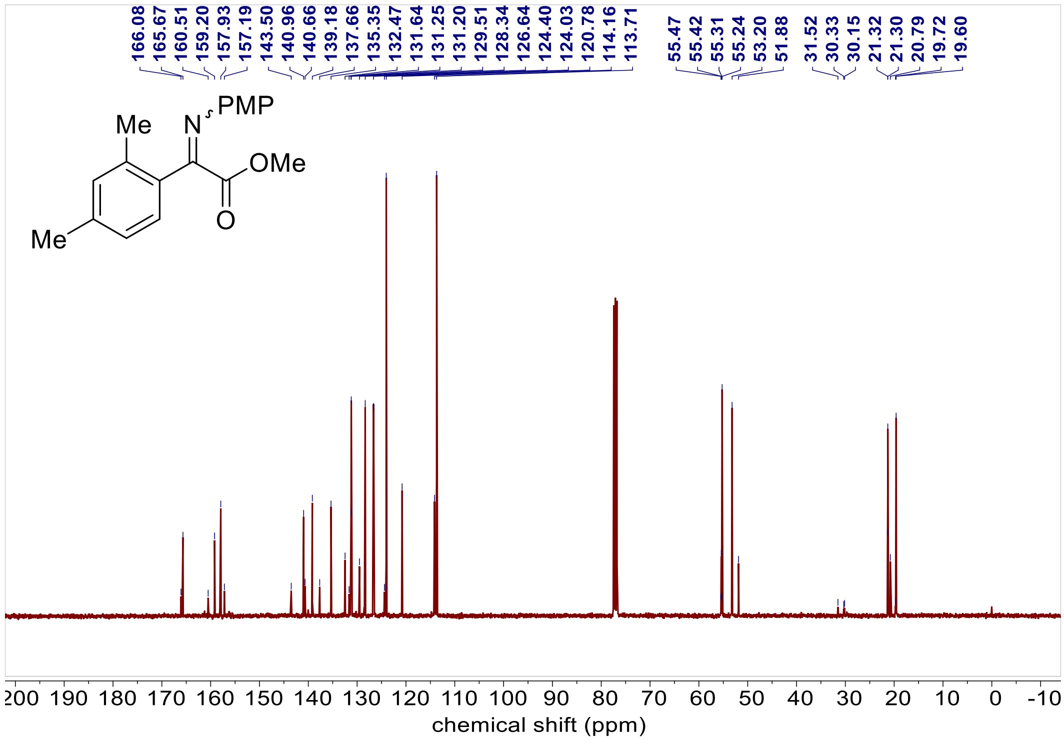

Supplementary Figure 29. ^{1}H NMR & ^{13}C NMR spectra of compound **1x** in $CDCl_3$

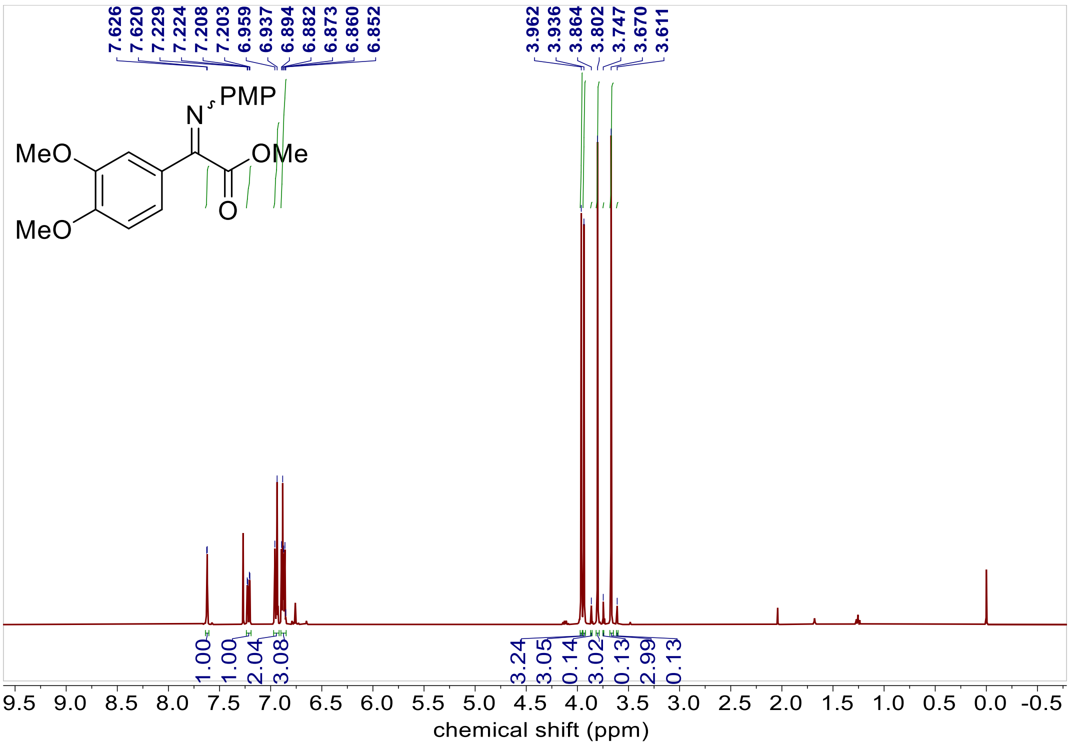

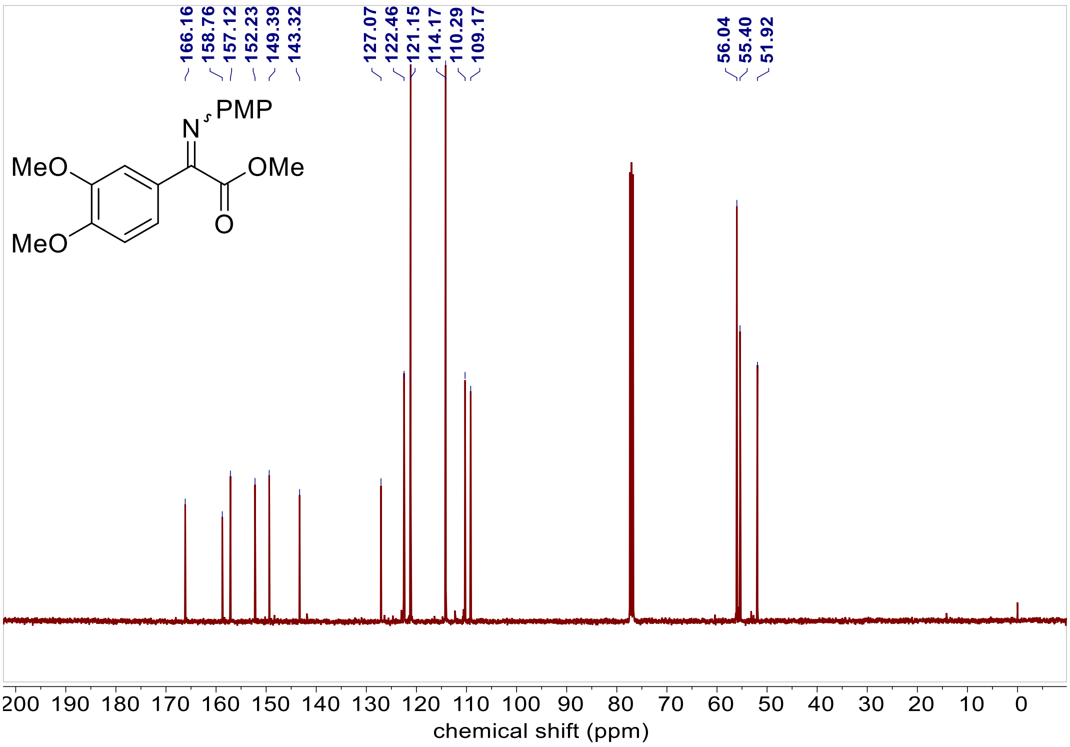

Supplementary Figure 30. ^{1}H NMR & ^{13}C NMR spectra of compound **1y** in $CDCl_3$

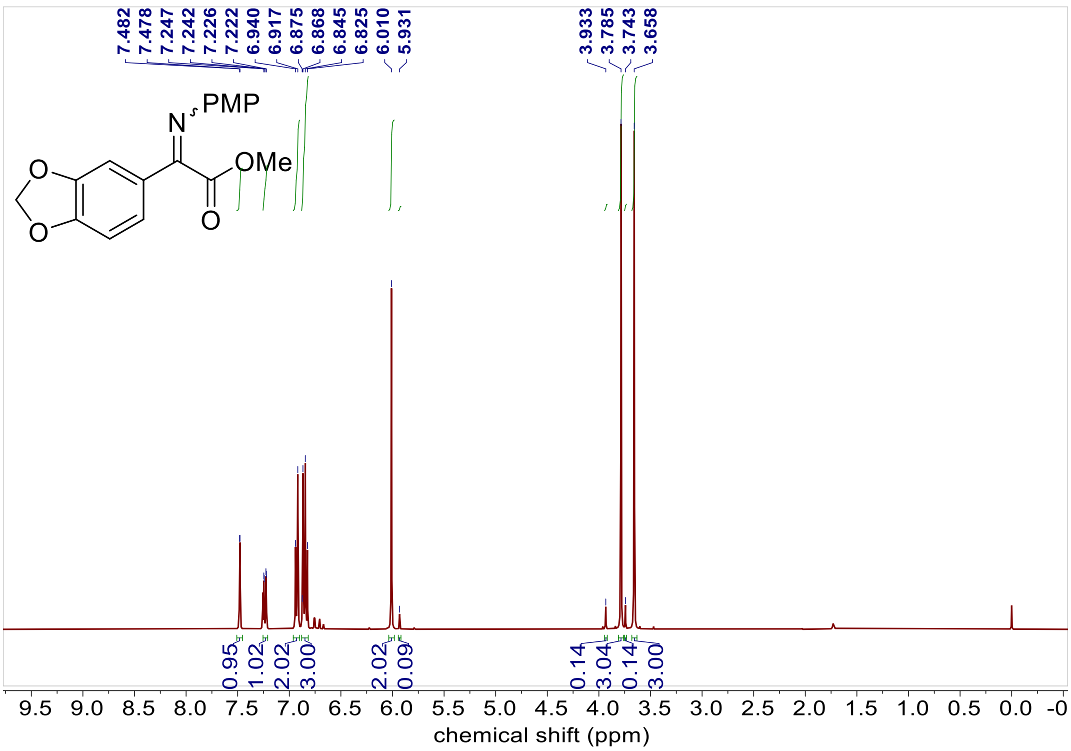

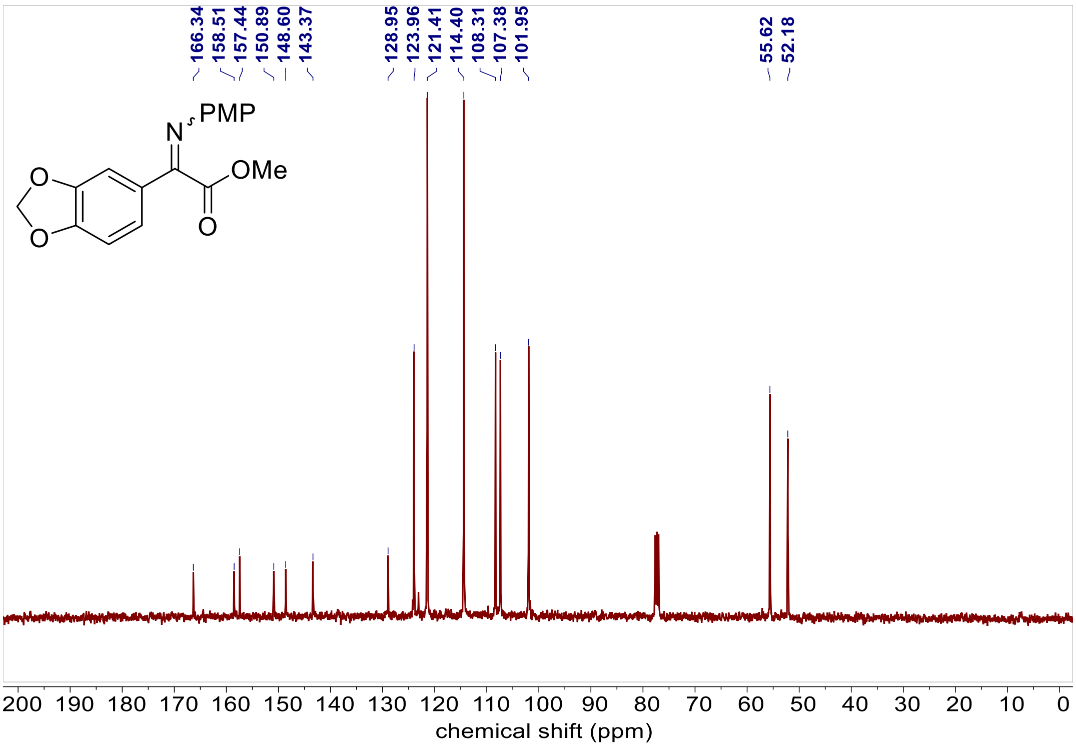

Supplementary Figure 31. ^{1}H NMR & ^{13}C NMR spectra of compound **1z** in $CDCl_3$

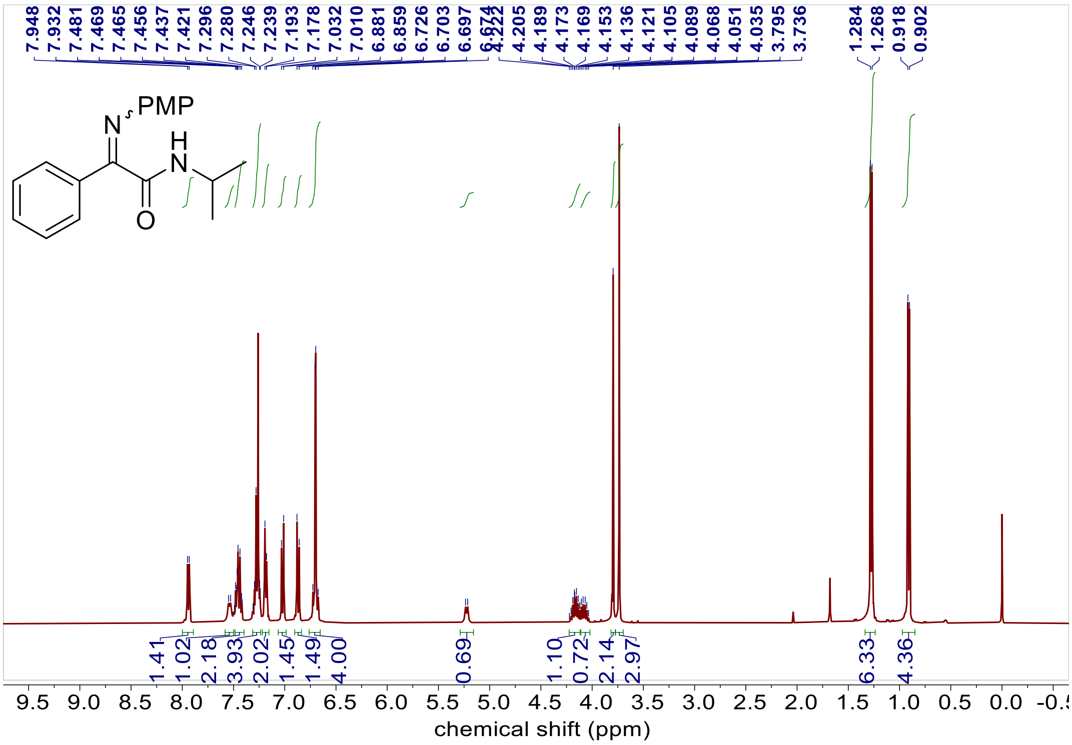

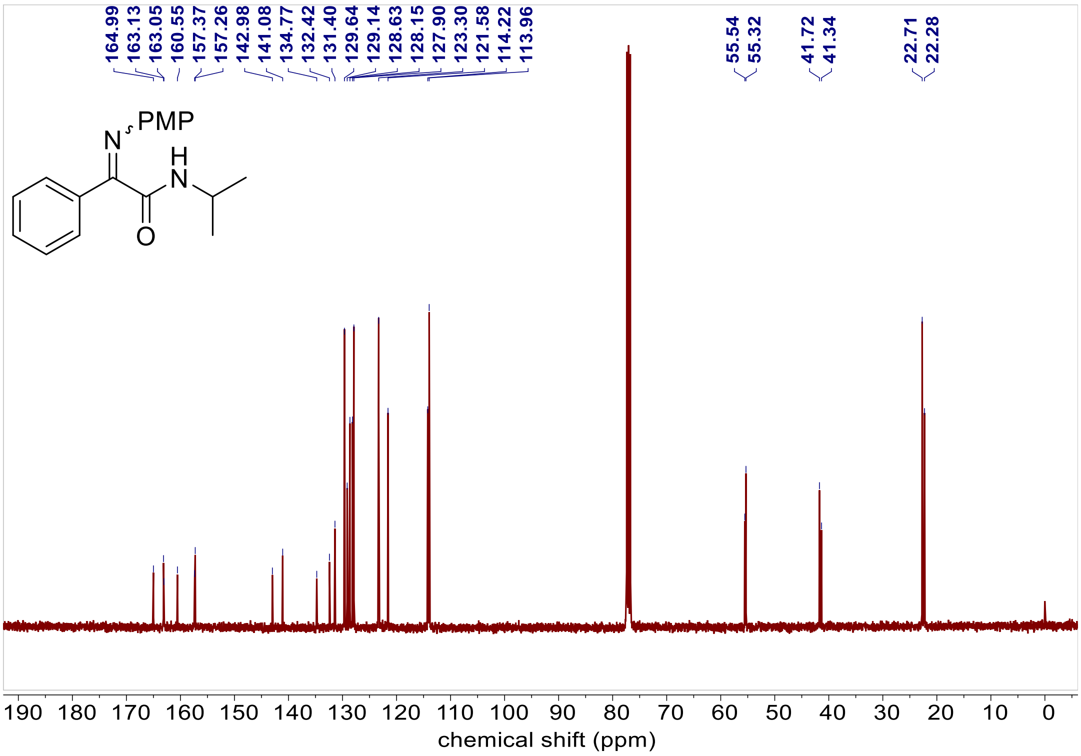

Supplementary Figure 32. ^{1}H NMR & ^{13}C NMR & spectra of compound **1af** in $CDCl_3$

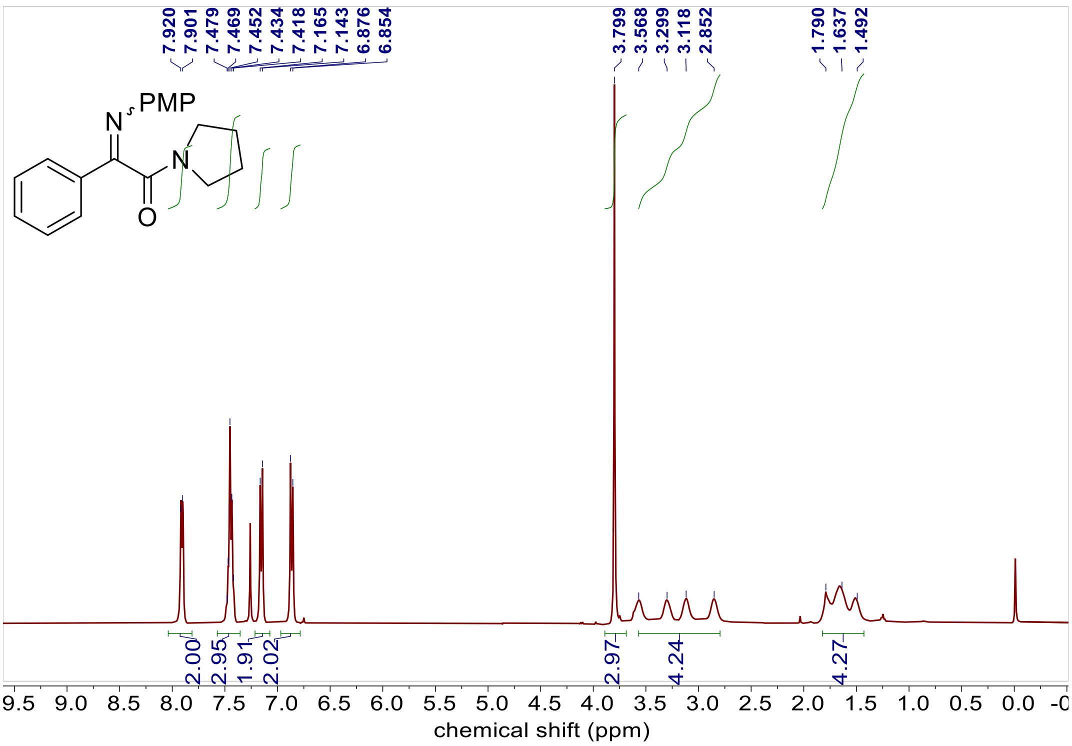

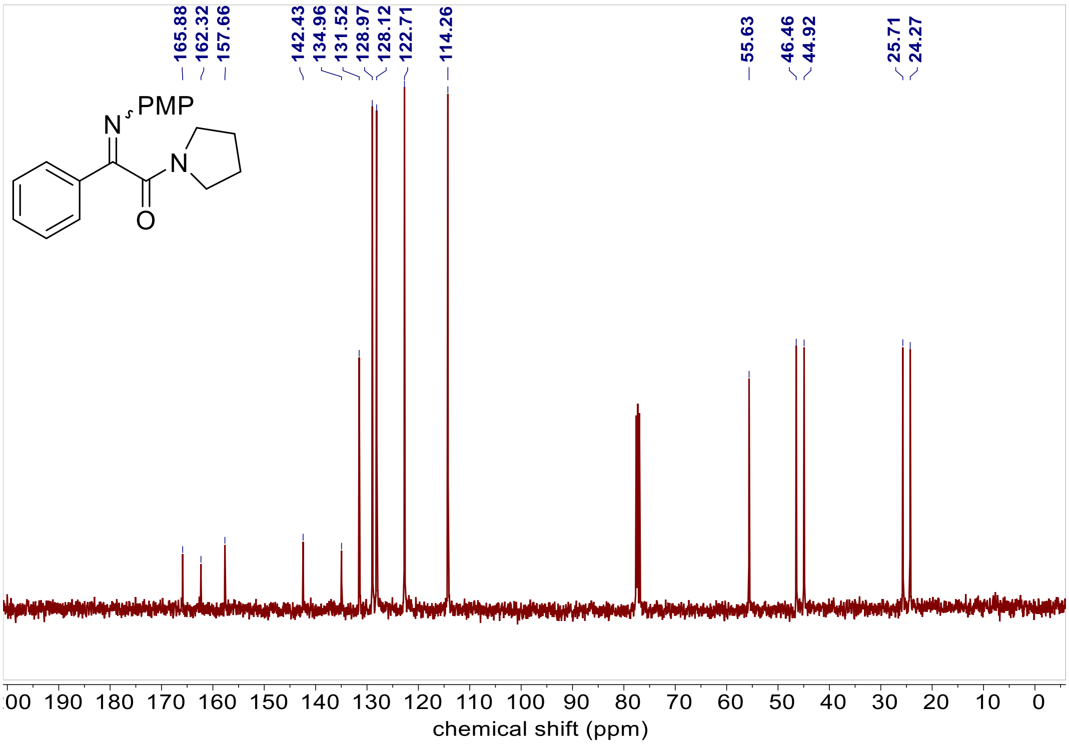

Supplementary Figure 33. ^{1}H NMR & ^{13}C NMR spectra of compound **1ag** in $CDCl_3$

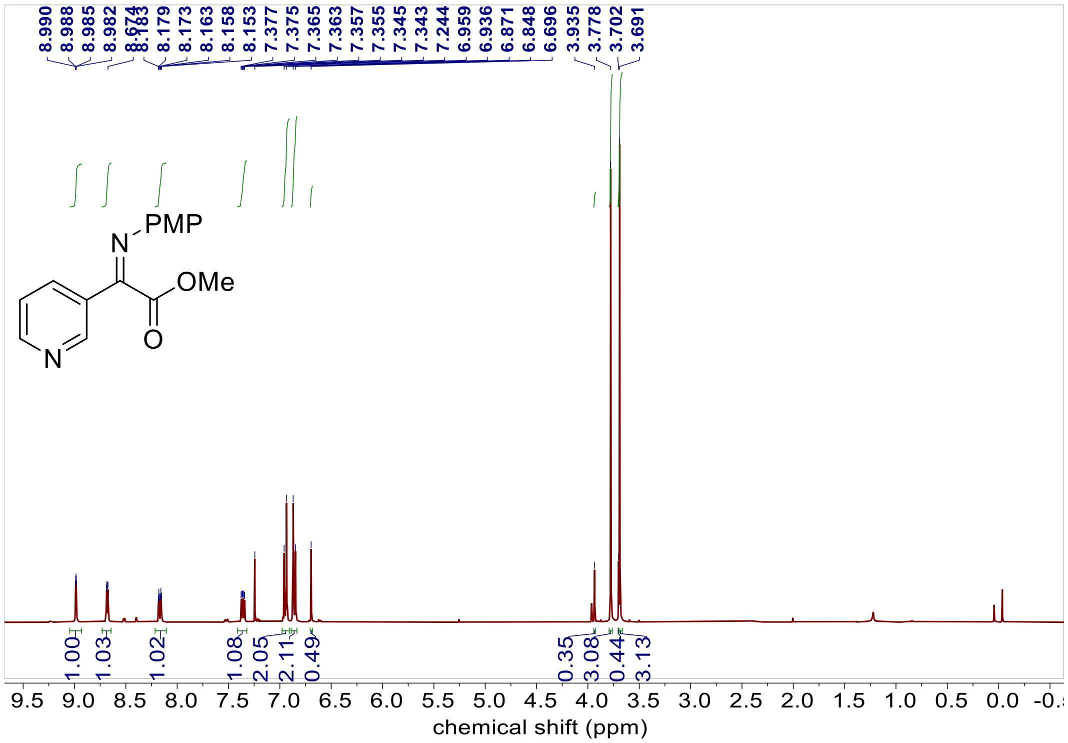

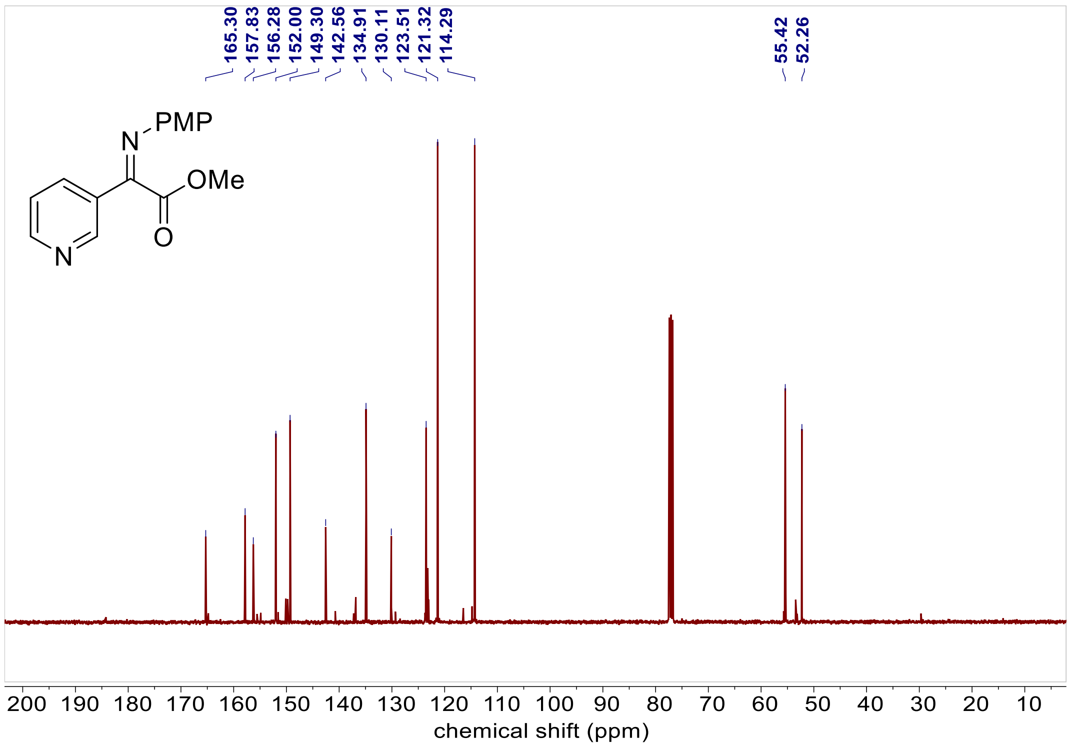

Supplementary Figure 34. ^{1}H NMR & ^{13}C NMR spectra of compound **1ah** in $CDCl_3$

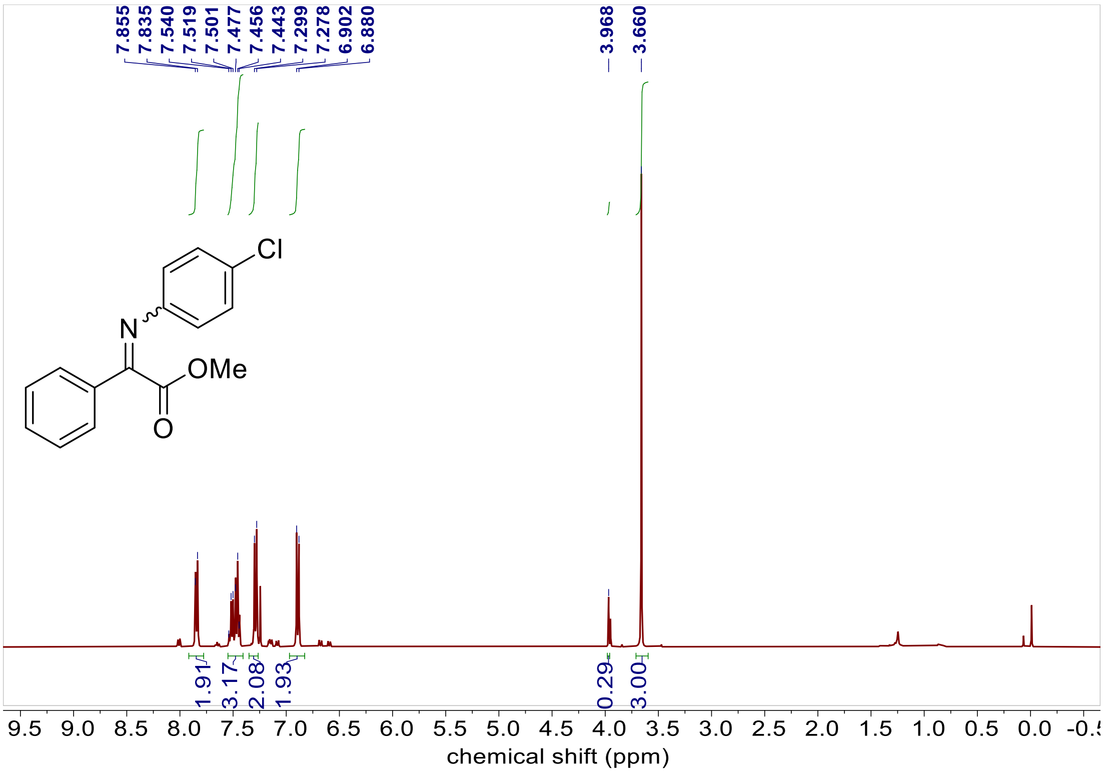

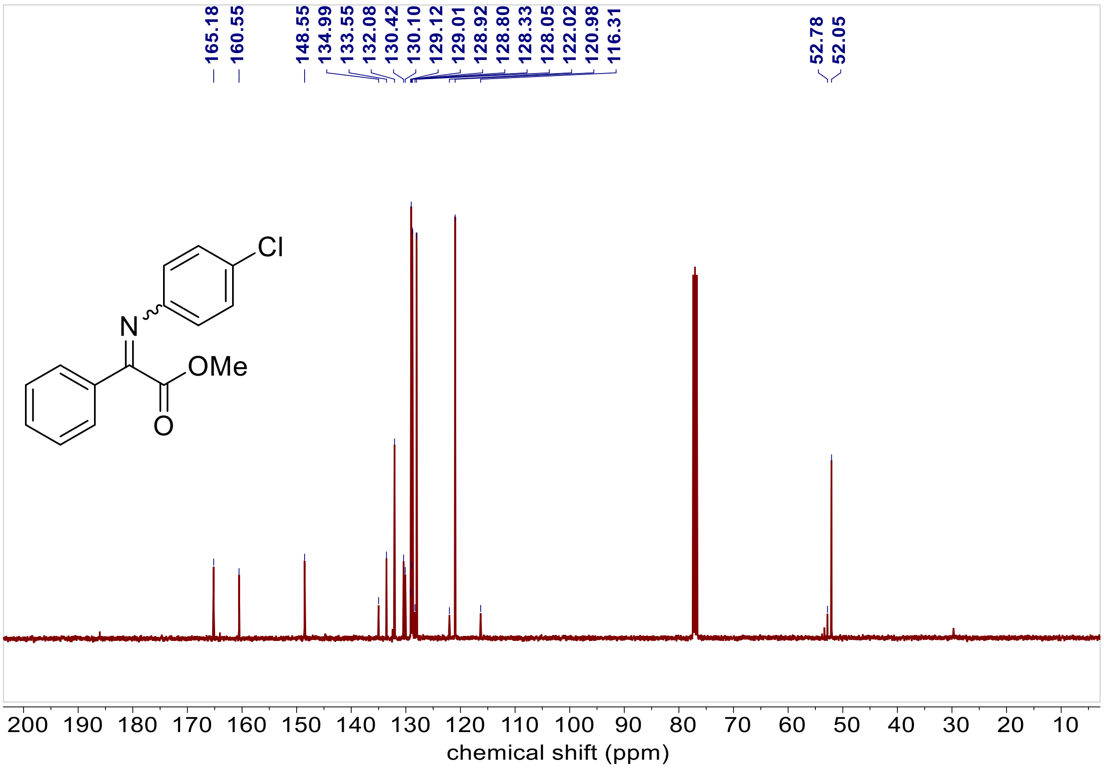

Supplementary Figure 35. ^{1}H NMR & ^{13}C NMR spectra of compound **1ak** in $CDCl_3$

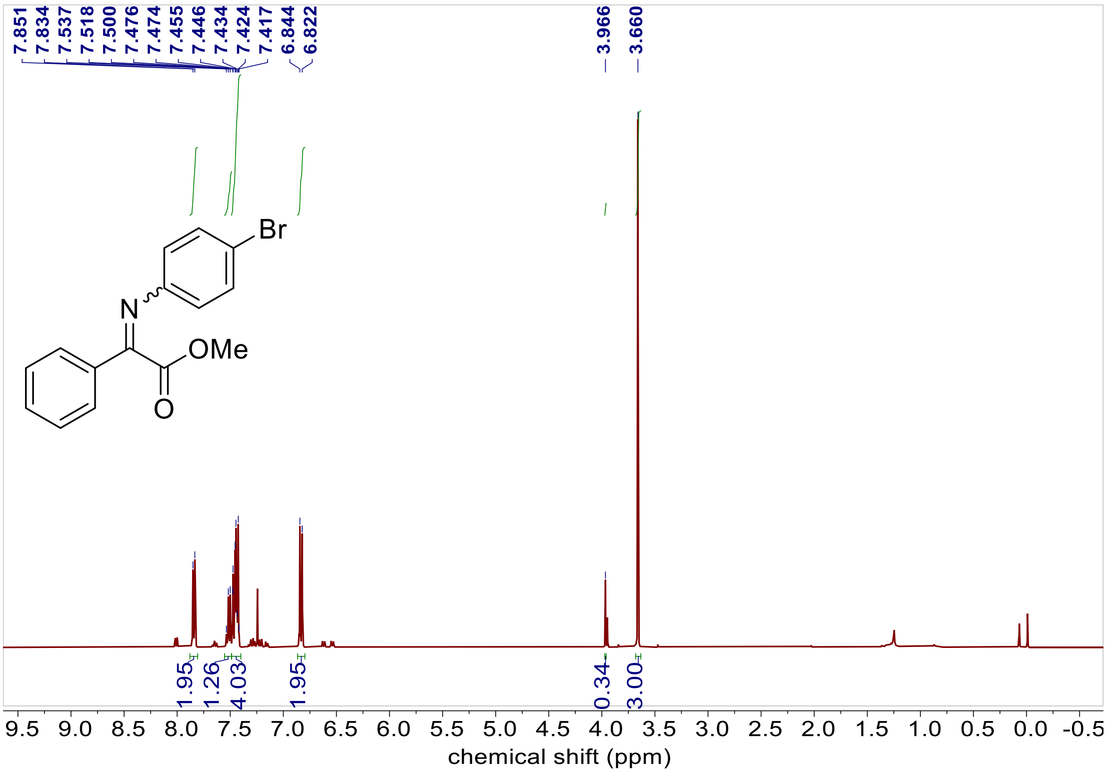

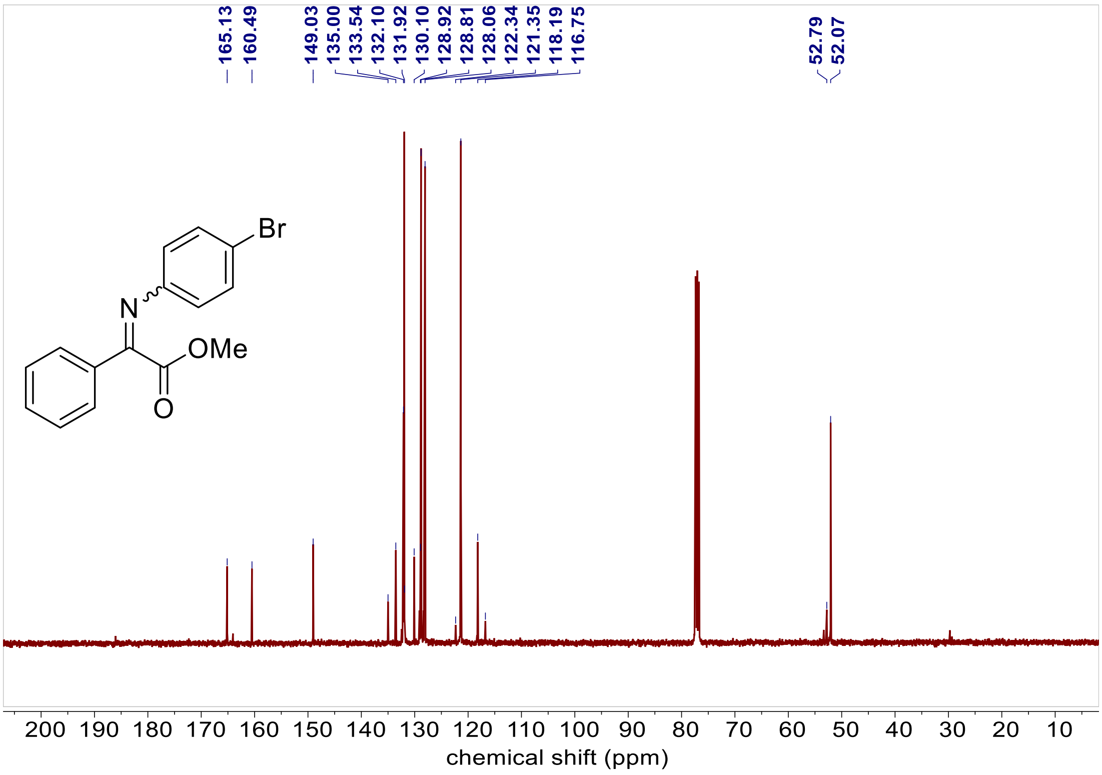

Supplementary Figure 36. ^{1}H NMR & ^{13}C NMR spectra of compound **1al** in $CDCl_3$

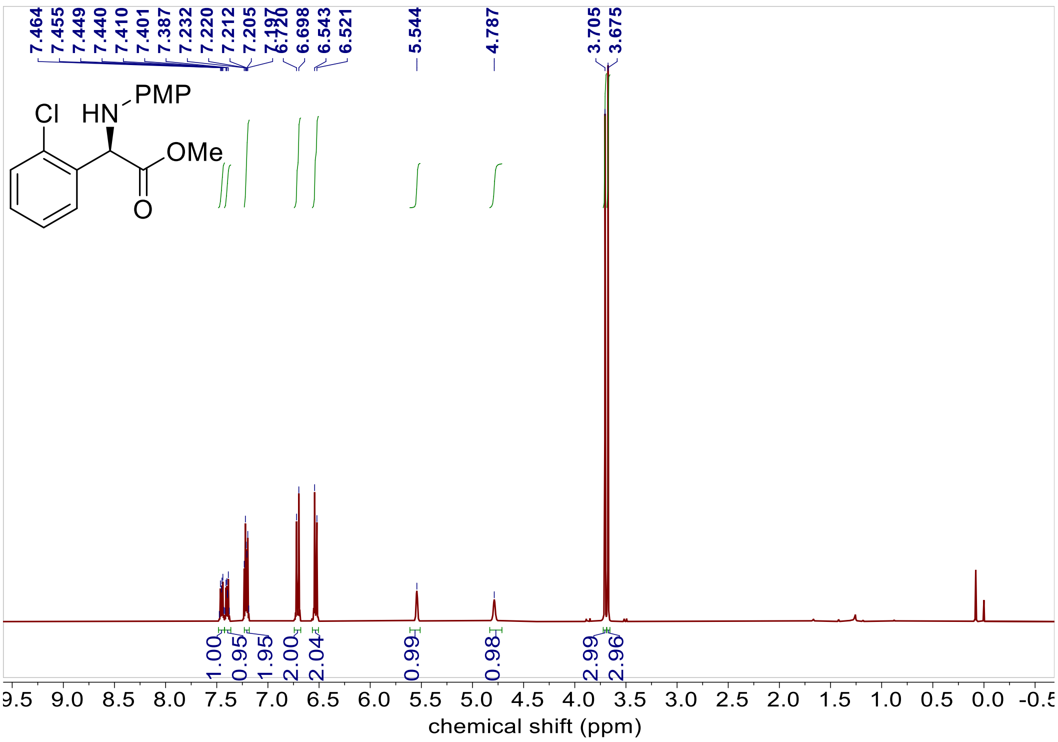

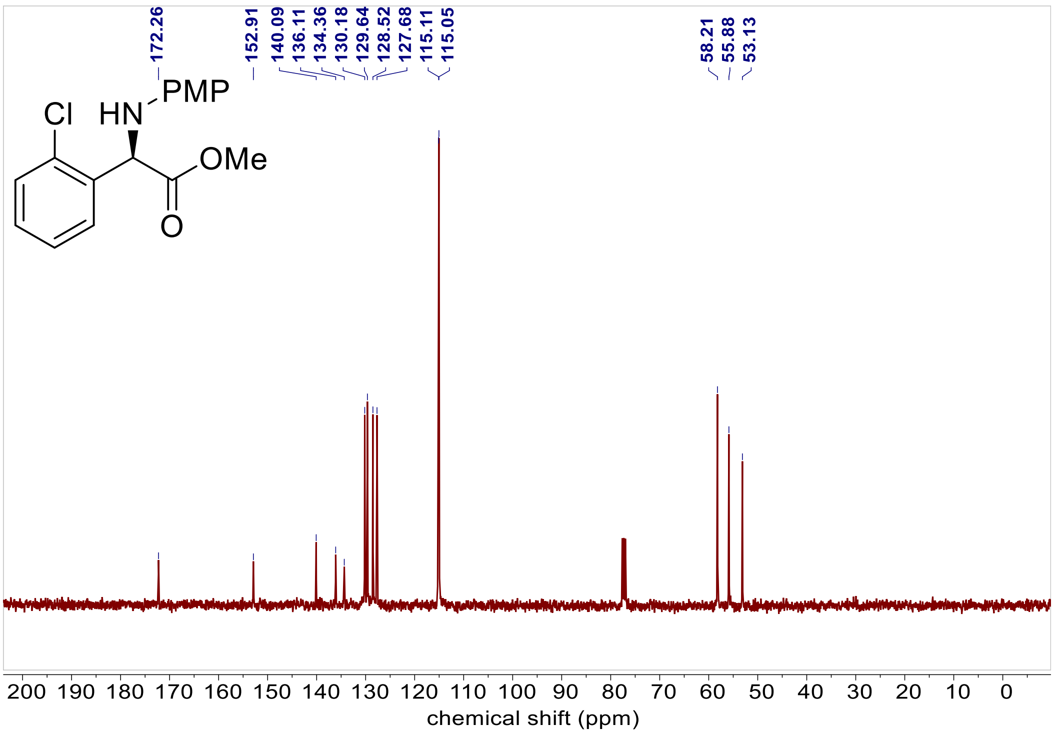

Supplementary Figure 37. ^{1}H NMR & ^{13}C NMR spectra of compound **2c** in $CDCl_3$

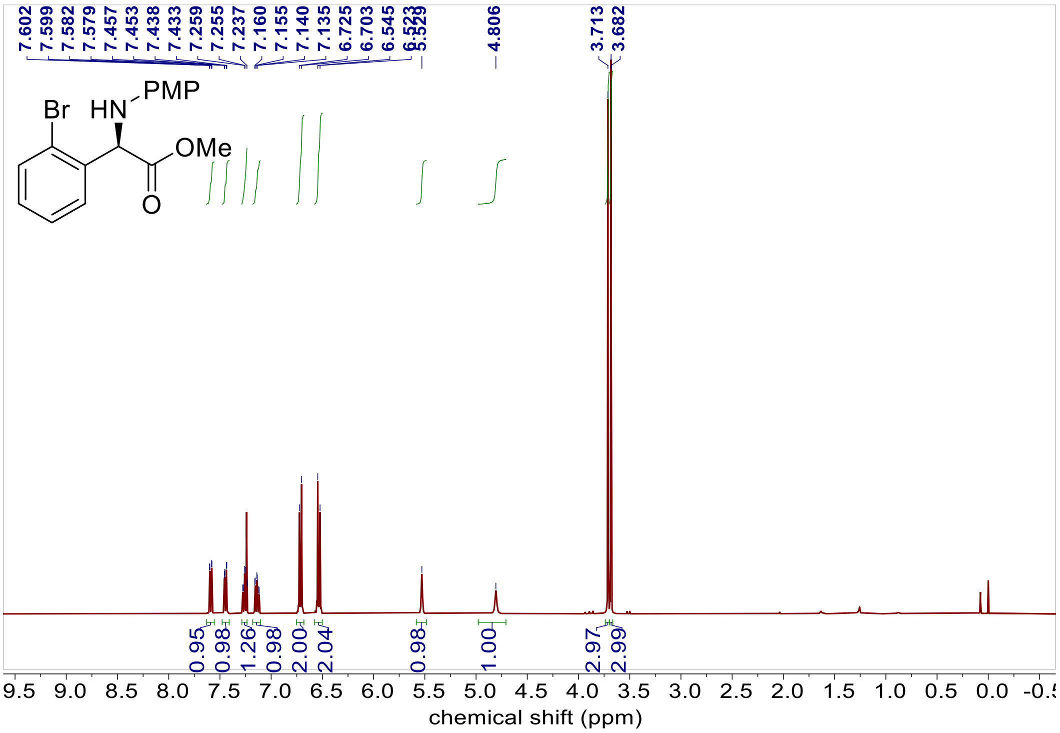

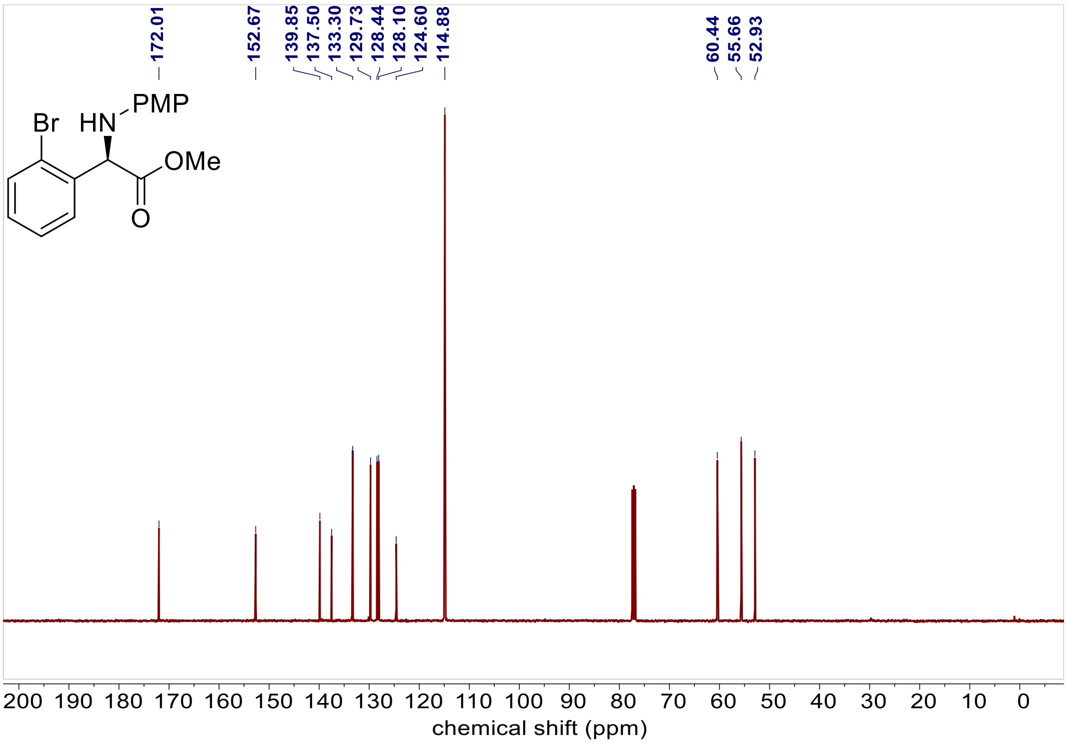

Supplementary Figure 38. ^{1}H NMR & ^{13}C NMR spectra of compound **2d** in $CDCl_3$

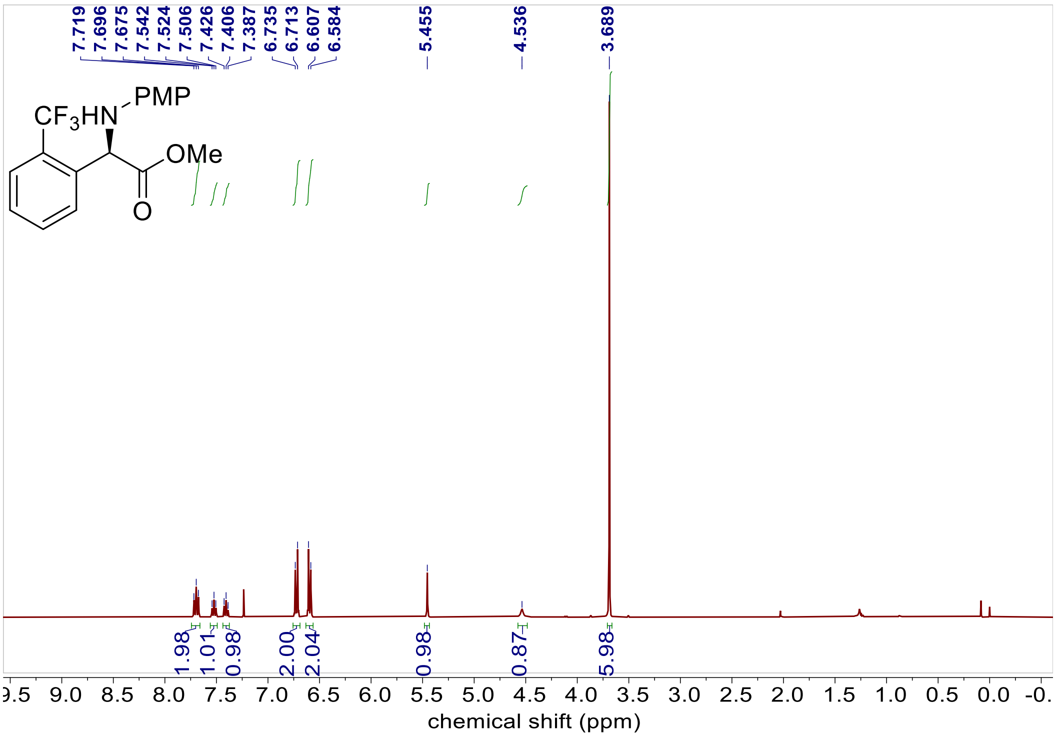
7.719
7.696
7.675
7.542
7.524
7.506
7.426
7.406
7.387
6.735
6.713
6.607
6.584
5.455
4.536
3.689
CF3HN
PMP
OMe
O
1.98
1.01
0.98
2.00
2.04
0.98
0.87
5.98
chemical shift (ppm)

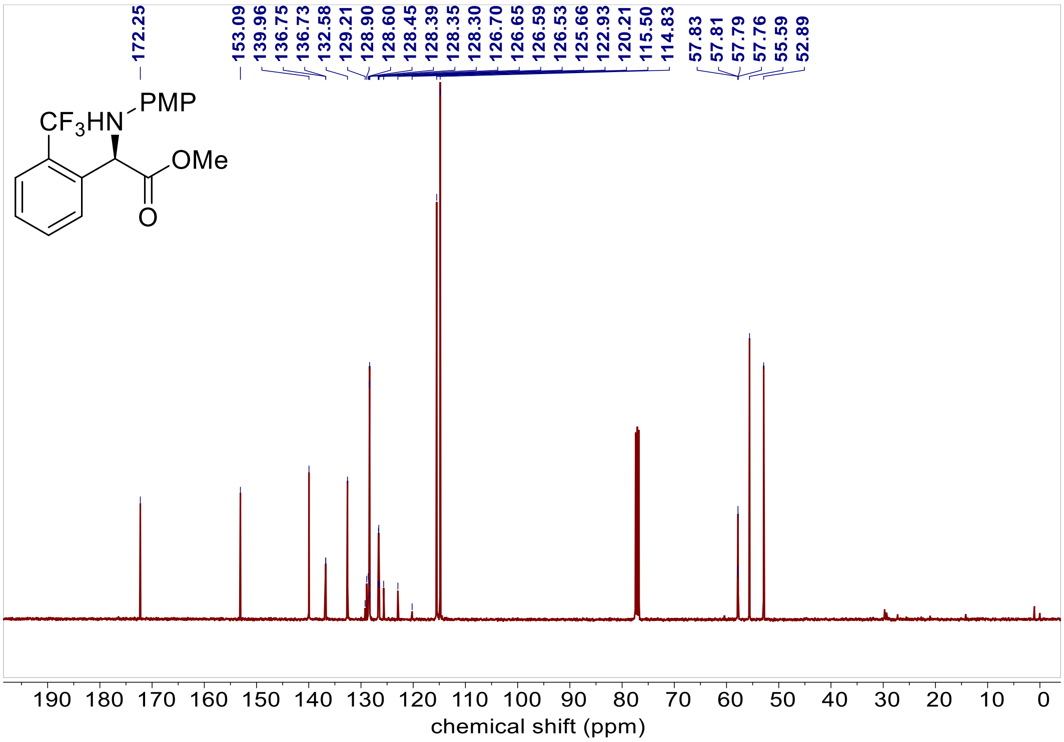
172.25
153.09
139.96
136.75
136.73
132.58
129.21
128.90
128.60
128.45
128.39
128.35
128.30
126.70
126.65
126.59
126.53
125.66
122.93
120.21
115.50
114.83
57.83
57.81
57.79
57.76
55.59
52.89
CF3HN
PMP
OMe
O
chemical shift (ppm)

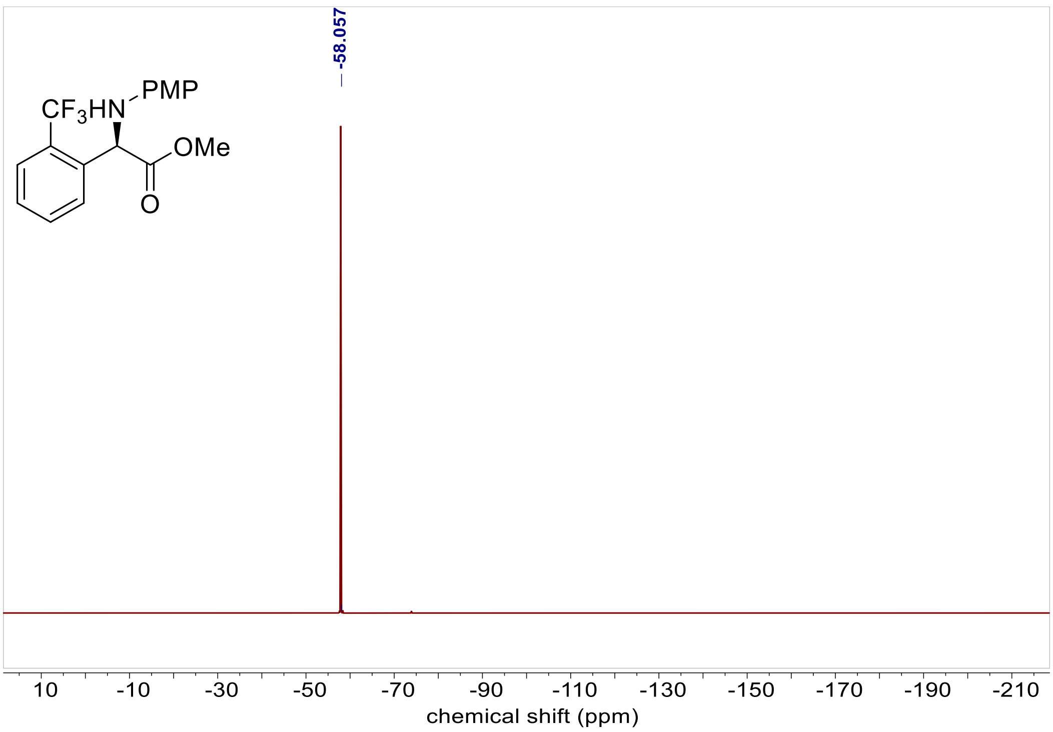

Supplementary Figure 39. ^{1}H NMR & ^{13}C NMR & ^{19}F NMR spectra of compound **2e** in $CDCl_3$

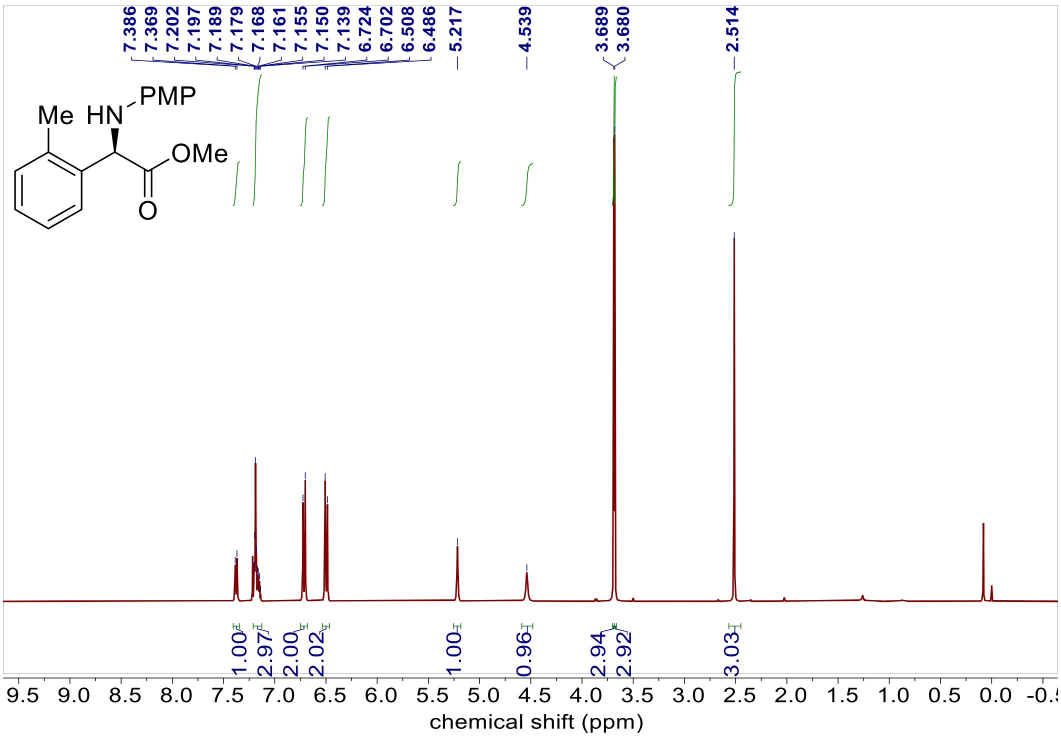

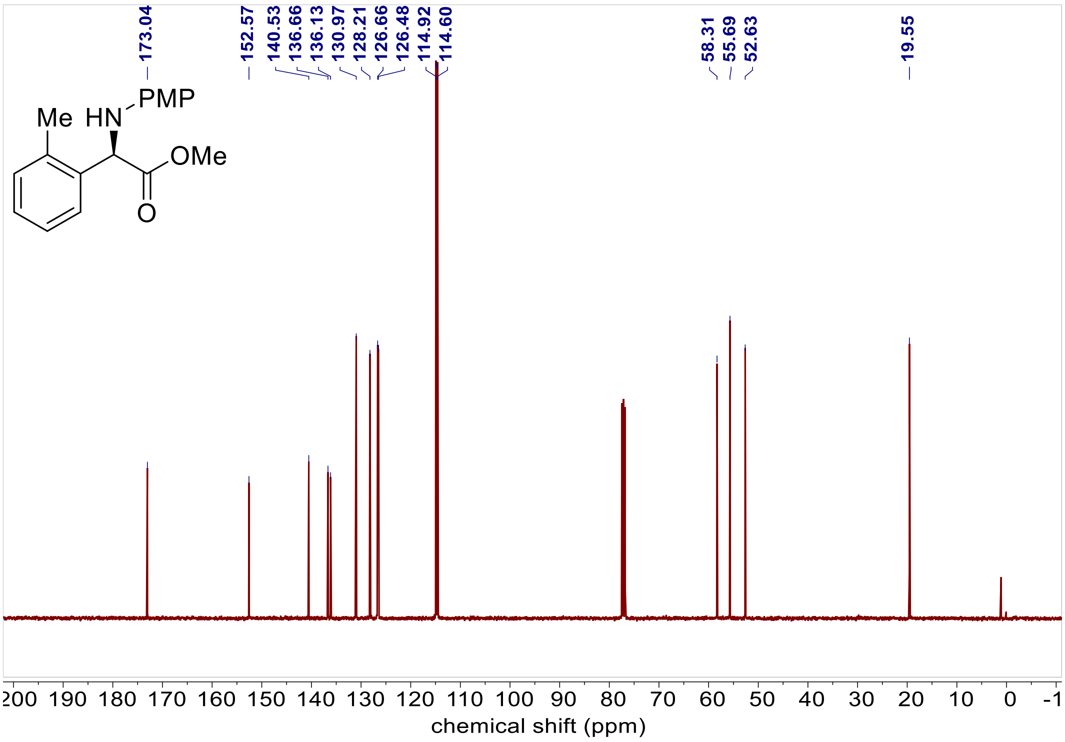

Supplementary Figure 40. ^{1}H NMR & ^{13}C NMR spectra of compound **2f** in $CDCl_3$

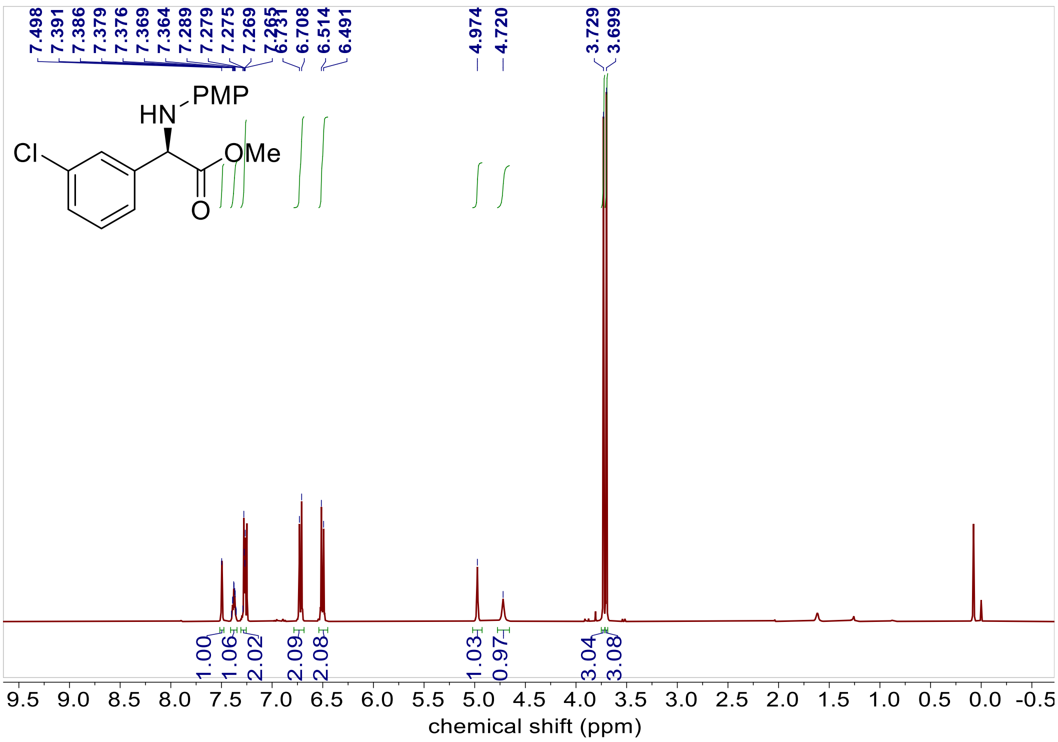

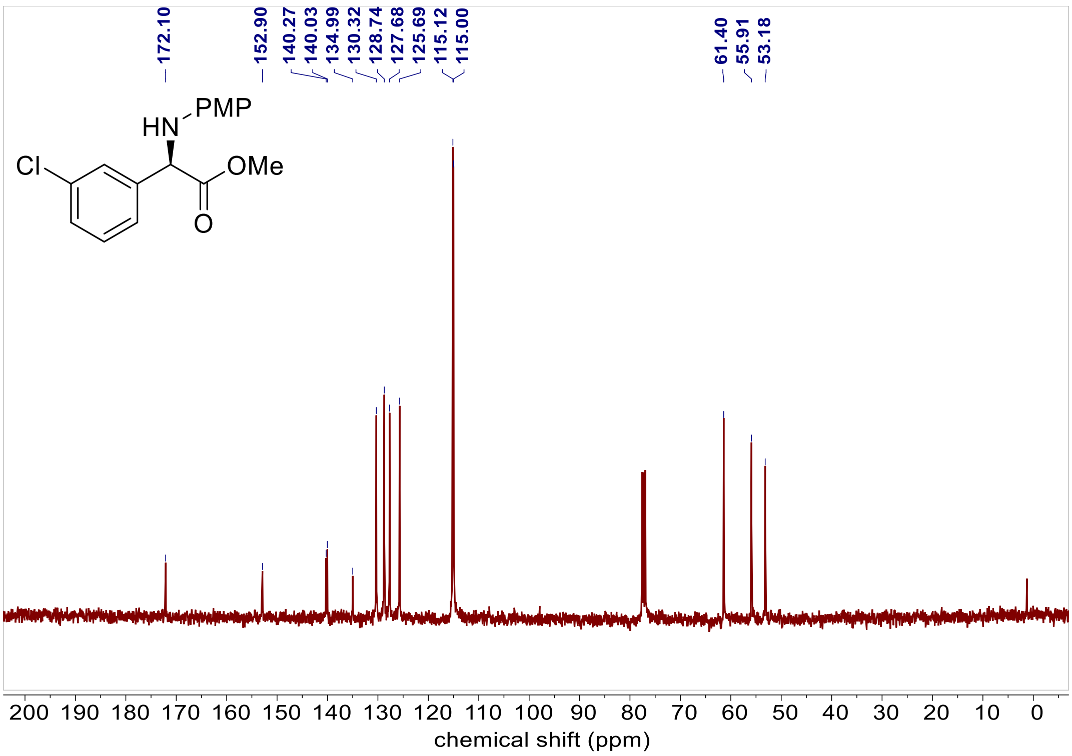

Supplementary Figure 41. ^{1}H NMR & ^{13}C NMR spectra of compound **2i** in $CDCl_3$

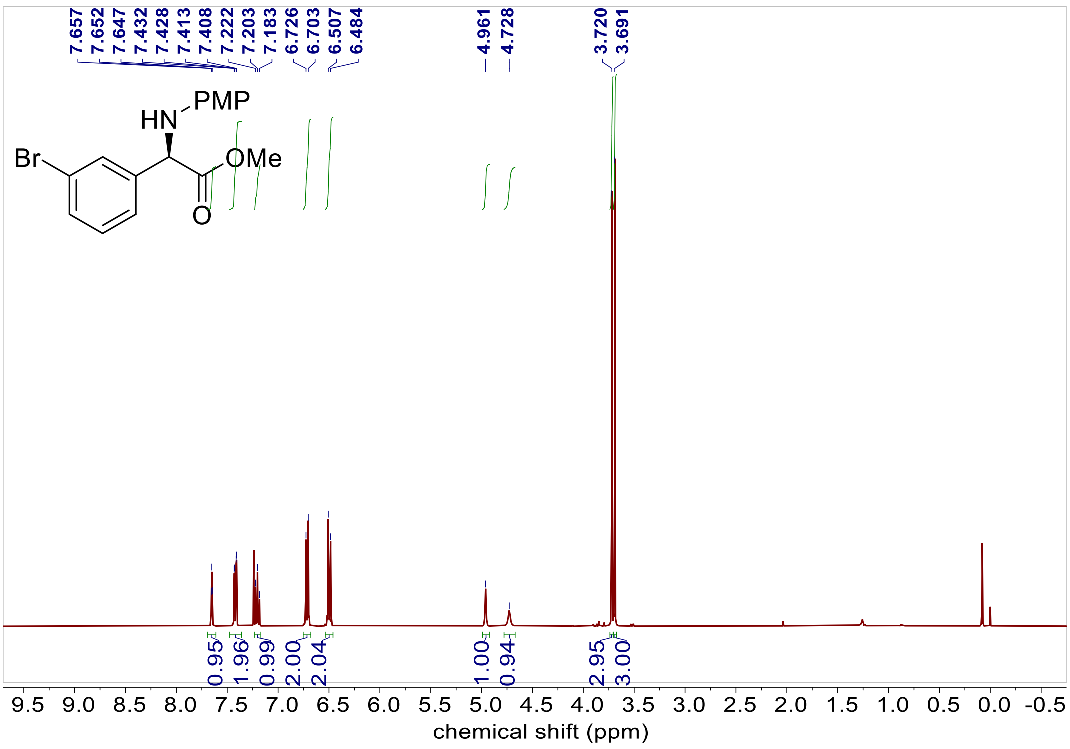

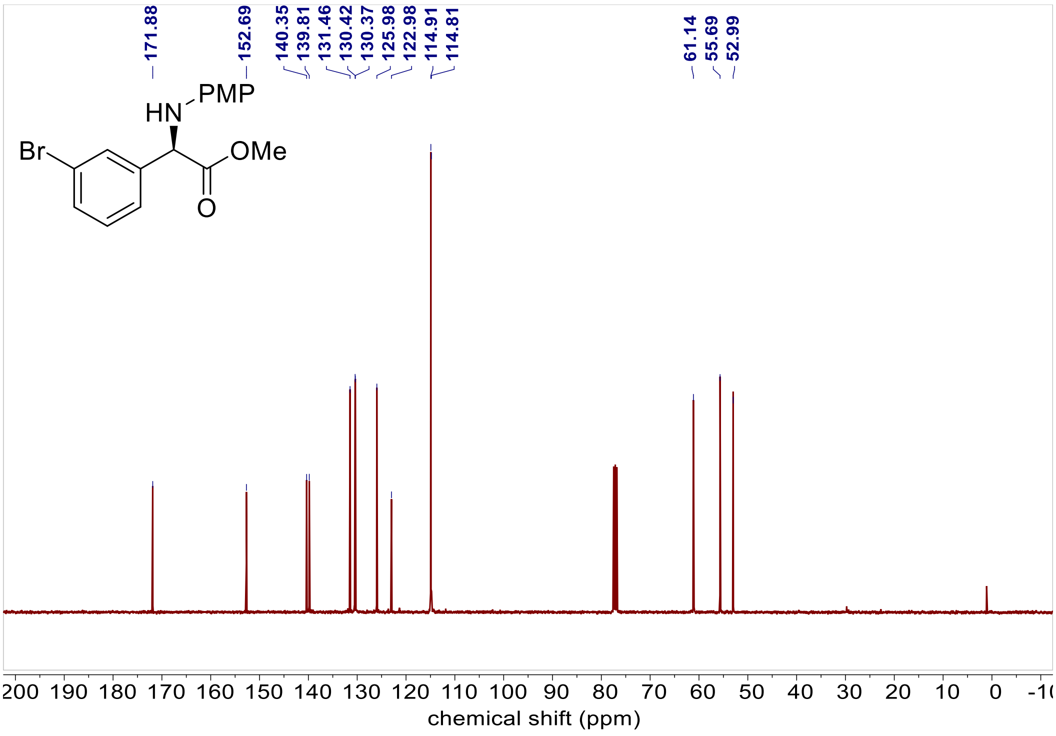

Supplementary Figure 42. ^{1}H NMR & ^{13}C NMR spectra of compound **2j** in $CDCl_3$

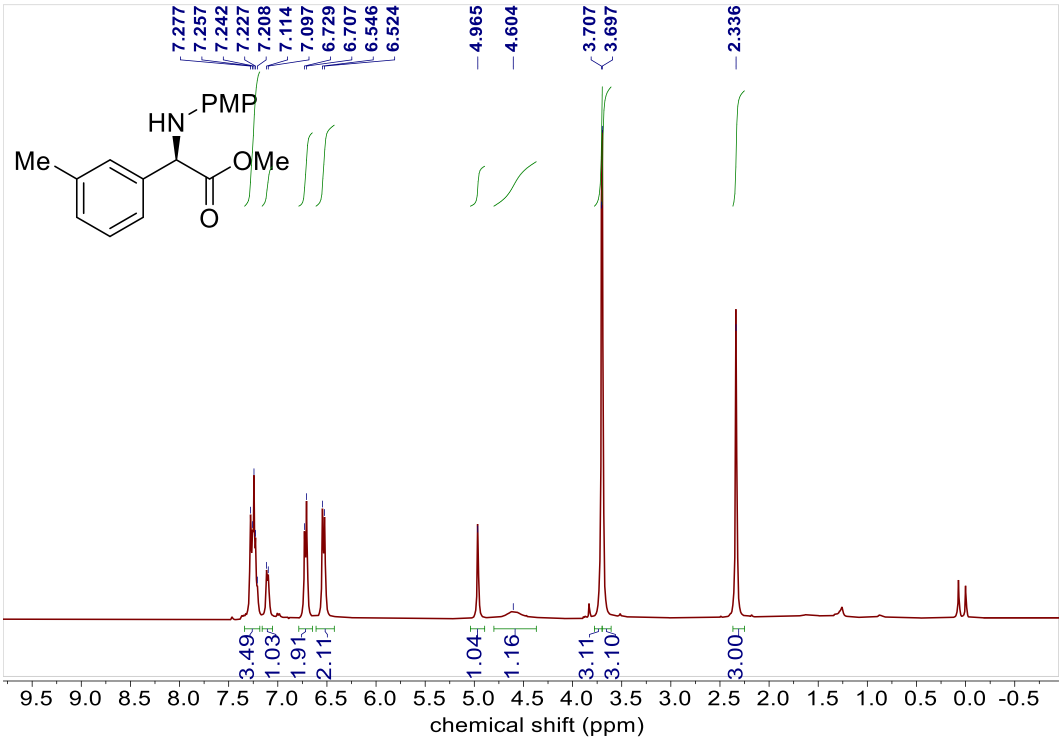

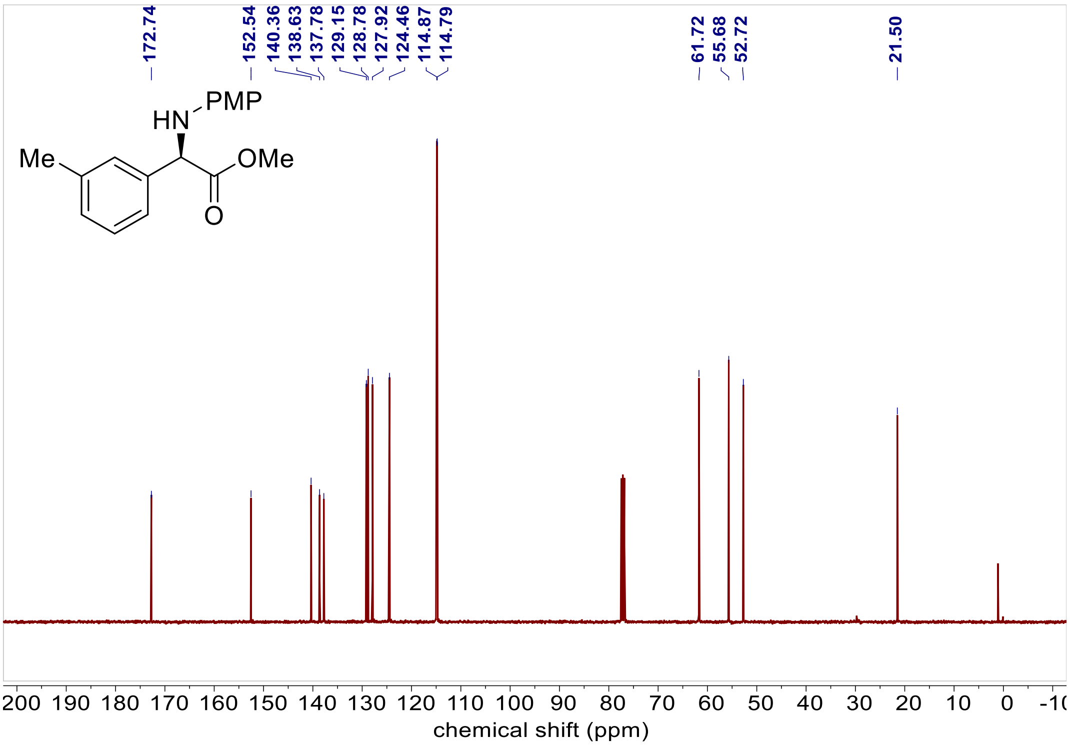

Supplementary Figure 43. ^{1}H NMR & ^{13}C NMR spectra of compound **2l** in $CDCl_3$

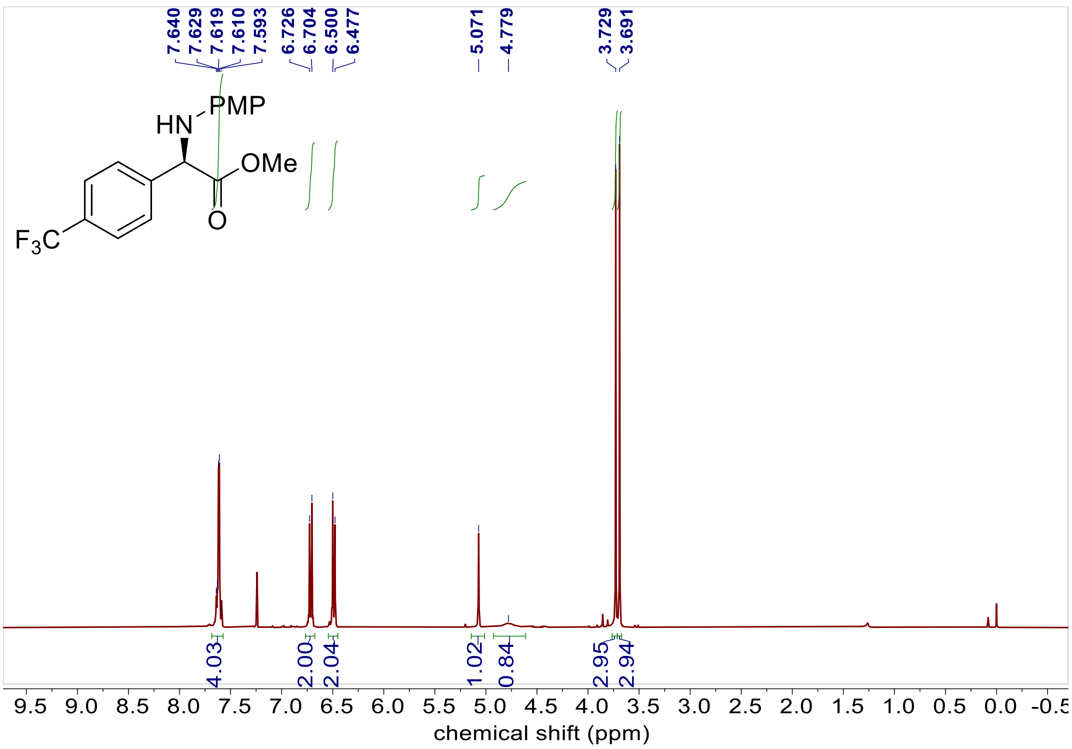

7.640
7.629
7.619
7.610
7.593
6.726
6.704
6.500
6.477
5.071
4.779
3.729
3.691
HN
PMP
OMe
O
F3C
4.03
2.00
2.04
1.02
0.84
2.95
2.94
9.5 9.0 8.5 8.0 7.5 7.0 6.5 6.0 5.5 5.0 4.5 4.0 3.5 3.0 2.5 2.0 1.5 1.0 0.5 0.0 -0.5
chemical shift (ppm)

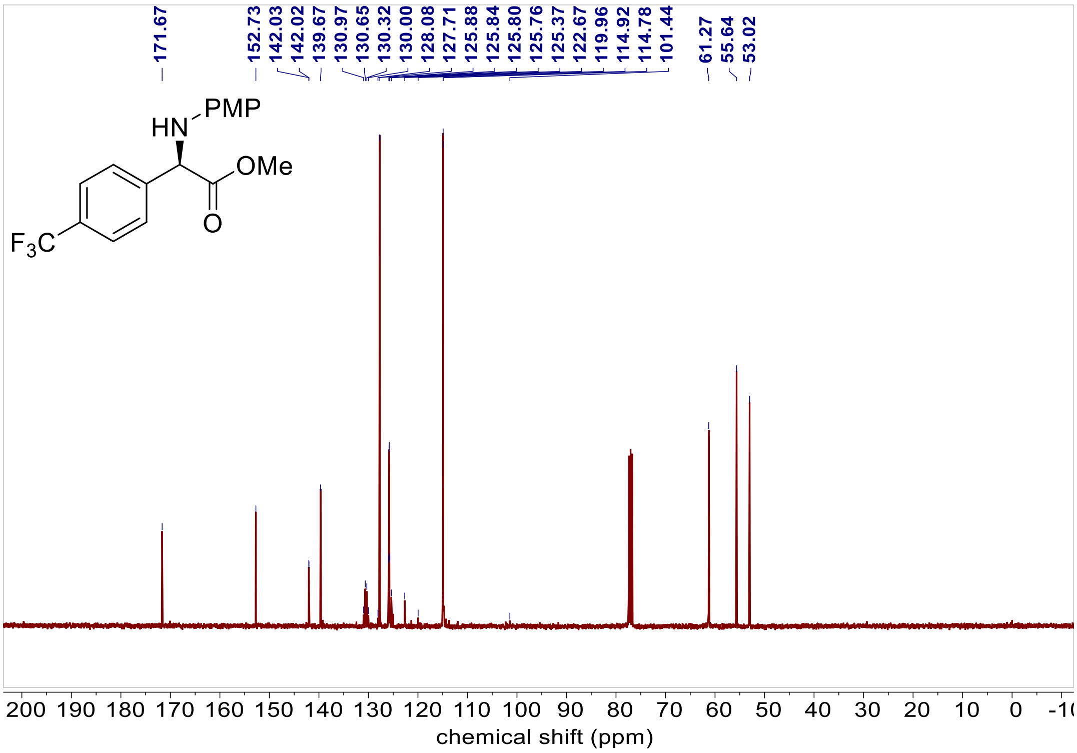

171.67
152.73
142.03
142.02
139.67
130.97
130.65
130.32
130.00
128.08
127.71
125.88
125.84
125.80
125.76
125.37
122.67
119.96
114.92
114.78
101.44
61.27
55.64
53.02
HN
PMP
OMe
O
F3C
200 190 180 170 160 150 140 130 120 110 100 90 80 70 60 50 40 30 20 10 0
chemical shift (ppm)

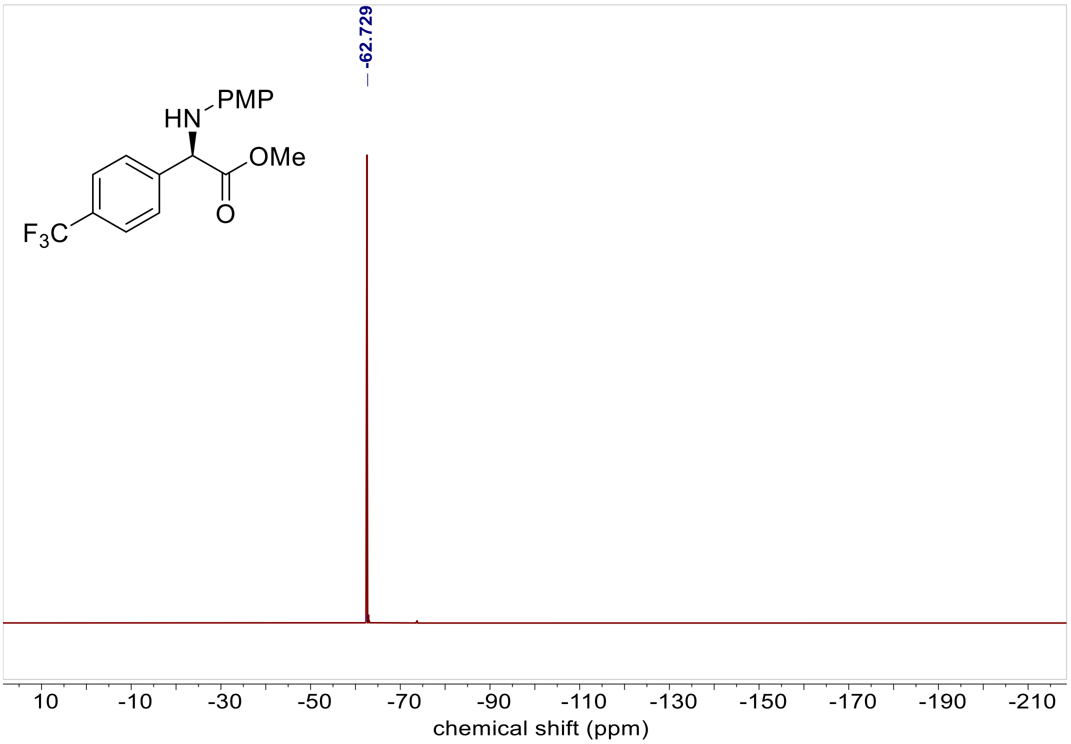

Supplementary Figure 44. ^{1}H NMR & ^{13}C NMR & ^{19}F NMR spectra of compound **2q** in $CDCl_3$

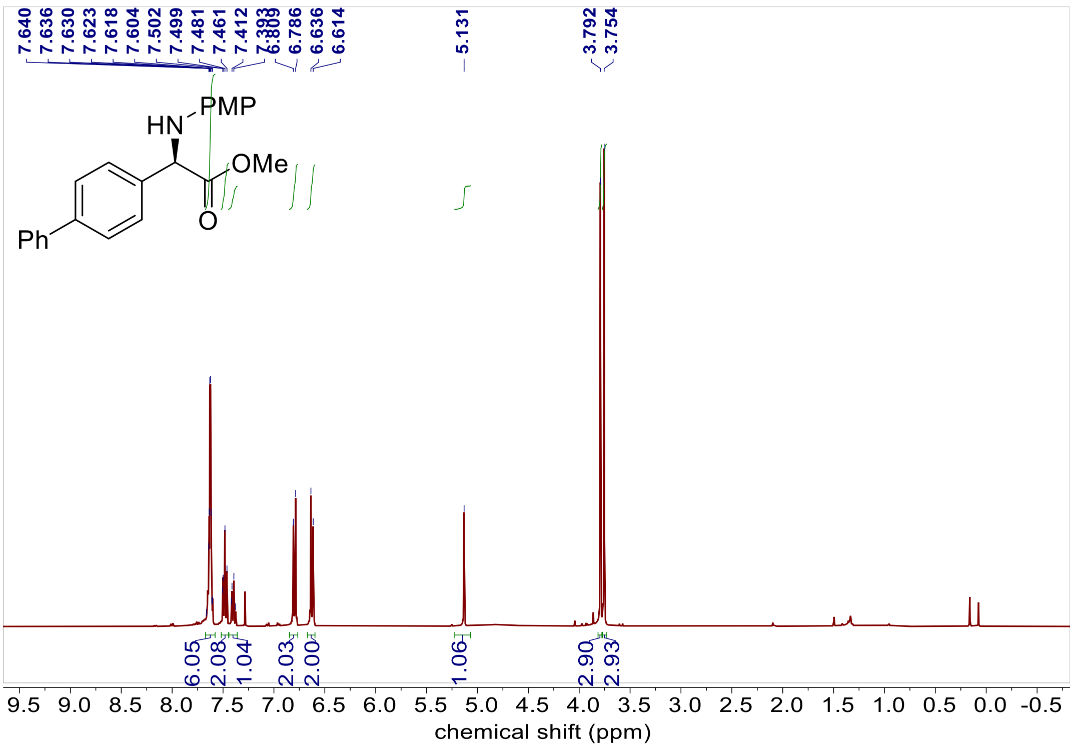

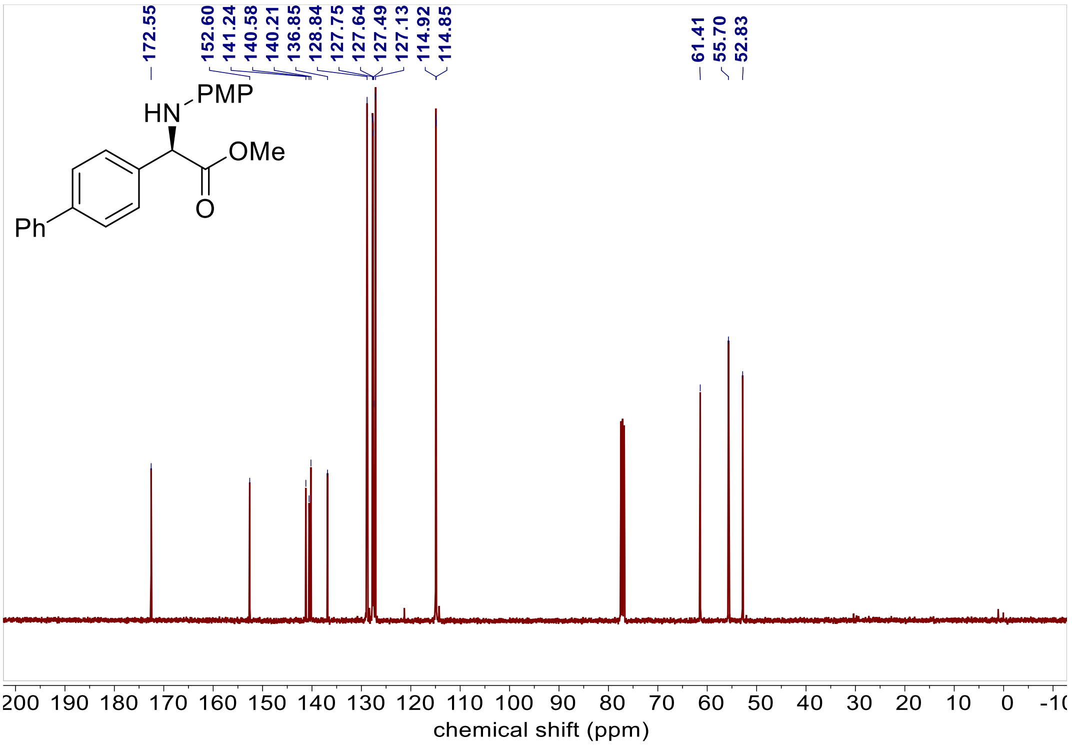

Supplementary Figure 45. ^{1}H NMR & ^{13}C NMR spectra of compound **2t** in $CDCl_3$

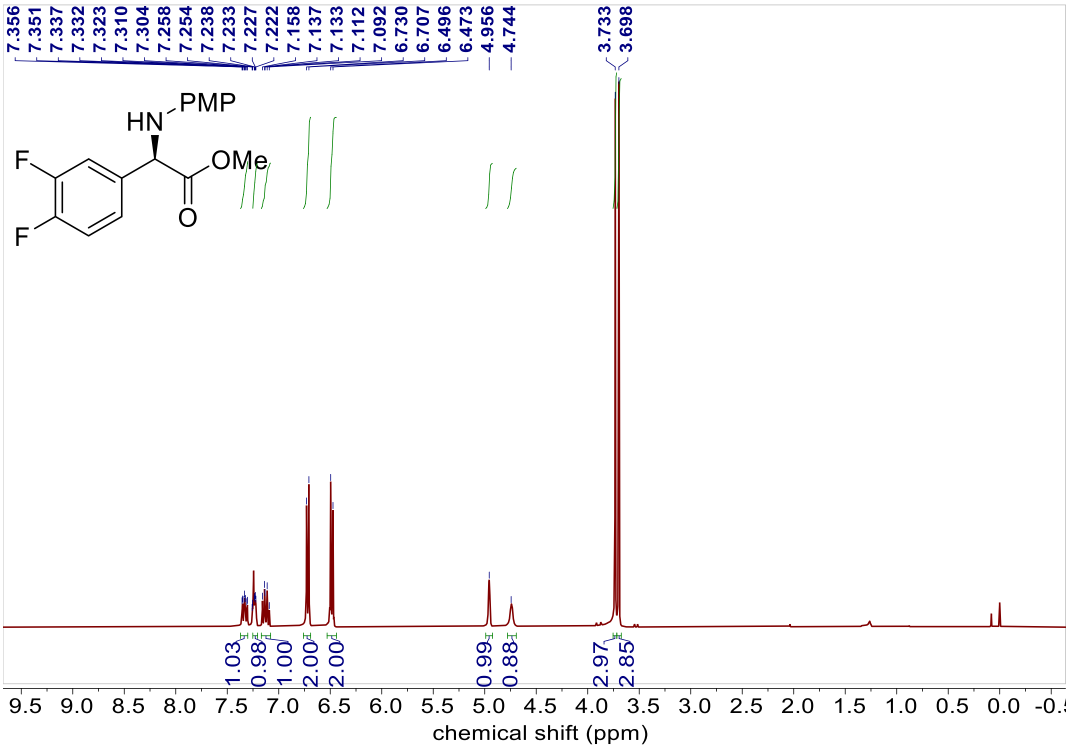
7.356
7.351
7.337
7.332
7.323
7.310
7.304
7.258
7.254
7.238
7.233
7.227
7.222
7.158
7.137
7.133
7.112
7.092
6.730
6.707
6.496
6.473
4.956
4.744
3.733
3.698
HN
PMP
F
F
OMe
O
1.03
0.98
1.00
2.00
2.00
0.99
0.88
2.97
2.85
9.5 9.0 8.5 8.0 7.5 7.0 6.5 6.0 5.5 5.0 4.5 4.0 3.5 3.0 2.5 2.0 1.5 1.0 0.5 0.0
chemical shift (ppm)

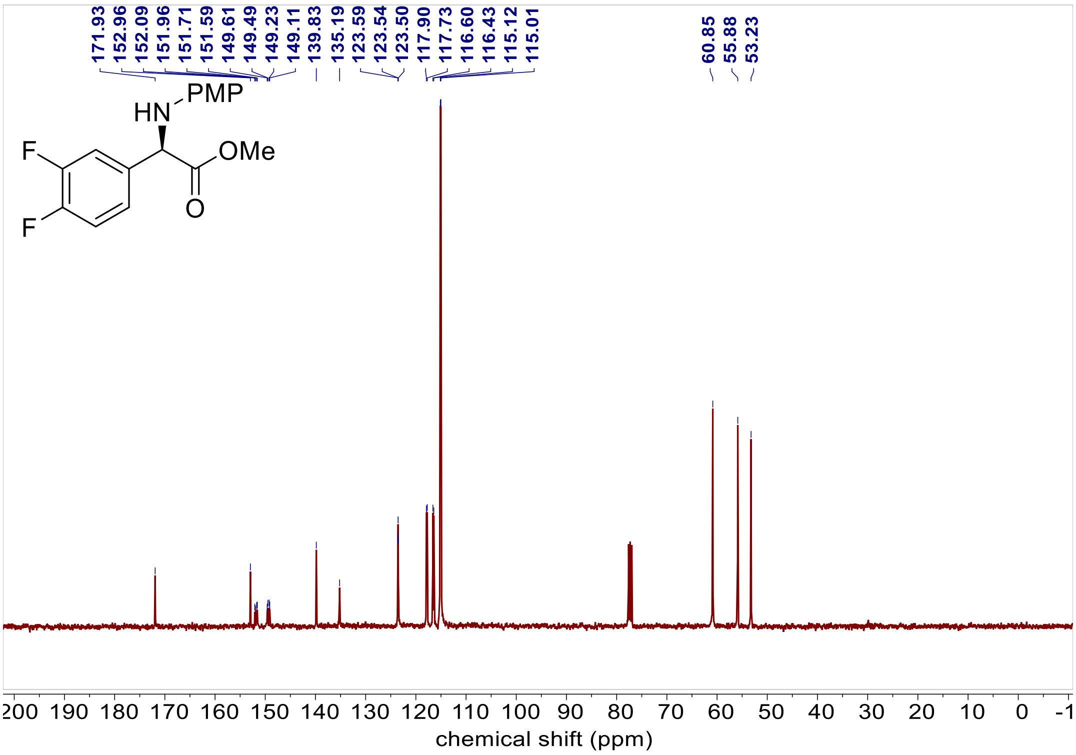
171.93
152.96
152.09
151.96
151.71
151.59
149.61
149.49
149.23
149.11
139.83
135.19
123.59
123.54
123.50
117.90
117.73
116.60
116.43
115.12
115.01
60.85
55.88
53.23
HN
PMP
F
F
OMe
O
200 190 180 170 160 150 140 130 120 110 100 90 80 70 60 50 40 30 20 10 0
chemical shift (ppm)

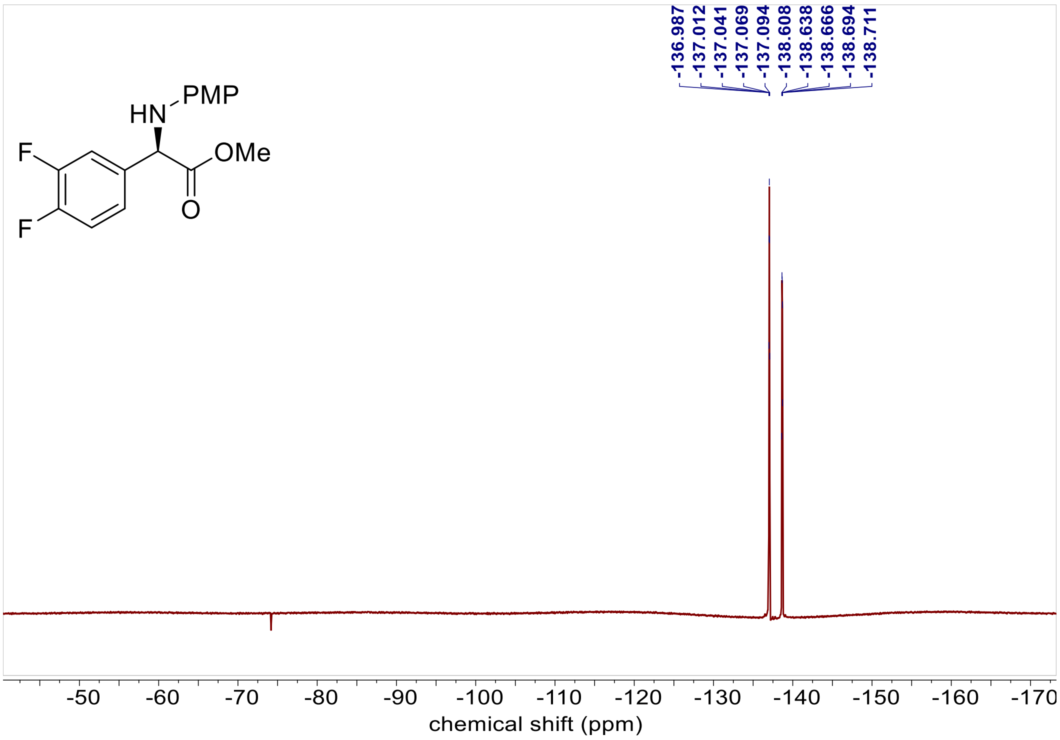

Supplementary Figure 46. ^{1}H NMR & ^{13}C NMR & ^{19}F NMR spectra of compound **2u** in $CDCl_3$

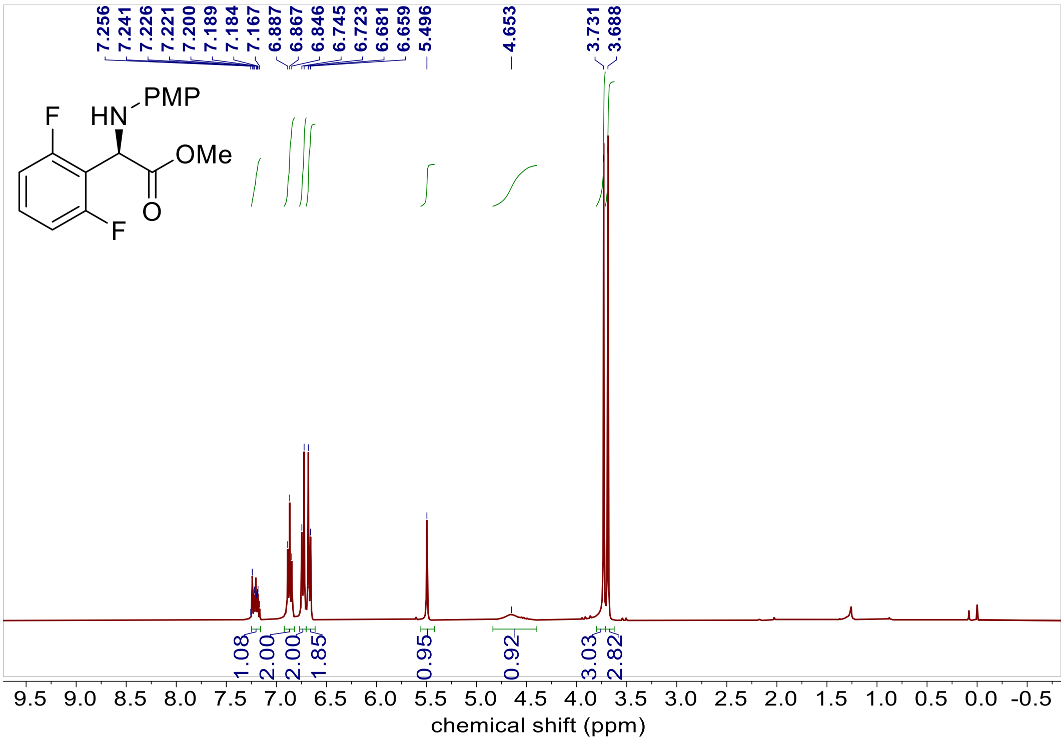
7.256
7.241
7.226
7.221
7.200
7.189
7.184
7.167
6.887
6.867
6.846
6.745
6.723
6.681
6.659
5.496
4.653
3.731
3.688
F
HN
PMP
OMe
O
F
1.08
2.00
2.00
1.85
0.95
0.92
3.03
2.82
9.5 9.0 8.5 8.0 7.5 7.0 6.5 6.0 5.5 5.0 4.5 4.0 3.5 3.0 2.5 2.0 1.5 1.0 0.5 0.0 -0.5
chemical shift (ppm)

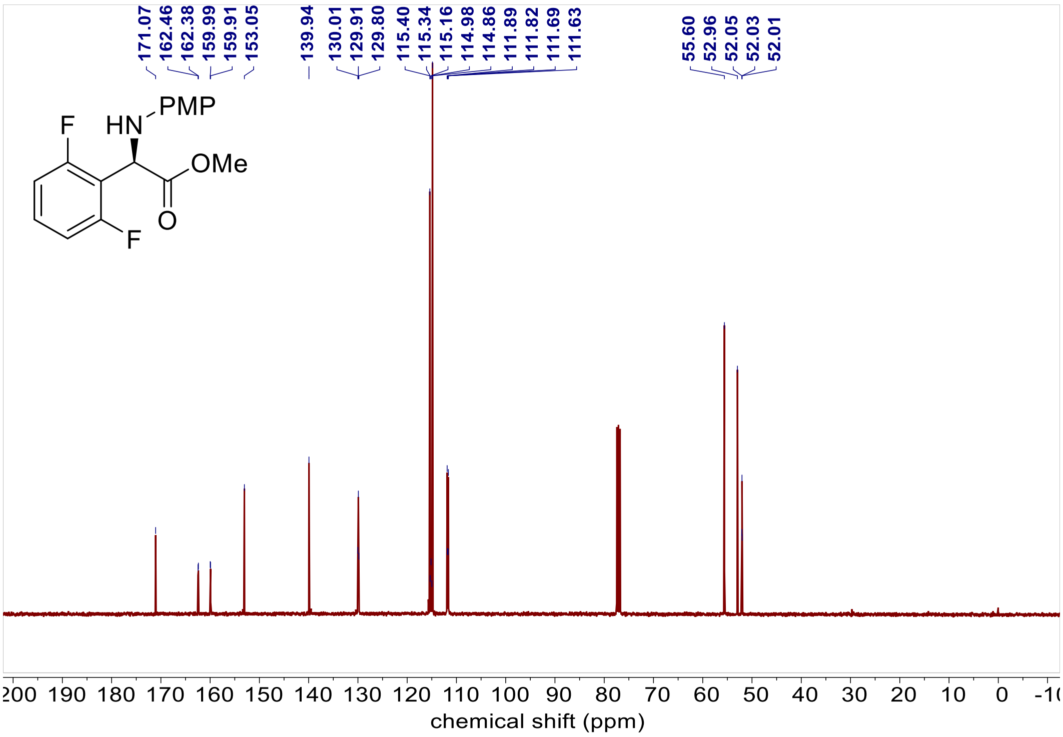
171.07
162.46
162.38
159.99
159.91
153.05
139.94
130.01
129.91
129.80
115.40
115.34
115.16
114.98
114.86
111.89
111.82
111.69
111.63
55.60
52.96
52.05
52.03
52.01
F
HN
PMP
OMe
O
F
200 190 180 170 160 150 140 130 120 110 100 90 80 70 60 50 40 30 20 10 0
chemical shift (ppm)

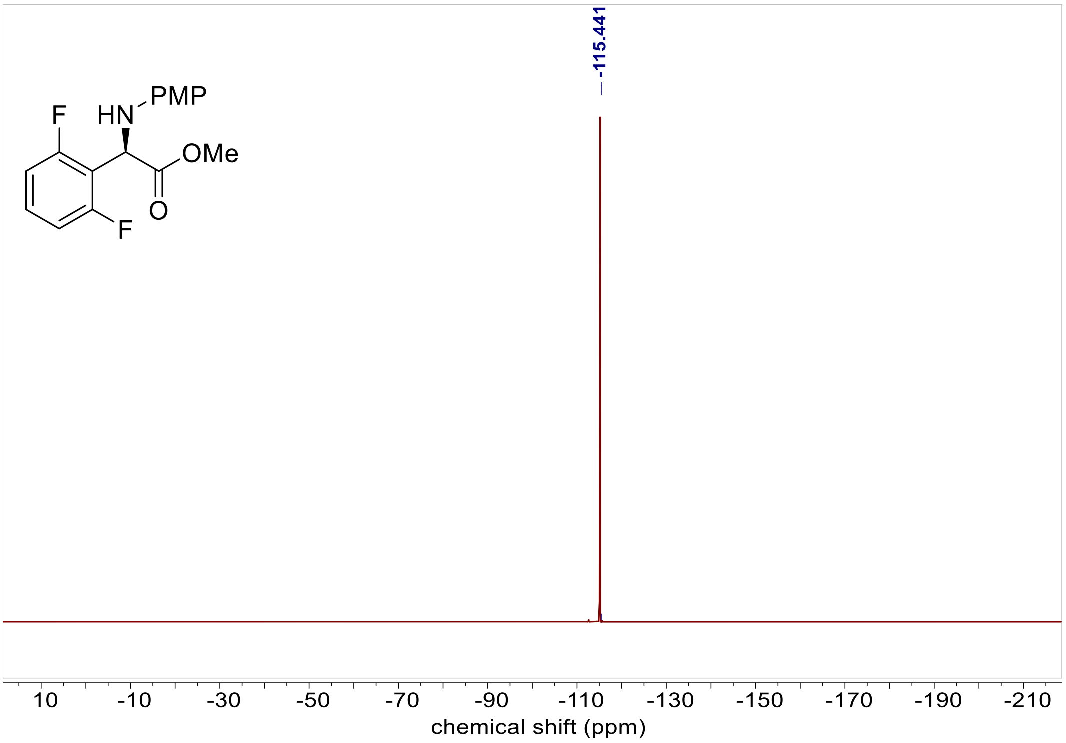

Supplementary Figure 47. ^1H NMR & ^{13}C NMR & ^{19}F NMR spectra of compound **2v** in $CDCl_3$

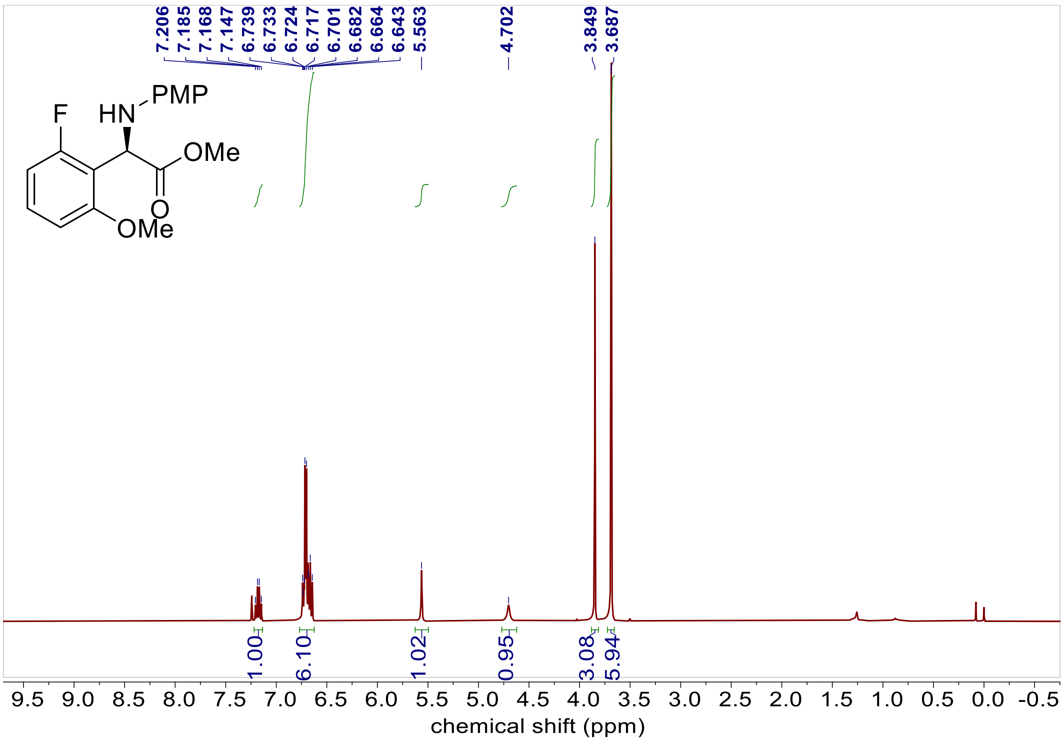

7.206
7.185
7.168
7.147
6.739
6.733
6.724
6.717
6.701
6.682
6.664
6.643
5.563
4.702
3.849
3.687
F
HN
PMP
OMe
O
OMe
1.00
6.10
1.02
0.95
3.08
5.94
9.5 9.0 8.5 8.0 7.5 7.0 6.5 6.0 5.5 5.0 4.5 4.0 3.5 3.0 2.5 2.0 1.5 1.0 0.5 0.0 -0.5
chemical shift (ppm)

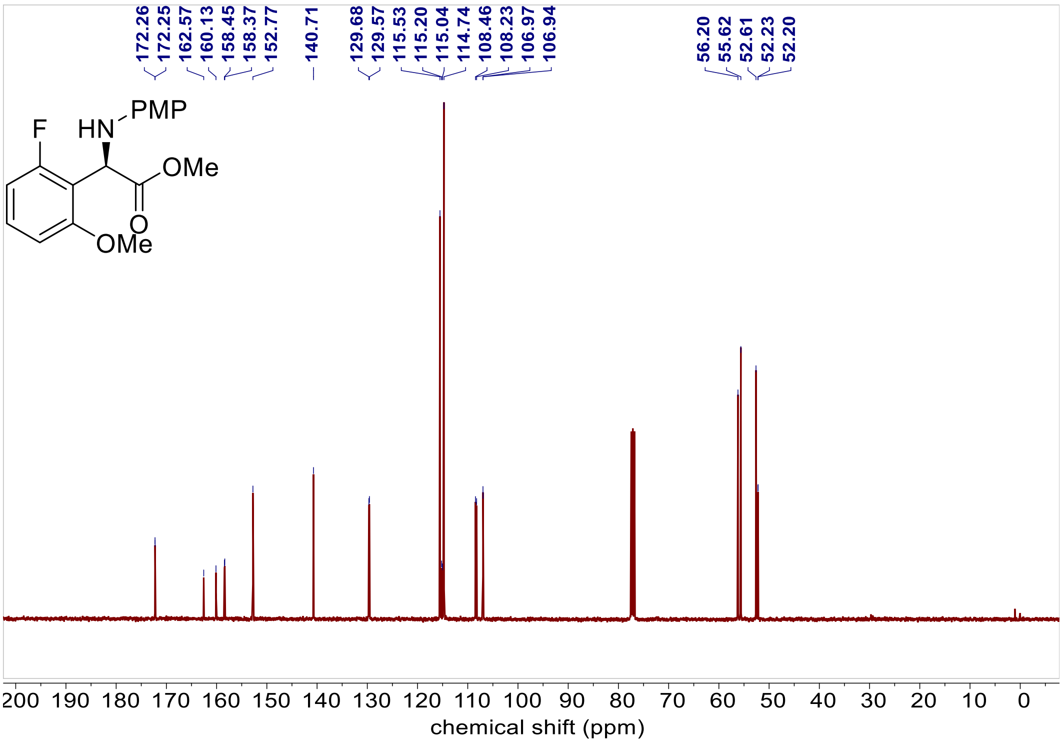

172.26
172.25
162.57
160.13
158.45
158.37
152.77
140.71
129.68
129.57
115.53
115.20
115.04
114.74
108.46
108.23
106.97
106.94
56.20
55.62
52.61
52.23
52.20
F
HN
PMP
OMe
O
OMe
200 190 180 170 160 150 140 130 120 110 100 90 80 70 60 50 40 30 20 10 0
chemical shift (ppm)

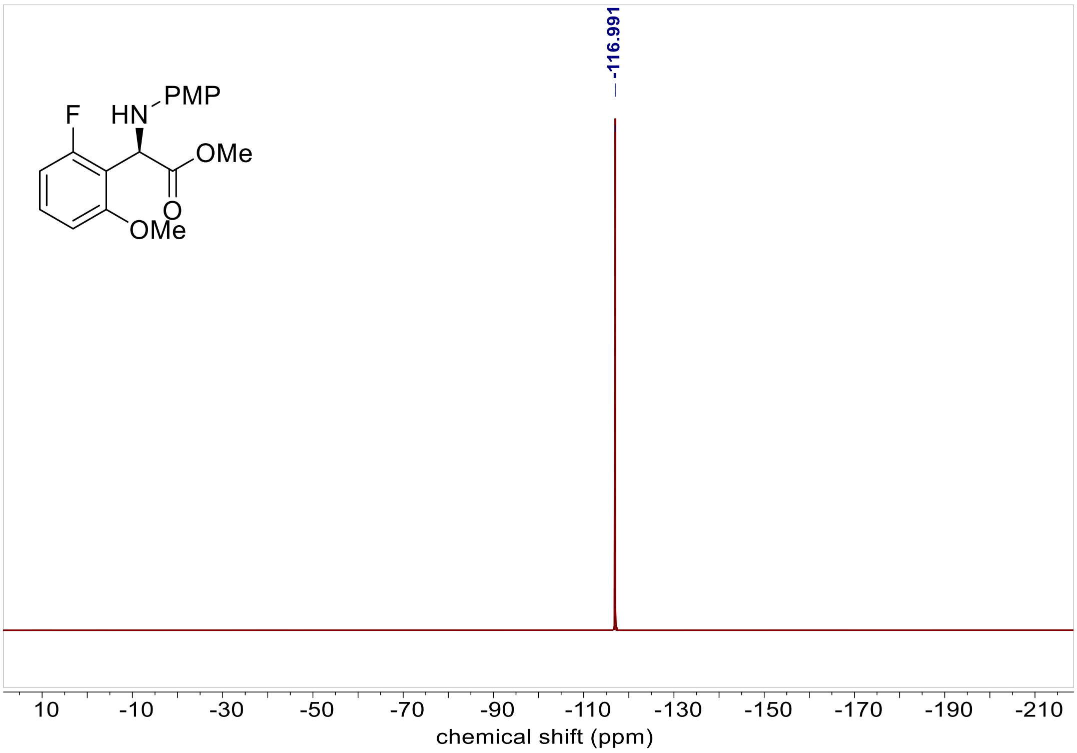

Supplementary Figure 48. ^{1}H NMR & ^{13}C NMR & ^{19}F NMR spectra of compound **2w** in $CDCl_3$

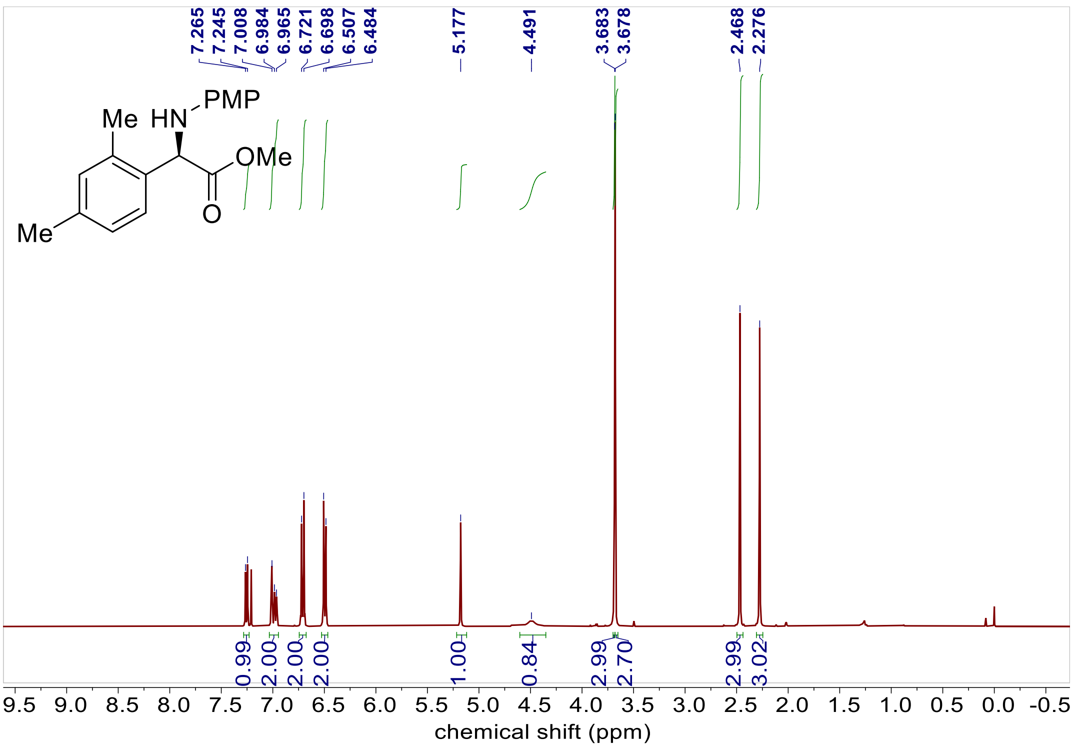

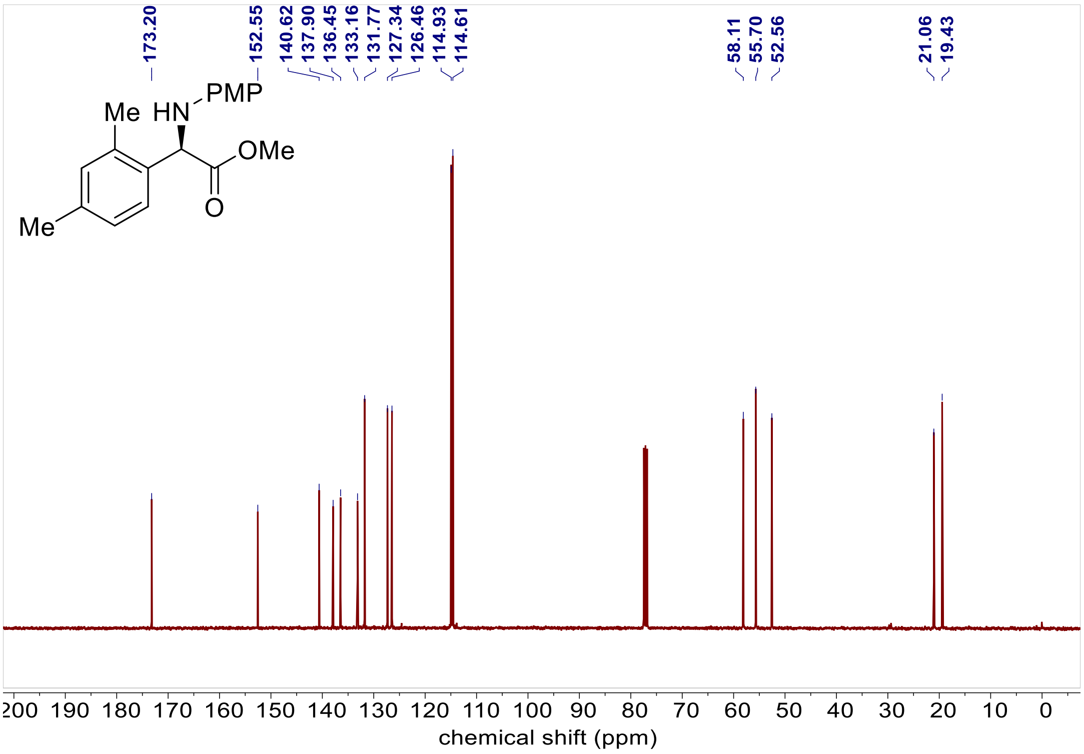

Supplementary Figure 49. ^{1}H NMR & ^{13}C NMR spectra of compound **2x** in $CDCl_3$

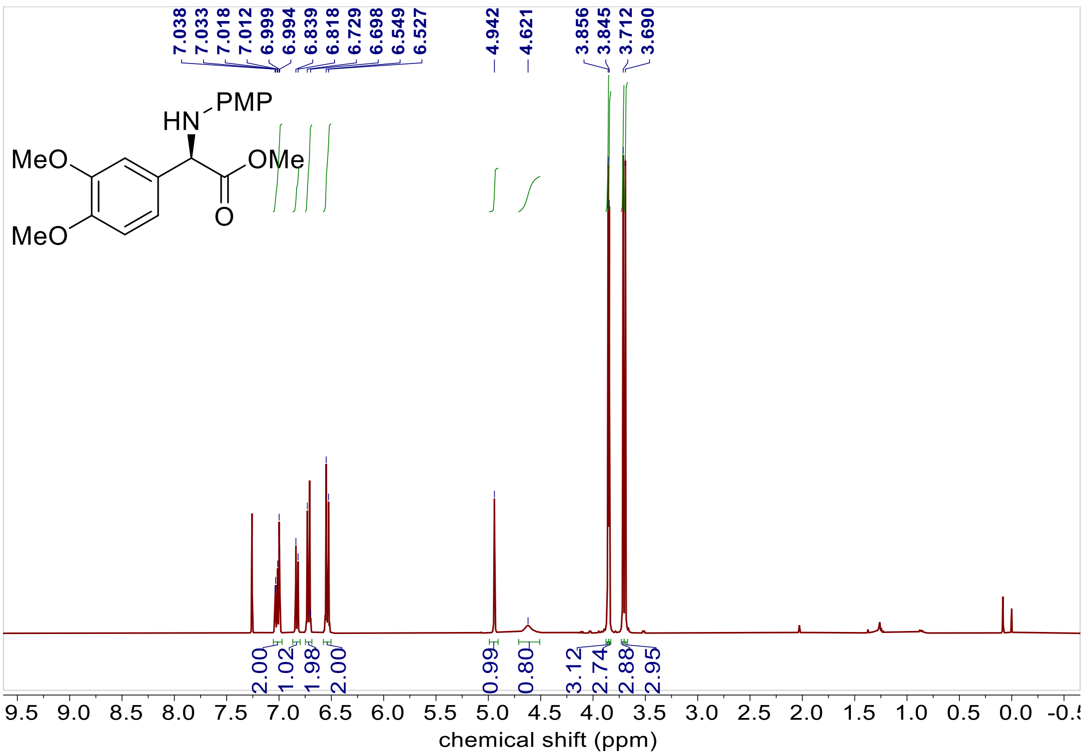

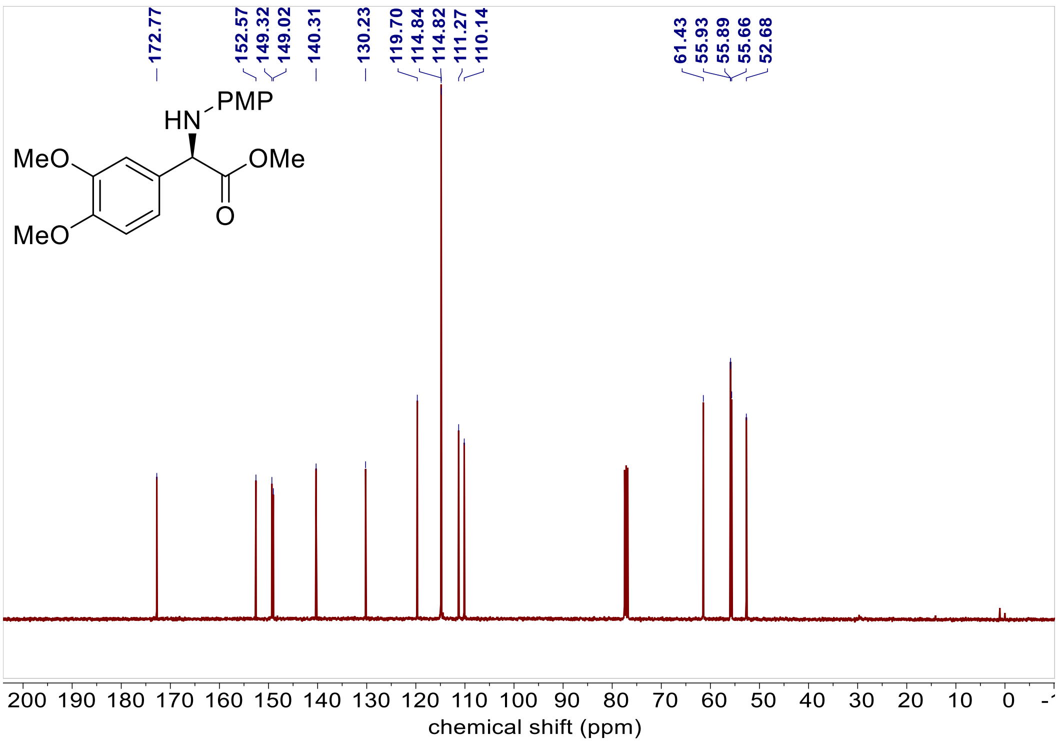

Supplementary Figure 50. ^{1}H NMR & ^{13}C NMR spectra of compound **2y** in $CDCl_3$

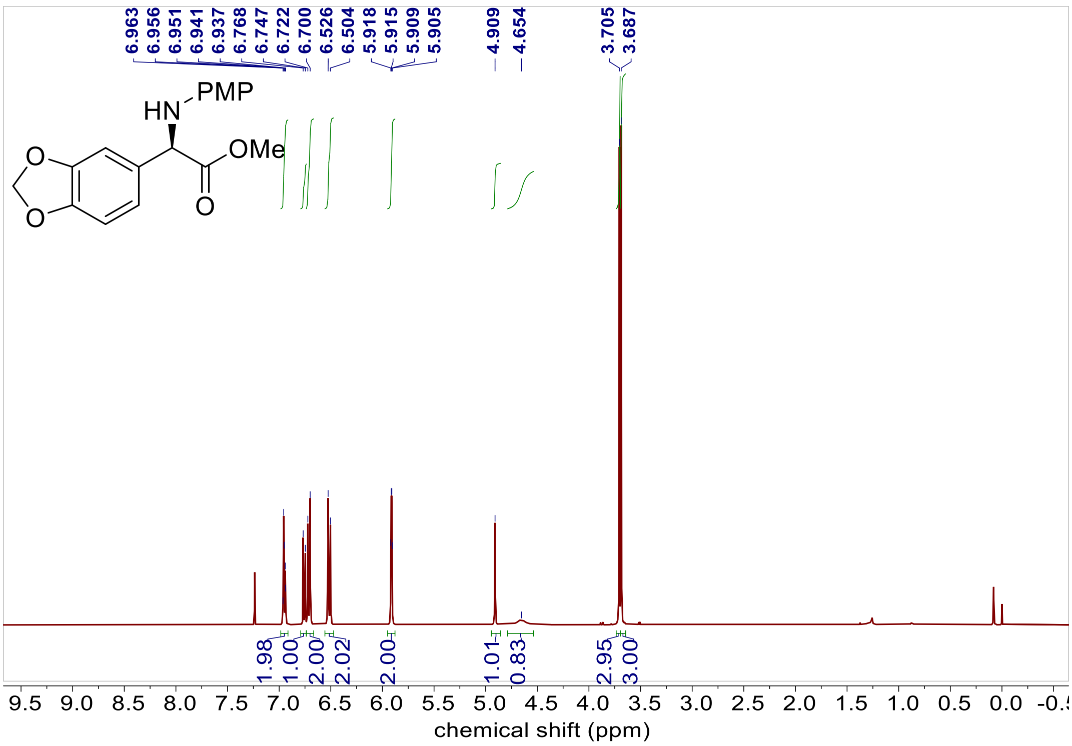

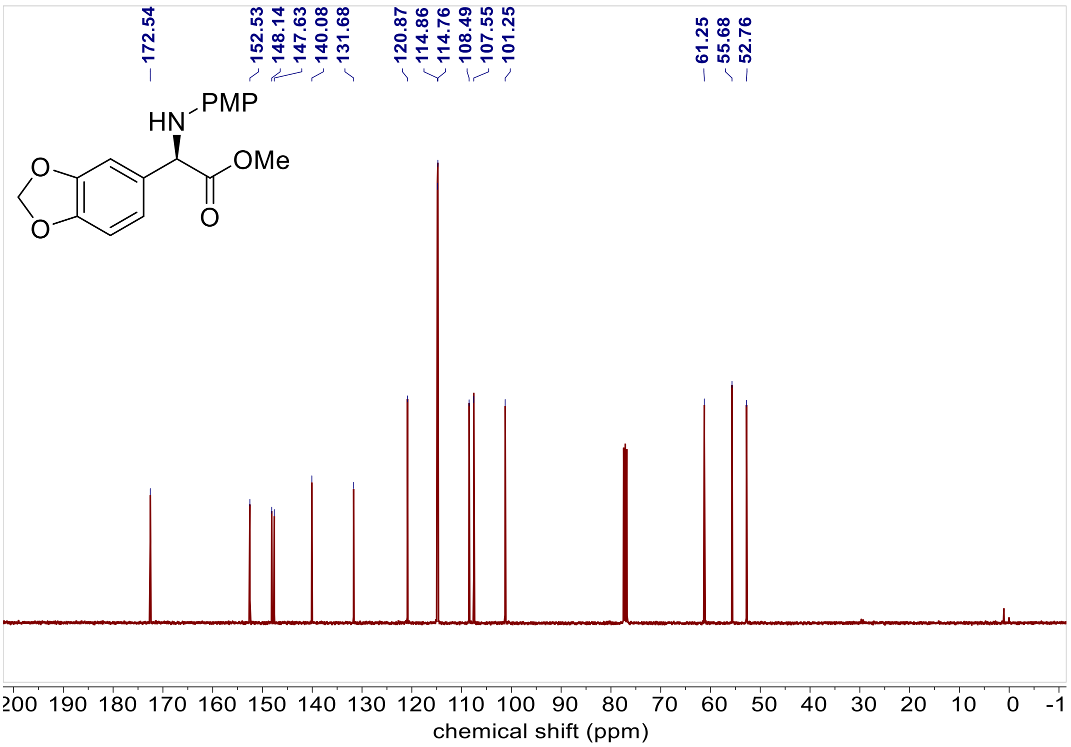

Supplementary Figure 51. ^{1}H NMR & ^{13}C NMR spectra of compound **2z** in $CDCl_3$

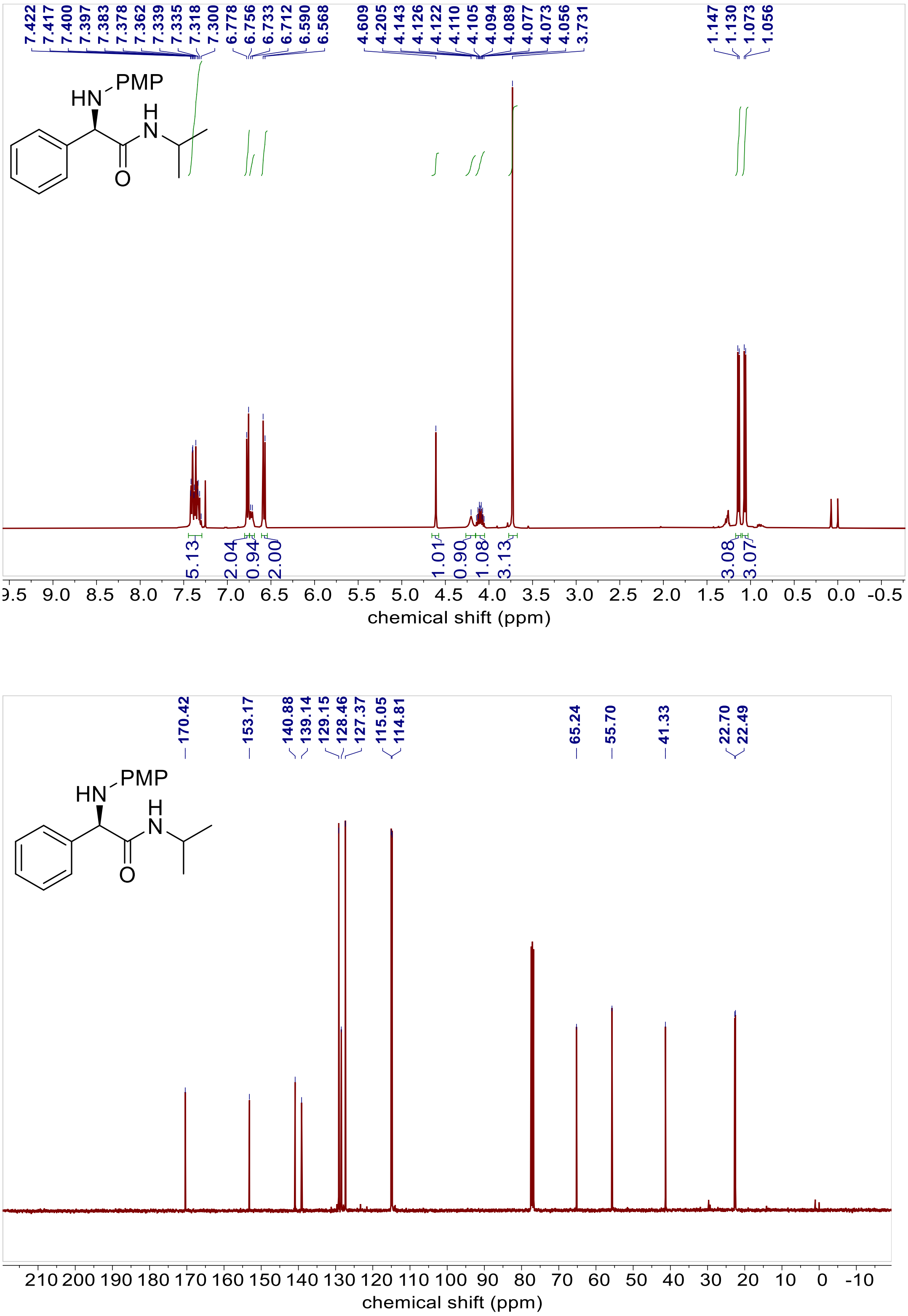

Supplementary Figure 52. ^{1}H NMR & ^{13}C NMR spectra of compound **2af** in $CDCl_3$

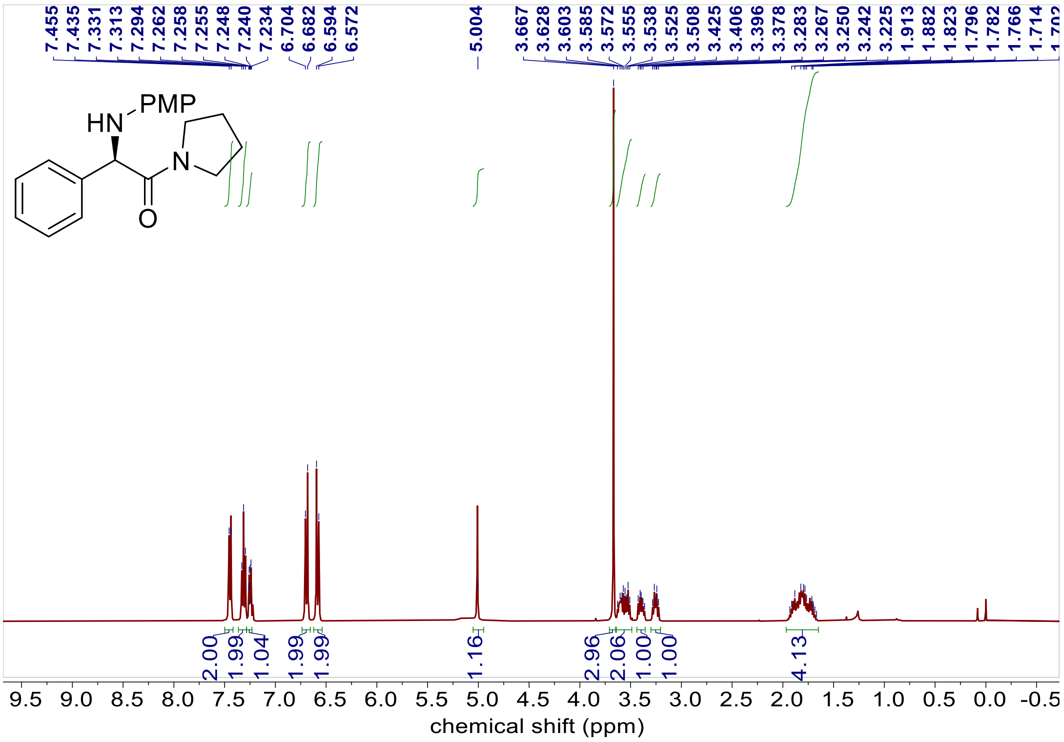

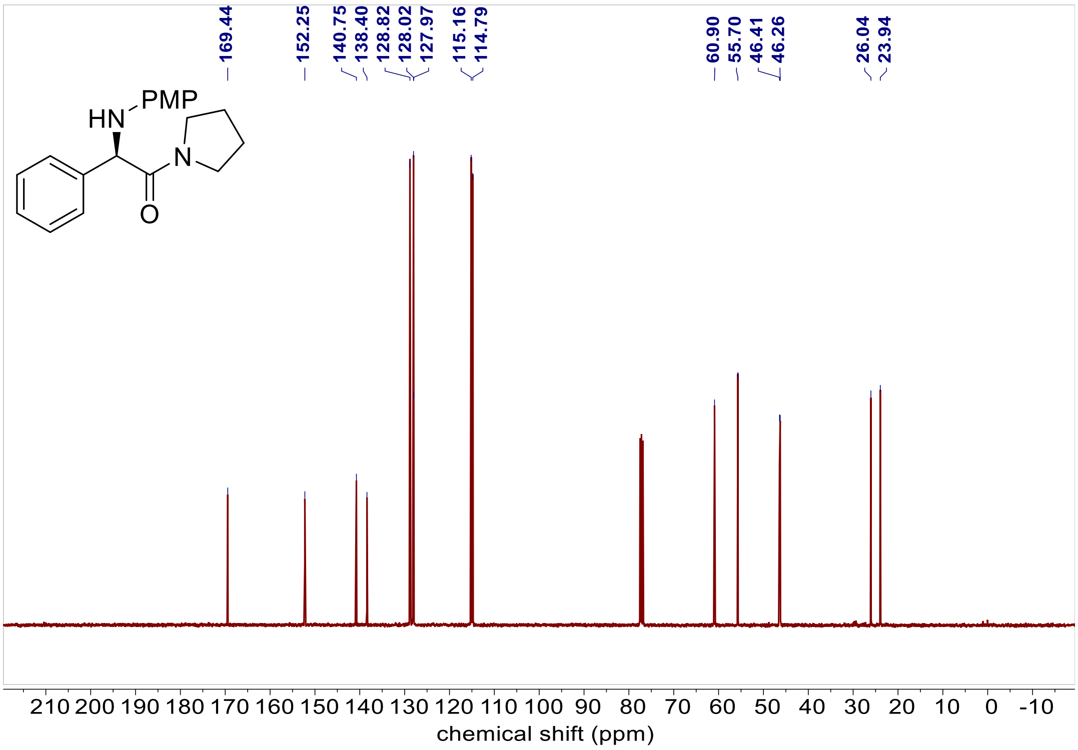

Supplementary Figure 53. ^{1}H NMR & ^{13}C NMR spectra of compound **2ag** in $CDCl_3$

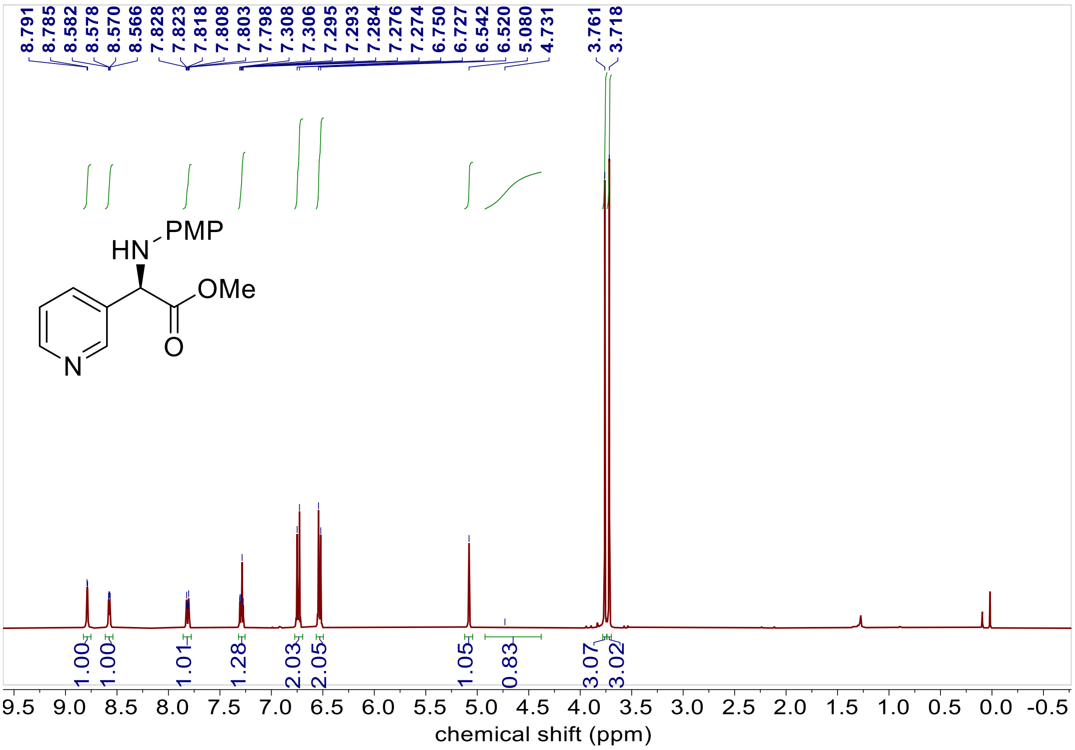

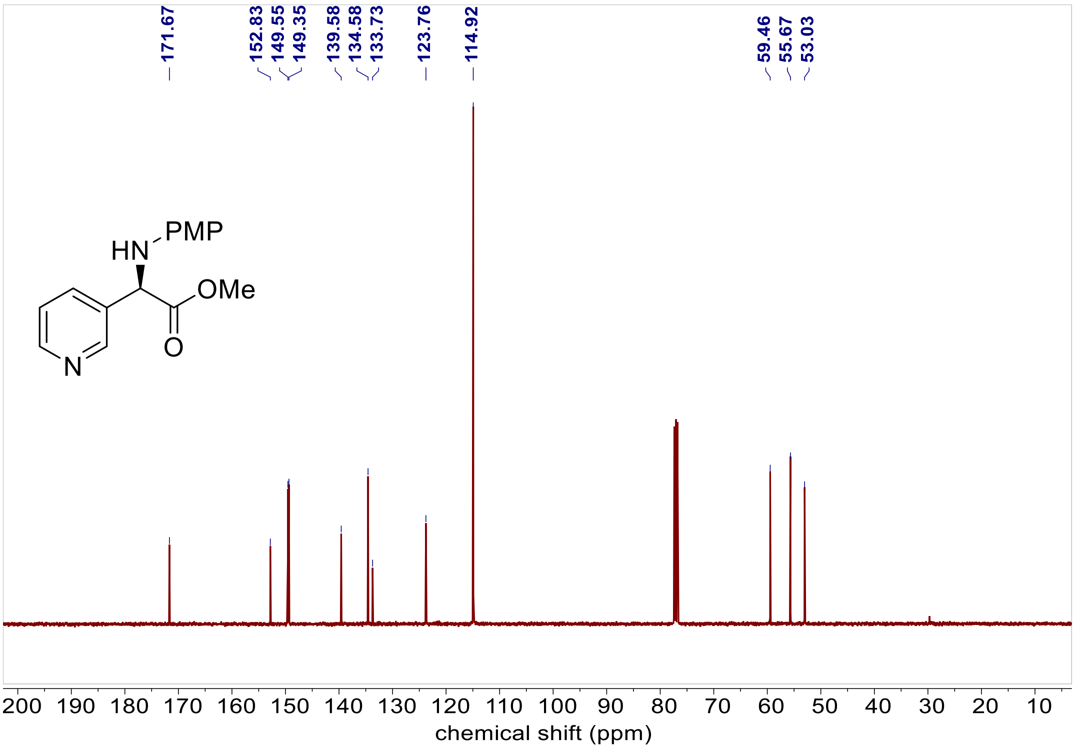

Supplementary Figure 54. ^{1}H NMR & ^{13}C NMR spectra of compound **2ah** in $CDCl_3$

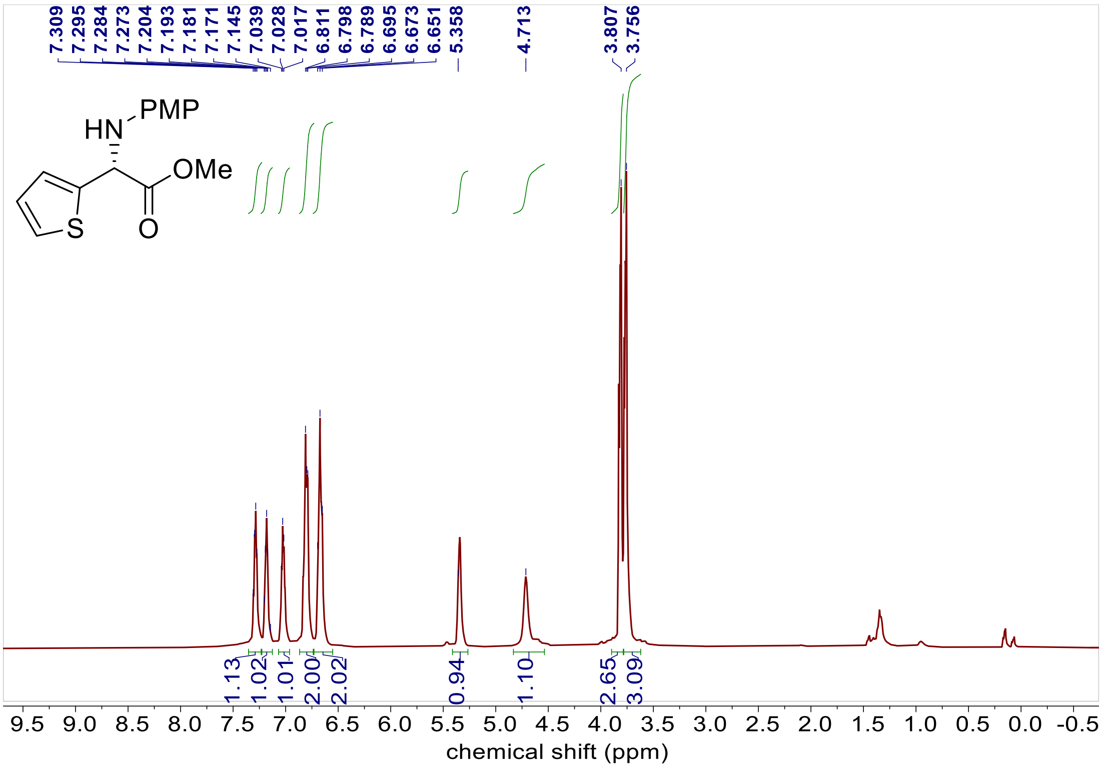

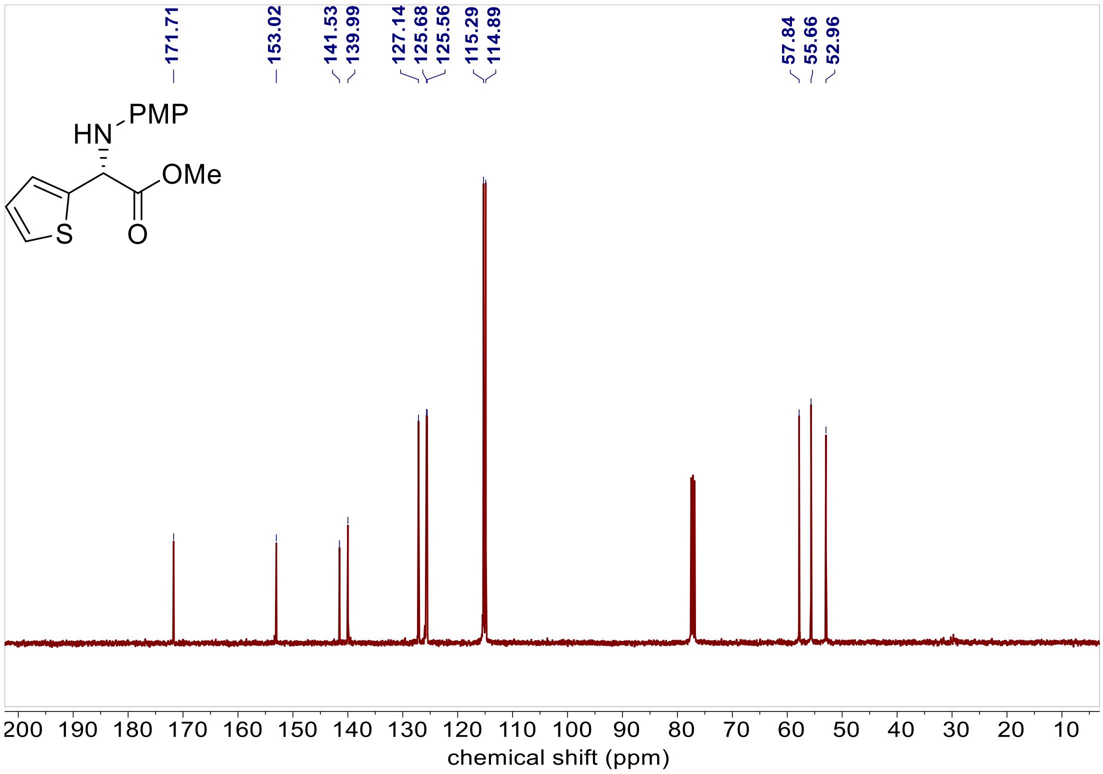

Supplementary Figure 55. ^{1}H NMR & ^{13}C NMR spectra of compound **2ai** in $CDCl_3$

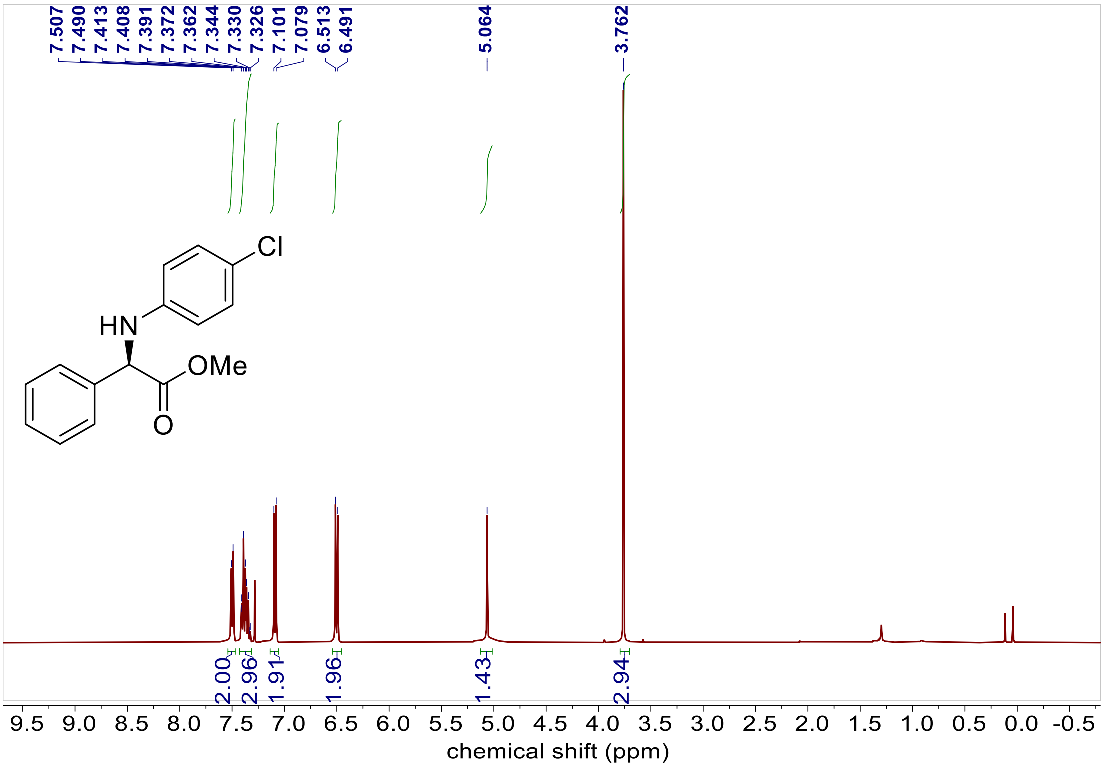

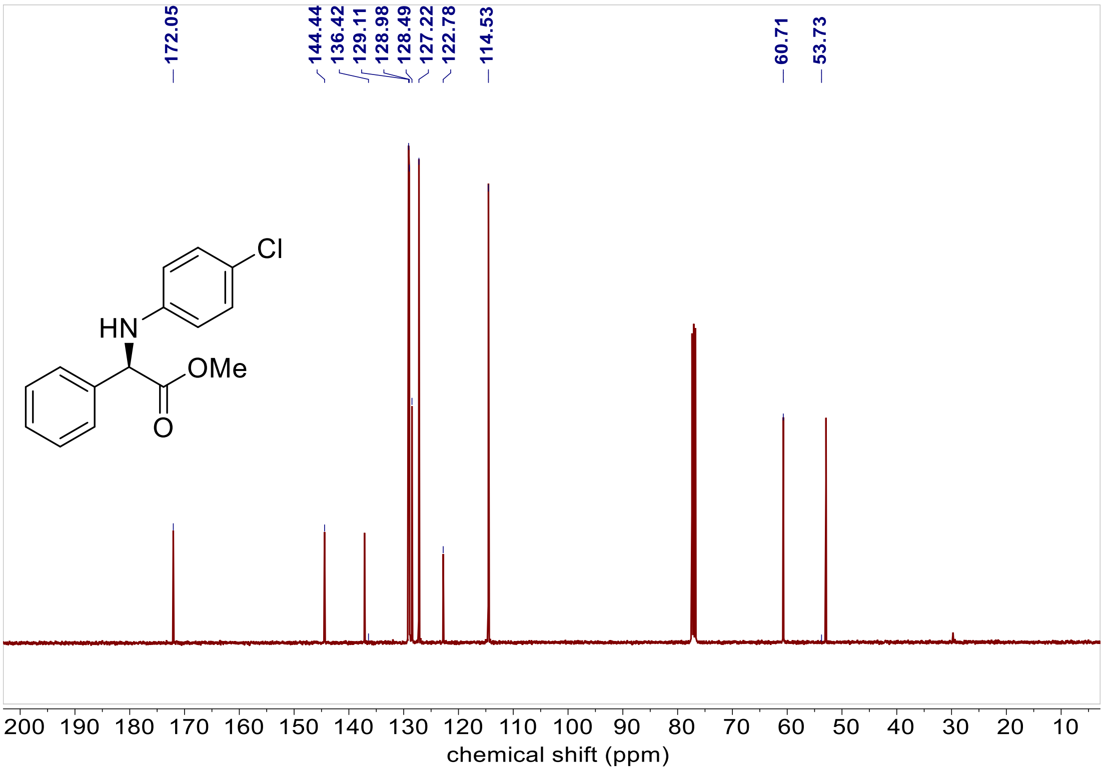

Supplementary Figure 56. ^{1}H NMR & ^{13}C NMR spectra of compound **2ak** in $CDCl_3$

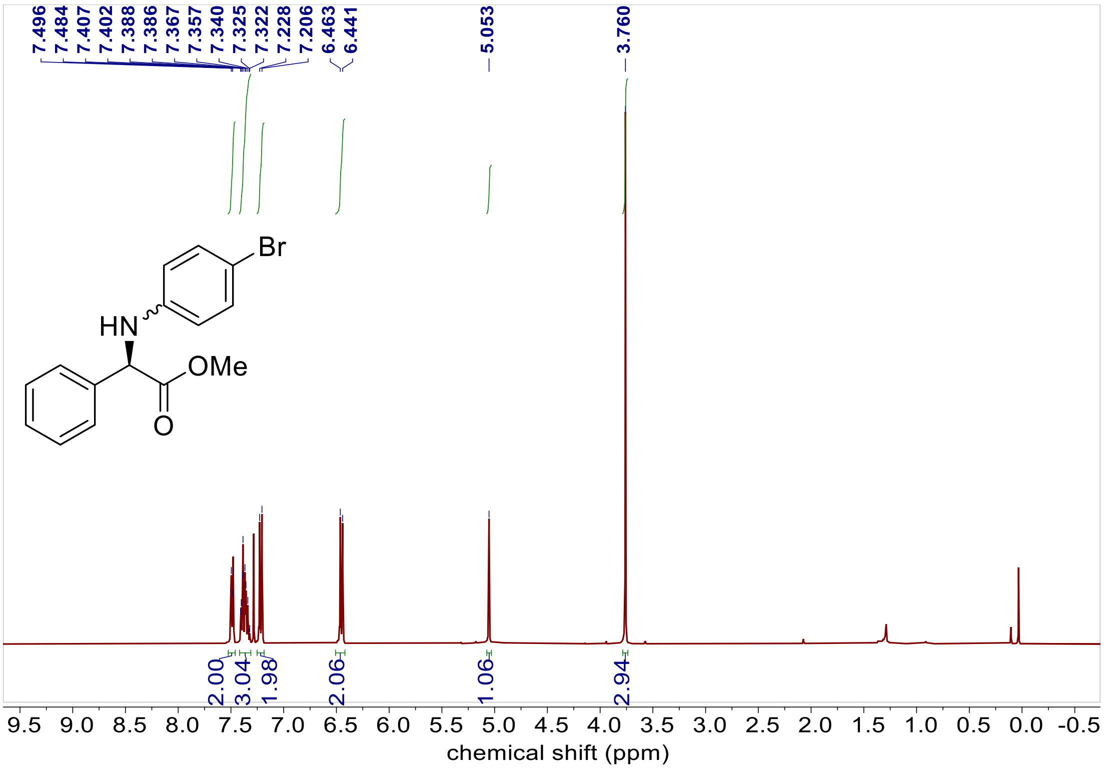

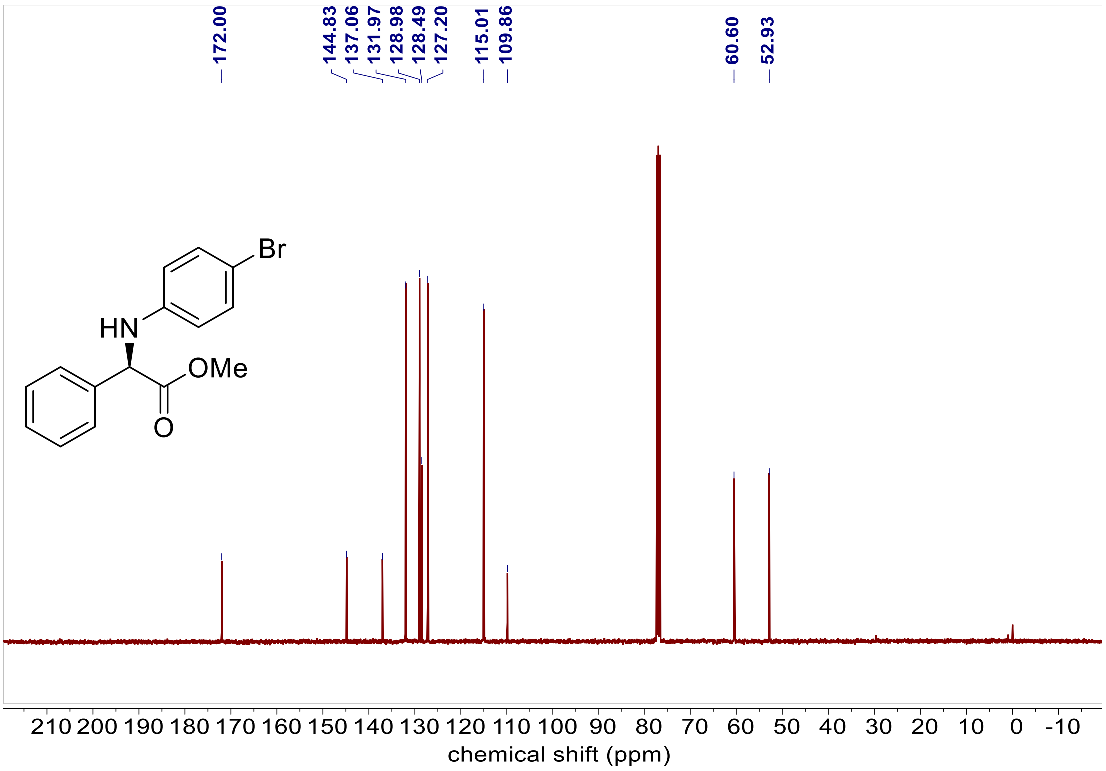

Supplementary Figure 57. ^{1}H NMR & ^{13}C NMR spectra of compound **2al** in $CDCl_3$

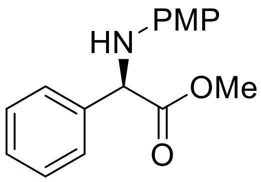

Methyl (*R*)-2-((4-methoxyphenyl)amino)-2-phenylacetate (2a)

HPLC conditions: DAICEL Chiralpak IE column, *n*-Hexane/*i*-PrOH = 95/5, 254 nm, 0.8 mL/min, t_{major} = 20.944 min, t_{minor} = 17.205 min.

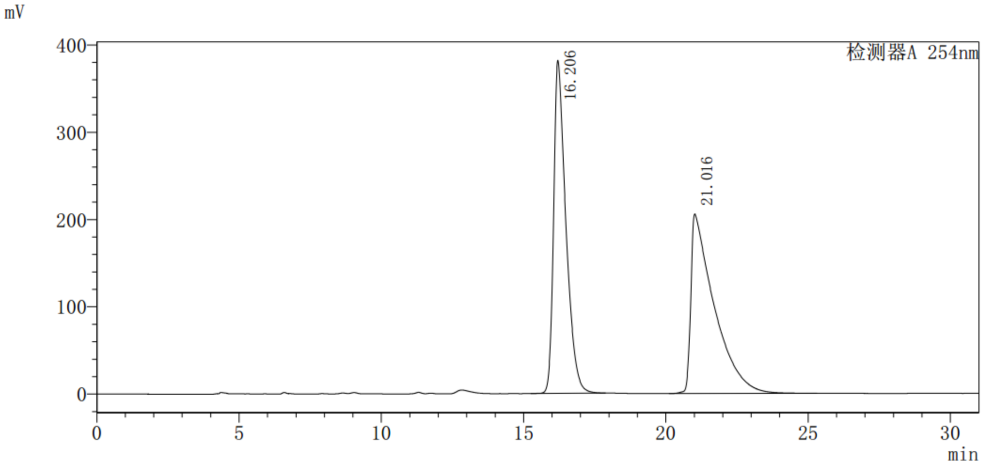

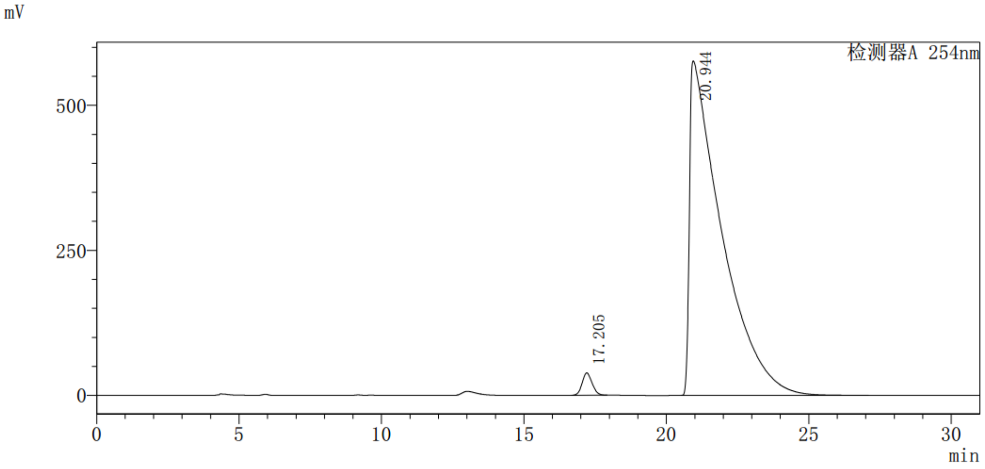

	Retention Time (min)	Relative Area (%)
**Peak 1**	17.205	2.030
**Peak 2**	20.944	97.970

Supplementary Figure 58. HPLC spectra of compound **2a**

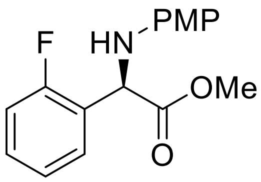

Methyl (*R*)-2-(2-fluorophenyl)-2-((4-methoxyphenyl)amino)acetate (2b)

HPLC conditions: DAICEL Chiralpak IE column, *n*-Hexane/*i*-PrOH = 95/5, 254 nm, 0.8 mL/min, t_{major} = 18.760 min, t_{minor} = 16.437 min.

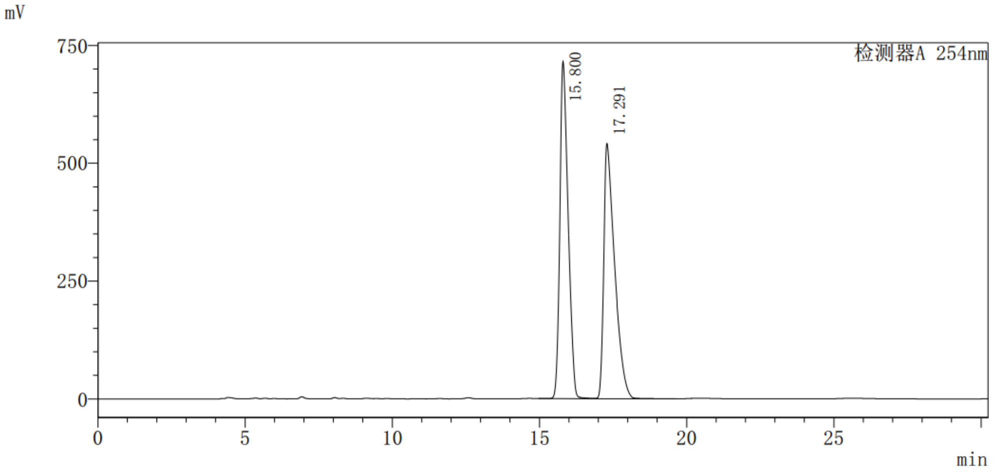

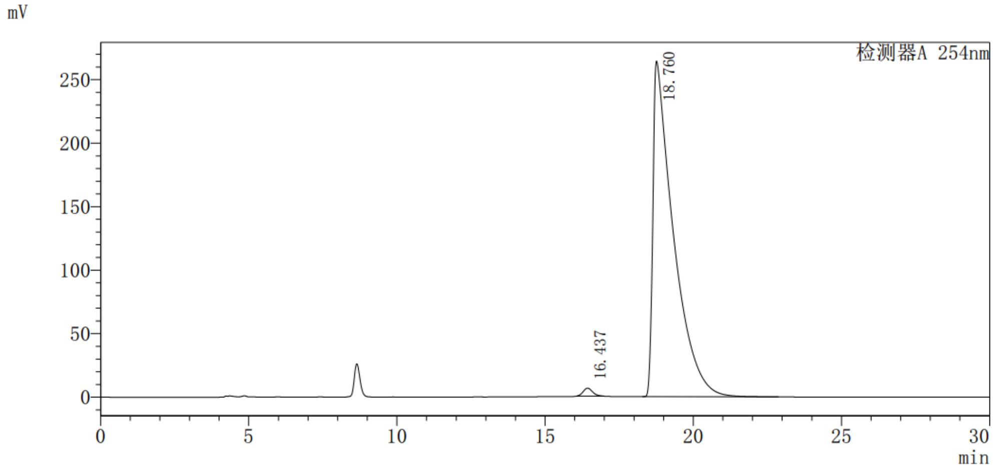

	Retention Time (min)	Relative Area (%)
**Peak 1**	16.437	1.098
**Peak 2**	18.760	98.902

Supplementary Figure 59. HPLC spectra of compound **2b**

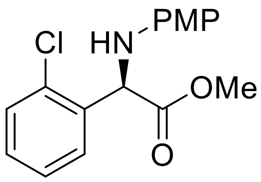

Methyl (*R*)-2-(2-chlorophenyl)-2-((4-methoxyphenyl)amino)acetate (2c)

HPLC conditions: DAICEL Chiralpak IE column, *n*-Hexane/*i*-PrOH = 95/5, 254 nm, 0.8 mL/min, t_{major} = 17.356 min, t_{minor} = 13.386 min.

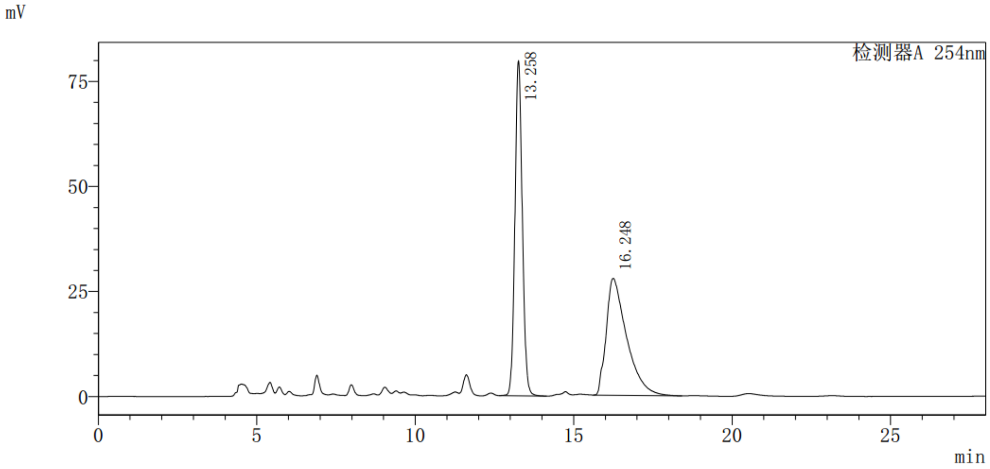

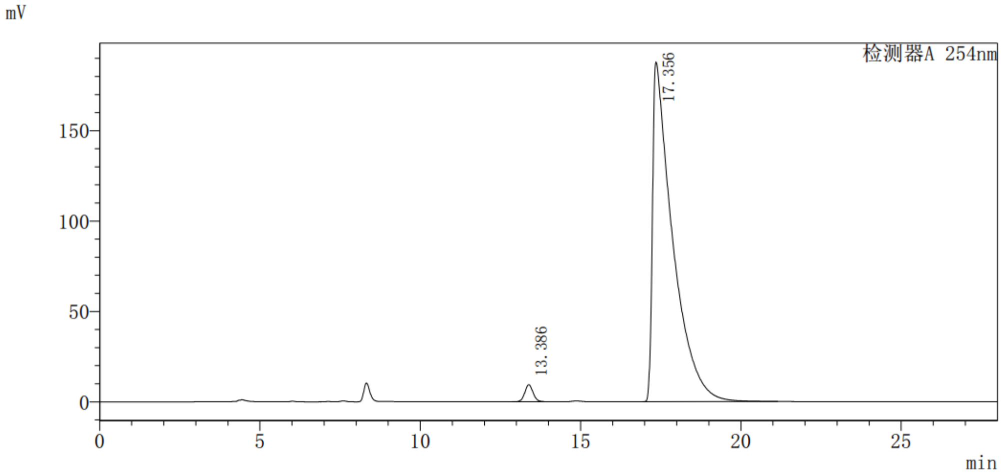

	Retention Time (min)	Relative Area (%)
**Peak 1**	13.386	1.990
**Peak 2**	17.356	98.010

Supplementary Figure 60. HPLC spectra of compound **2c**

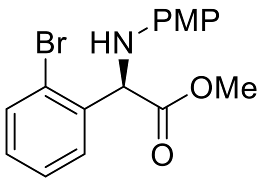

Methyl (*R*)-2-(2-bromophenyl)-2-((4-methoxyphenyl)amino)acetate (2d)

HPLC conditions: DAICEL Chiralpak IE column, *n*-Hexane/*i*-PrOH = 90/10, 254 nm, 0.8 mL/min, t_{major} = 12.954 min, t_{minor} = 10.477 min.

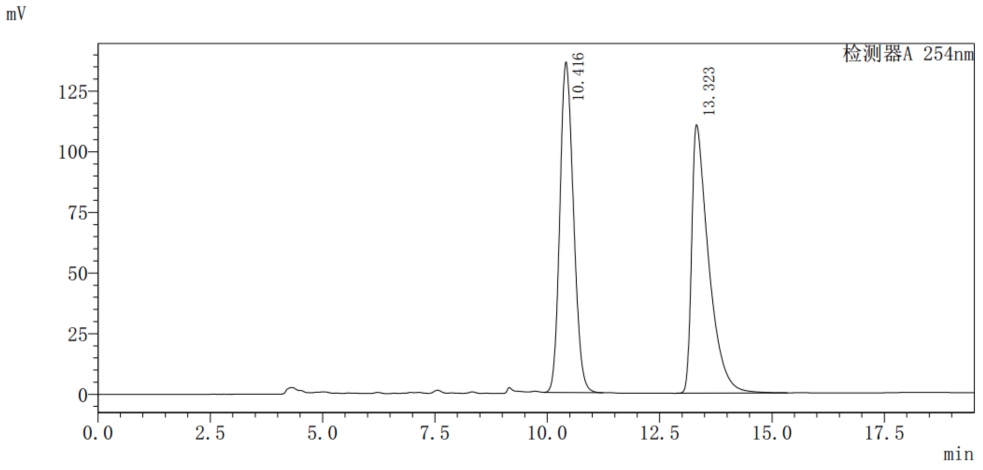

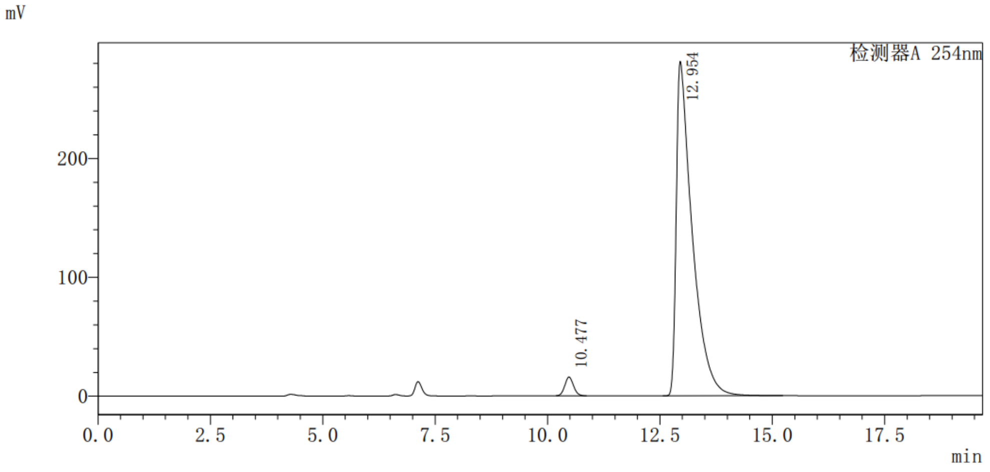

	Retention Time (min)	Relative Area (%)
**Peak 1**	10.477	2.958
**Peak 2**	12.954	97.042

Supplementary Figure 61. HPLC spectra of compound **2d**

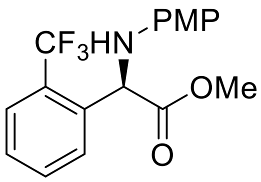

Methyl (*R*)-2-((4-methoxyphenyl)amino)-2-(2-(trifluoromethyl)phenyl)acetate (2e)

HPLC conditions: HPLC conditions: DAICEL Chiralpak IC-3 column, *n*-Hexane/*i*-PrOH = 95/5, 254 nm, 0.8 mL/min, t_{major} = 13.071 min, t_{minor} = 16.722 min.

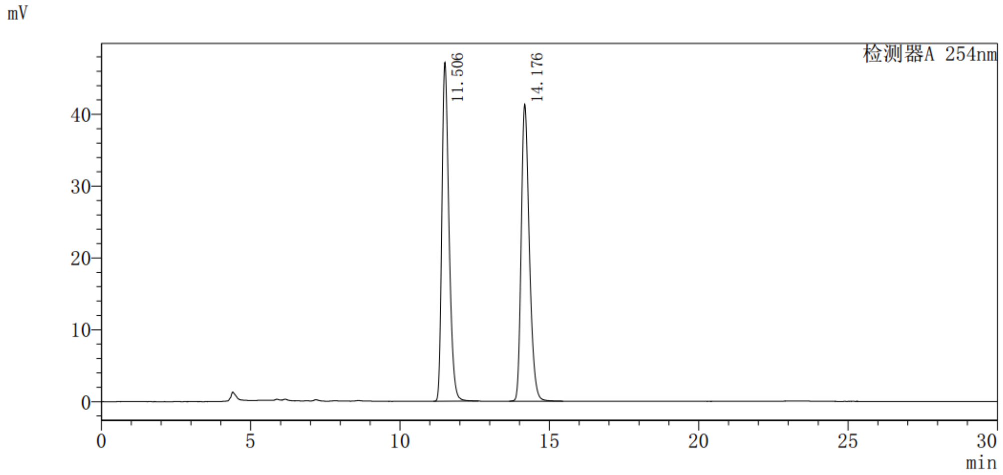

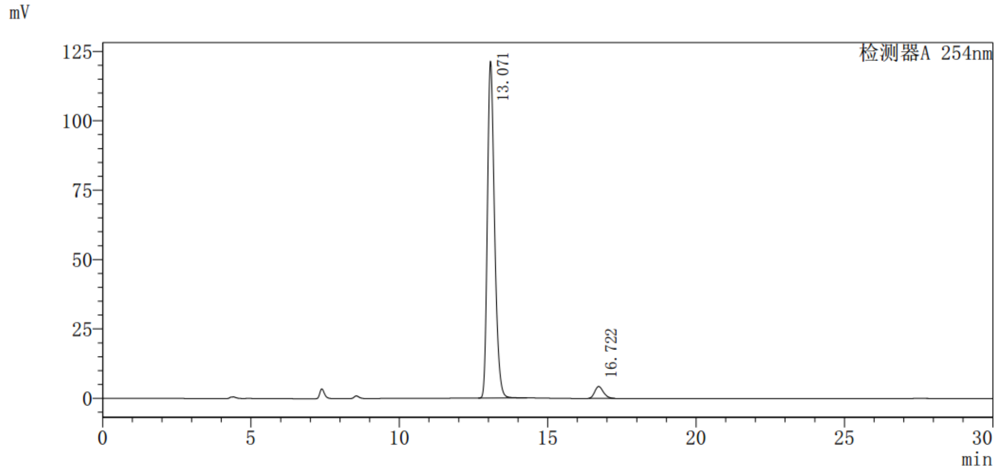

	Retention Time (min)	Relative Area (%)
**Peak 1**	13.071	95.918
**Peak 2**	16.722	4.082

Supplementary Figure 62. HPLC spectra of compound **2e**

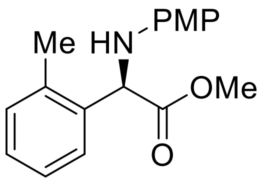

Methyl (*R*)-2-((4-methoxyphenyl)amino)-2-(*o*-tolyl)acetate (2f)

HPLC conditions: DAICEL Chiralpak IE column, *n*-Hexane/*i*-PrOH = 95/5, 254 nm, 0.8 mL/min, t_{major} = 20.640 min, t_{minor} = 15.016 min.

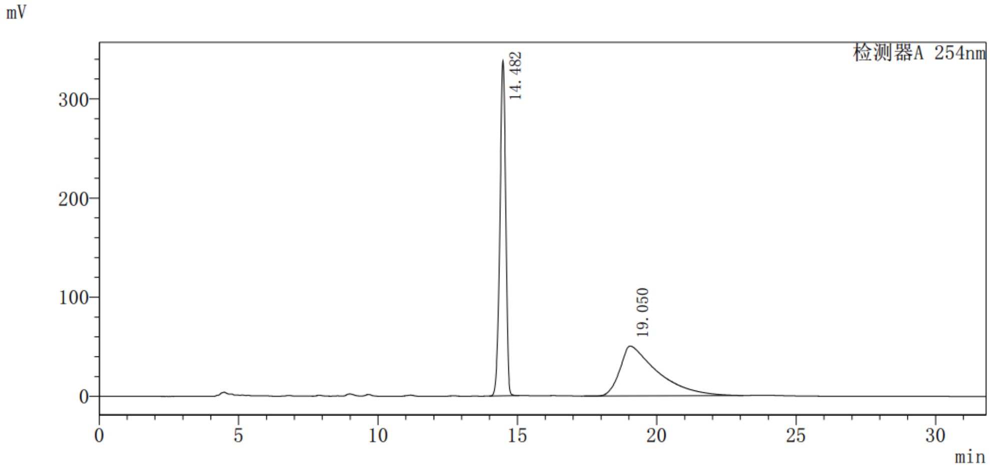

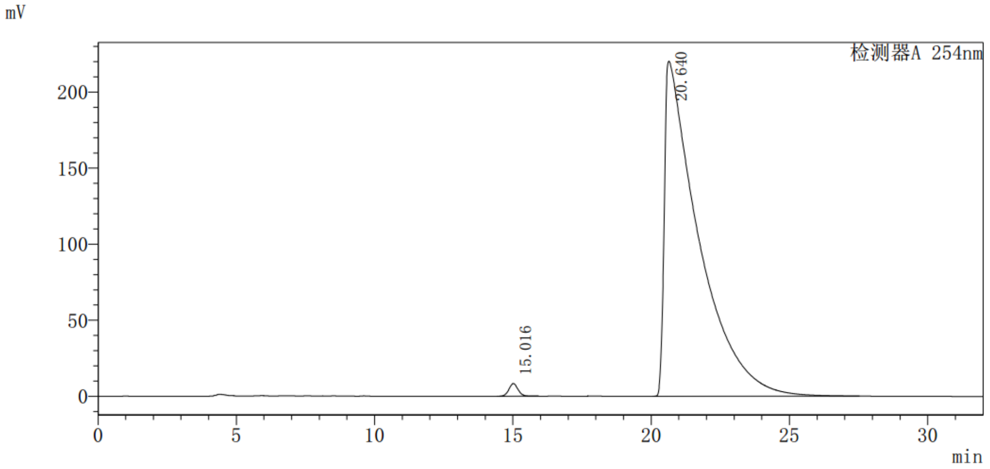

	Retention Time (min)	Relative Area (%)
**Peak 1**	15.016	1.008
**Peak 2**	20.640	98.992

Supplementary Figure 63. HPLC spectra of compound **2f**

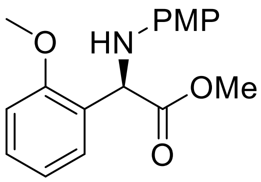

Methyl (*R*)-2-(2-methoxyphenyl)-2-((4-methoxyphenyl)amino)acetate (2g)

HPLC conditions: DAICEL Chiralpak IE column, *n*-Hexane/*i*-PrOH = 95/5, 254 nm, 0.8 mL/min, t_{major} = 32.553 min, t_{minor} = 29.835 min.

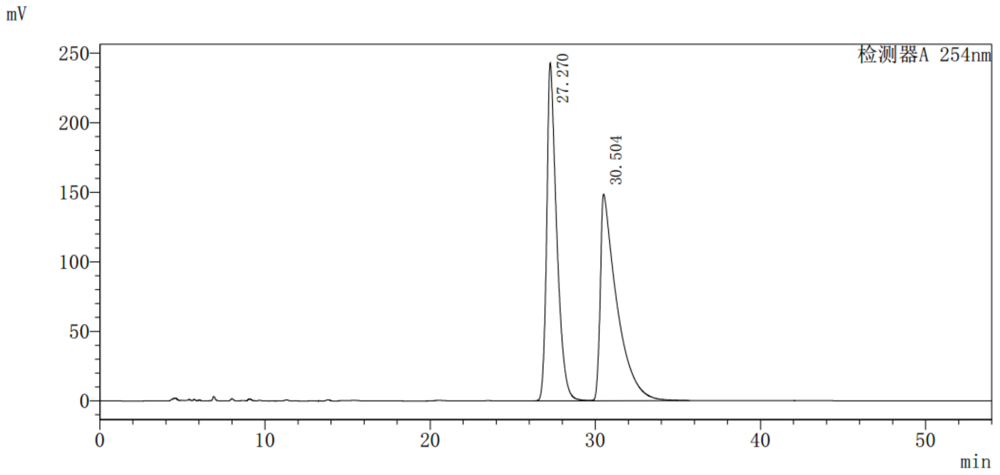

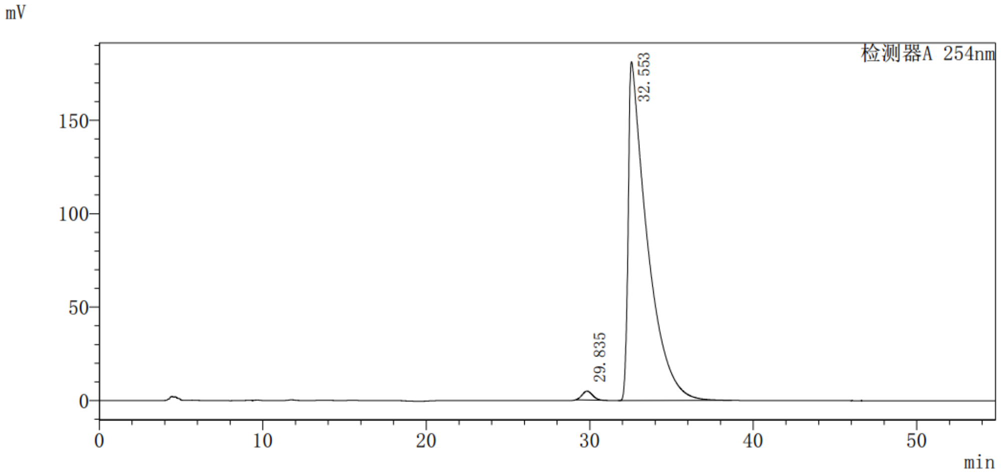

	Retention Time (min)	Relative Area (%)
**Peak 1**	29.835	1.400
**Peak 2**	32.553	98.600

Supplementary Figure 64. HPLC spectra of compound **2g**

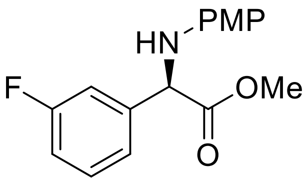

Methyl (*R*)-2-(3-fluorophenyl)-2-((4-methoxyphenyl)amino)acetate (2h)

HPLC conditions: DAICEL Chiralpak IE column, *n*-Hexane/*i*-PrOH = 95/5, 254 nm, 0.8 mL/min, t_{major} = 24.444 min, t_{minor} = 14.223 min.

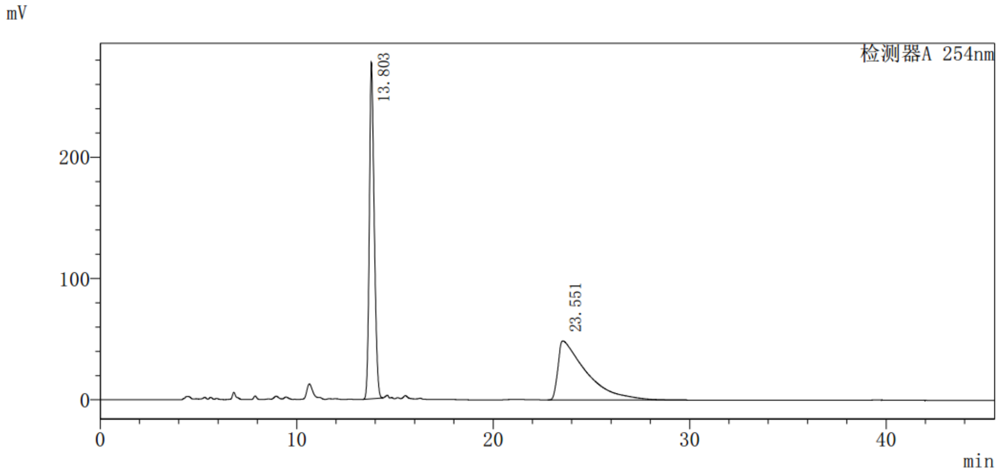

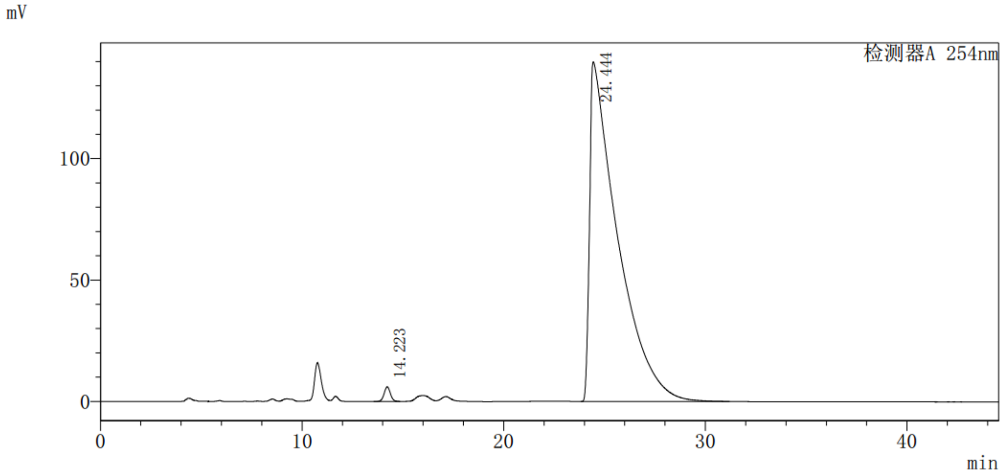

	Retention Time (min)	Relative Area (%)
**Peak 1**	14.223	0.956
**Peak 2**	24.444	99.044

Supplementary Figure 65. HPLC spectra of compound **2h**

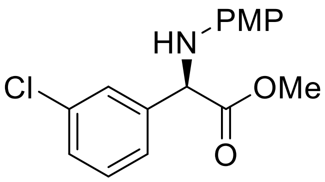

Methyl (*R*)-2-(3-chlorophenyl)-2-((4-methoxyphenyl)amino)acetate (2i)

HPLC conditions: DAICEL Chiralpak IE column, *n*-Hexane/*i*-PrOH = 95/5, 254 nm, 0.8 mL/min, t_{major} = 17.912 min, t_{minor} = 13.519 min.

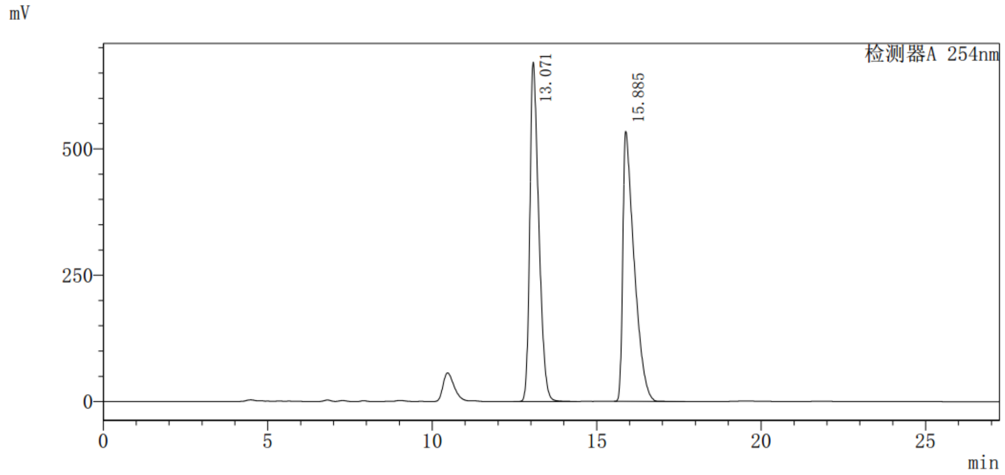

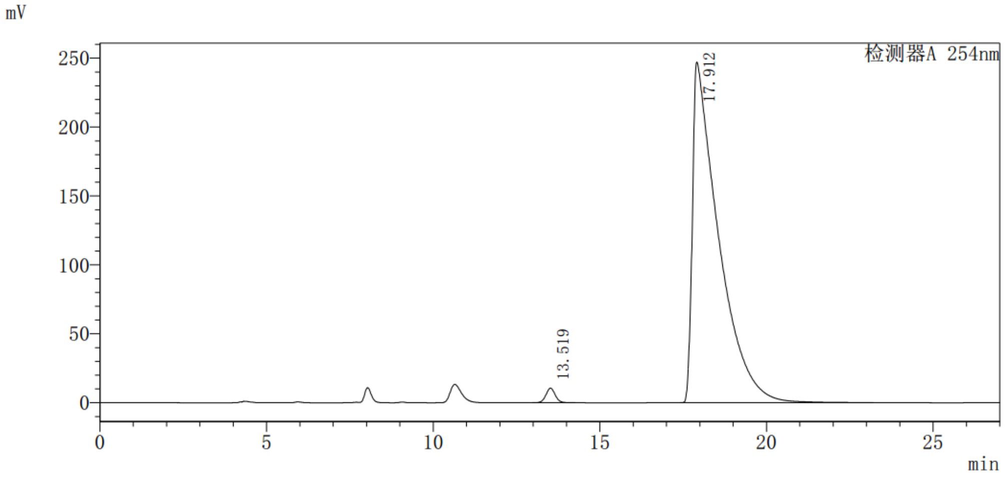

	Retention Time (min)	Relative Area (%)
**Peak 1**	13.519	1.508
**Peak 2**	17.912	98.492

Supplementary Figure 66. HPLC spectra of compound **2i**

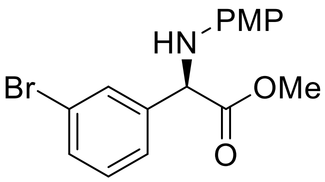

Methyl (*R*)-2-(3-bromophenyl)-2-((4-methoxyphenyl)amino)acetate (2j)

HPLC conditions: DAICEL Chiralpak IE column, *n*-Hexane/*i*-PrOH = 95/5, 254 nm, 0.8 mL/min, t_{major} = 16.794 min, t_{minor} = 13.547 min.

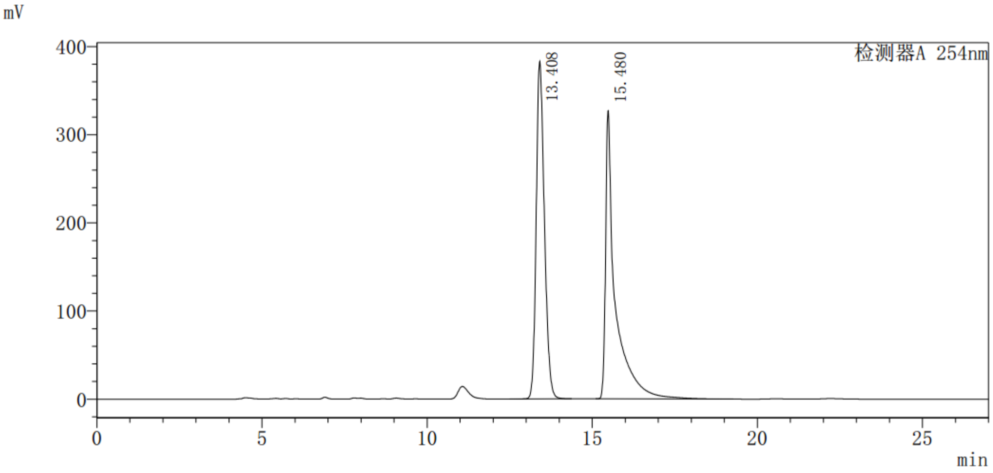

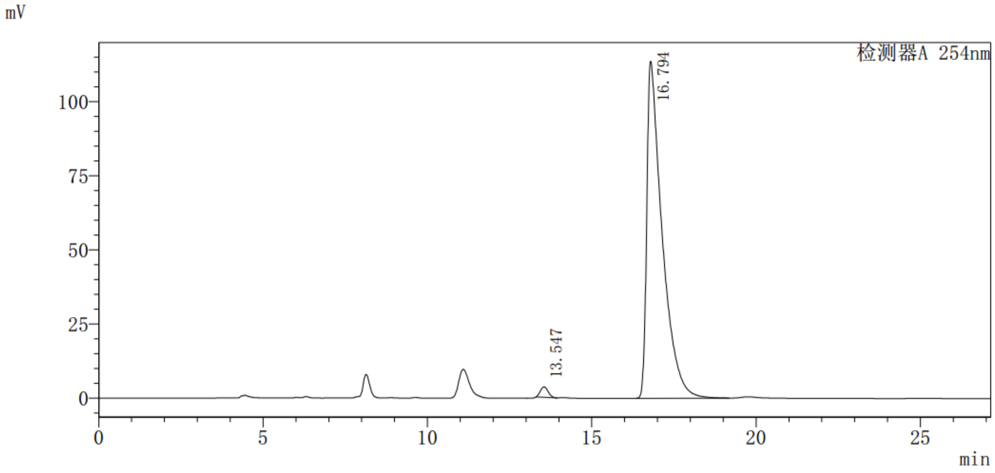

	Retention Time (min)	Relative Area (%)
**Peak 1**	13.547	1.546
**Peak 2**	16.794	98.454

Supplementary Figure 67. HPLC spectra of compound **2j**

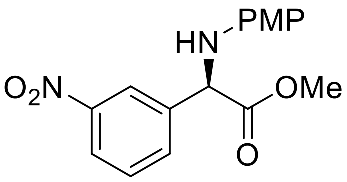

Methyl (*R*)-2-((4-methoxyphenyl)amino)-2-(3-nitrophenyl)acetate (2k)

HPLC conditions: HPLC conditions: DAICEL Chiralpak IE column, n-Hexane/i-PrOH = 90/10, 254 nm, 0.8 mL/min, t_{major} = 39.927 min, t_{minor} = 26.133 min..

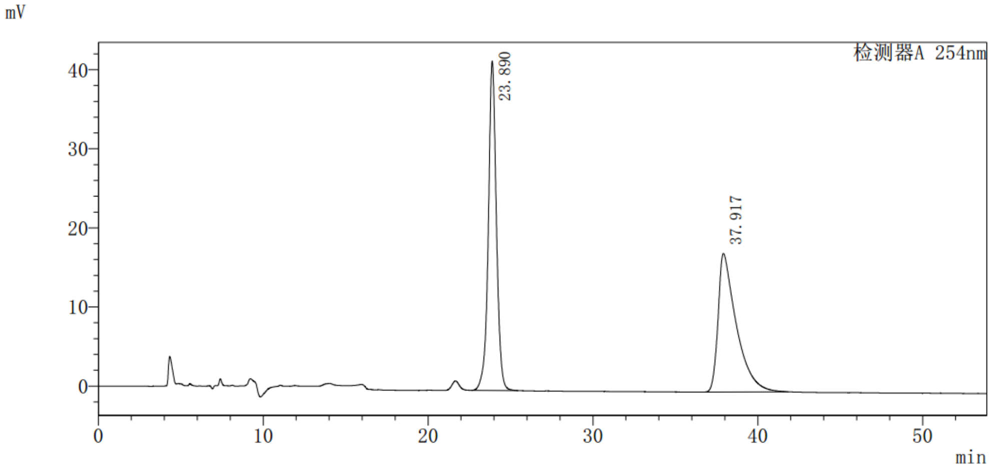

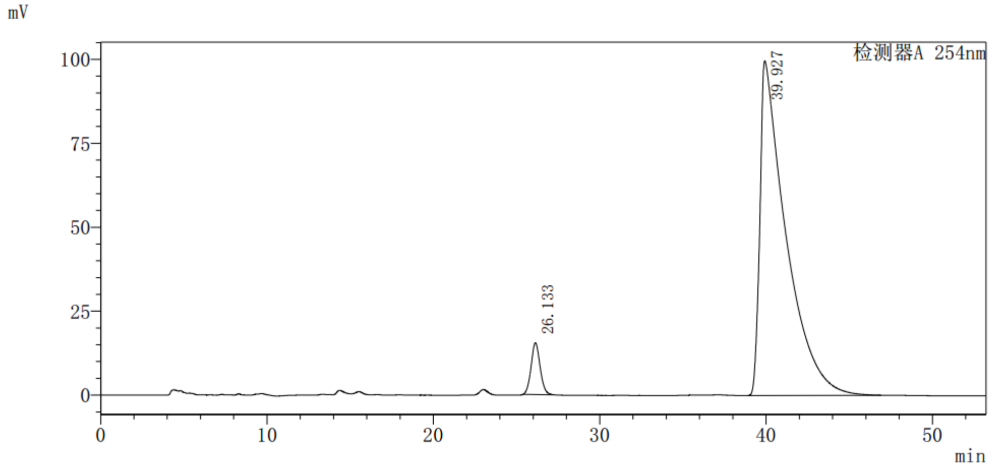

	Retention Time (min)	Relative Area (%)
**Peak 1**	26.133	5.099
**Peak 2**	39.927	94.901

Supplementary Figure 68. HPLC spectra of compound **2k**

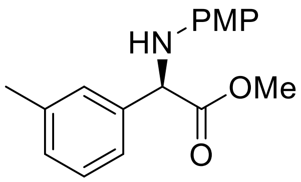

Methyl (*R*)-2-((4-methoxyphenyl)amino)-2-(*m*-tolyl)acetate (2l)

HPLC conditions: DAICEL Chiralpak IE column, *n*-Hexane/*i*-PrOH = 95/5, 254 nm, 0.8 mL/min, t_{major} = 18.768 min, t_{minor} = 14.484 min.

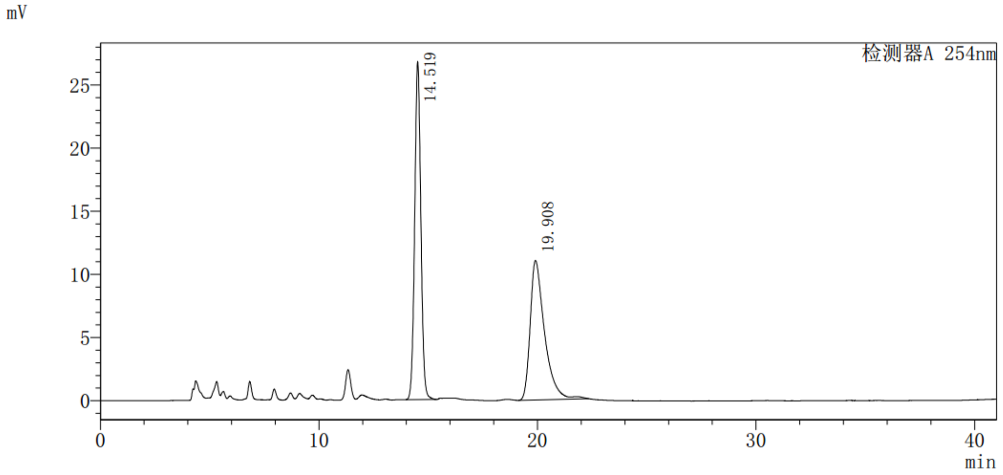

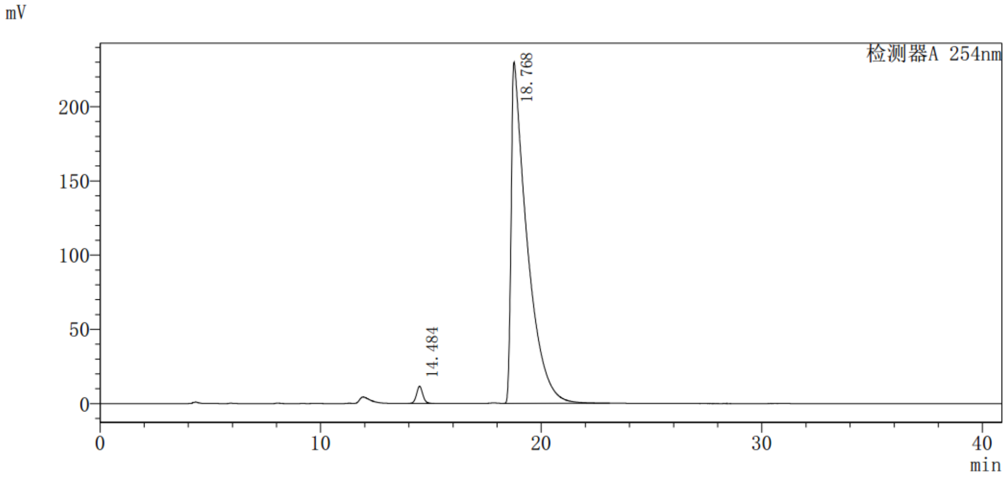

	Retention Time (min)	Relative Area (%)
**Peak 1**	14.484	1.948
**Peak 2**	18.768	98.052

Supplementary Figure 69. HPLC spectra of compound **2l**

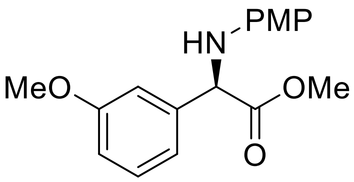

Methyl (*R*)-2-(3-methoxyphenyl)-2-((4-methoxyphenyl)amino)acetate (2m)

HPLC conditions: DAICEL Chiralpak IE column, *n*-Hexane/*i*-PrOH = 90/10, 254 nm, 0.8 mL/min, t_{major} = 28.527 min, t_{minor} = 18.349 min..

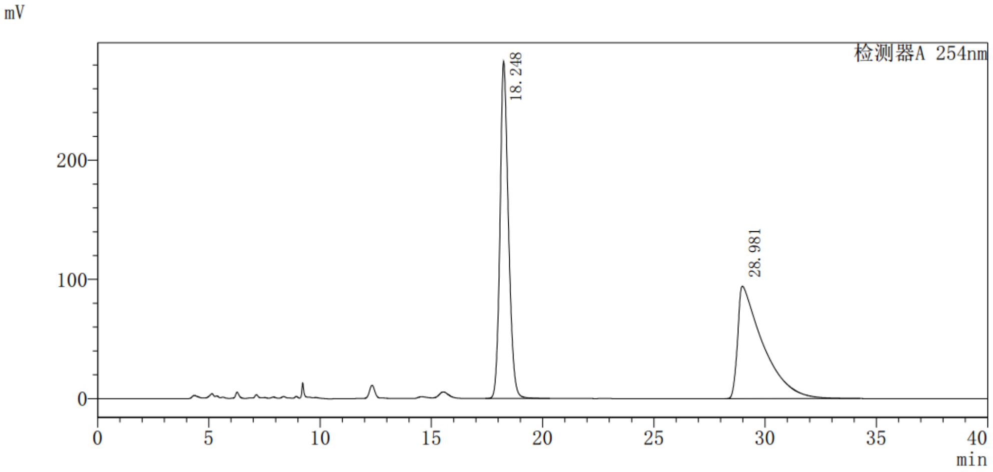

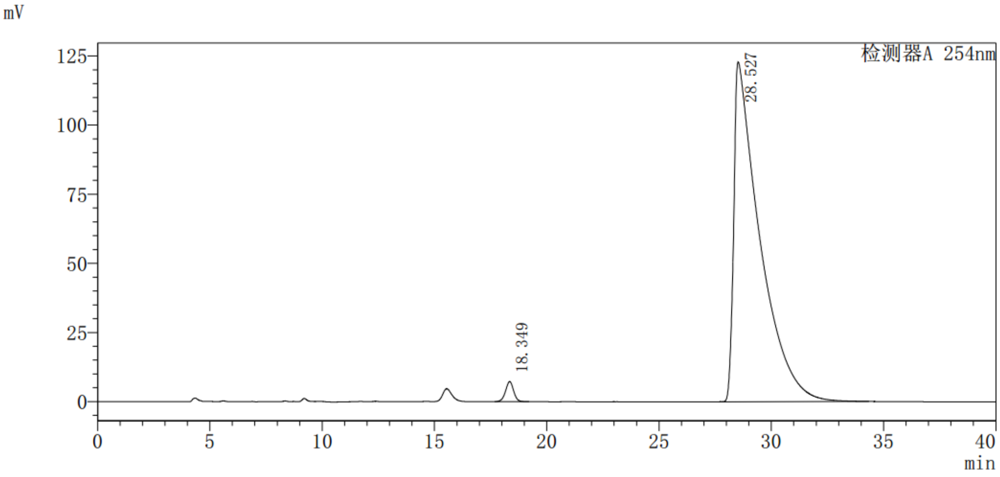

	Retention Time (min)	Relative Area (%)
**Peak 1**	18.349	1.804
**Peak 2**	28.527	98.196

Supplementary Figure 70. HPLC spectra of compound **2m**

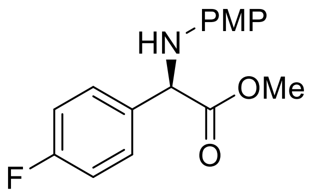

Methyl (*R*)-2-(4-fluorophenyl)-2-((4-methoxyphenyl)amino)acetate (2n)

HPLC conditions: DAICEL Chiralpak IE column, *n*-Hexane/*i*-PrOH = 95/5, 254 nm, 0.8 mL/min, t_{major} = 17.385 min, t_{minor} = 15.893 min.

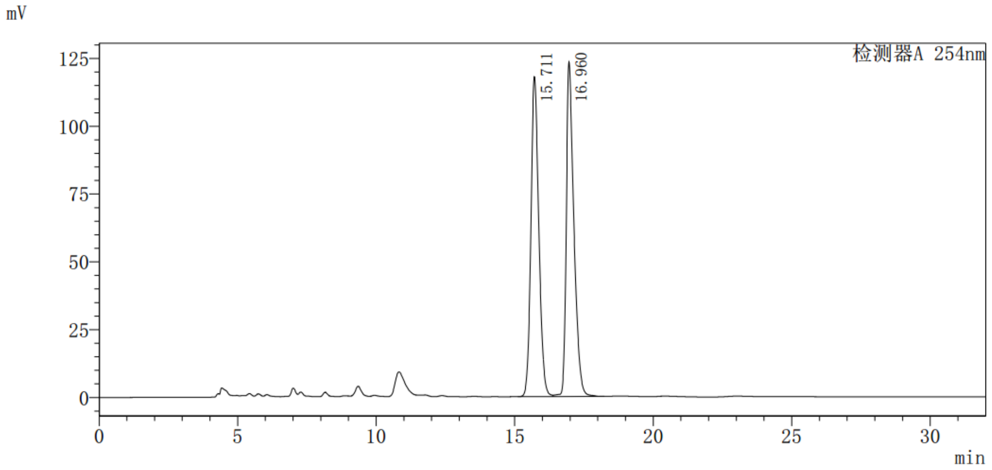

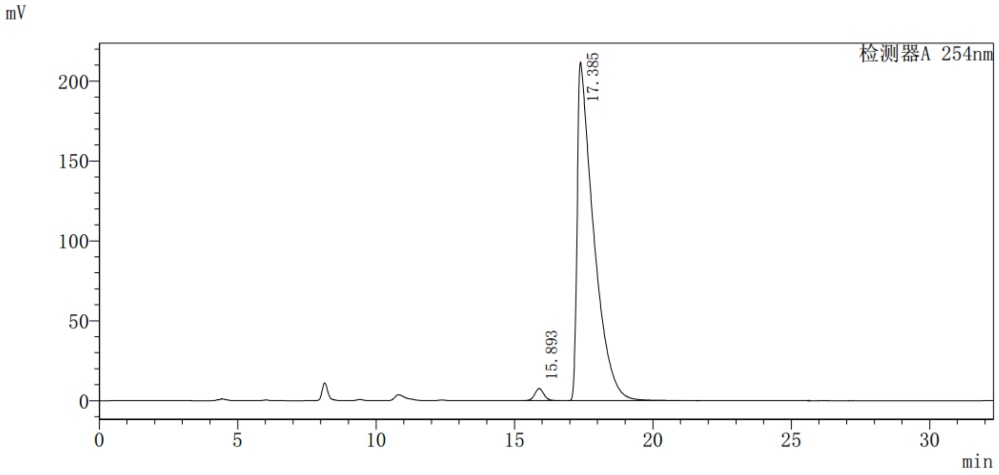

	Retention Time (min)	Relative Area (%)
**Peak 1**	15.893	1.918
**Peak 2**	17.385	98.082

Supplementary Figure 71. HPLC spectra of compound **2n**

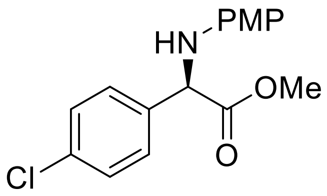

Methyl (*R*)-2-(4-chlorophenyl)-2-((4-methoxyphenyl)amino)acetate (2o)

HPLC conditions: DAICEL Chiralpak IC-3 column, *n*-Hexane/*i*-PrOH = 95/5, 254 nm, 0.8 mL/min, t_{major} = 17.291 min, t_{minor} = 19.911 min.

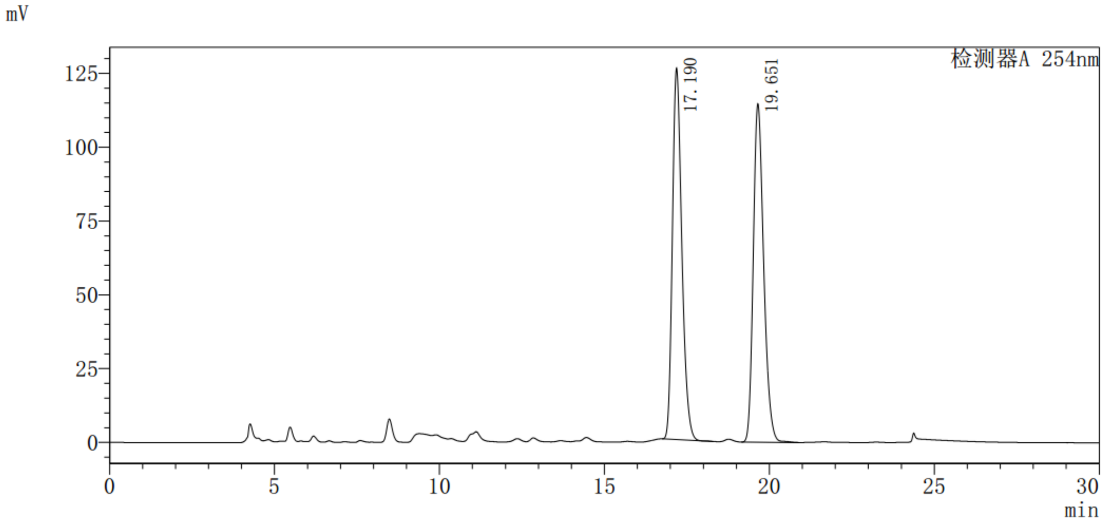

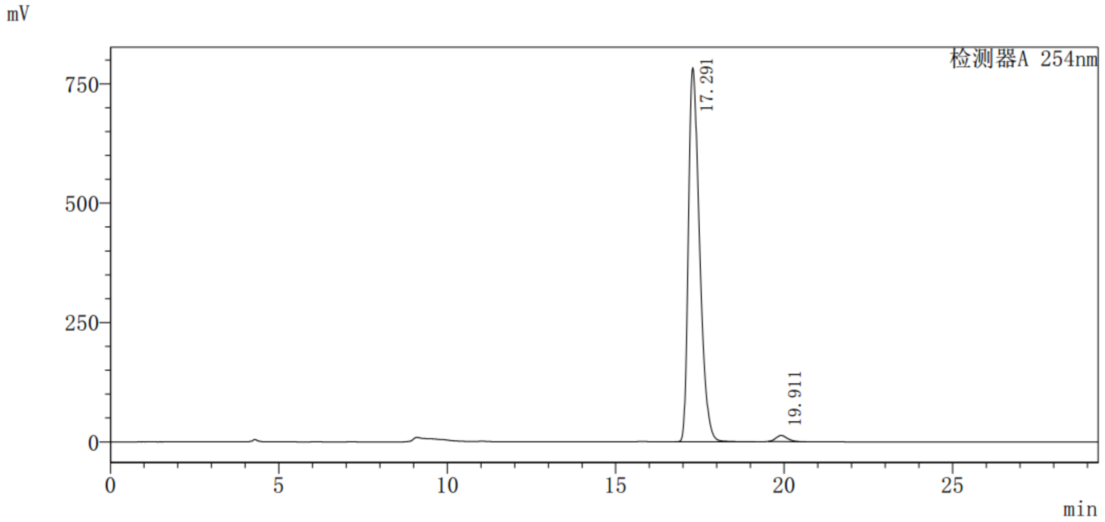

	Retention Time (min)	Relative Area (%)
**Peak 1**	17.291	98.365
**Peak 2**	19.911	1.635

Supplementary Figure 72. HPLC spectra of compound **2o**

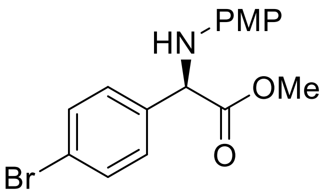

Methyl (*R*)-2-(4-bromophenyl)-2-((4-methoxyphenyl)amino)acetate (2p)

HPLC conditions: DAICEL Chiralpak IC-3 column, *n*-Hexane/*i*-PrOH = 95/5, 254 nm, 0.8 mL/min, t_{major} = 18.308 min, t_{minor} = 20.893 min.

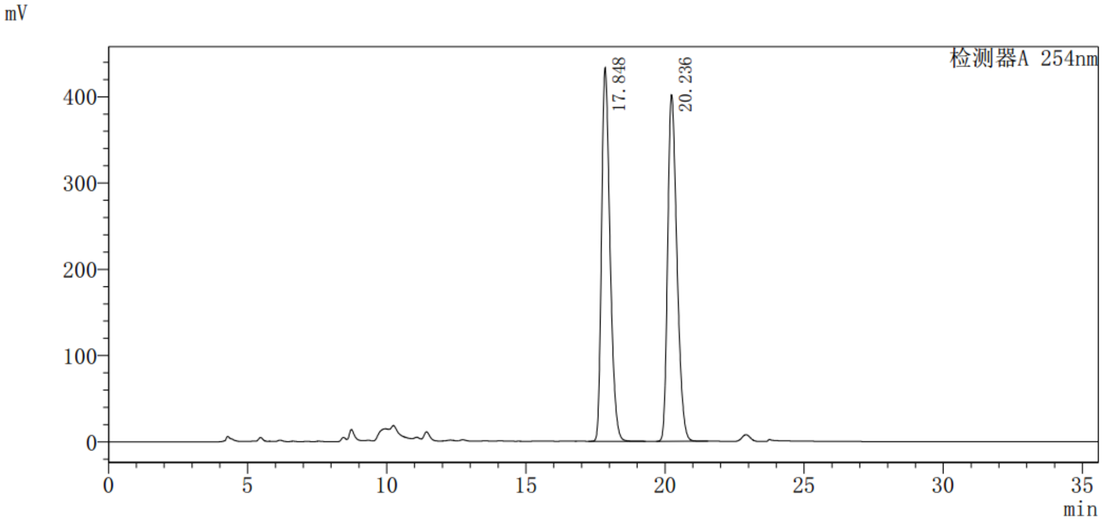

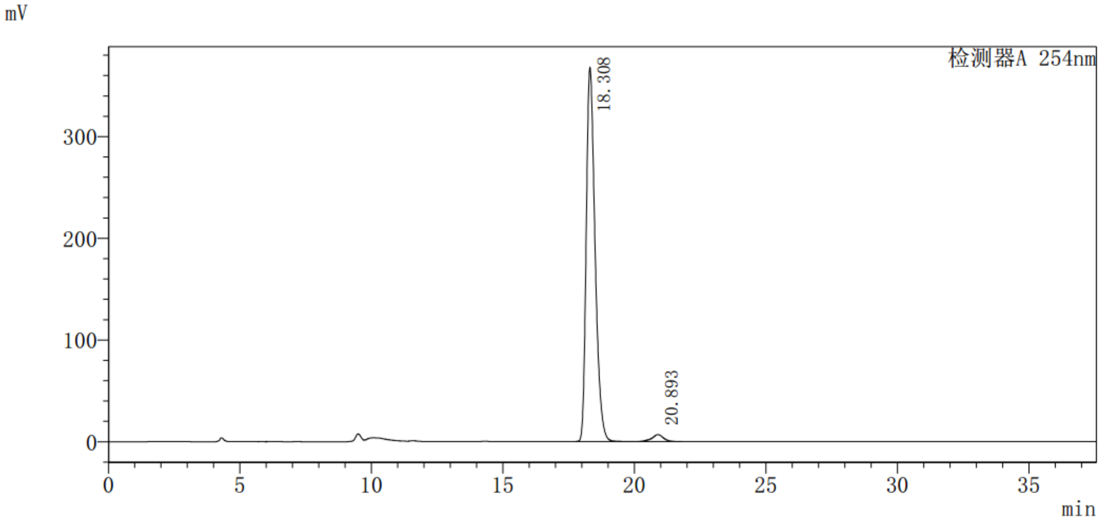

	Retention Time (min)	Relative Area (%)
**Peak 1**	18.308	97.603
**Peak 2**	20.893	2.397

Supplementary Figure 73. HPLC spectra of compound **2p**

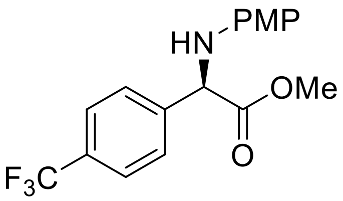

Methyl (*R*)-2-((4-methoxyphenyl)amino)-2-(4-(trifluoromethyl)phenyl)acetate (2q)

HPLC conditions: DAICEL Chiralpak IC-3 column, *n*-Hexane/*i*-PrOH = 95/5, 254 nm, 0.8 mL/min, t_{major} = 12.291 min, t_{minor} = 14.295 min.

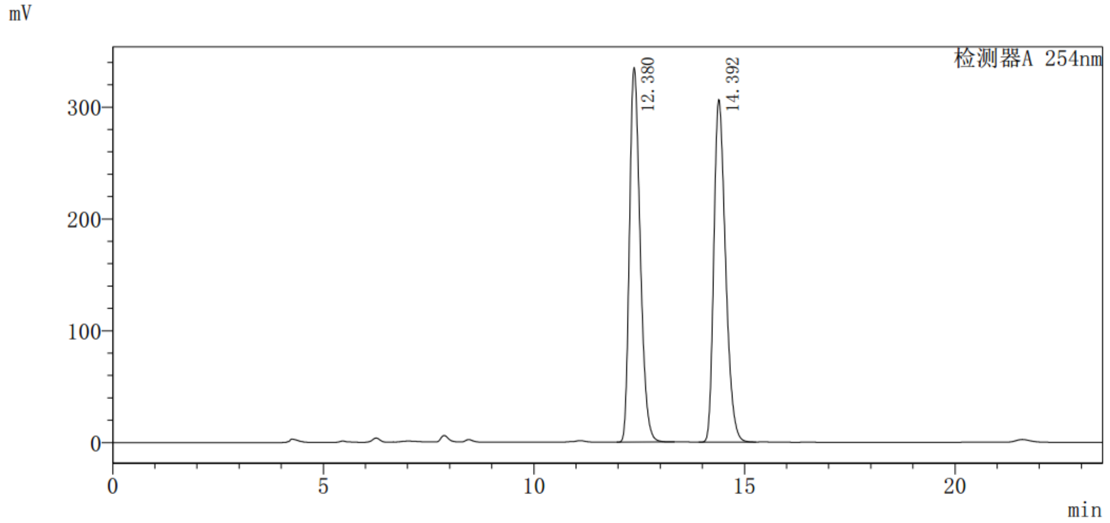

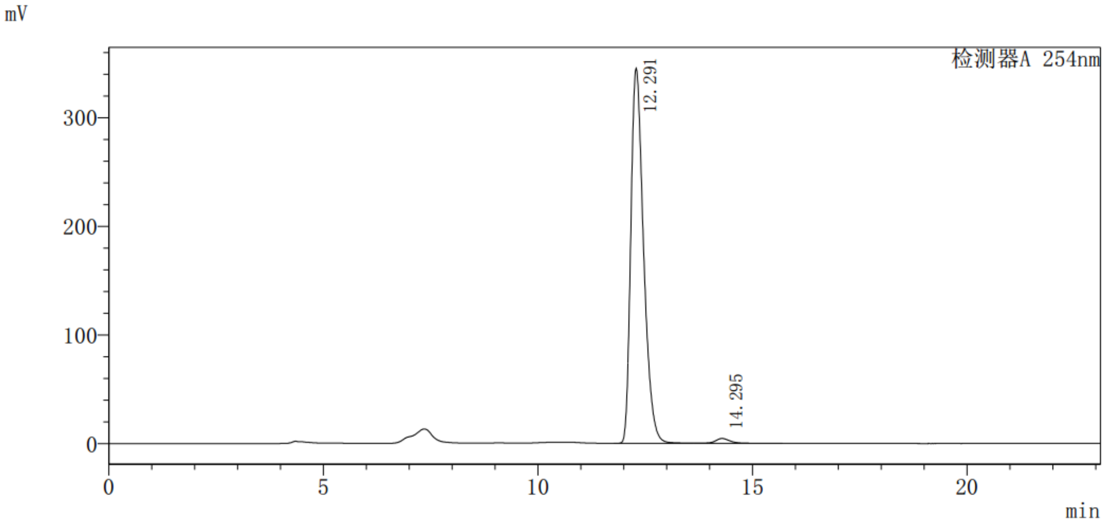

	Retention Time (min)	Relative Area (%)
**Peak 1**	12.291	98.421
**Peak 2**	14.295	1.579

Supplementary Figure 74. HPLC spectra of compound **2q**

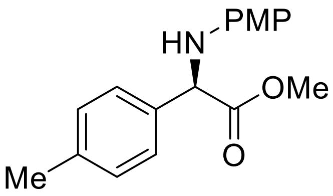

Methyl (*R*)-2-((4-methoxyphenyl)amino)-2-(*p*-tolyl)acetate (2r)

HPLC conditions: DAICEL Chiralpak IE column, *n*-Hexane/*i*-PrOH = 95/5, 254 nm, 0.8 mL/min, t_{major} = 24.215 min, t_{minor} = 19.510 min.

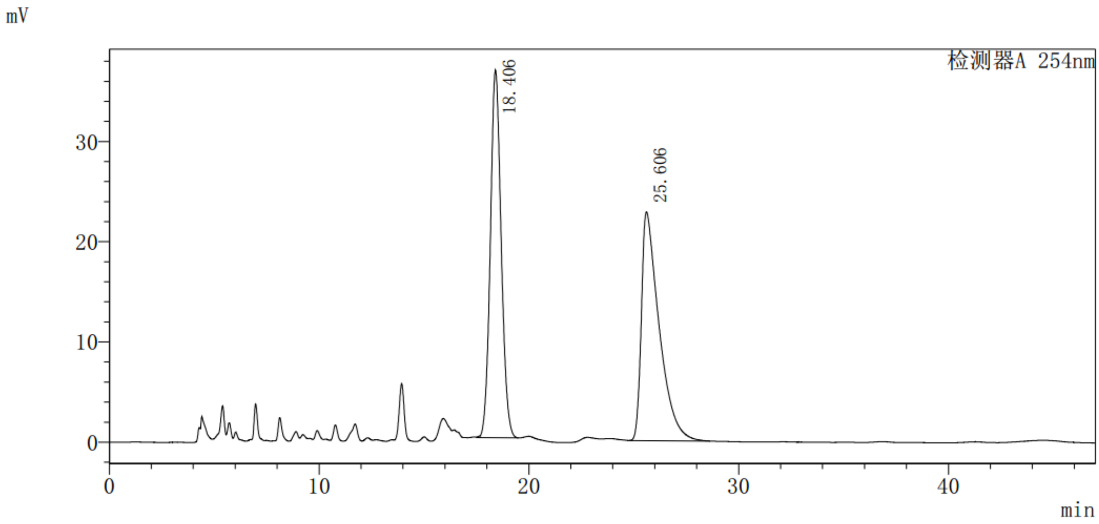

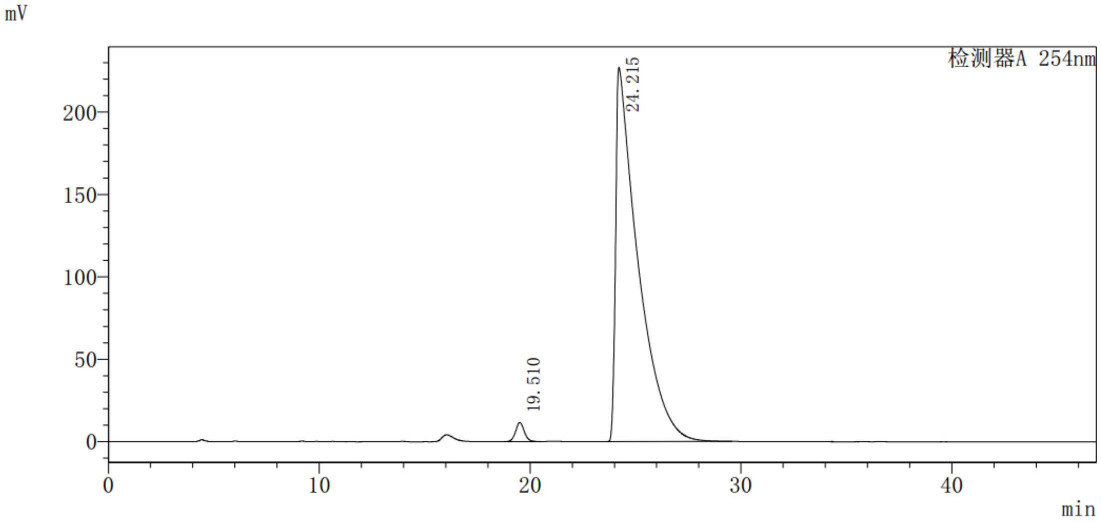

	Retention Time (min)	Relative Area (%)
**Peak 1**	19.510	1.978
**Peak 2**	24.215	98.022

Supplementary Figure 75. HPLC spectra of compound **2r**

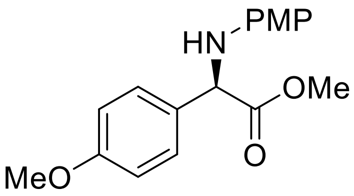

Methyl (*R*)-2-(4-methoxyphenyl)-2-((4-methoxyphenyl)amino)acetate (2s)

HPLC conditions: DAICEL Chiralpak IE column, *n*-Hexane/*i*-PrOH = 90/10, 254 nm, 0.8 mL/min, t_{major} = 32.183 min, t_{minor} = 21.198 min.

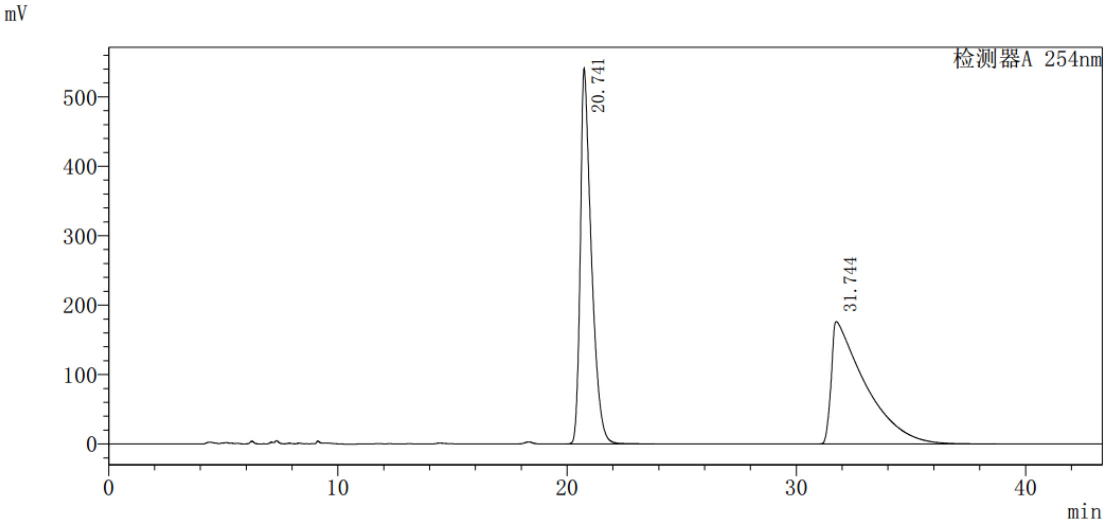

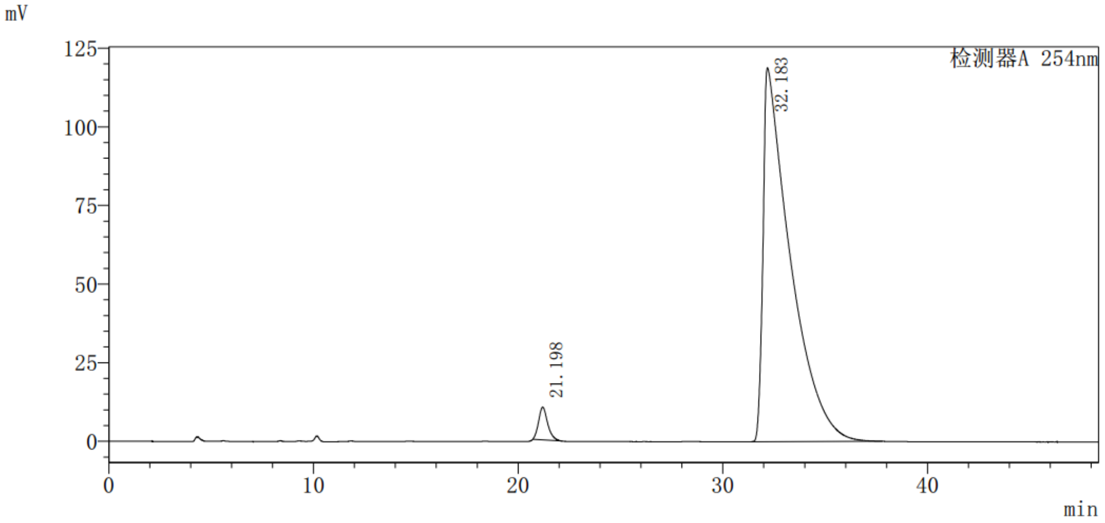

	Retention Time (min)	Relative Area (%)
**Peak 1**	21.198	2.855
**Peak 2**	32.183	97.145

Supplementary Figure 76. HPLC spectra of compound **2s**

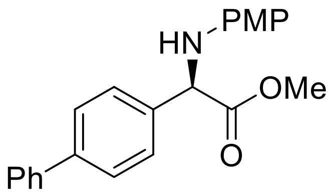

Methyl (*R*)-2-([1,1'-biphenyl]-4-yl)-2-((4-methoxyphenyl)amino)acetate (2t)

HPLC conditions: DAICEL Chiralpak IE column, *n*-Hexane/*i*-PrOH = 90/10, 254 nm, 0.8 mL/min, t_{major} = 35.929 min, t_{minor} = 19.924 min.

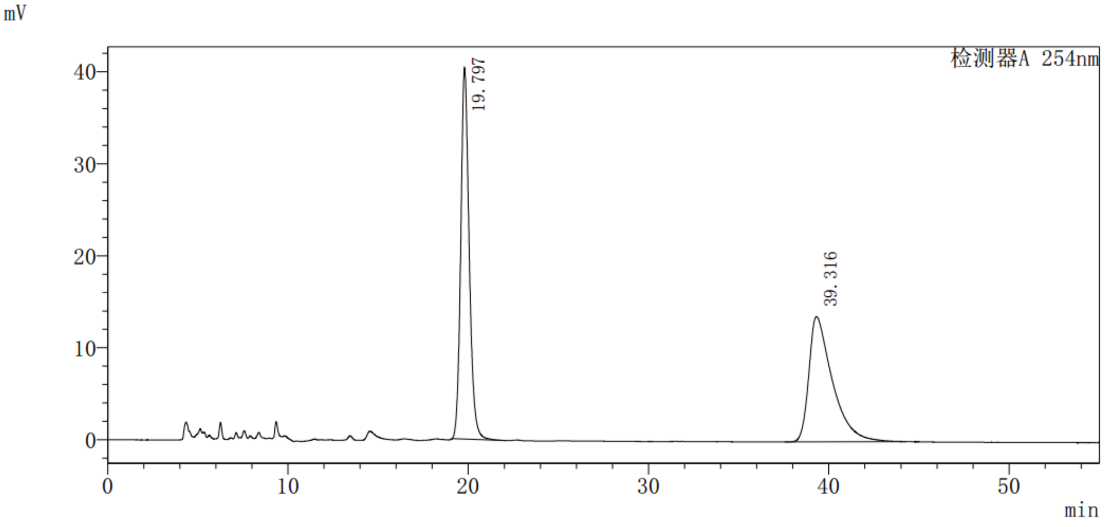

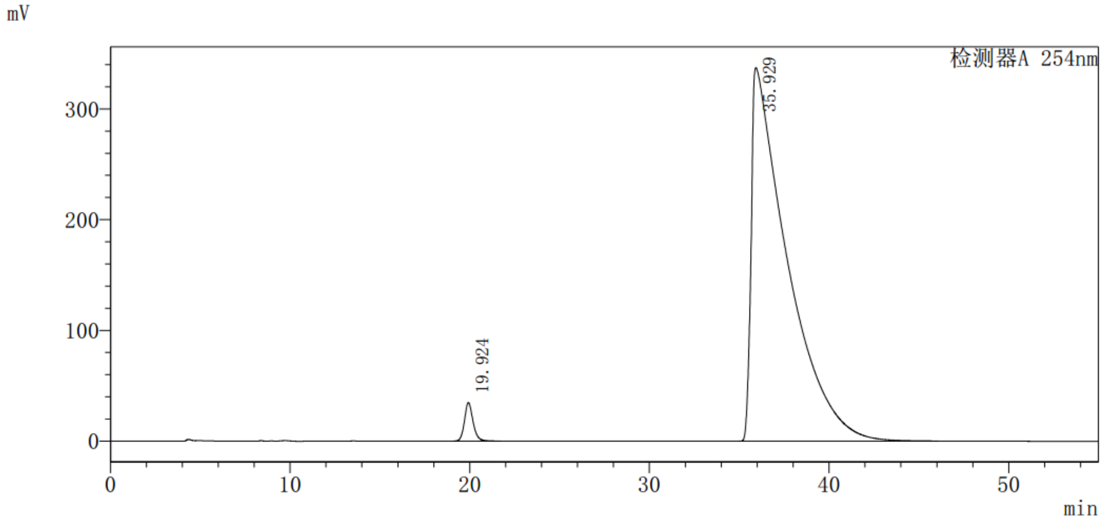

	Retention Time (min)	Relative Area (%)
**Peak 1**	19.924	2.405
**Peak 2**	35.929	97.595

Supplementary Figure 77. HPLC spectra of compound **2t**

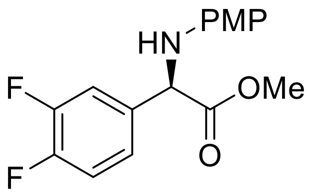

Methyl (*R*)-2-(3,4-difluorophenyl)-2-((4-methoxyphenyl)amino)acetate (2u)

HPLC conditions: DAICEL Chiralpak IE column, *n*-Hexane/*i*-PrOH = 98/2, 254 nm, 0.8 mL/min, t_{major} = 39.634 min, t_{minor} = 21.782 min.

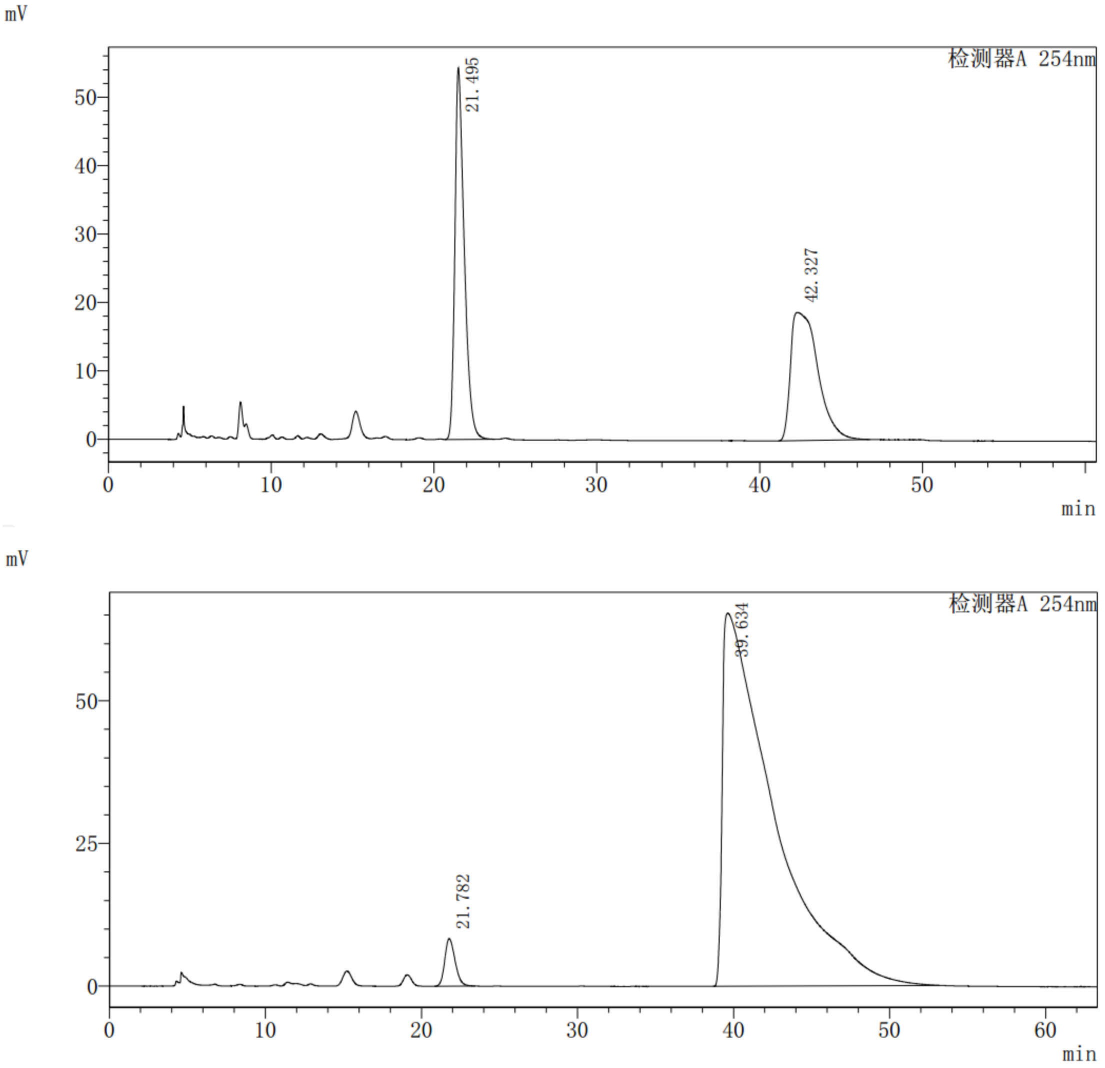

	Retention Time (min)	Relative Area (%)
**Peak 1**	21.782	2.503
**Peak 2**	39.634	97.497

Supplementary Figure 78. HPLC spectra of compound **2u**

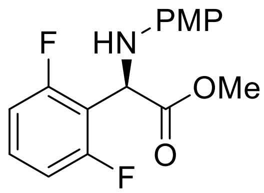

Methyl (*R*)-2-(2,6-difluorophenyl)-2-((4-methoxyphenyl)amino)acetate (2v)

HPLC conditions: DAICEL Chiralpak IE column, *n*-Hexane/*i*-PrOH = 95/5, 254 nm, 0.8 mL/min, t_{major} = 25.203 min, t_{minor} = 22.758 min.

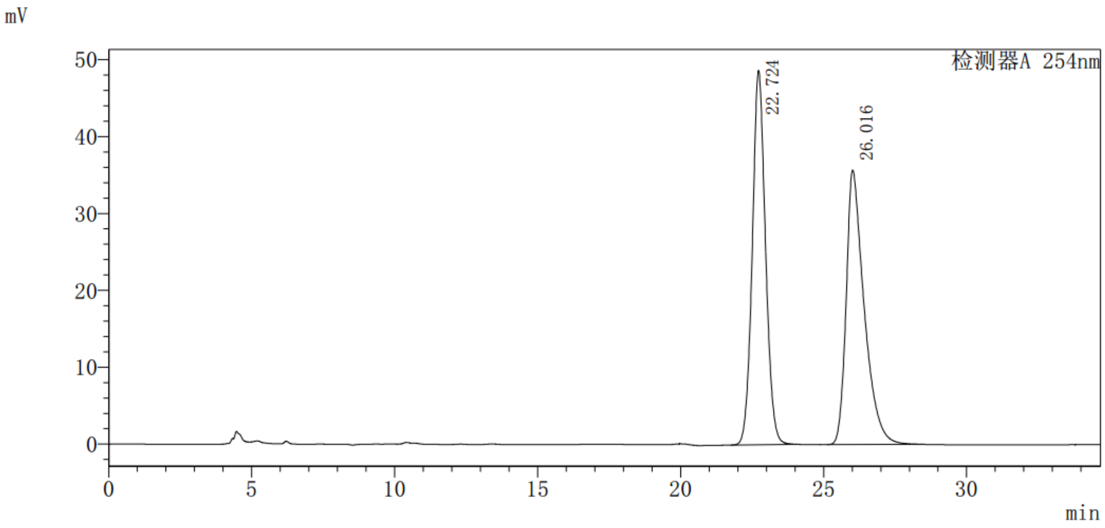

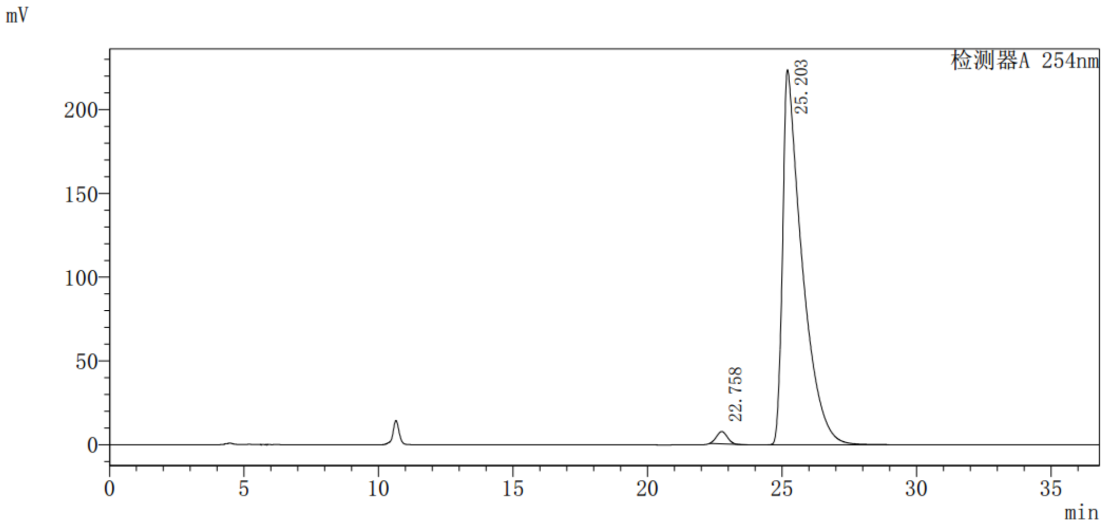

	Retention Time (min)	Relative Area (%)
**Peak 1**	22.758	1.779
**Peak 2**	25.203	98.221

Supplementary Figure 79. HPLC spectra of compound **2v**

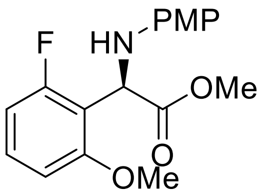

Methyl (*R*)-2-(2-fluoro-6-methoxyphenyl)-2-((4-methoxyphenyl)amino)acetate (2w)

HPLC conditions: DAICEL Chiralpak IE column, *n*-Hexane/*i*-PrOH = 90/10, 254 nm, 0.8 mL/min, t_{major} = 26.262 min, t_{minor} = 28.215 min.

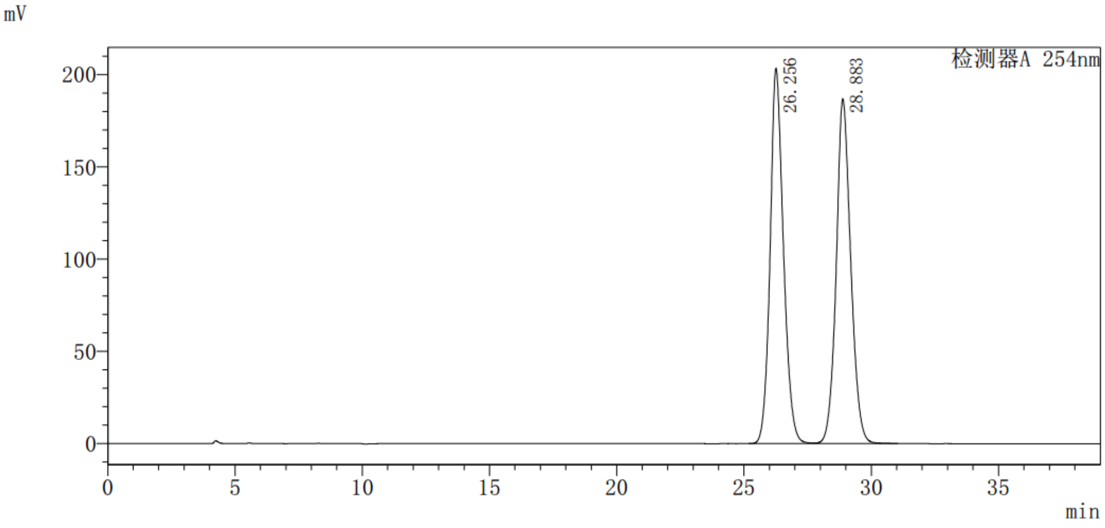

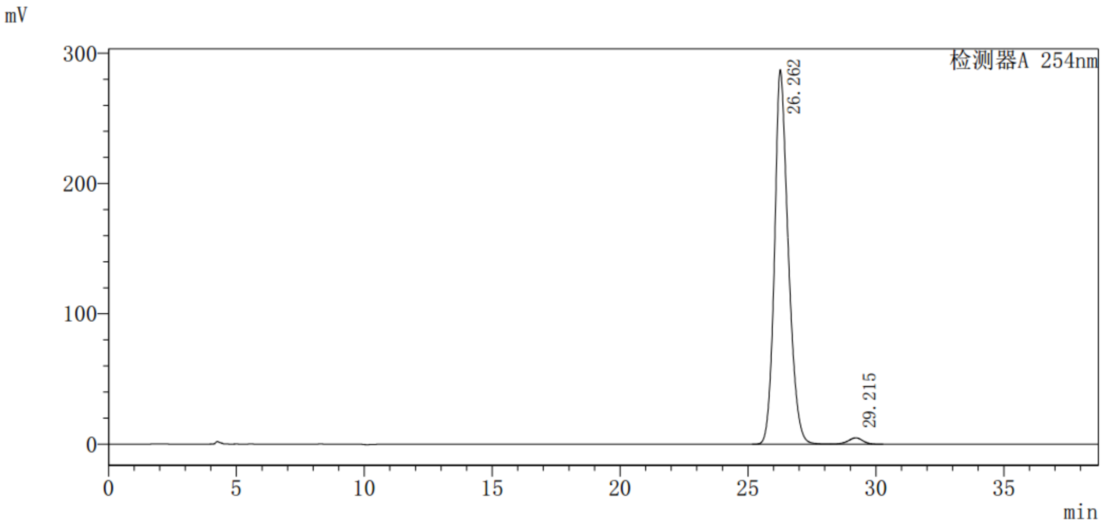

	Retention Time (min)	Relative Area (%)
**Peak 1**	26.262	98.179
**Peak 2**	28.215	1.821

Supplementary Figure 80. HPLC spectra of compound **2w**

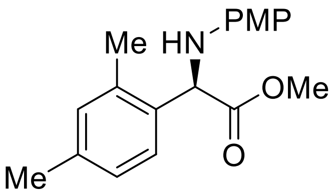

Methyl (*R*)-2-(2,4-dimethylphenyl)-2-((4-methoxyphenyl)amino)acetate (2x)

HPLC conditions: DAICEL Chiralpak IC-3 column, *n*-Hexane/*i*-PrOH = 90/10, 254 nm, 0.8 mL/min, t_{major} = 14.820 min, t_{minor} = 17.046 min.

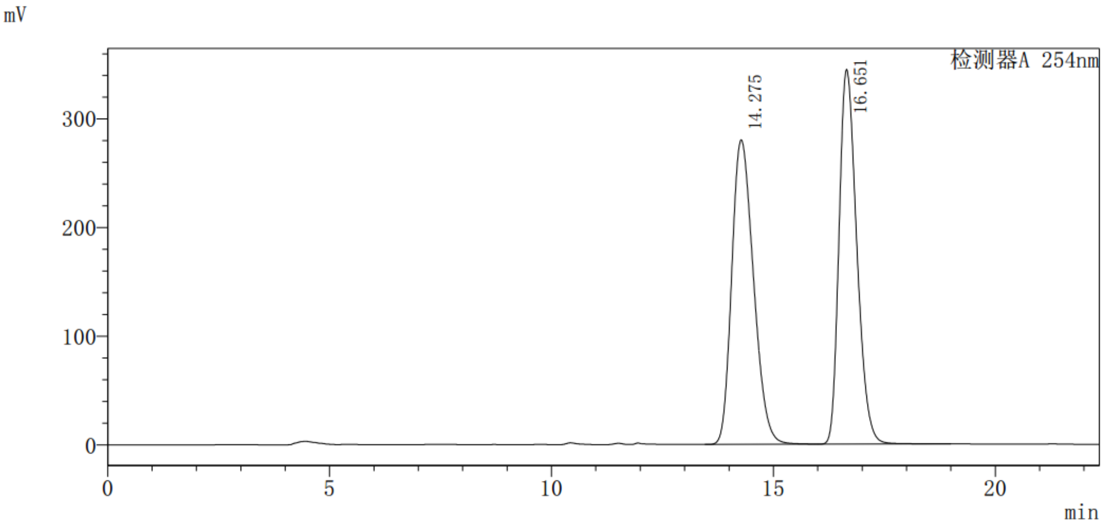

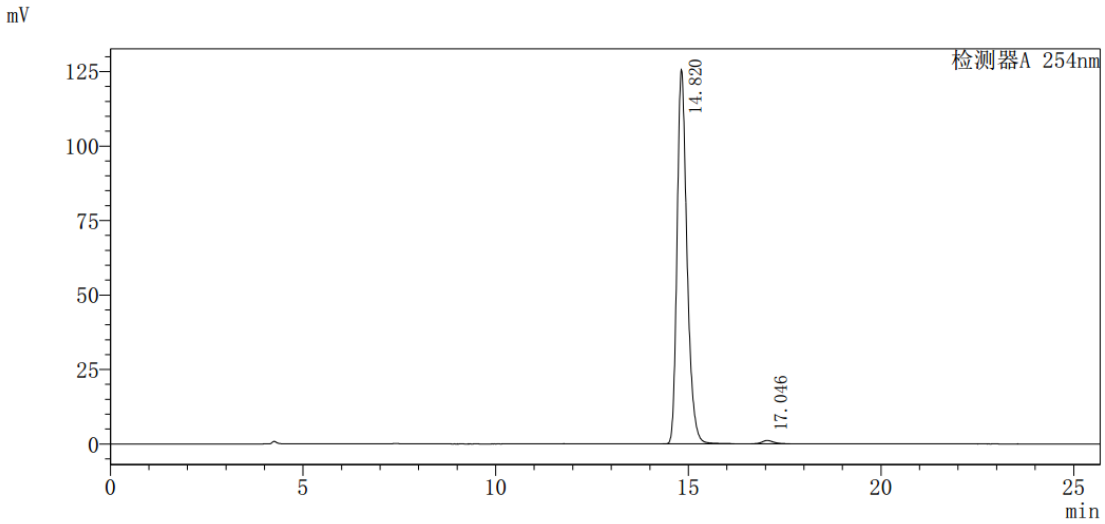

	Retention Time (min)	Relative Area (%)
**Peak 1**	14.820	99.027
**Peak 2**	17.046	0.973

Supplementary Figure 81. HPLC spectra of compound **2x**

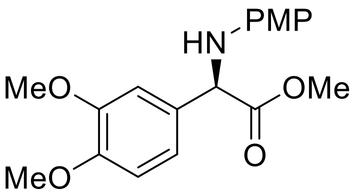

Methyl (*R*)-2-(3,4-dimethoxyphenyl)-2-((4-methoxyphenyl)amino)acetate (2y)

HPLC conditions: DAICEL Chiralpak IE column, *n*-Hexane/*i*-PrOH = 80/20, 254 nm, 0.8 mL/min, t_{major} = 28.426 min, t_{minor} = 24.648 min.

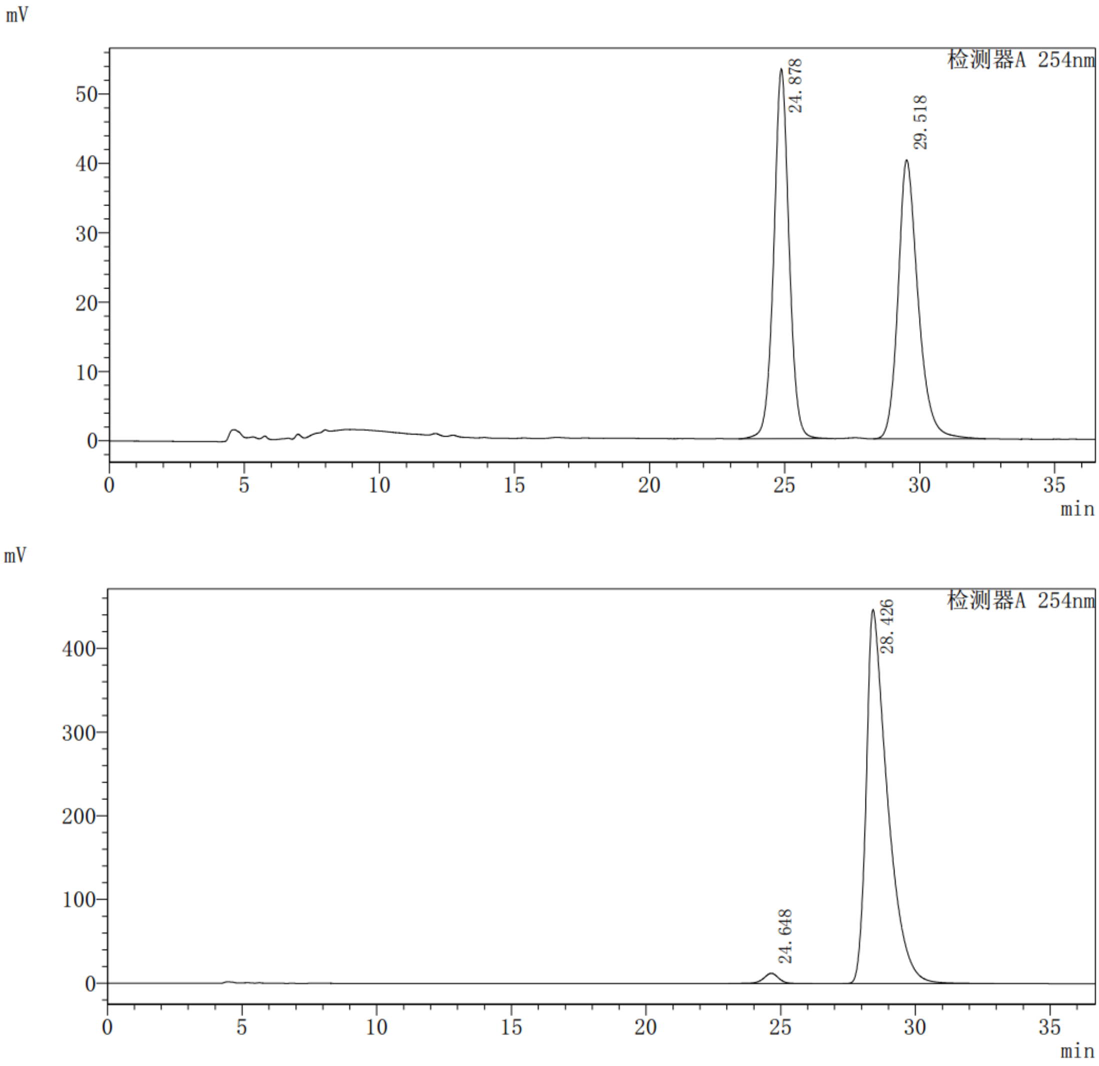

	Retention Time (min)	Relative Area (%)
**Peak 1**	24.648	1.944
**Peak 2**	28.426	98.056

Supplementary Figure 82. HPLC spectra of compound **2y**

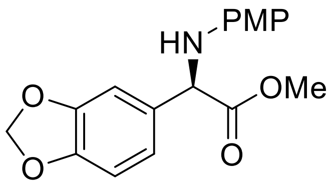

Methyl (*R*)-2-(benzo[*d*][1,3]dioxol-5-yl)-2-((4-methoxyphenyl)amino)acetate (2z)

HPLC conditions: DAICEL Chiralpak IE column, *n*-Hexane/*i*-PrOH = 80/20, 254 nm, 0.8 mL/min, t_{major} = 28.090 min, t_{minor} = 16.268 min.

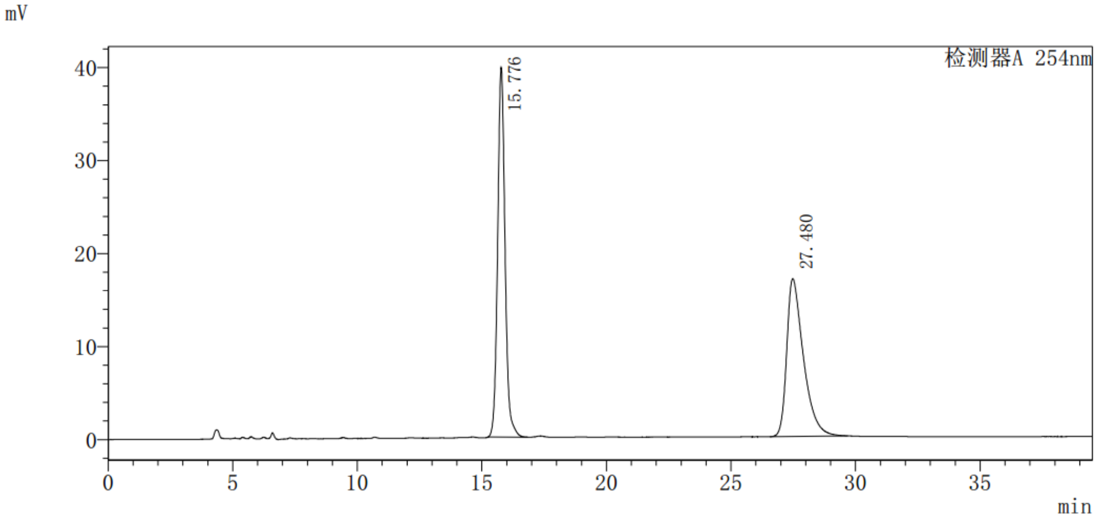

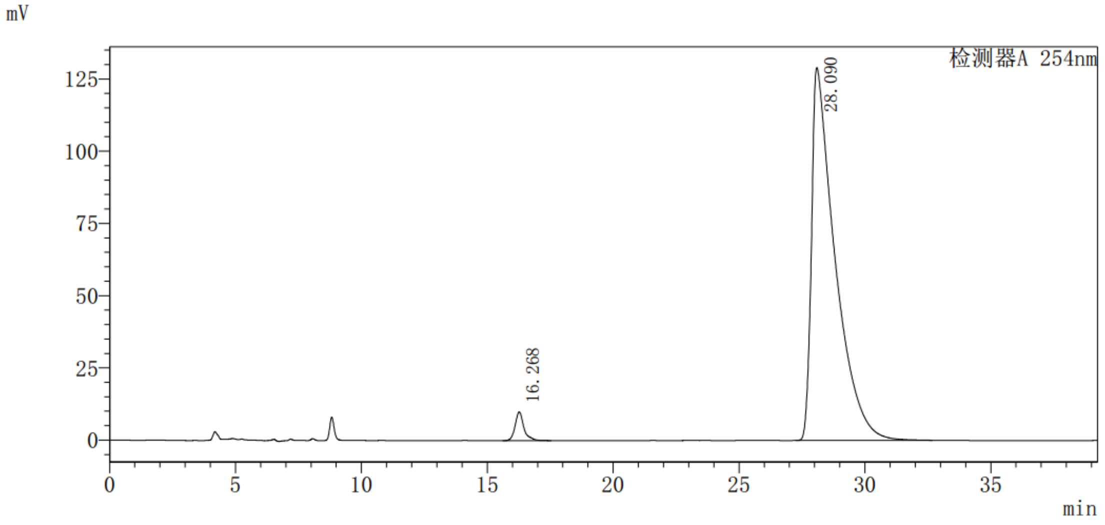

	Retention Time (min)	Relative Area (%)
**Peak 1**	16.268	2.813
**Peak 2**	28.090	97.187

Supplementary Figure 83. HPLC spectra of compound **2z**

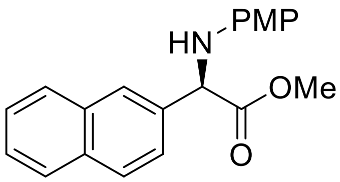

Methyl (*R*)-2-((4-methoxyphenyl)amino)-2-(naphthalen-2-yl)acetate (2aa)

HPLC conditions: DAICEL Chiralpak IE column, *n*-Hexane/*i*-PrOH = 90/10, 254 nm, 0.8 mL/min, t_{major} = 24.698 min, t_{minor} = 15.563 min.

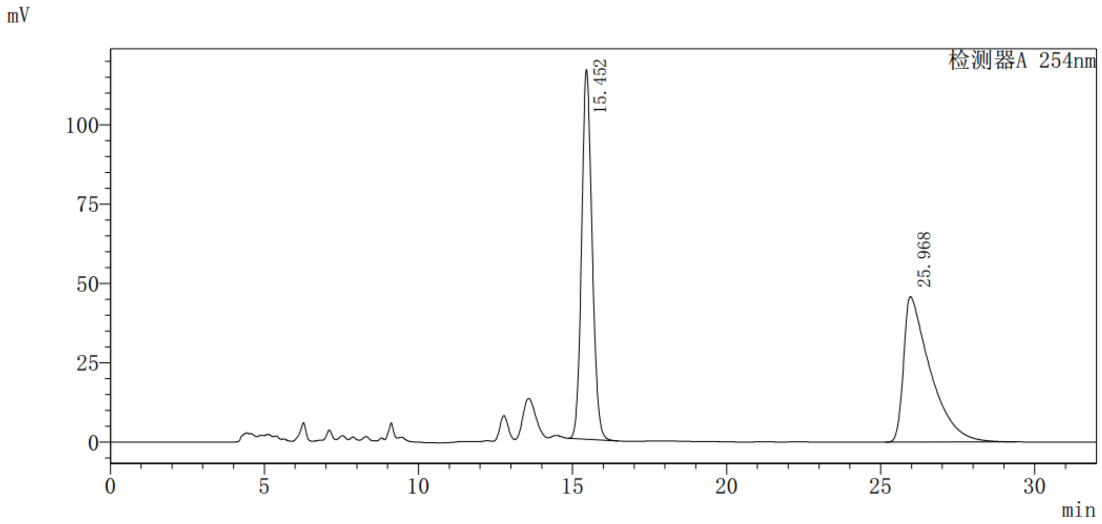

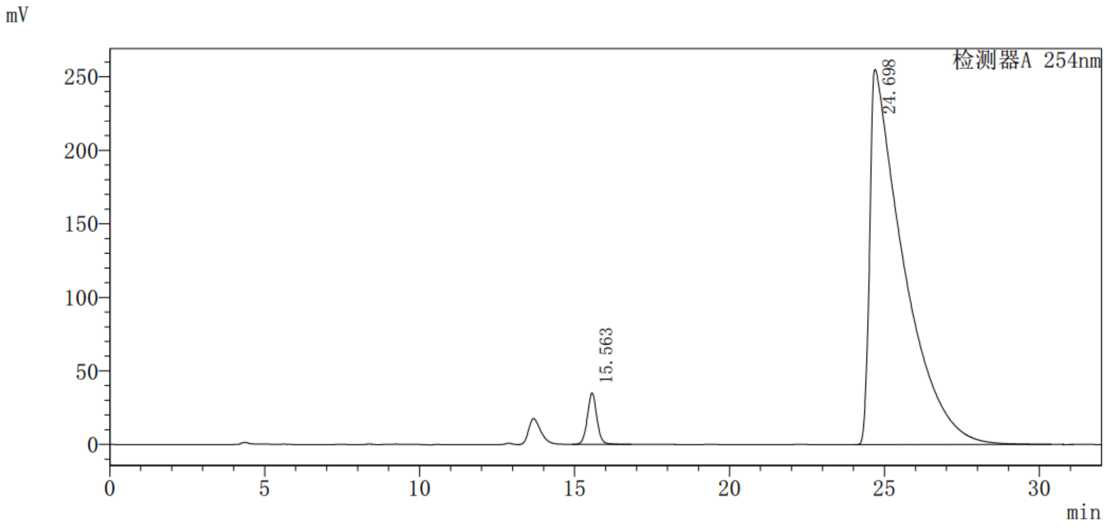

	Retention Time (min)	Relative Area (%)
**Peak 1**	15.563	3.741
**Peak 2**	24.698	96.259

Supplementary Figure 84. HPLC spectra of compound **2aa**

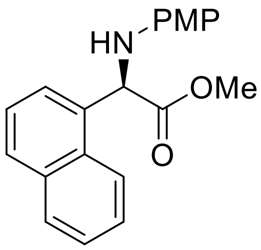

Methyl (*R*)-2-((4-methoxyphenyl)amino)-2-(naphthalen-1-yl)acetate (2ab)

HPLC conditions: DAICEL Chiralpak IE column, *n*-Hexane/*i*-PrOH = 95/5, 254 nm, 0.8 mL/min, t_{major} = 25.272 min, t_{minor} = 19.838 min.

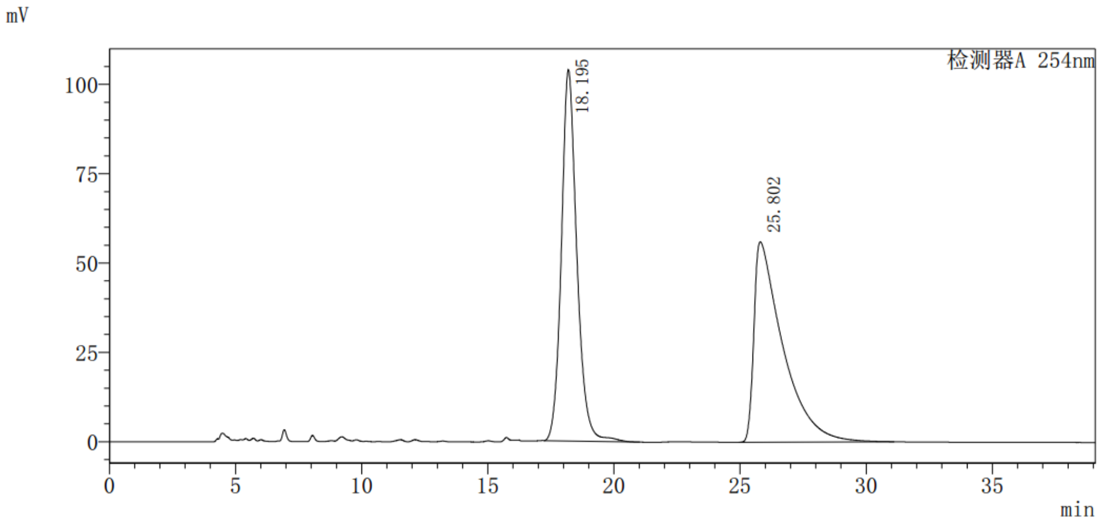

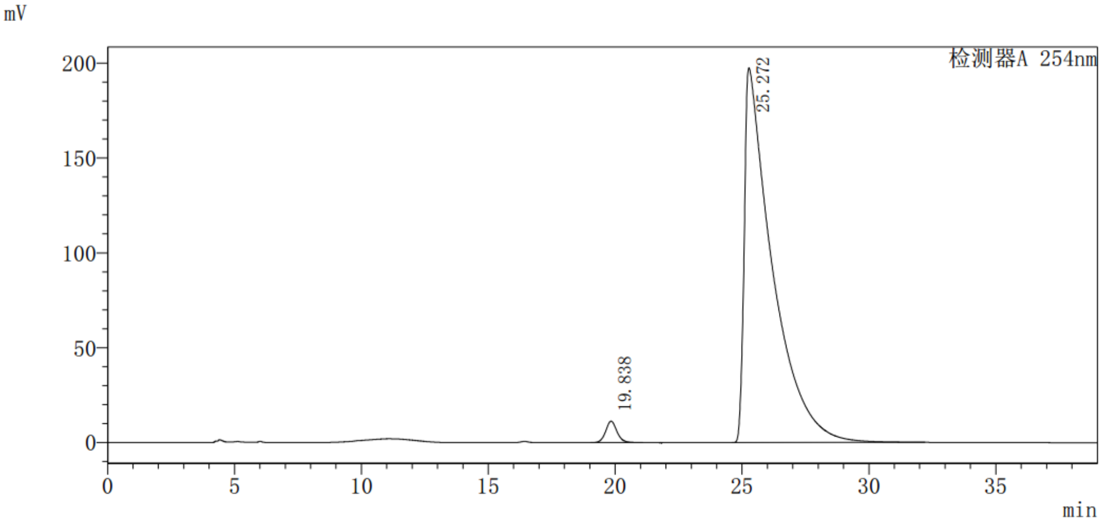

	Retention Time (min)	Relative Area (%)
**Peak 1**	19.838	2.261
**Peak 2**	25.272	97.739

Supplementary Figure 85. HPLC spectra of compound **2ab**

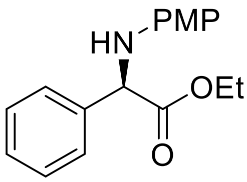

Ethyl (*R*)-2-((4-methoxyphenyl)amino)-2-phenylacetate (2ac)

HPLC conditions: DAICEL Chiralpak IE column, *n*-Hexane/*i*-PrOH = 95/5, 254 nm, 0.8 mL/min, t_{major} = 20.575 min, t_{minor} = 17.438 min.

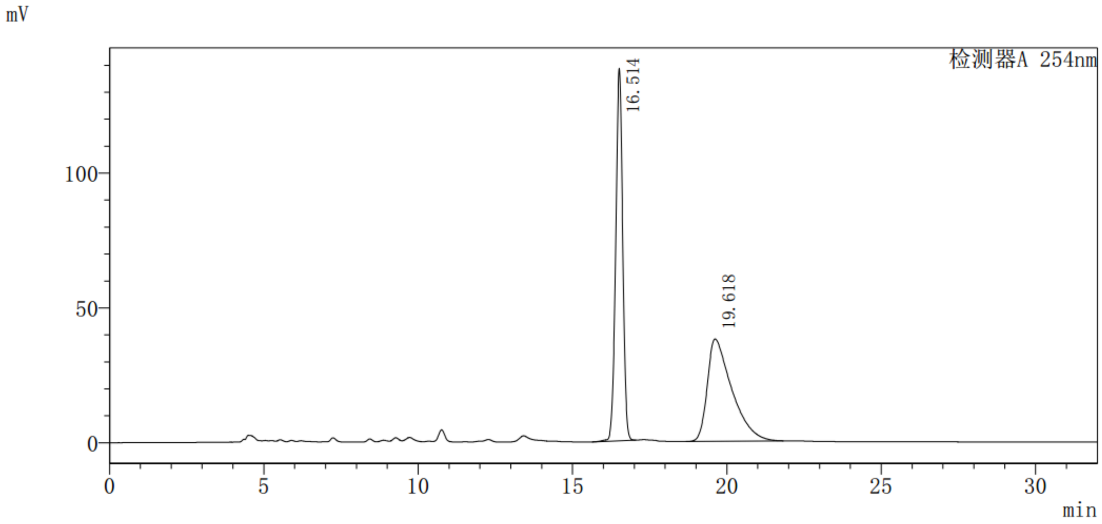

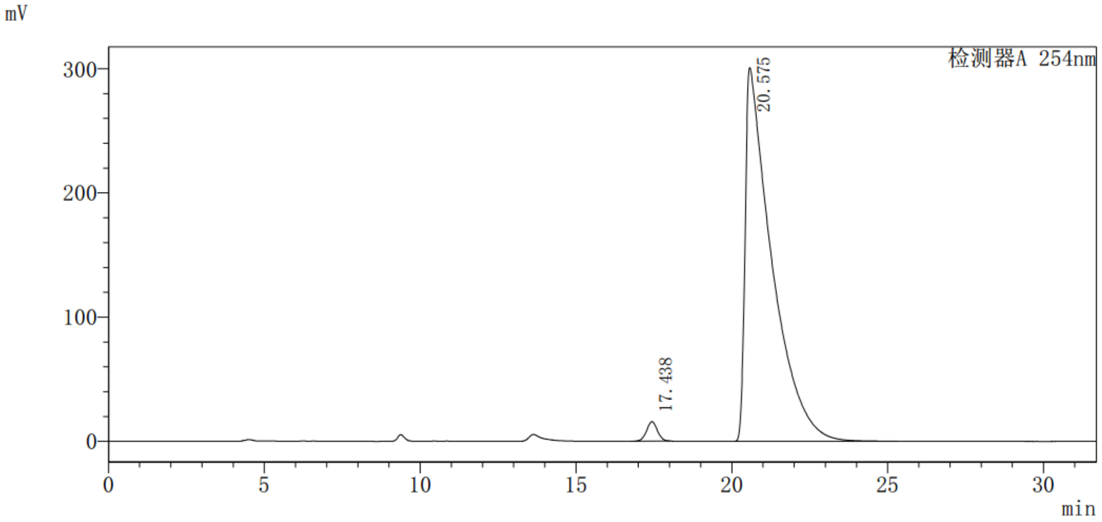

	Retention Time (min)	Relative Area (%)
**Peak 1**	17.438	2.085
**Peak 2**	20.575	97.915

Supplementary Figure 86. HPLC spectra of compound **2ac**

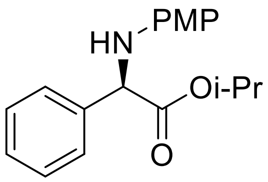

Isopropyl (*R*)-2-((4-methoxyphenyl)amino)-2-phenylacetate (2ad)

HPLC conditions: DAICEL Chiralpak AS-H column, *n*-Hexane/*i*-PrOH = 95/5, 254 nm, 0.8 mL/min, t_{major} = 14.978 min, t_{minor} = 12.037 min.

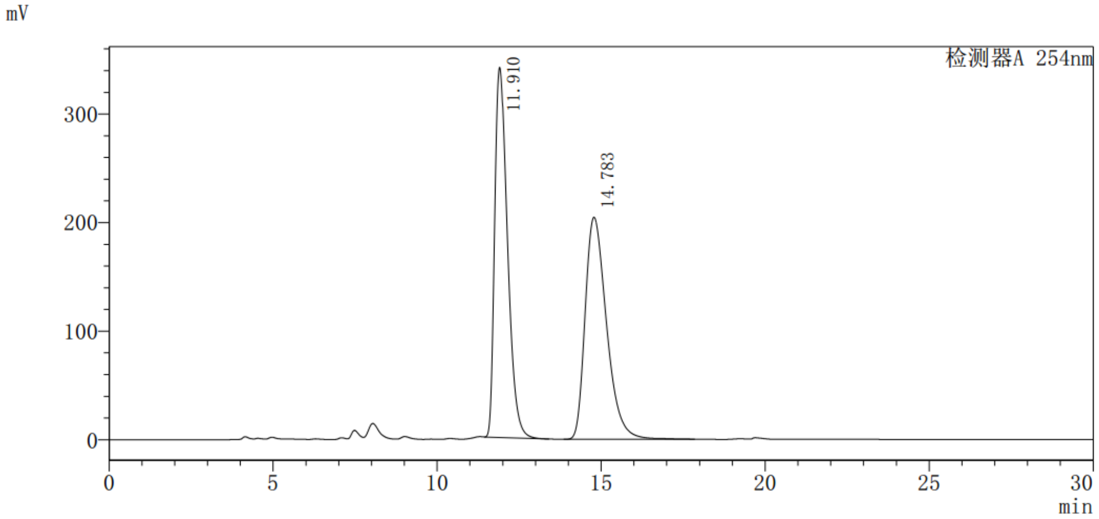

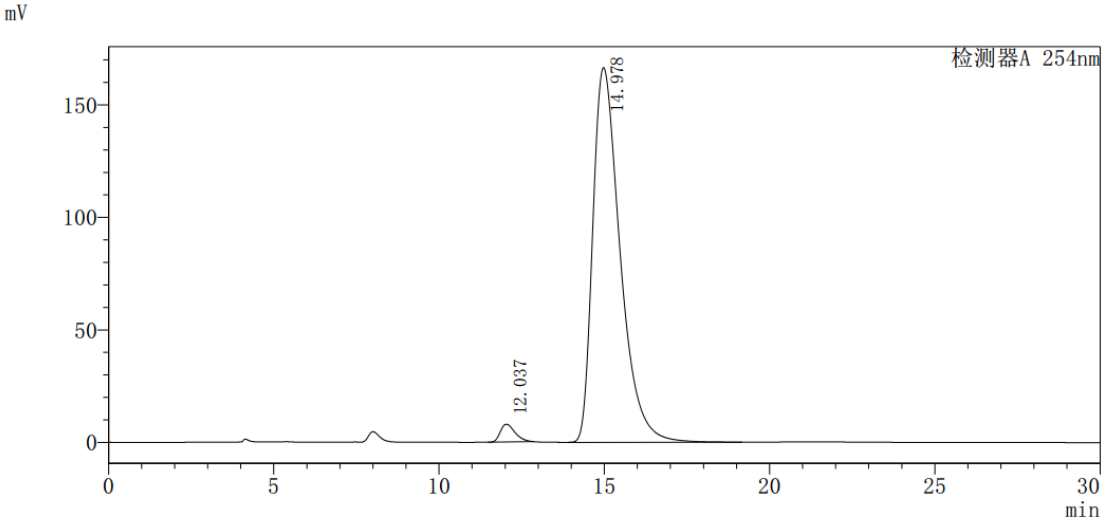

	Retention Time (min)	Relative Area (%)
**Peak 1**	12.037	2.470
**Peak 2**	14.978	97.530

Supplementary Figure 87. HPLC spectra of compound **2ad**

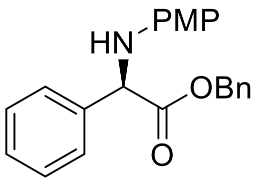

Benzyl (*R*)-2-((4-methoxyphenyl)amino)-2-phenylacetate (2ae)

HPLC conditions: DAICEL Chiralpak IC-3 column, *n*-Hexane/*i*-PrOH = 90/10, 254 nm, 0.8 mL/min, t_{major} = 14.094 min, t_{minor} = 18.201 min.

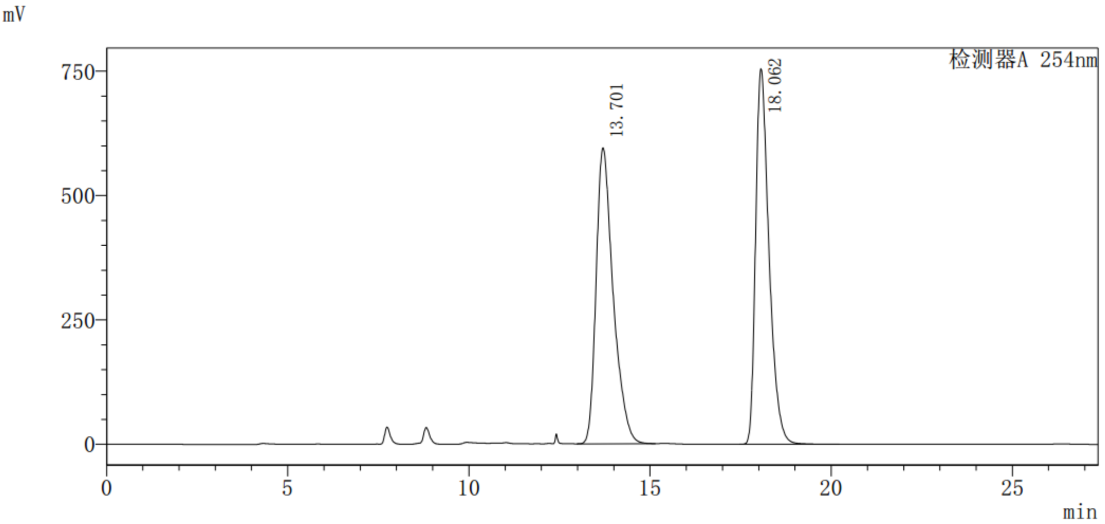

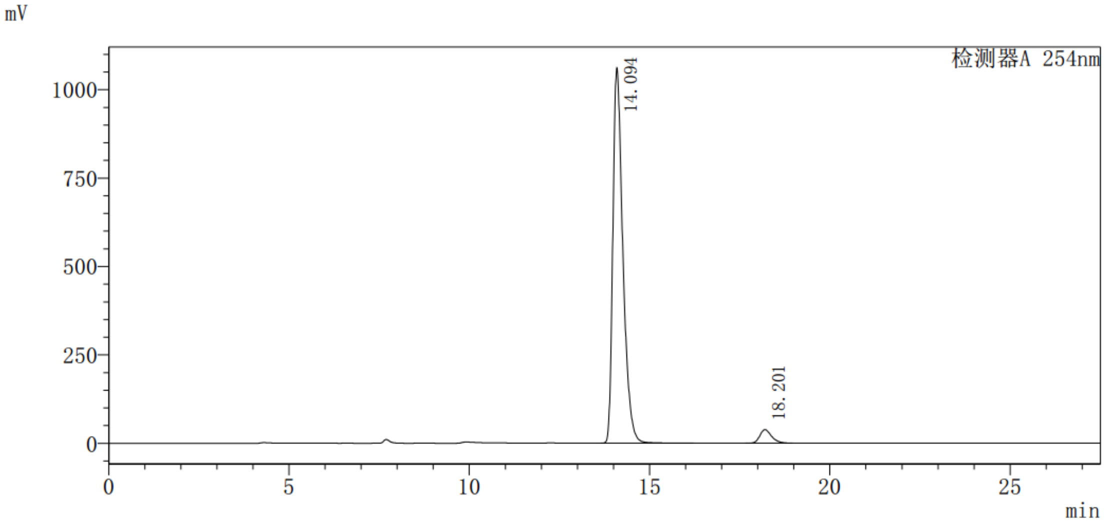

	Retention Time (min)	Relative Area (%)
**Peak 1**	14.094	95.927
**Peak 2**	18.201	4.073

Supplementary Figure 88. HPLC spectra of compound **2ae**

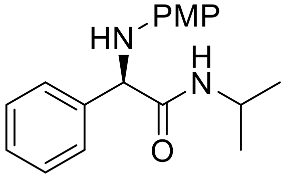

(*R*)-*N*-isopropyl-2-((4-methoxyphenyl)amino)-2-phenylacetamide (2af)

HPLC conditions: DAICEL Chiralpak OJ-H column, *n*-Hexane/*i*-PrOH = 90/10, 254 nm, 0.8 mL/min, t_{major} = 28.647 min, t_{minor} = 35.398 min.

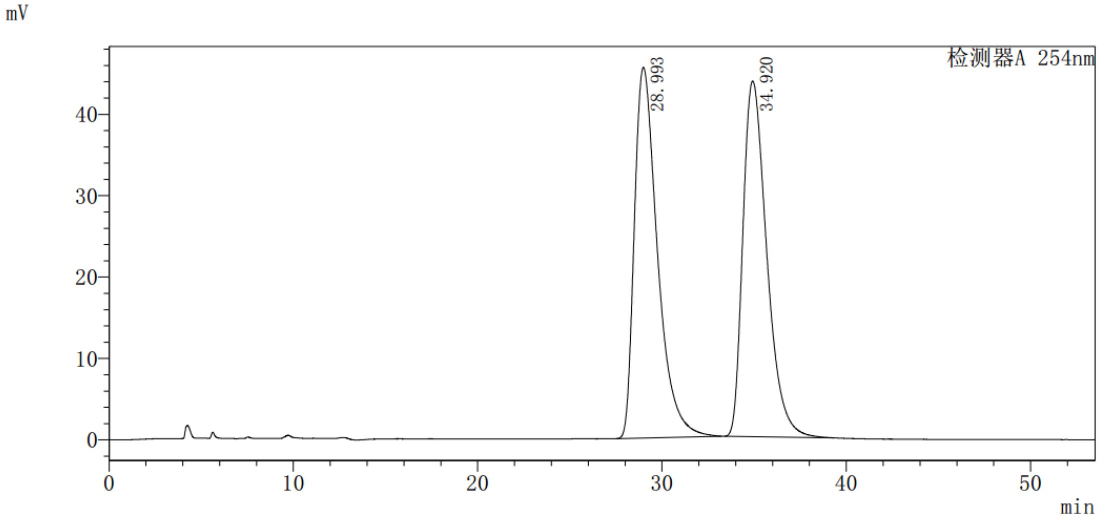

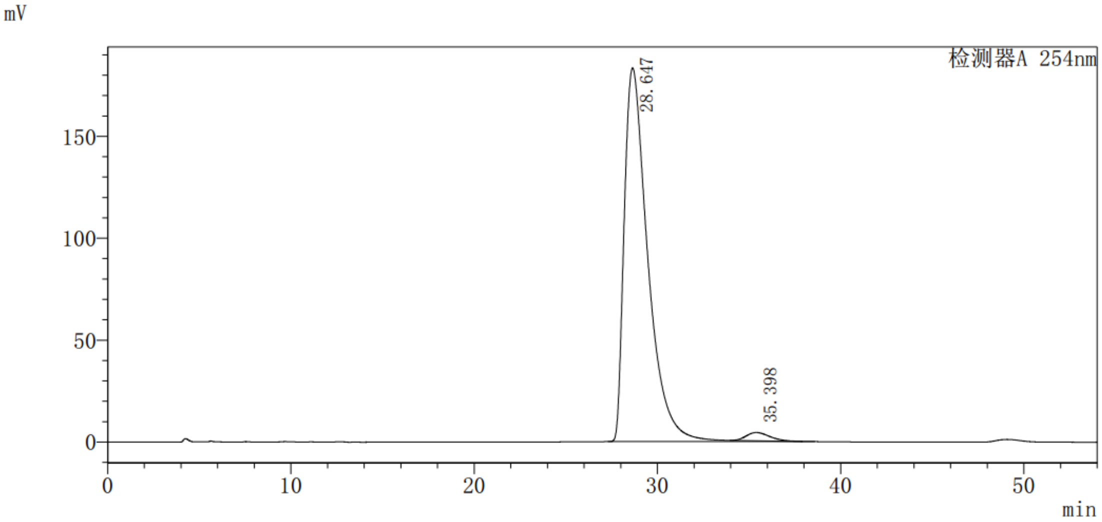

	Retention Time (min)	Relative Area (%)
**Peak 1**	28.647	97.847
**Peak 2**	35.398	2.153

Supplementary Figure 89. HPLC spectra of compound **2af**

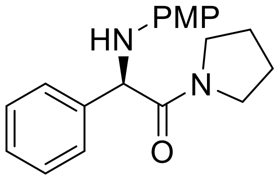

(*R*)-2-((4-methoxyphenyl)amino)-2-phenyl-1-(pyrrolidin-1-yl)ethan-1-one (2ag)

HPLC conditions: DAICEL Chiralpak OJ-H column, *n*-Hexane/*i*-PrOH = 80/20, 254 nm, 0.8 mL/min, t_{major} = 27.700 min, t_{minor} = 36.316 min.

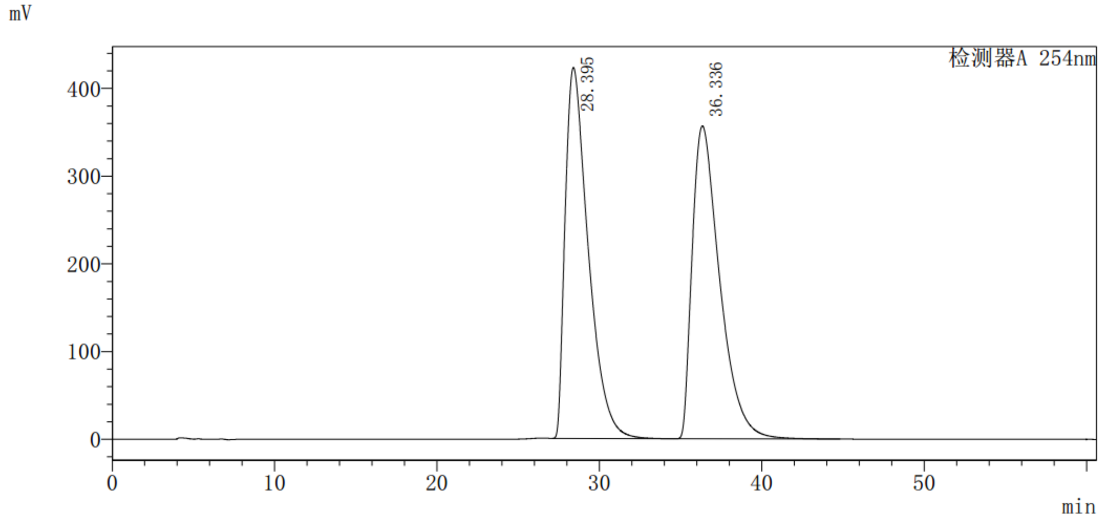

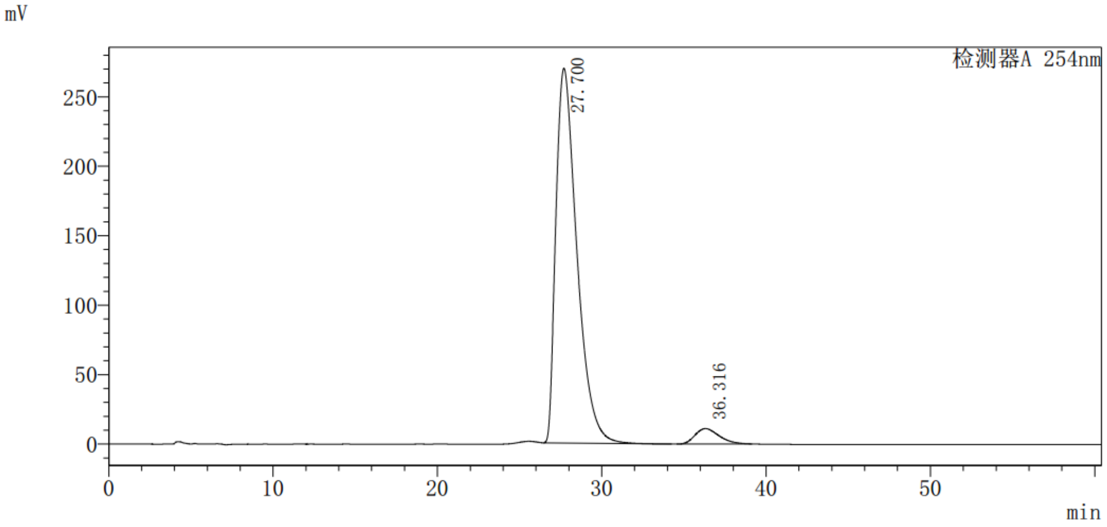

	Retention Time (min)	Relative Area (%)
**Peak 1**	27.700	95.481
**Peak 2**	36.316	4.519

Supplementary Figure 90. HPLC spectra of compound **2ag**

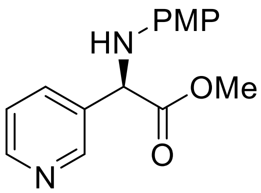

Methyl (*R*)-2-((4-methoxyphenyl)amino)-2-(pyridin-3-yl)acetate (2ah)

HPLC conditions: DAICEL Chiralpak OD-H column, *n*-Hexane/*i*-PrOH = 80/20, 254 nm, 0.8 mL/min, t_{major} = 12.962 min, t_{minor} = 18.073 min.

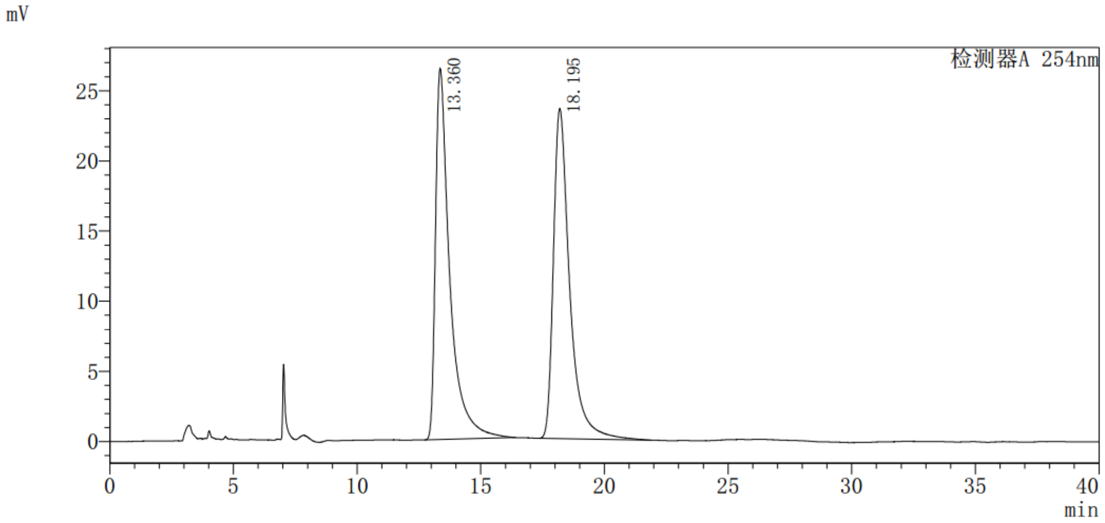

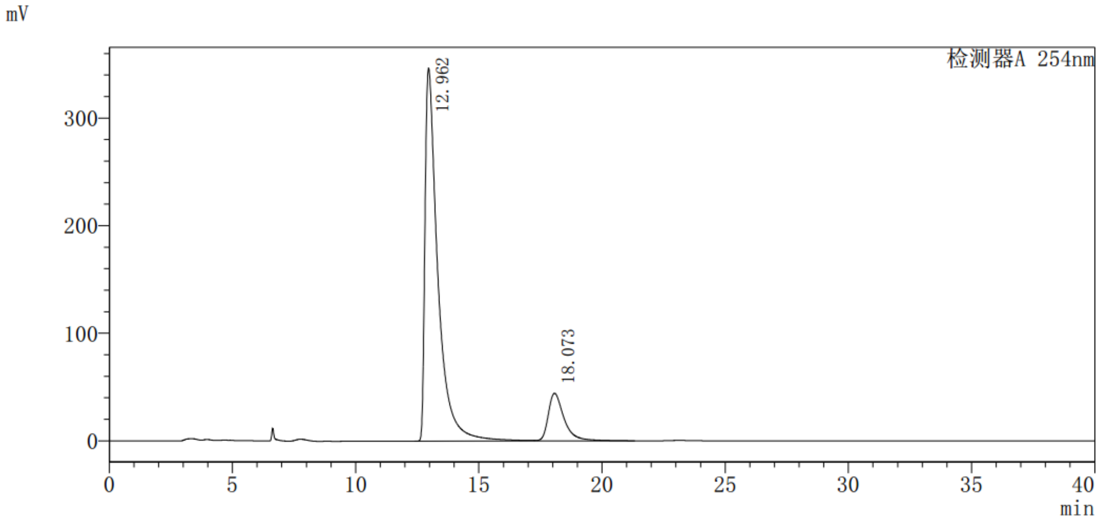

	Retention Time (min)	Relative Area (%)
**Peak 1**	12.962	86.383
**Peak 2**	18.073	13.617

Supplementary Figure 91. HPLC spectra of compound **2ah**

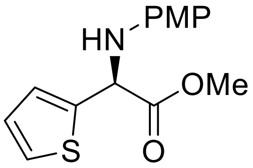

Methyl (*S*)-2-((4-methoxyphenyl)amino)-2-(thiophen-2-yl)acetate (2ai)

HPLC conditions: DAICEL Chiralpak IC-3 column, *n*-Hexane/*i*-PrOH = 95/5, 254 nm, 0.8 mL/min, t_{major} = 20.352 min, t_{minor} = 18.426 min.

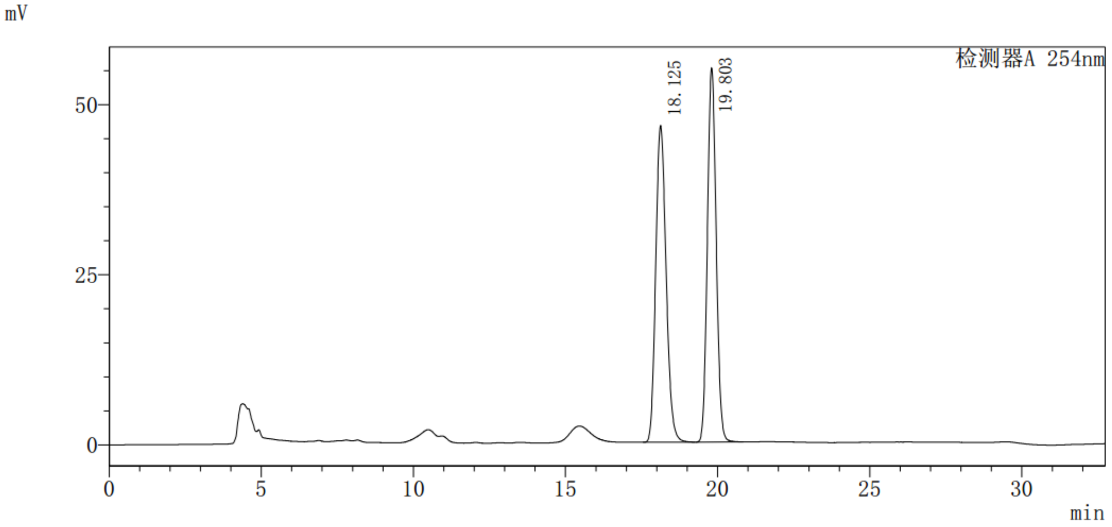

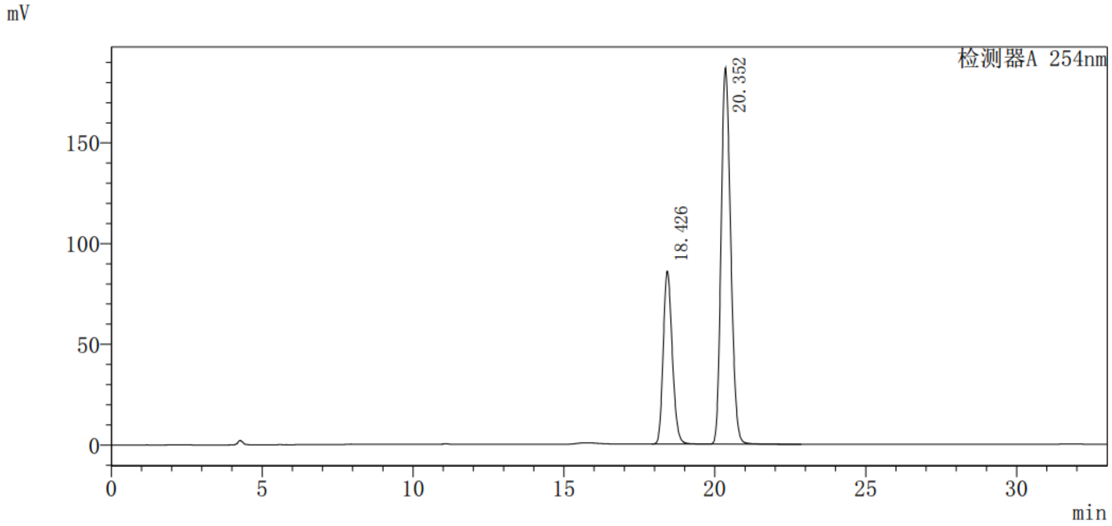

	Retention Time(min)	Relative Area (%)
**Peak 1**	18.426	30.210
**Peak 2**	20.352	69.790

Supplementary Figure 92. HPLC spectra of compound **2ai**

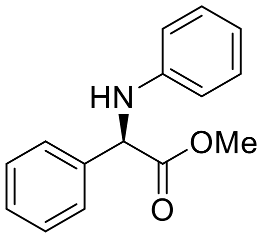

Methyl (*R*)-2-phenyl-2-(phenylamino)acetate (2aj)

HPLC conditions: DAICEL Chiralpak IE column, *n*-Hexane/*i*-PrOH = 98/2, 254 nm, 0.8 mL/min, t_{major} = 9.209 min, t_{minor} = 8.735 min.

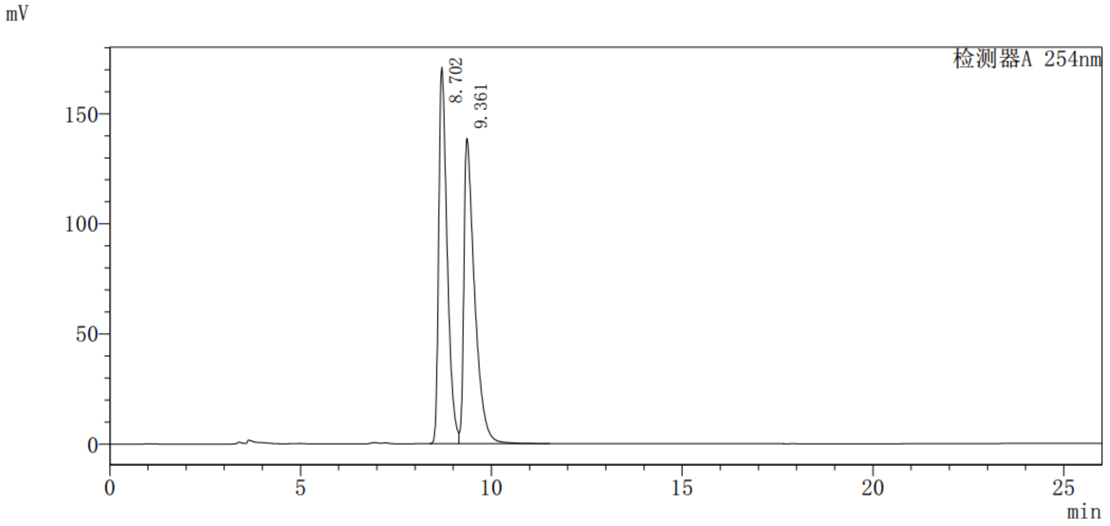

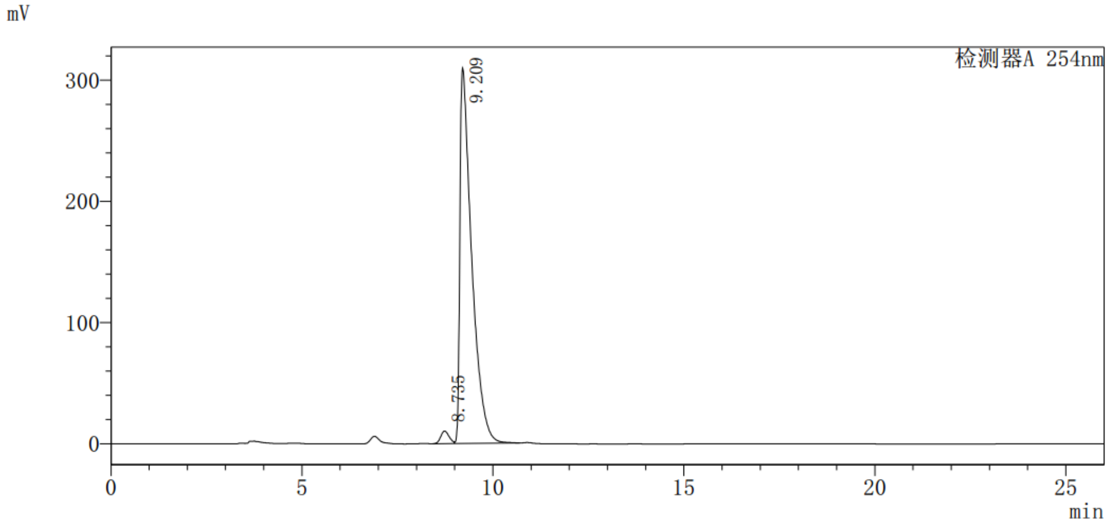

	Retention Time(min)	Relative Area (%)
**Peak 1**	8.735	2.346
**Peak 2**	9.209	97.654

Supplementary Figure 93. HPLC spectra of compound **2aj**

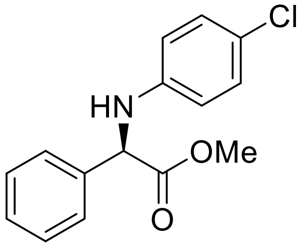

Methyl (*R*)-2-((4-chlorophenyl)amino)-2-phenylacetate (2ak)

HPLC conditions: DAICEL Chiralpak OD-H column, *n*-Hexane/*i*-PrOH = 95/5, 254 nm, 0.8 mL/min, t_{major} = 9.299 min, t_{minor} = 10.523 min.

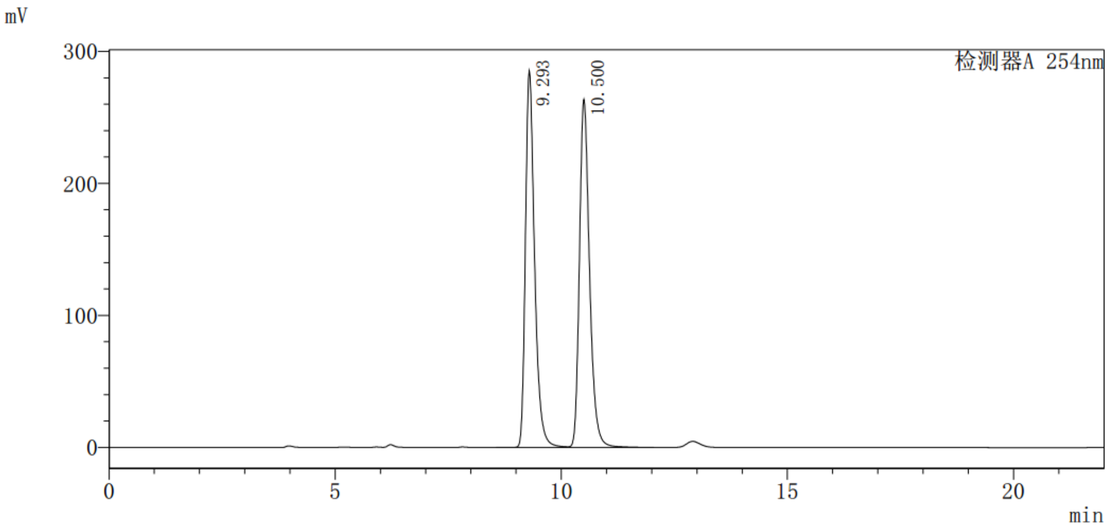

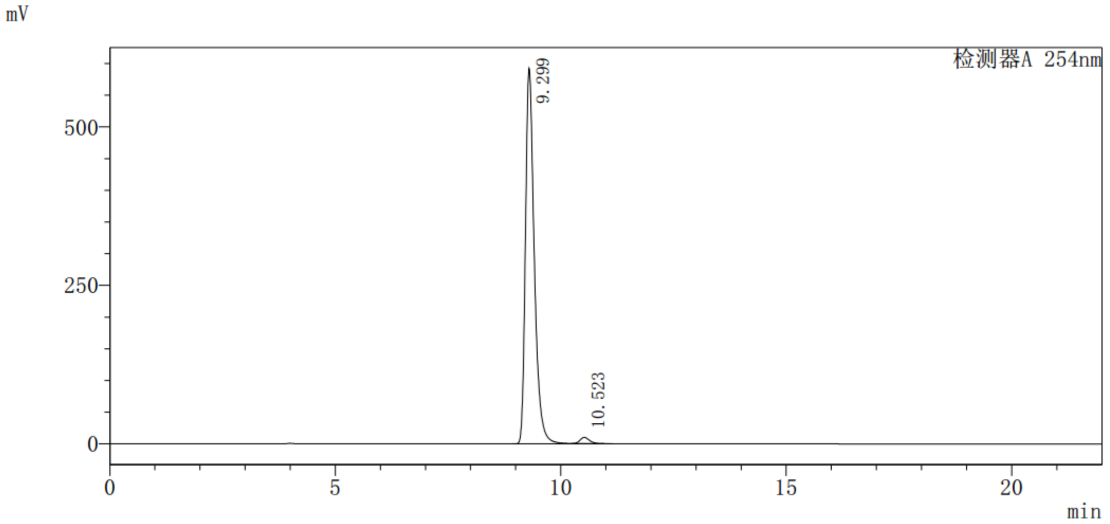

	Retention Time(min)	Relative Area (%)
**Peak 1**	9.299	98.258
**Peak 2**	10.523	1.742

Supplementary Figure 94. HPLC spectra of compound **2ak**

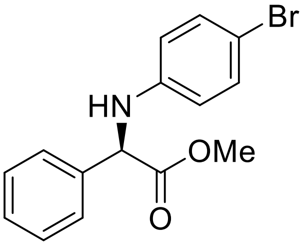

Methyl (*R*)-2-((4-bromophenyl)amino)-2-phenylacetate (2al)

HPLC conditions: DAICEL Chiralpak OD-H column, *n*-Hexane/*i*-PrOH = 95/5, 254 nm, 0.8 mL/min, t_{major} = 9.874 min, t_{minor} = 11.602 min.

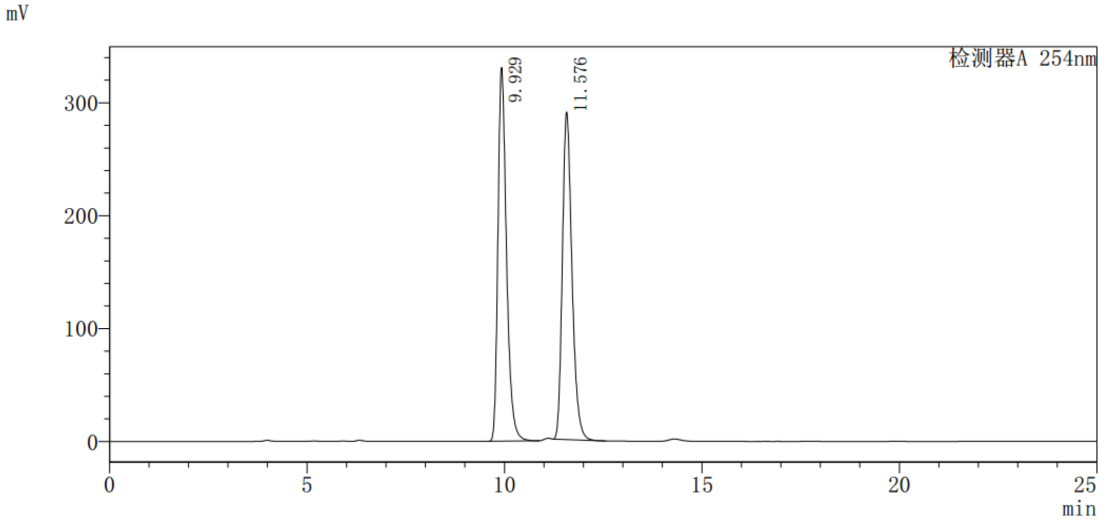

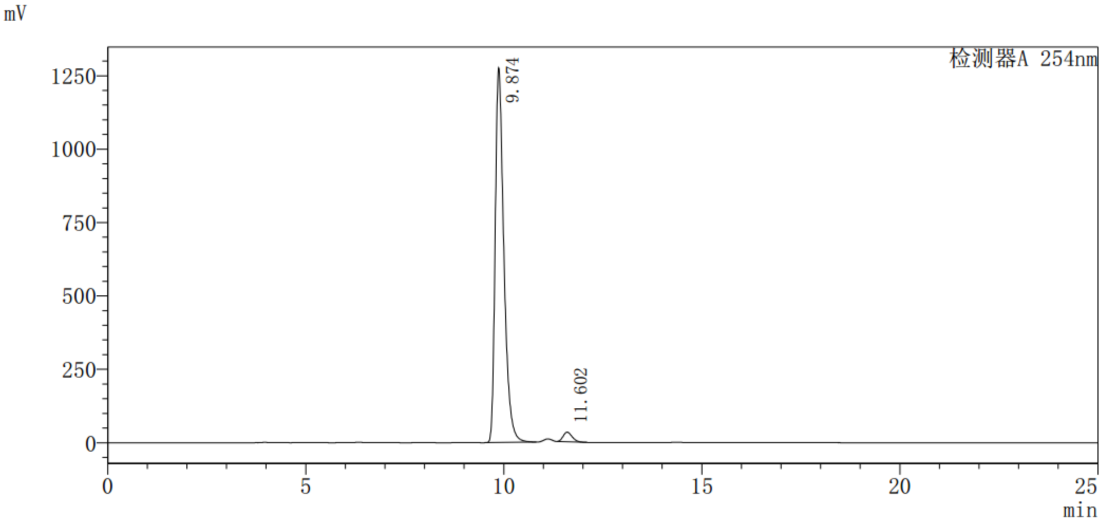

	Retention Time(min)	Relative Area (%)
**Peak 1**	9.874	97.384
**Peak 2**	11.602	2.616

Supplementary Figure 95. HPLC spectra of compound **2al**

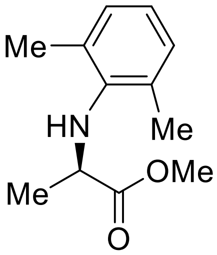

Methyl (2,6-dimethylphenyl)-D-alaninate (2am)

HPLC conditions: DAICEL Chiralpak OD-H column, *n*-Hexane/*i*-PrOH = 99/1, 254 nm, 0.8 mL/min, t_{major} = 7.695 min, t_{minor} = 8.402 min.

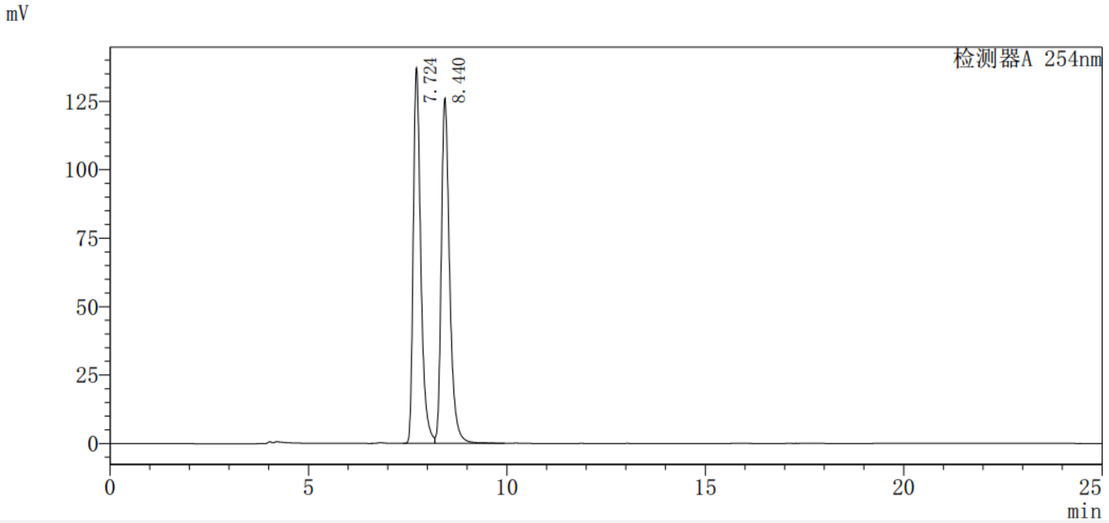

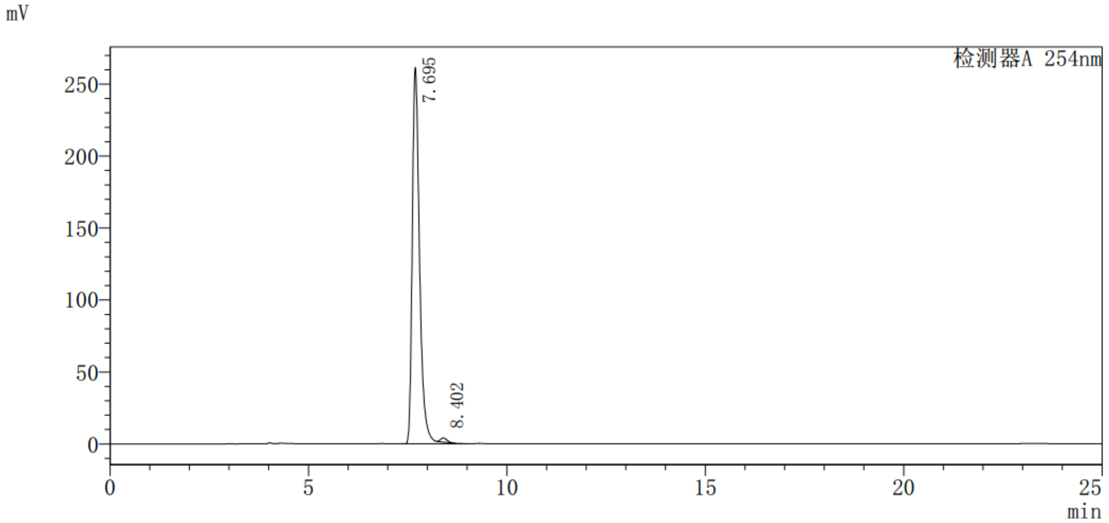

	Retention Time(min)	Relative Area (%)
**Peak 1**	7.695	99.062
**Peak 2**	8.402	0.938

Supplementary Figure 96. HPLC spectra of compound **2am**

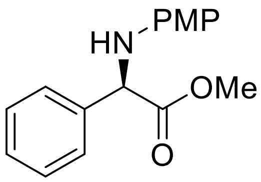

Methyl (*R*)-2-((4-methoxyphenyl)amino)-2-phenylacetate (2a on a gram scale)

HPLC conditions: DAICEL Chiralpak IE column, *n*-Hexane/*i*-PrOH = 95/5, 254 nm, 0.8 mL/min, t_{major} = 23.299 min, t_{minor} = 18.670 min.

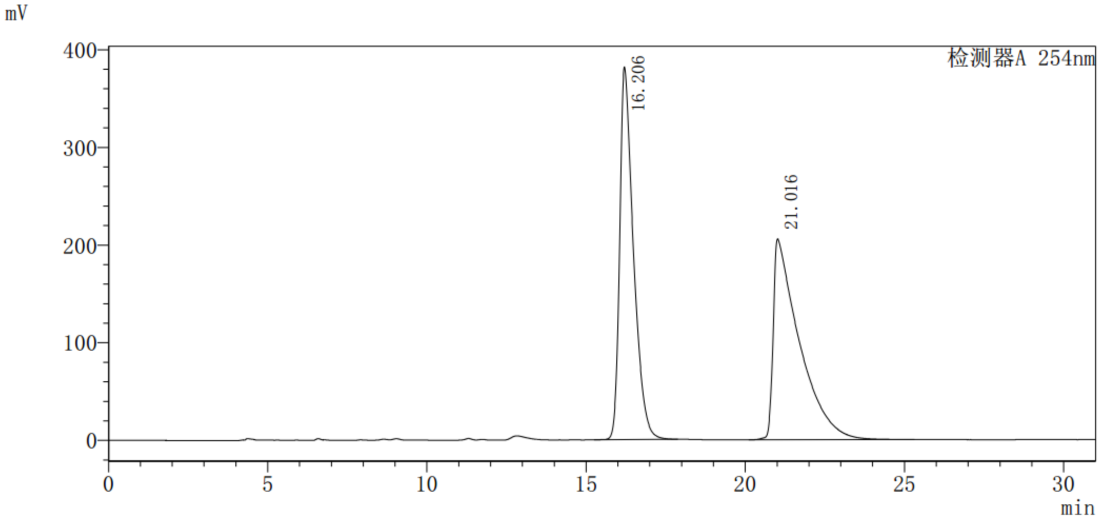

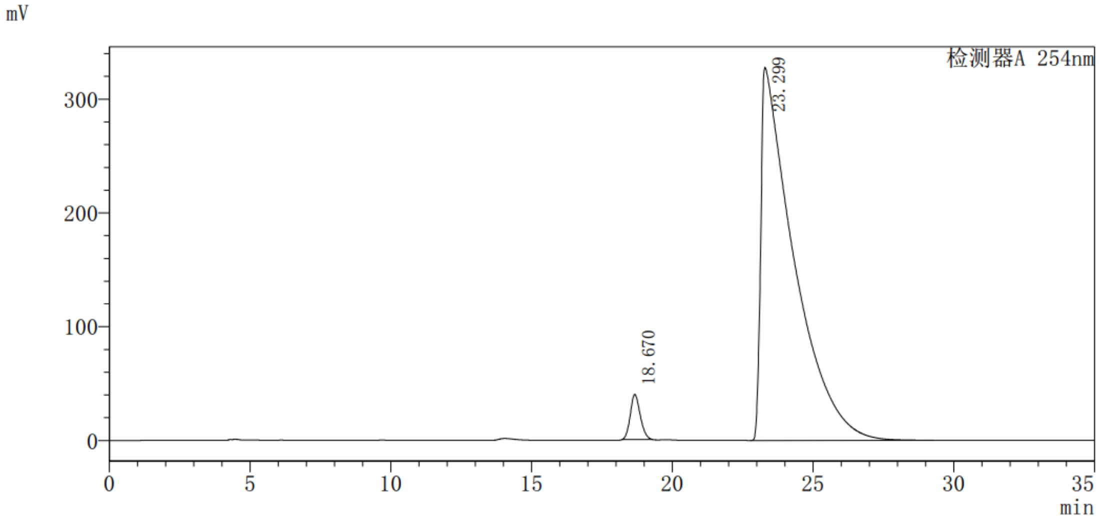

	Retention Time (min)	Relative Area (%)
**Peak 1**	18.670	3.535
**Peak 2**	23.299	96.465

Supplementary Figure 97. HPLC spectra of compound **2a**

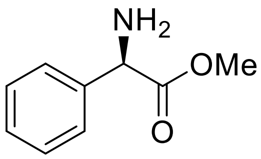

Methyl (*R*)-2-amino-2-phenylacetate (3)

HPLC conditions: DAICEL Chiralpak AS-H column, *n*-Hexane/*i*-PrOH = 90/10, 210 nm, 0.8 mL/min, t_{major} = 16.186 min, t_{minor} = 13.545 min.

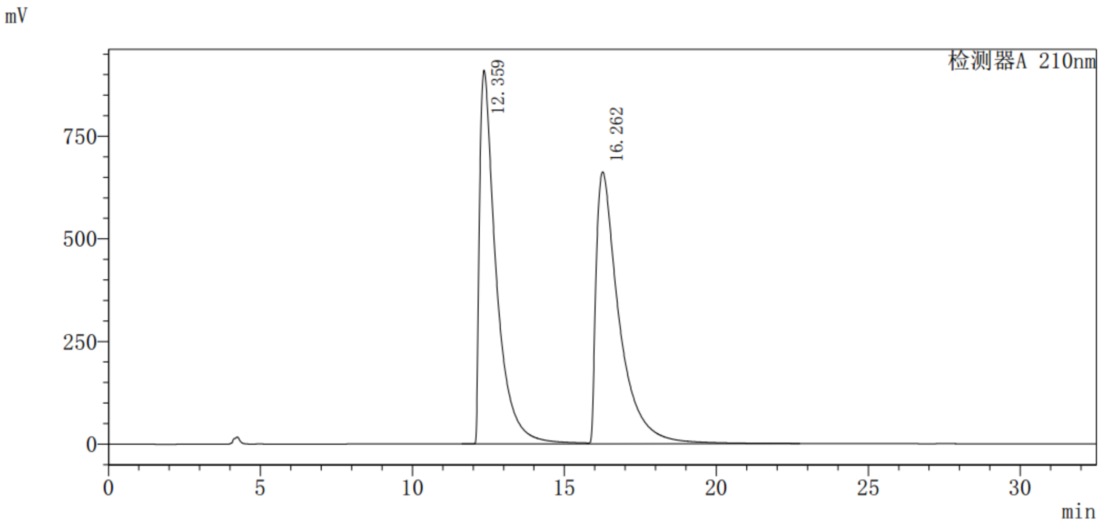

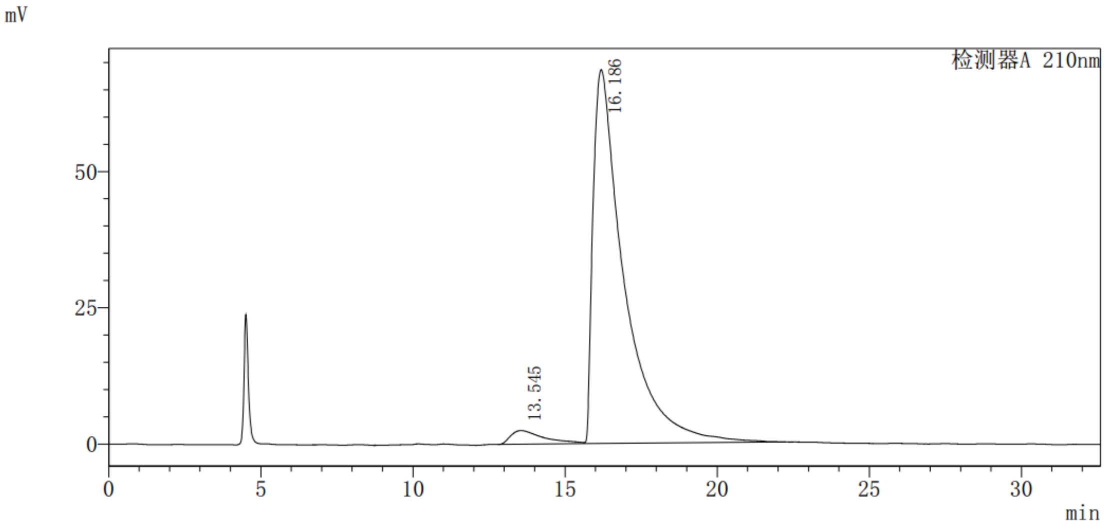

	Retention Time (min)	Relative Area (%)
**Peak 1**	13.545	3.767
**Peak 2**	16.186	96.223

Supplementary Figure 98. HPLC spectra of compound **3**

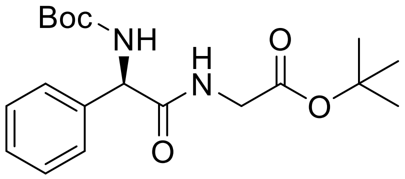

Tert-butyl (_R_)-(2-((tert-butoxycarbonyl)amino)-2-phenylacetyl)glycinate (5)

HPLC conditions: DAICEL Chiralpak IC-3 column, *n*-Hexane/*i*-PrOH = 80/20, 210 nm, 0.6 mL/min, t_{major} = 16.604 min, t_{minor} = 24.156 min.

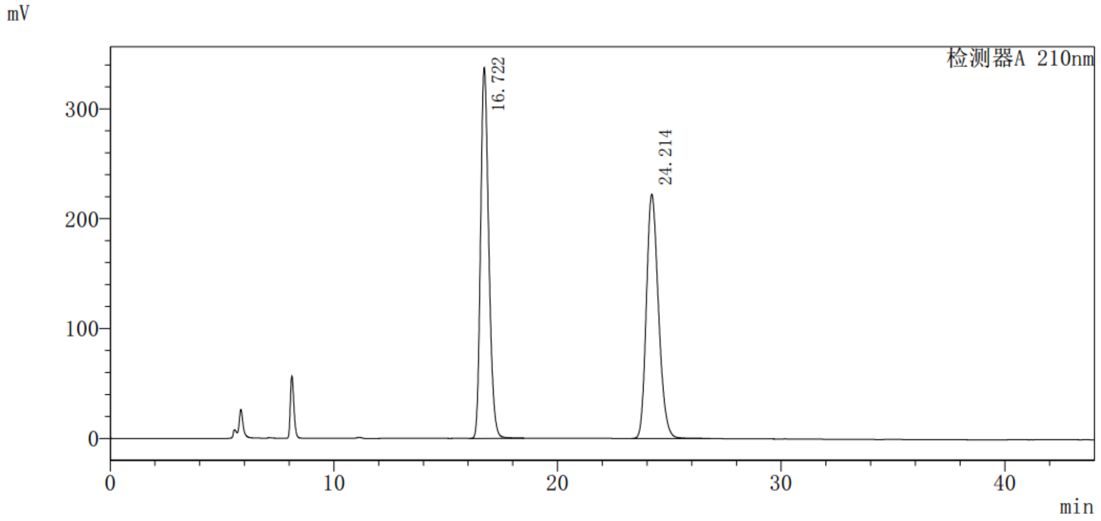

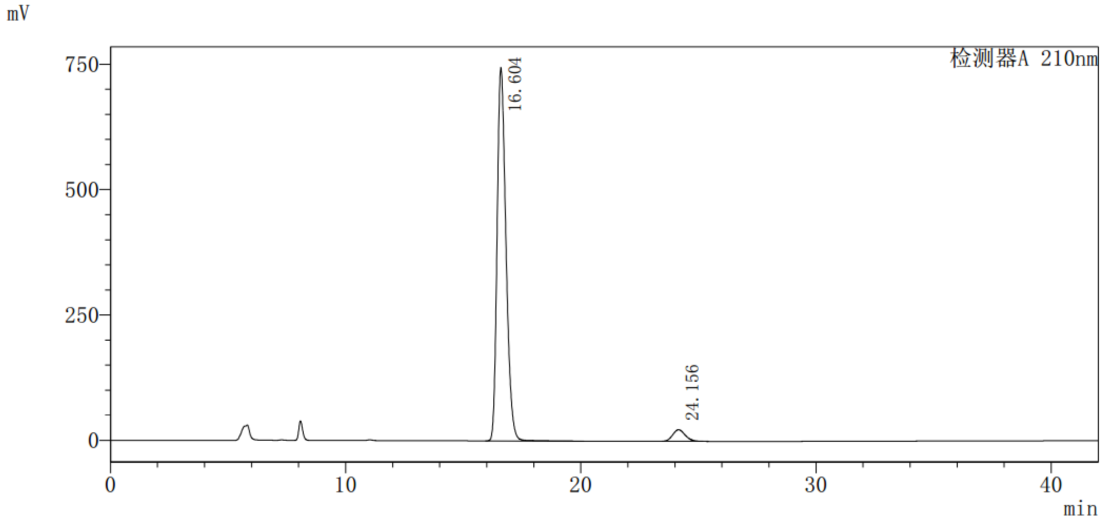

	Retention Time (min)	Relative Area (%)
**Peak 1**	16.604	95.829
**Peak 2**	24.156	4.172

Supplementary Figure 99. HPLC spectra of compound **5**

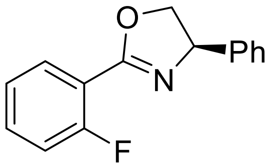

(*R*)-2-(2-Fluorophenyl)-4-phenyl-4,5-dihydrooxazole (7)

HPLC conditions: DAICEL Chiralpak OC-H column, *n*-Hexane/*i*-PrOH = 90/10, 254 nm, 0.8 mL/min, t_{major} = 18.447 min, t_{minor} = 21.848 min.

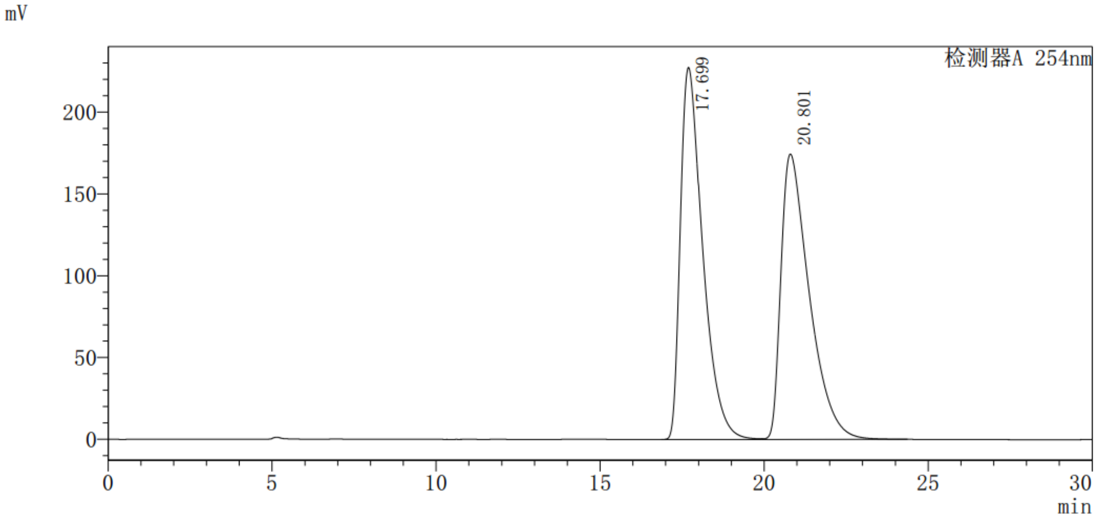

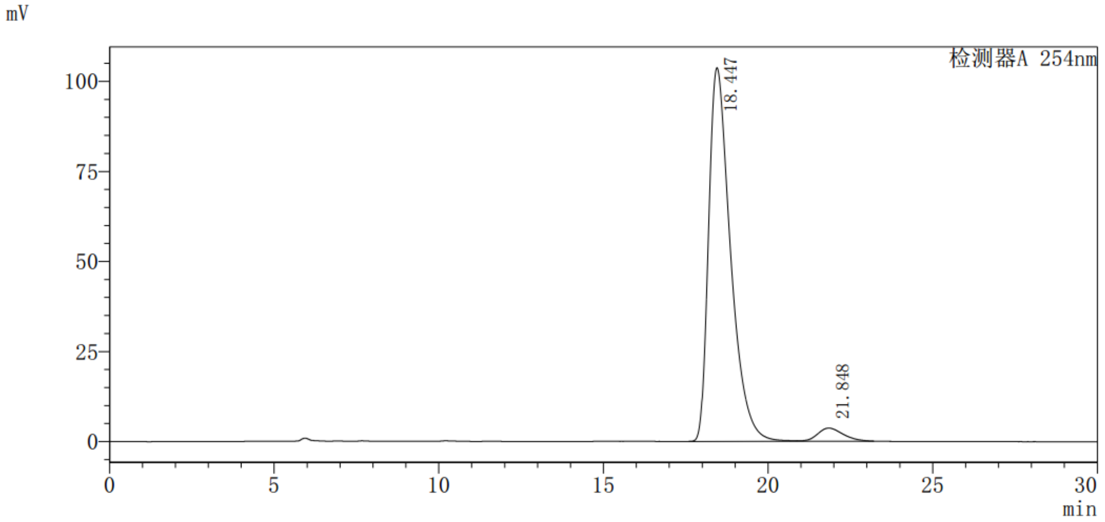

	Retention Time (min)	Relative Area (%)
**Peak 1**	18.447	96.058
**Peak 2**	21.848	3.942

Supplementary Figure 100. HPLC spectra of compound **7**

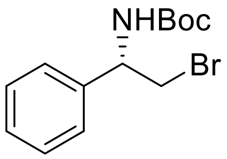

Tert-butyl (_S_)-(2-bromo-1-phenylethyl)carbamate (9)

HPLC conditions: DAICEL Chiralpak AS-H column, n-Hexane/i-PrOH = 95/5, 210 nm, 0.8 mL/min, t_{major} = 8.476 min, t_{minor} = 11.593 min.

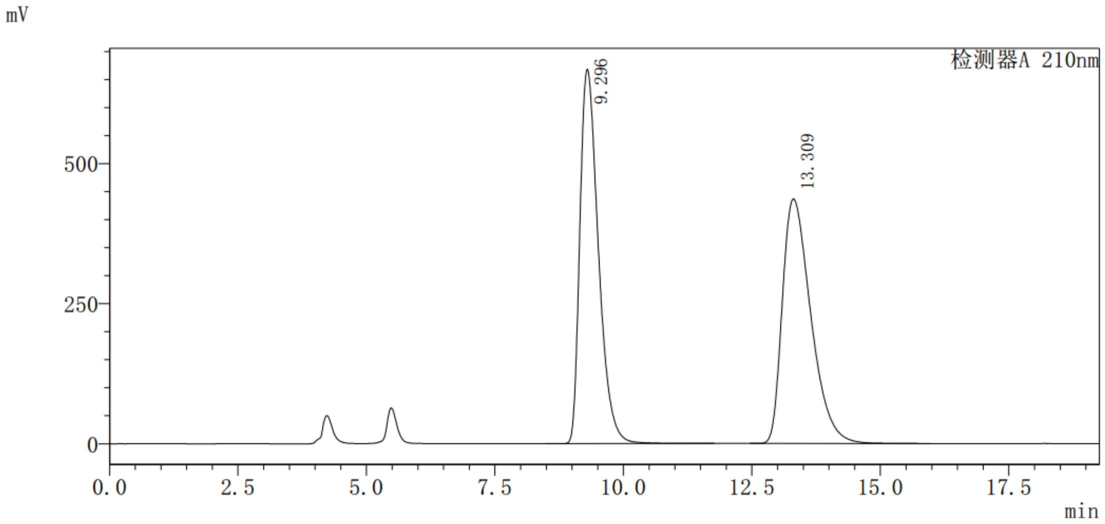

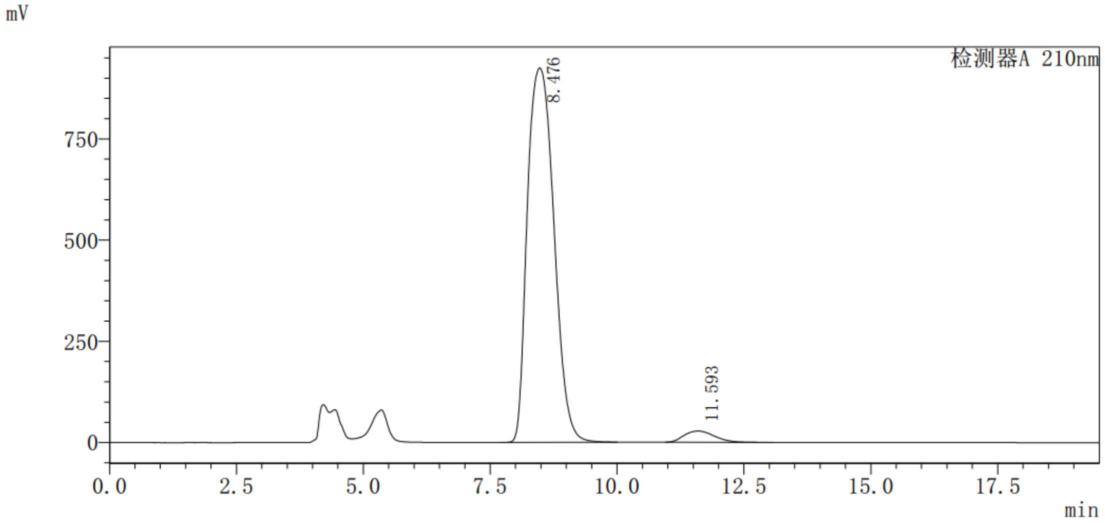

	Retention Time (min)	Relative Area (%)
**Peak 1**	8.476	96.673
**Peak 2**	11.593	3.327

Supplementary Figure 101. HPLC spectra of compound **9**

Supplementary References

1. Guo, H., Li, J., Liu, D. & Zhang, W. The synthesis of chiral α-aryl α-hydroxy carboxylic acids via RuPHOX-Ru catalyzed asymmetric hydrogenation. *Adv. Synth. Catal.* **359**, 3665-3673 (2017).
2. Chen, J., Li, F., Wang, F., Hu, Y., Zhang, Z., Zhao, M. & Zhang, W. $Pd(OAc)_2$-catalyzed asymmetric hydrogenation of α-iminoesters. *Org. Lett.* **21**, 9060-9065 (2019).
3. Shang, G., Yang, Q. & Zhang, X. Rh-catalyzed asymmetric hydrogenation of α-aryl imino esters: an efficient enantioselective synthesis of aryl glycine derivatives. *Angew. Chem. Int. Ed.* **45**, 6360-6362 (2006).
4. Kang, Q., Zhao, Z. A. & You, S.-L. Highly enantioselective transfer hydrogenation of α-imino esters by a phosphoric acid. *Adv. Synth. Catal.* **349**, 1657-1660 (2007).
5. Curto, J. M., Dickstein, J. S., Berritt, S. & Kozlowski, M. C. Asymmetric Synthesis of α-Allyl-α-Aryl α-Amino Acids by Tandem Alkylation/π-Allylation of α-Iminoesters. *Org. Lett.* **16**, 1948–1951 (2014)
6. Hua, X.& Hua, X. Ir-catalyzed Asymmetric Hydrogenation of α-Imino Esters with Chiral Ferrocenylphosphine-Phosphoramidite Ligands. *Adv. Synth. Catal.* **361**, 5063–5068 (2019)
7. Lohans, C. T., Chan, H. T. H., Malla, T. R., Kumar, K., Kamps, J. J. A. G., McArdle, D. J. B., Groesen, E., Munnik, M., Tooke, C. L., Spencer, J., Paton, R. S., Brem, J. & Schofield, C. J. Non-hydrolytic β-lactam antibiotic fragmentation by L,D -transpeptidases and serine β-lactamase cysteine variants. *Angew. Chem. Int. Ed.* **58**, 1990-1994 (2019).
8. Chen, J., Lu, X., Lou, W., Ye, Y.; Jiang, H. & Zeng, W. Palladium(II)-catalyzed enantioselective arylation of α-imino esters. *J. Org. Chem.* **77**, 8541-8548 (2012).
9. Wang, H.Y., Huang, K., Jesús, M. D., Espinosa, S., Piñero-Santiago, L. E., Barnes, C. L. & Ortiz-Marciales, M. Synthesis of enantiopure 1,2-azido and 1,2-amino alcohols via regio- and stereoselective ring-opening of enantiopure epoxides by sodium azide in hot water. *Tetrahedron: Asymmetry* **27**, 91-100 (2016).
10. Sedinkin, S. L., Rath, N. P. & Bauer, E. B. Synthesis and structural characterization of new phosphinooxazoline complexes of iron. *Journal of Organometallic Chemistry* **693**, 3081-3091 (2008).
11. Yan, S., Appleby, T., Larson, G., Wu, J. Z., Hamatake, R. K., Hong, Z. & Yao, N. Thiazolone-acylsulfonamides as novel HCV NS5B polymerase allosteric inhibitors: convergence of structure-based drug design and X-ray crystallographic study. *Bioorg. Med. Chem. Lett.* **17**, 1991-1995 (2007).
12. Li, B., Chen, J., Zhang, Z., Gridnev, I. D. & Zhang, W. Nickel-catalyzed asymmetric hydrogenation of *N*-sulfonyl imines. *Angew. Chem. Int. Ed.* **58**, 7329-7334 (2019).
13. Hu, Y., Chen, J., Li, B., Zhang, Z., Gridnev, I. D. & Zhang, W. Nickel-catalyzed asymmetric hydrogenation of 2-amidoacrylates. *Angew. Chem. Int. Ed.* **59**, 5371-5375 (2020).
14. Gridnev, I. D. & Dub, P. A. Enantioselection in Asymmetric Catalysis. (CRC Press, Boca Raton, London, New York, 2017).
15. Chai, J.-D. & Head_Gordon, M. Long-range corrected hybrid density functionals with damped atom-atom dispersion corrections. *Phys. Chem. Chem. Phys.* **10**, 6615-6620 (2008).
16. Frisch, M. J., Trucks, G. W., Schlegel, H. B., Scuseria, G. E., Robb, M. A., Cheeseman, J. R., Scalmani, G., Barone, V., Mennucci, B., Petersson, G. A., Nakatsuji, H., Caricato, M., Li, X., Hratchian, H. P., Izmaylov, A. F., Bloino, J., Zheng, G., Sonnenberg, J. L., Hada, M., Ehara, M., Toyota, K., Fukuda, R., Hasegawa, J., Ishida,

M., Nakajima, T., Honda, Y., Kitao, O., Nakai, H., Vreven, T., Montgomery, J. A., Peralta, Jr., Ogliaro, J. E., Bearpark, F, M., Heyd, J. J., Brothers, E., Kudin, K. N., Staroverov, V. N., Kobayashi, R., Normand, J., Raghavachari, K., Rendell, A., Burant, J. C., Iyengar, S. S., Tomasi, J., Cossi, M., Rega, N., Millam, J. M., Klene, M., Knox, J. E., Cross, J. B., Bakken, V., Adamo, C., Jaramillo, J., Gomperts, R., Stratmann, R. E., Yazyev, O., Austin, A. J., Cammi, R., Pomelli, C., Ochterski, J. W., Martin, R. L., Morokuma, K., Zakrzewski, V. G., Voth, G. A., Salvador, P., Dannenberg, J. J., Dapprich, S., Daniels, A. D., Farkas, O., Foresman, J. B., Ortiz, J. V., Cioslowski, J. & Fox, D. J. *Gaussian 09, Revision D.01*, Gaussian, Inc.: Wallingford, CT (2009).

17. Ditchfield, R., Hehre, W. J. & Pople, J. A. Self-consistent molecular-orbital methods. IX. An extended gaussian-type basis for molecular-orbital studies of organic molecules. *J. Chem. Phys.* **54**, 724-728 (1971).
18. Hehre, W. J., Ditchfield, R. & Pople, J. A. Self-consistent molecular orbital methods. XII. Further extensions of gaussian-type basis sets for use in molecular orbital studies of organic molecules. *J. Chem. Phys.* **56**, 2257-2261 (1972).
19. Hariharan, P. C. & Pople, J. A. The influence of polarization functions on MO hydrogenation energies. *Theor. Chim. Acta.* **28**, 213-222 (1973).
20. Hariharan, P. C. & Pople, J. A. Accuracy of AHn equilibrium geometries by single determinant molecular orbital theory. *Mol. Phys.* **27**, 209-214 (1974).
21. Gordon, M. S. The isomers of silacyclopropane. *Chem. Phys. Lett.* **76**, 163-168 (1980).
22. Marenich, A. V., Cramer, C. J. & Truhlar, D. G. Universal solvation model based on solute electron density and on a continuum model of the solvent defined by the bulk dielectric constant and atomic surface tensions. *J. Phys. Chem. B* **113**, 6378-6396 (2009).
