# Supplementary material for: Ni-catalyzed asymmetric hydrogenation of N-aryl imino esters for the efficient synthesis of chiral α-aryl glycines
Source: Nat Commun. 2020 Nov 23;11:5935. doi: 10.1038/s41467-020-19807-5 (PMC7683563; doi:10.1038/s41467-020-19807-5)
Supplement: Supplementary file 2 — Description of Additional Supplementary Files [file 41467_2020_19807_MOESM2_ESM.pdf]

Description of Additional Supplementary Files

File Name: Supplementary Data 1

Description: Cartesian coordinates and energy values (a.u.) of the computed intermediates
