# Supplementary material for: Ni-catalyzed asymmetric hydrogenation of N-aryl imino esters for the efficient synthesis of chiral α-aryl glycines
Source: Nat Commun. 2020 Nov 23;11:5935. doi: 10.1038/s41467-020-19807-5 (PMC7683563; doi:10.1038/s41467-020-19807-5)
Supplement: Supplementary file 3 — Supplementary Data 1 [file 41467_2020_19807_MOESM3_ESM.zip › NCOMMS-20-34270B .docx]

# Cartesian coordinates and energy values (a.u.) of the computed intermediates

**Catalytic cycle *E(R)***

**13** ***E(R)***

Electronic energy -3716.714214

EE+ Zero-point Energy -3716.010311

EE + Thermal Free Energy Correction -3716.083181

15 1.210066000 0.130403000 1.361253000

6 3.605002000 -0.129775000 0.006152000

6 3.030426000 0.304084000 1.209978000

15 2.441336000 -0.394554000 -1.379890000

6 2.715378000 1.082210000 -2.500249000

6 2.323047000 2.337464000 -1.707667000

1 1.278355000 2.299556000 -1.381656000

1 2.954102000 2.474303000 -0.823779000

6 4.173496000 1.203604000 -2.960646000

1 4.251563000 2.049893000 -3.652325000

1 4.854055000 1.399643000 -2.128413000

1 4.518561000 0.312427000 -3.493444000

6 1.810366000 0.940293000 -3.732139000

1 0.750609000 0.967218000 -3.465523000

1 2.008245000 1.773799000 -4.415020000

1 2.003789000 0.010303000 -4.277306000

6 3.059091000 -1.877816000 -2.232670000

1 4.075740000 -1.746624000 -2.611346000

1 3.057691000 -2.701297000 -1.514295000

1 2.394769000 -2.131336000 -3.060993000

6 0.662860000 1.762210000 1.960207000

1 0.676826000 2.466116000 1.123992000

1 -0.358908000 1.701545000 2.341002000

1 1.313989000 2.138408000 2.753319000

6 0.947759000 -1.083455000 2.762302000

6 1.389245000 -0.556696000 4.131635000

1 1.130573000 -1.300189000 4.894258000

1 2.468315000 -0.398645000 4.187204000

1 0.883811000 0.377090000 4.396928000

6 1.729777000 -2.355337000 2.411692000

1 1.510614000 -3.133050000 3.151602000

1 1.449603000 -2.741969000 1.427322000

1 2.810272000 -2.181646000 2.417081000

6 -0.557297000 -1.389830000 2.809012000

1 -0.894796000 -1.891623000 1.900015000

1 -0.763581000 -2.058824000 3.651893000

1 -1.153618000 -0.483000000 2.950759000

6 3.861530000 0.754854000 2.240347000

6 4.994153000 -0.229469000 -0.108360000

6 5.243995000 0.686800000 2.106825000

1 3.436057000 1.161922000 3.150953000

6 5.810326000 0.162873000 0.946626000

1 5.443396000 -0.602564000 -1.023102000

1 5.881082000 1.031084000 2.915365000

1 6.888565000 0.080264000 0.854582000

6 -2.239236000 -1.425675000 -0.473616000

7 -1.426330000 -0.430791000 -0.403569000

1 2.444270000 3.219822000 -2.345640000

28 0.481121000 -0.506454000 -0.626034000

1 0.293596000 -0.777030000 -2.065636000

6 -1.902759000 0.889772000 -0.126335000

6 -1.506495000 1.926480000 -0.962781000

6 -2.675655000 1.164589000 1.004659000

6 -1.884961000 3.238330000 -0.693910000

1 -0.916769000 1.708144000 -1.847747000

6 -3.040821000 2.468427000 1.287593000

1 -2.974702000 0.362574000 1.670587000

6 -2.651432000 3.513512000 0.440128000

1 -1.574931000 4.024428000 -1.371351000

1 -3.629674000 2.698835000 2.169206000

8 -3.065761000 4.751557000 0.803550000

6 -1.646659000 -2.784979000 -0.753022000

8 -2.290490000 -3.692279000 -1.233947000

8 -0.360311000 -2.859075000 -0.444520000

6 0.314975000 -4.073580000 -0.818203000

1 1.346340000 -3.943668000 -0.498363000

1 -0.140181000 -4.923914000 -0.308075000

1 0.262999000 -4.208308000 -1.899873000

6 -3.717429000 -1.366443000 -0.399431000

6 -4.392398000 -2.208089000 0.489166000

6 -4.439223000 -0.490757000 -1.216116000

6 -5.778445000 -2.151158000 0.581235000

1 -3.831454000 -2.895533000 1.115508000

6 -5.825690000 -0.451485000 -1.132212000

1 -3.916996000 0.153226000 -1.916357000

6 -6.495935000 -1.274318000 -0.229062000

1 -6.298701000 -2.796085000 1.281903000

1 -6.384400000 0.223266000 -1.772609000

1 -7.578481000 -1.234690000 -0.159692000

6 -2.694447000 5.851380000 -0.016156000

1 -3.102026000 5.748912000 -1.027865000

1 -3.123820000 6.734520000 0.457112000

1 -1.605642000 5.961481000 -0.067238000

**TS1** ***E(R)***

Electronic energy -3716.690367

EE+ Zero-point Energy -3715.987884

EE + Thermal Free Energy Correction -3716.060712

Imaginary frequency *i*30.3

15 1.444204000 1.379757000 0.580589000

6 3.620315000 -0.034248000 -0.379673000

6 3.203297000 1.221676000 0.090263000

15 2.306229000 -1.251805000 -0.754602000

6 2.302698000 -1.456541000 -2.611096000

6 1.731747000 -0.157508000 -3.197744000

1 0.699742000 0.011222000 -2.870032000

1 2.329717000 0.713853000 -2.908172000

6 3.698855000 -1.713307000 -3.189143000

1 3.600760000 -1.912080000 -4.262252000

1 4.357668000 -0.849288000 -3.072796000

1 4.181281000 -2.584352000 -2.734368000

6 1.391000000 -2.642454000 -2.955918000

1 0.381502000 -2.514582000 -2.554758000

1 1.315292000 -2.729484000 -4.045347000

1 1.796788000 -3.585567000 -2.575483000

6 2.885404000 -2.806738000 -0.010222000

1 3.818726000 -3.152832000 -0.462968000

1 3.052850000 -2.627840000 1.055419000

1 2.118466000 -3.576041000 -0.125128000

6 0.928458000 2.993444000 -0.086941000

1 0.861738000 2.919698000 -1.175088000

1 -0.056557000 3.256851000 0.305305000

1 1.635075000 3.785605000 0.172650000

6 1.467247000 1.542722000 2.443917000

6 2.091890000 2.850730000 2.936256000

1 2.035704000 2.881250000 4.030682000

1 3.146591000 2.934352000 2.661872000

1 1.560863000 3.727963000 2.553811000

6 2.250718000 0.342310000 2.993244000

1 2.156608000 0.313280000 4.084585000

1 1.868125000 -0.605338000 2.597680000

1 3.315530000 0.407582000 2.748809000

6 0.004363000 1.469508000 2.910895000

1 -0.442451000 0.499039000 2.680616000

1 -0.035524000 1.611623000 3.996713000

1 -0.609544000 2.251926000 2.452067000

6 4.146291000 2.241444000 0.251249000

6 4.981304000 -0.284120000 -0.578169000

6 5.493901000 1.995662000 0.012927000

1 3.835810000 3.230868000 0.570866000

6 5.914909000 0.725546000 -0.375522000

1 5.316051000 -1.264735000 -0.901191000

1 6.219405000 2.792217000 0.144506000

1 6.969709000 0.526457000 -0.535819000

6 -1.912527000 -1.461505000 0.481535000

7 -1.379322000 -0.295260000 0.345544000

1 1.733420000 -0.218166000 -4.291725000

28 0.460822000 -0.500418000 -0.040478000

1 -0.095811000 -1.787854000 -0.449423000

6 -2.148959000 0.853279000 0.009307000

6 -1.831411000 1.585448000 -1.127142000

6 -3.171583000 1.287942000 0.856192000

6 -2.547409000 2.734472000 -1.450968000

1 -1.024235000 1.253594000 -1.774153000

6 -3.877212000 2.437876000 0.549494000

1 -3.404530000 0.727584000 1.756027000

6 -3.574081000 3.164836000 -0.608634000

1 -2.287919000 3.280524000 -2.349611000

1 -4.667993000 2.793152000 1.201744000

8 -4.327906000 4.273126000 -0.821311000

6 -1.098505000 -2.561397000 1.133794000

8 -1.172253000 -3.728228000 0.810294000

8 -0.412877000 -2.106567000 2.174813000

6 0.384163000 -3.078694000 2.871186000

1 0.880058000 -2.529955000 3.669571000

1 -0.256475000 -3.859214000 3.286170000

1 1.115609000 -3.517781000 2.190459000

6 -3.304378000 -1.824716000 0.139776000

6 -4.059861000 -2.621784000 1.008126000

6 -3.862311000 -1.398476000 -1.072245000

6 -5.371584000 -2.949155000 0.686432000

1 -3.630669000 -2.973200000 1.941502000

6 -5.165800000 -1.747860000 -1.397967000

1 -3.271997000 -0.804383000 -1.761136000

6 -5.925503000 -2.512888000 -0.514990000

1 -5.959685000 -3.551549000 1.370910000

1 -5.590617000 -1.420511000 -2.341184000

1 -6.948727000 -2.774470000 -0.765762000

6 -4.029920000 5.081327000 -1.950869000

1 -4.152850000 4.524944000 -2.886418000

1 -4.747492000 5.901806000 -1.925976000

1 -3.013098000 5.485463000 -1.892875000

**14** ***E(R)***

Electronic energy -3716.718372

EE+ Zero-point Energy -3716.011863

EE + Thermal Free Energy Correction -3716.084847

15 1.471172000 1.604358000 0.187128000

6 3.609146000 -0.021582000 -0.467427000

6 3.182947000 1.311897000 -0.382909000

15 2.319389000 -1.303495000 -0.350302000

6 2.198808000 -2.100055000 -2.032171000

6 1.577767000 -1.053526000 -2.969421000

1 0.572551000 -0.758630000 -2.647335000

1 2.192026000 -0.147809000 -3.023445000

6 3.554052000 -2.550390000 -2.587431000

1 3.384500000 -3.098046000 -3.521283000

1 4.205267000 -1.703198000 -2.816471000

1 4.079206000 -3.221133000 -1.900346000

6 1.283867000 -3.326889000 -1.897349000

1 0.325921000 -3.096530000 -1.421504000

1 1.071302000 -3.724249000 -2.895672000

1 1.767369000 -4.121708000 -1.320916000

6 2.951867000 -2.531322000 0.830581000

1 3.908291000 -2.948016000 0.502966000

1 3.084543000 -2.037667000 1.797013000

1 2.226654000 -3.339353000 0.949173000

6 0.832467000 2.919503000 -0.889177000

1 0.855239000 2.562919000 -1.922017000

1 -0.199060000 3.154632000 -0.621492000

1 1.439607000 3.825122000 -0.807405000

6 1.616493000 2.285285000 1.921763000

6 2.635169000 3.425774000 2.023101000

1 2.585624000 3.845540000 3.033890000

1 3.658106000 3.078158000 1.858054000

1 2.421931000 4.235964000 1.318452000

6 2.049151000 1.122092000 2.826925000

1 2.186996000 1.490714000 3.849218000

1 1.294048000 0.328526000 2.851610000

1 2.998486000 0.686867000 2.495668000

6 0.231107000 2.798739000 2.340264000

1 -0.532416000 2.021859000 2.252402000

1 0.277372000 3.116241000 3.387938000

1 -0.076788000 3.665209000 1.746272000

6 4.088089000 2.343844000 -0.648252000

6 4.956771000 -0.306458000 -0.705423000

6 5.417049000 2.049895000 -0.930810000

1 3.764358000 3.378892000 -0.621048000

6 5.857582000 0.727753000 -0.930554000

1 5.303704000 -1.334331000 -0.725581000

1 6.115421000 2.855228000 -1.134225000

1 6.902126000 0.502815000 -1.120085000

6 -1.246093000 -1.288767000 0.825111000

7 -1.171902000 0.137759000 0.894472000

1 1.497354000 -1.469070000 -3.979594000

28 0.465545000 -0.304021000 0.223562000

1 -0.284499000 -1.731915000 0.272655000

6 -2.179358000 0.903389000 0.251310000

6 -2.103182000 1.312094000 -1.079613000

6 -3.297898000 1.291094000 1.000041000

6 -3.098199000 2.100786000 -1.657069000

1 -1.250199000 1.011686000 -1.682047000

6 -4.303476000 2.057983000 0.434812000

1 -3.370753000 0.976157000 2.036769000

6 -4.206669000 2.473128000 -0.897746000

1 -2.997649000 2.401235000 -2.693385000

1 -5.172373000 2.353305000 1.014746000

8 -5.239918000 3.232366000 -1.358736000

6 -1.054089000 -1.940953000 2.195292000

8 -0.240467000 -2.817354000 2.412206000

8 -1.873324000 -1.444546000 3.106855000

6 -1.787106000 -2.026129000 4.419101000

1 -2.548000000 -1.520521000 5.010367000

1 -1.989839000 -3.097655000 4.369412000

1 -0.796055000 -1.851586000 4.842397000

6 -2.366155000 -1.913213000 0.015212000

6 -3.547332000 -2.341479000 0.621286000

6 -2.237329000 -1.995025000 -1.371970000

6 -4.582432000 -2.854633000 -0.156475000

1 -3.670946000 -2.267311000 1.696516000

6 -3.271420000 -2.506982000 -2.146975000

1 -1.324209000 -1.646496000 -1.846420000

6 -4.447176000 -2.940951000 -1.538989000

1 -5.497601000 -3.186673000 0.323428000

1 -3.156200000 -2.568905000 -3.224426000

1 -5.255597000 -3.343323000 -2.141347000

6 -5.191001000 3.674352000 -2.705500000

1 -5.174427000 2.831090000 -3.405529000

1 -6.101938000 4.252461000 -2.864195000

1 -4.320657000 4.315572000 -2.886434000

**15** ***E(R)***

Electronic energy -3717.893501

EE+ Zero-point Energy -3717.168492

EE + Thermal Free Energy Correction --3717.242401

15 1.160983000 -0.036586000 1.361565000

6 3.640649000 -0.171101000 0.162292000

6 2.975708000 0.184559000 1.342703000

15 2.589184000 -0.359131000 -1.303469000

6 2.925676000 1.144074000 -2.364736000

6 2.681026000 2.389526000 -1.500358000

1 1.664543000 2.413641000 -1.091327000

1 3.389189000 2.455278000 -0.668889000

6 4.361108000 1.151430000 -2.901849000

1 4.494622000 2.040354000 -3.528329000

1 5.100697000 1.202601000 -2.098909000

1 4.572869000 0.274179000 -3.520630000

6 1.941542000 1.127960000 -3.545206000

1 0.909206000 1.287041000 -3.217587000

1 2.197782000 1.943164000 -4.229980000

1 1.987498000 0.190618000 -4.109116000

6 3.169138000 -1.834622000 -2.188537000

1 4.248954000 -1.808872000 -2.353765000

1 2.918583000 -2.712411000 -1.586673000

1 2.657499000 -1.910483000 -3.151146000

6 0.522284000 1.420744000 2.236127000

1 0.679175000 2.302259000 1.609818000

1 -0.545368000 1.309364000 2.424528000

1 1.040478000 1.555498000 3.188588000

6 0.882205000 -1.538919000 2.440141000

6 1.591557000 -1.434596000 3.794909000

1 1.289303000 -2.293058000 4.405454000

1 2.679411000 -1.465875000 3.697753000

1 1.312067000 -0.527278000 4.339682000

6 1.432396000 -2.744473000 1.664316000

1 1.268281000 -3.656401000 2.248688000

1 0.935027000 -2.865219000 0.697927000

1 2.508958000 -2.648759000 1.486237000

6 -0.630054000 -1.672738000 2.672337000

1 -1.187719000 -1.593359000 1.737395000

1 -0.832539000 -2.645288000 3.135113000

1 -0.991101000 -0.897274000 3.356219000

6 3.719606000 0.577711000 2.458680000

6 5.035846000 -0.236111000 0.133932000

6 5.109127000 0.553358000 2.411532000

1 3.223601000 0.886335000 3.372637000

6 5.766267000 0.118731000 1.262121000

1 5.553480000 -0.555834000 -0.764573000

1 5.681200000 0.856388000 3.282512000

1 6.850132000 0.070826000 1.240109000

6 -2.260887000 -1.061906000 -1.132528000

7 -1.353211000 -0.339458000 -0.239437000

1 2.809115000 3.282421000 -2.121827000

28 0.473920000 -0.406249000 -0.702304000

6 -1.773587000 1.001865000 -0.023949000

6 -1.270265000 2.060750000 -0.785700000

6 -2.671259000 1.324894000 1.008953000

6 -1.590921000 3.392686000 -0.513654000

1 -0.608026000 1.848579000 -1.622364000

6 -3.017727000 2.637837000 1.279558000

1 -3.077816000 0.526874000 1.621546000

6 -2.469270000 3.683528000 0.527152000

1 -1.157488000 4.177425000 -1.122996000

1 -3.702055000 2.877711000 2.087741000

8 -2.855176000 4.940827000 0.887218000

6 -1.803074000 -2.490451000 -1.397220000

8 -1.894738000 -3.026323000 -2.486112000

8 -1.327773000 -3.110097000 -0.322095000

6 -0.947395000 -4.482750000 -0.495060000

1 -0.647211000 -4.828616000 0.492631000

1 -1.793602000 -5.067076000 -0.861738000

1 -0.111699000 -4.558631000 -1.194510000

6 -3.698818000 -1.120861000 -0.620730000

6 -4.036979000 -1.840366000 0.529374000

6 -4.691972000 -0.403384000 -1.286487000

6 -5.344773000 -1.836235000 1.002929000

1 -3.273491000 -2.398822000 1.061636000

6 -6.002117000 -0.395669000 -0.812319000

1 -4.434203000 0.165083000 -2.176221000

6 -6.331192000 -1.112270000 0.334304000

1 -5.595312000 -2.398863000 1.897296000

1 -6.763109000 0.173255000 -1.337870000

1 -7.350974000 -1.107637000 0.706995000

6 -2.320522000 6.031623000 0.155200000

1 -2.607011000 5.983727000 -0.901776000

1 -2.745443000 6.931261000 0.601837000

1 -1.227826000 6.070387000 0.234910000

1 -2.305645000 -0.610853000 -2.138551000

1 -0.066395000 -0.429270000 -2.240140000

1 0.264143000 -1.141657000 -2.140643000

**TS2** ***E(R)***

Electronic energy -3717.882751

EE+ Zero-point Energy -3717.160169

EE + Thermal Free Energy Correction -3717.233286

Imaginary frequency *i*1063.4

15 0.984669000 0.316633000 1.353356000

6 3.540660000 -0.257132000 0.479202000

6 2.806287000 0.319136000 1.524981000

15 2.628451000 -0.570040000 -1.064185000

6 3.193795000 0.758764000 -2.253340000

6 2.790183000 2.111953000 -1.650365000

1 1.707904000 2.180283000 -1.493522000

1 3.289187000 2.296012000 -0.693567000

6 4.709545000 0.720352000 -2.480203000

1 4.970316000 1.487269000 -3.217789000

1 5.267264000 0.940794000 -1.566886000

1 5.044364000 -0.244516000 -2.873236000

6 2.478244000 0.550221000 -3.595949000

1 1.396206000 0.677956000 -3.507354000

1 2.843912000 1.297679000 -4.308057000

1 2.679284000 -0.438845000 -4.020208000

6 3.201861000 -2.182998000 -1.670487000

1 4.283678000 -2.194415000 -1.824806000

1 2.939868000 -2.936755000 -0.923865000

1 2.700444000 -2.423393000 -2.610457000

6 0.507245000 2.039083000 1.692103000

1 0.944953000 2.678583000 0.921794000

1 -0.578693000 2.147112000 1.661768000

1 0.874350000 2.354668000 2.672946000

6 0.325119000 -0.745928000 2.736975000

6 0.897175000 -0.402463000 4.114974000

1 0.347756000 -0.977373000 4.869230000

1 1.953097000 -0.670431000 4.200921000

1 0.780987000 0.658551000 4.358595000

6 0.668813000 -2.197014000 2.374035000

1 0.359391000 -2.860558000 3.188977000

1 0.148077000 -2.508892000 1.464759000

1 1.745864000 -2.330570000 2.219909000

6 -1.196227000 -0.544805000 2.752800000

1 -1.623926000 -0.669385000 1.756349000

1 -1.650754000 -1.288040000 3.417445000

1 -1.465733000 0.447933000 3.126727000

6 3.481114000 0.809463000 2.647025000

6 4.918425000 -0.449077000 0.613297000

6 4.857758000 0.649747000 2.758674000

1 2.935617000 1.310500000 3.439119000

6 5.571295000 -0.007550000 1.758301000

1 5.485061000 -0.930455000 -0.177180000

1 5.373482000 1.027123000 3.635842000

1 6.641113000 -0.157767000 1.861345000

6 -2.316048000 -1.202933000 -1.317988000

7 -1.409243000 -0.214156000 -0.725304000

1 3.080643000 2.912236000 -2.339607000

28 0.515781000 -0.425961000 -0.667675000

6 -1.908807000 1.104859000 -0.573701000

6 -1.347023000 2.161498000 -1.296063000

6 -2.909219000 1.418349000 0.362489000

6 -1.725925000 3.486860000 -1.084222000

1 -0.583715000 1.951609000 -2.041025000

6 -3.311410000 2.728972000 0.565600000

1 -3.361329000 0.632399000 0.955544000

6 -2.716011000 3.774816000 -0.147245000

1 -1.248595000 4.271081000 -1.660130000

1 -4.080846000 2.963300000 1.294829000

8 -3.168222000 5.027343000 0.143759000

6 -1.581722000 -2.493105000 -1.659795000

8 -1.865293000 -3.191875000 -2.609872000

8 -0.624484000 -2.799594000 -0.784224000

6 0.115961000 -4.003368000 -1.039659000

1 0.841557000 -4.074496000 -0.230884000

1 -0.553530000 -4.865261000 -1.027550000

1 0.621979000 -3.934820000 -2.004472000

6 -3.521898000 -1.570242000 -0.458800000

6 -3.385385000 -2.332808000 0.704211000

6 -4.786980000 -1.114141000 -0.829033000

6 -4.495004000 -2.612130000 1.495093000

1 -2.409244000 -2.706090000 0.997822000

6 -5.899024000 -1.394051000 -0.038835000

1 -4.897978000 -0.522901000 -1.733869000

6 -5.754007000 -2.139675000 1.127868000

1 -4.376847000 -3.202235000 2.398826000

1 -6.877148000 -1.027025000 -0.334302000

1 -6.618972000 -2.357268000 1.746937000

6 -2.588620000 6.118776000 -0.552761000

1 -2.763285000 6.043153000 -1.632188000

1 -3.080527000 7.014262000 -0.171358000

1 -1.511571000 6.189470000 -0.361349000

1 -2.705734000 -0.845065000 -2.282186000

1 -0.445495000 -0.292314000 -1.872791000

1 0.346571000 -0.703571000 -2.199002000

**16** ***E(R)***

Electronic energy -3717.929516

EE+ Zero-point Energy -3717.201210

EE + Thermal Free Energy Correction -3717.275514

15 1.341281000 1.315128000 0.334961000

6 3.767196000 0.109003000 -0.191594000

6 3.118308000 1.350375000 -0.113832000

15 2.665830000 -1.351665000 -0.286505000

6 2.884598000 -1.978173000 -2.043209000

6 2.485650000 -0.837151000 -2.991474000

1 1.452415000 -0.510199000 -2.824630000

1 3.139608000 0.033497000 -2.880158000

6 4.329303000 -2.406009000 -2.329570000

1 4.377894000 -2.818295000 -3.343667000

1 5.024609000 -1.564794000 -2.287402000

1 4.674140000 -3.182525000 -1.639606000

6 1.958750000 -3.182414000 -2.267107000

1 0.905344000 -2.903082000 -2.186060000

1 2.130569000 -3.578654000 -3.273987000

1 2.155034000 -3.988133000 -1.551993000

6 3.386878000 -2.578369000 0.844223000

1 4.460826000 -2.703027000 0.686543000

1 3.213120000 -2.236282000 1.868141000

1 2.886258000 -3.539614000 0.707982000

6 0.617963000 2.733334000 -0.547887000

1 0.655326000 2.522709000 -1.619837000

1 -0.426229000 2.857505000 -0.253951000

1 1.150202000 3.666761000 -0.349404000

6 1.369566000 1.756575000 2.158022000

6 2.105311000 3.066138000 2.455781000

1 2.017080000 3.282214000 3.526938000

1 3.170447000 3.004468000 2.218976000

1 1.675548000 3.913699000 1.912605000

6 2.055518000 0.597176000 2.895274000

1 2.036358000 0.789425000 3.973959000

1 1.544948000 -0.354210000 2.710932000

1 3.101893000 0.486812000 2.592423000

6 -0.086349000 1.878378000 2.622486000

1 -0.643743000 0.960601000 2.429134000

1 -0.105469000 2.067809000 3.701894000

1 -0.604531000 2.706886000 2.129766000

6 3.864440000 2.525546000 -0.238361000

6 5.161644000 0.058418000 -0.271819000

6 5.247058000 2.465976000 -0.376205000

1 3.375497000 3.493634000 -0.205733000

6 5.898381000 1.234516000 -0.360848000

1 5.677706000 -0.896148000 -0.272730000

1 5.819319000 3.383351000 -0.471166000

1 6.980580000 1.189498000 -0.430783000

6 -1.985786000 -1.985219000 0.014344000

7 -1.263927000 -0.774086000 -0.506454000

1 2.563388000 -1.185620000 -4.027334000

28 0.671867000 -0.763742000 -0.000106000

6 -2.007252000 0.466226000 -0.563469000

6 -2.093102000 1.132180000 -1.779021000

6 -2.620785000 1.015410000 0.561237000

6 -2.752862000 2.355020000 -1.884187000

1 -1.628009000 0.703669000 -2.663747000

6 -3.284707000 2.227092000 0.467559000

1 -2.582128000 0.494530000 1.509559000

6 -3.348405000 2.910031000 -0.752199000

1 -2.793236000 2.852267000 -2.845659000

1 -3.764141000 2.662024000 1.338446000

8 -4.015477000 4.094712000 -0.733438000

6 -1.562076000 -2.381305000 1.417415000

8 -1.589057000 -3.535273000 1.792662000

8 -1.210147000 -1.363368000 2.193265000

6 -0.792368000 -1.706671000 3.525245000

1 -0.577019000 -0.761244000 4.019204000

1 -1.593692000 -2.235062000 4.044681000

1 0.103438000 -2.330015000 3.484484000

6 -3.494877000 -1.927278000 -0.133622000

6 -4.351514000 -1.763379000 0.954721000

6 -4.032440000 -2.012008000 -1.420080000

6 -5.728234000 -1.686256000 0.758926000

1 -3.954144000 -1.689015000 1.963224000

6 -5.405702000 -1.924921000 -1.617383000

1 -3.367268000 -2.141690000 -2.270177000

6 -6.257292000 -1.762640000 -0.525967000

1 -6.385939000 -1.560822000 1.613264000

1 -5.811924000 -1.989913000 -2.621915000

1 -7.330257000 -1.698353000 -0.677805000

6 -4.089188000 4.835066000 -1.942385000

1 -4.625609000 4.279348000 -2.719722000

1 -4.642462000 5.743893000 -1.703884000

1 -3.091100000 5.103885000 -2.306689000

1 -1.620643000 -2.808562000 -0.603479000

1 -1.074034000 -1.013114000 -1.480552000

1 0.630280000 -2.235226000 -0.068776000

**Catalytic cycle *E(S)***

**13** ***E(S)***

Electronic energy -3716.712432

EE+ Zero-point Energy -3716.008537

EE + Thermal Free Energy Correction -3716.083262

15 -1.437526000 0.222755000 1.439219000

6 -3.570839000 0.767832000 -0.228911000

6 -3.174434000 0.712254000 1.115803000

15 -2.441286000 -0.045593000 -1.420808000

6 -3.344277000 -1.606511000 -1.930382000

6 -3.654000000 -2.397770000 -0.651096000

1 -2.741512000 -2.642341000 -0.097577000

1 -4.328277000 -1.851041000 0.015132000

6 -4.643556000 -1.309155000 -2.687998000

1 -5.106060000 -2.258464000 -2.980722000

1 -5.367533000 -0.771470000 -2.070980000

1 -4.465925000 -0.733516000 -3.601819000

6 -2.414496000 -2.432896000 -2.831413000

1 -1.520074000 -2.763034000 -2.296025000

1 -2.952677000 -3.323516000 -3.174968000

1 -2.098060000 -1.871949000 -3.717353000

6 -2.400830000 1.065292000 -2.860838000

1 -3.401813000 1.357141000 -3.187294000

1 -1.844909000 1.965068000 -2.582586000

1 -1.881481000 0.575918000 -3.688133000

6 -1.580221000 -1.075759000 2.711000000

1 -2.190599000 -1.886749000 2.305710000

1 -0.591023000 -1.473422000 2.949351000

1 -2.046816000 -0.701749000 3.626316000

6 -0.637601000 1.692307000 2.272628000

6 -1.457667000 2.311961000 3.408499000

1 -0.841341000 3.065433000 3.912712000

1 -2.355065000 2.813558000 3.038024000

1 -1.753844000 1.572741000 4.159413000

6 -0.409898000 2.750689000 1.184599000

1 0.025662000 3.651156000 1.632055000

1 0.270584000 2.398725000 0.406064000

1 -1.354263000 3.035140000 0.706380000

6 0.692627000 1.181496000 2.849102000

1 1.232043000 0.532952000 2.155241000

1 1.341927000 2.030673000 3.088776000

1 0.526846000 0.618045000 3.772742000

6 -4.088038000 1.060927000 2.115715000

6 -4.820105000 1.298831000 -0.563018000

6 -5.346362000 1.545869000 1.776664000

1 -3.822211000 0.960475000 3.162512000

6 -5.698954000 1.698427000 0.437285000

1 -5.116546000 1.394912000 -1.602346000

1 -6.047561000 1.820122000 2.558369000

1 -6.667932000 2.109614000 0.172662000

6 1.961847000 -1.524144000 0.096338000

7 1.325573000 -0.465869000 -0.270038000

1 -4.142475000 -3.340530000 -0.921494000

28 -0.589592000 -0.397358000 -0.504129000

1 -0.362546000 -0.719839000 -1.923399000

6 2.016193000 0.743576000 -0.596076000

6 2.981650000 1.289951000 0.246291000

6 1.626315000 1.447503000 -1.739979000

6 3.558681000 2.521894000 -0.040423000

1 3.278018000 0.771296000 1.150303000

6 2.217726000 2.658815000 -2.047396000

1 0.864094000 1.033226000 -2.389834000

6 3.181776000 3.210713000 -1.195207000

1 4.293231000 2.927225000 0.644430000

1 1.932066000 3.202339000 -2.941848000

8 3.683639000 4.411525000 -1.569993000

6 4.640331000 5.033619000 -0.722466000

1 4.888447000 5.981765000 -1.199541000

1 4.222262000 5.225557000 0.271602000

1 5.547510000 4.426534000 -0.631150000

6 3.434280000 -1.703127000 0.105372000

6 4.106971000 -2.028518000 1.285592000

6 4.148218000 -1.543315000 -1.085501000

6 5.490002000 -2.174949000 1.275041000

1 3.551877000 -2.152455000 2.210049000

6 5.528938000 -1.705141000 -1.092097000

1 3.622236000 -1.293863000 -2.002085000

6 6.201254000 -2.016101000 0.087700000

1 6.012067000 -2.415334000 2.195674000

1 6.078460000 -1.586024000 -2.020303000

1 7.279907000 -2.138006000 0.081407000

6 1.064954000 -2.682791000 0.437912000

8 -0.145651000 -2.625237000 0.312378000

8 1.718755000 -3.745534000 0.856403000

6 0.918181000 -4.894095000 1.189671000

1 0.374343000 -5.237249000 0.307625000

1 1.626094000 -5.649747000 1.523095000

1 0.218650000 -4.641802000 1.988751000

**TS1** ***E(S)***

Electronic energy -3716.688347

EE+ Zero-point Energy -3715.985146

EE + Thermal Free Energy Correction -3716.057791

Imaginary frequency *i*40.7

15 -1.629955000 1.152302000 1.079014000

6 -3.525704000 0.328532000 -0.758672000

6 -3.347045000 1.000119000 0.461365000

15 -2.115227000 -0.682538000 -1.350477000

6 -2.710046000 -2.455771000 -1.339436000

6 -2.855656000 -2.852621000 0.135270000

1 -1.881558000 -2.872148000 0.629362000

1 -3.511503000 -2.165103000 0.681012000

6 -4.047919000 -2.652068000 -2.062513000

1 -4.257557000 -3.726162000 -2.116418000

1 -4.877007000 -2.183375000 -1.527694000

1 -4.027967000 -2.268650000 -3.087721000

6 -1.645897000 -3.331421000 -2.015719000

1 -0.669046000 -3.234423000 -1.534666000

1 -1.955119000 -4.379636000 -1.938891000

1 -1.538706000 -3.095343000 -3.079382000

6 -1.874668000 -0.172313000 -3.079934000

1 -2.770351000 -0.358187000 -3.678919000

1 -1.655504000 0.898901000 -3.093502000

1 -1.028413000 -0.709247000 -3.513611000

6 -1.757757000 0.804063000 2.861762000

1 -2.212654000 -0.181919000 2.984830000

1 -0.761349000 0.789507000 3.308894000

1 -2.373040000 1.542353000 3.383133000

6 -1.215617000 2.966193000 0.894229000

6 -2.241140000 3.915721000 1.521535000

1 -1.854163000 4.939183000 1.454133000

1 -3.198477000 3.891631000 0.995667000

1 -2.417615000 3.700609000 2.580180000

6 -1.121169000 3.245014000 -0.612240000

1 -0.870340000 4.299175000 -0.774774000

1 -0.345930000 2.639025000 -1.088972000

1 -2.073551000 3.047979000 -1.116492000

6 0.144805000 3.182360000 1.572946000

1 0.912459000 2.504667000 1.192219000

1 0.483166000 4.207135000 1.383867000

1 0.075032000 3.049891000 2.657586000

6 -4.443586000 1.611959000 1.076735000

6 -4.758246000 0.404775000 -1.414832000

6 -5.682107000 1.629081000 0.446019000

1 -4.329929000 2.088941000 2.044692000

6 -5.829180000 1.059569000 -0.817223000

1 -4.888721000 -0.052982000 -2.389444000

1 -6.528037000 2.107750000 0.929067000

1 -6.784327000 1.111147000 -1.329938000

6 1.738978000 -1.412353000 0.671934000

7 1.383572000 -0.189302000 0.498634000

1 -3.286914000 -3.857090000 0.206225000

28 -0.437684000 -0.282815000 -0.115539000

1 0.177179000 -1.375421000 -0.870583000

6 2.295929000 0.773707000 -0.019224000

6 3.409083000 1.146365000 0.724498000

6 2.033482000 1.394760000 -1.241571000

6 4.271923000 2.133277000 0.255840000

1 3.600269000 0.670467000 1.681078000

6 2.893961000 2.368092000 -1.718710000

1 1.170329000 1.096898000 -1.831007000

6 4.015041000 2.747999000 -0.970949000

1 5.129299000 2.410200000 0.857050000

1 2.709299000 2.852589000 -2.671690000

8 4.785419000 3.721740000 -1.519258000

6 5.914140000 4.178084000 -0.787461000

1 6.363480000 4.964621000 -1.394242000

1 5.618141000 4.593784000 0.182040000

1 6.645031000 3.375626000 -0.637953000

6 3.019476000 -2.045049000 0.296514000

6 3.650655000 -2.904580000 1.202818000

6 3.578760000 -1.833995000 -0.969501000

6 4.848912000 -3.517350000 0.859450000

1 3.214383000 -3.078738000 2.182092000

6 4.764265000 -2.469641000 -1.315393000

1 3.079433000 -1.188478000 -1.683679000

6 5.404684000 -3.302693000 -0.399846000

1 5.344581000 -4.168980000 1.571329000

1 5.190067000 -2.313849000 -2.301112000

1 6.335310000 -3.791145000 -0.670910000

6 0.759686000 -2.322670000 1.387131000

8 0.536972000 -3.461438000 1.036208000

8 0.267407000 -1.743571000 2.471508000

6 -0.602614000 -2.541868000 3.293166000

1 -0.141324000 -3.509917000 3.495759000

1 -0.726899000 -1.978428000 4.215497000

1 -1.564540000 -2.680933000 2.796913000

**14** ***E(S)***

Electronic energy -3716.713283

EE+ Zero-point Energy -3716.006894

EE + Thermal Free Energy Correction -3716.080179

15 1.195932000 -1.687078000 0.595075000

6 3.538287000 -0.611511000 -0.398960000

6 3.015000000 -1.601696000 0.447288000

15 2.411065000 0.749592000 -0.850109000

6 3.135988000 2.319759000 -0.139932000

6 2.950147000 2.238611000 1.381970000

1 1.892865000 2.175558000 1.656269000

1 3.465788000 1.367407000 1.800917000

6 4.619728000 2.517426000 -0.467132000

1 4.918467000 3.519219000 -0.138503000

1 5.253713000 1.798149000 0.056542000

1 4.819618000 2.449166000 -1.541136000

6 2.342940000 3.498374000 -0.727762000

1 1.259572000 3.378641000 -0.638587000

1 2.613247000 4.411927000 -0.187557000

1 2.583910000 3.652847000 -1.784046000

6 2.461965000 0.888817000 -2.661897000

1 3.477156000 1.075697000 -3.022354000

1 2.091843000 -0.043623000 -3.094768000

1 1.810763000 1.704178000 -2.985528000

6 0.855701000 -2.174478000 2.309790000

1 1.309474000 -1.438724000 2.978607000

1 -0.224163000 -2.181282000 2.476827000

1 1.261016000 -3.165651000 2.530436000

6 0.689143000 -3.114114000 -0.510983000

6 1.672341000 -4.287795000 -0.429873000

1 1.259064000 -5.121455000 -1.008504000

1 2.649719000 -4.041114000 -0.851372000

1 1.812797000 -4.637556000 0.597942000

6 0.648853000 -2.577216000 -1.946912000

1 0.410486000 -3.394114000 -2.636656000

1 -0.111836000 -1.799354000 -2.068990000

1 1.619697000 -2.161683000 -2.239193000

6 -0.694768000 -3.607564000 -0.067883000

1 -1.429853000 -2.805214000 -0.013180000

1 -1.054785000 -4.341468000 -0.797351000

1 -0.647225000 -4.103283000 0.906226000

6 3.870625000 -2.550295000 1.014235000

6 4.883871000 -0.664489000 -0.774508000

6 5.220955000 -2.555120000 0.684903000

1 3.483473000 -3.299422000 1.696892000

6 5.719181000 -1.637194000 -0.237634000

1 5.285269000 0.056613000 -1.477754000

1 5.881554000 -3.293351000 1.128035000

1 6.764817000 -1.669349000 -0.526163000

6 -1.040961000 1.657776000 0.463525000

7 -1.160547000 0.299227000 0.889803000

1 3.367416000 3.136569000 1.850324000

28 0.474338000 0.278405000 0.074541000

1 0.007830000 1.837887000 -0.047239000

6 -2.257492000 -0.484415000 0.441471000

6 -3.053788000 -1.091438000 1.409733000

6 -2.557869000 -0.729641000 -0.908978000

6 -4.109493000 -1.936373000 1.066727000

1 -2.837199000 -0.901044000 2.456577000

6 -3.608363000 -1.556446000 -1.265791000

1 -1.951392000 -0.279679000 -1.688086000

6 -4.387120000 -2.173118000 -0.279213000

1 -4.697658000 -2.395079000 1.852819000

1 -3.830734000 -1.752900000 -2.309894000

8 -5.383132000 -2.981967000 -0.732703000

6 -6.157712000 -3.685952000 0.225635000

1 -6.853998000 -4.300928000 -0.345316000

1 -5.529602000 -4.333494000 0.847916000

1 -6.724667000 -3.000962000 0.866186000

6 -2.003385000 2.229825000 -0.560935000

6 -3.319356000 2.515280000 -0.193188000

6 -1.589293000 2.449448000 -1.873635000

6 -4.214438000 3.004927000 -1.138135000

1 -3.646137000 2.340845000 0.827597000

6 -2.485380000 2.942334000 -2.817432000

1 -0.564757000 2.221970000 -2.161585000

6 -3.799917000 3.218823000 -2.451301000

1 -5.239002000 3.217332000 -0.849590000

1 -2.156166000 3.108058000 -3.838389000

1 -4.501315000 3.599602000 -3.187082000

6 -0.911553000 2.640421000 1.633612000

8 -0.234913000 3.648471000 1.589853000

8 -1.662587000 2.292577000 2.665749000

6 -1.654758000 3.197887000 3.782231000

1 -2.015052000 4.180942000 3.472418000

1 -2.329393000 2.760878000 4.516075000

1 -0.646619000 3.281837000 4.192553000

**15** ***E(S)***

Electronic energy -3717.903270

EE+ Zero-point Energy -3717.178721

EE + Thermal Free Energy Correction -3717.252302

15 1.452922000 -1.319030000 0.773722000

6 3.672028000 -0.413307000 -0.584040000

6 3.256237000 -1.134744000 0.542501000

15 2.384815000 0.549531000 -1.424535000

6 2.703681000 2.339917000 -0.983653000

6 2.489827000 2.483019000 0.530891000

1 1.460374000 2.242749000 0.820406000

1 3.168955000 1.840476000 1.100871000

6 4.125331000 2.775352000 -1.355253000

1 4.227389000 3.845685000 -1.144493000

1 4.881793000 2.250166000 -0.766867000

1 4.338444000 2.624097000 -2.418094000

6 1.690935000 3.213712000 -1.739178000

1 0.657982000 2.993038000 -1.455494000

1 1.878598000 4.262825000 -1.486570000

1 1.786848000 3.110920000 -2.824441000

6 2.629842000 0.328377000 -3.208227000

1 3.631669000 0.634049000 -3.519902000

1 2.486695000 -0.729692000 -3.443180000

1 1.885692000 0.913619000 -3.753278000

6 1.184314000 -1.117162000 2.557870000

1 1.497314000 -0.112174000 2.852687000

1 0.129579000 -1.259444000 2.799631000

1 1.774456000 -1.847440000 3.117056000

6 1.077835000 -3.099097000 0.337922000

6 1.977377000 -4.082411000 1.097105000

1 1.643557000 -5.099477000 0.863521000

1 3.024248000 -4.002114000 0.794833000

1 1.912509000 -3.955513000 2.181931000

6 1.310361000 -3.262328000 -1.170739000

1 1.099721000 -4.298297000 -1.456925000

1 0.657506000 -2.611605000 -1.761194000

1 2.348255000 -3.042840000 -1.442908000

6 -0.387059000 -3.378399000 0.701029000

1 -1.074345000 -2.681602000 0.220047000

1 -0.640378000 -4.392250000 0.372293000

1 -0.548415000 -3.329969000 1.782324000

6 4.211654000 -1.737496000 1.365385000

6 5.022570000 -0.380974000 -0.938814000

6 5.558988000 -1.670295000 1.027890000

1 3.912225000 -2.273200000 2.259882000

6 5.961793000 -1.017225000 -0.135451000

1 5.343306000 0.148715000 -1.829929000

1 6.296700000 -2.143272000 1.667965000

1 7.011709000 -0.990109000 -0.408176000

6 -1.323394000 0.820826000 1.360776000

7 -1.244028000 -0.077136000 0.234929000

1 2.682087000 3.520986000 0.823449000

28 0.423073000 0.018269000 -0.615356000

6 -2.392684000 -0.449744000 -0.438846000

6 -3.698595000 -0.050953000 -0.091543000

6 -2.285627000 -1.305891000 -1.559659000

6 -4.820330000 -0.492024000 -0.794458000

1 -3.868752000 0.650123000 0.718106000

6 -3.395802000 -1.738241000 -2.265737000

1 -1.305216000 -1.654440000 -1.870445000

6 -4.679656000 -1.342849000 -1.886633000

1 -5.796570000 -0.144047000 -0.475865000

1 -3.278265000 -2.400039000 -3.118968000

8 -5.714740000 -1.832311000 -2.642855000

6 -7.028035000 -1.452339000 -2.274444000

1 -7.696696000 -1.946498000 -2.980862000

1 -7.272899000 -1.781705000 -1.257229000

1 -7.169552000 -0.366981000 -2.345802000

6 -1.473847000 2.310361000 1.046726000

6 -1.041489000 3.243936000 1.993350000

6 -1.941799000 2.772892000 -0.183710000

6 -1.076527000 4.607153000 1.720118000

1 -0.658328000 2.895384000 2.949180000

6 -1.979888000 4.138899000 -0.459922000

1 -2.281012000 2.069471000 -0.936722000

6 -1.546170000 5.060043000 0.488595000

1 -0.727697000 5.315721000 2.465343000

1 -2.346133000 4.480243000 -1.423482000

1 -1.569069000 6.123153000 0.269526000

6 -2.290209000 0.361523000 2.461392000

8 -2.948051000 1.097353000 3.169509000

8 -2.272881000 -0.961936000 2.601971000

6 -3.134132000 -1.501258000 3.613420000

1 -4.174683000 -1.256011000 3.389135000

1 -2.984256000 -2.579094000 3.579313000

1 -2.863326000 -1.110095000 4.596335000

1 -0.357337000 0.757376000 1.878529000

1 -0.383789000 1.058231000 -1.625924000

1 -0.224585000 0.441323000 -2.084959000

**TS2** ***E(S)***

Electronic energy -3717.889633

EE+ Zero-point Energy -3717.166512

EE + Thermal Free Energy Correction -3717.238533

Imaginary frequency *i*1142.8

15 1.466931000 -1.385129000 0.765953000

6 3.680193000 -0.503807000 -0.628460000

6 3.267131000 -1.258704000 0.479291000

15 2.401469000 0.519258000 -1.424120000

6 2.731225000 2.275258000 -0.861463000

6 2.628929000 2.297047000 0.670756000

1 1.641109000 1.964488000 1.010290000

1 3.388887000 1.665951000 1.142131000

6 4.115063000 2.761035000 -1.304276000

1 4.240345000 3.800910000 -0.982665000

1 4.919289000 2.176763000 -0.849363000

1 4.232703000 2.733608000 -2.391918000

6 1.645663000 3.184487000 -1.457467000

1 0.649437000 2.909582000 -1.100767000

1 1.839485000 4.216819000 -1.146925000

1 1.643130000 3.158367000 -2.551935000

6 2.678459000 0.416277000 -3.213531000

1 3.695669000 0.706887000 -3.486641000

1 2.502091000 -0.616653000 -3.525213000

1 1.965380000 1.065818000 -3.726312000

6 1.256462000 -1.060986000 2.540962000

1 1.624913000 -0.054175000 2.755193000

1 0.198714000 -1.119329000 2.807779000

1 1.818231000 -1.779928000 3.142998000

6 0.999408000 -3.158076000 0.433642000

6 1.847222000 -4.163787000 1.218824000

1 1.441843000 -5.167368000 1.047300000

1 2.888830000 -4.168167000 0.888156000

1 1.820924000 -3.976294000 2.296817000

6 1.184494000 -3.391991000 -1.072519000

1 0.915845000 -4.425592000 -1.315721000

1 0.547810000 -2.728824000 -1.667596000

1 2.223957000 -3.231847000 -1.378651000

6 -0.475831000 -3.315408000 0.831315000

1 -1.117324000 -2.580290000 0.340545000

1 -0.817231000 -4.313757000 0.536405000

1 -0.610782000 -3.223331000 1.913479000

6 4.223451000 -1.904540000 1.267241000

6 5.027097000 -0.483065000 -0.997953000

6 5.567293000 -1.853250000 0.912856000

1 3.922695000 -2.454931000 2.152751000

6 5.965522000 -1.166026000 -0.232042000

1 5.347387000 0.072089000 -1.873814000

1 6.305020000 -2.361430000 1.525445000

1 7.011822000 -1.148108000 -0.519281000

6 -1.308626000 1.017126000 1.222377000

7 -1.309021000 0.124349000 0.076527000

1 2.773446000 3.323380000 1.025110000

28 0.474346000 -0.038473000 -0.653197000

6 -2.490943000 -0.382699000 -0.477991000

6 -3.782642000 -0.010718000 -0.083567000

6 -2.380190000 -1.309236000 -1.531171000

6 -4.914387000 -0.559630000 -0.687316000

1 -3.939045000 0.736828000 0.686772000

6 -3.500312000 -1.842070000 -2.147061000

1 -1.394497000 -1.627675000 -1.858033000

6 -4.780949000 -1.479197000 -1.724519000

1 -5.890844000 -0.241199000 -0.340915000

1 -3.394676000 -2.558392000 -2.956099000

8 -5.823308000 -2.070512000 -2.383255000

6 -7.136833000 -1.720989000 -1.981690000

1 -7.810468000 -2.297014000 -2.617451000

1 -7.319523000 -1.982809000 -0.932673000

1 -7.330591000 -0.651648000 -2.127531000

6 -1.432666000 2.499328000 0.895930000

6 -0.792953000 3.423648000 1.725302000

6 -2.078988000 2.955608000 -0.253304000

6 -0.795674000 4.779046000 1.413483000

1 -0.272041000 3.072694000 2.612681000

6 -2.083524000 4.313276000 -0.566861000

1 -2.573675000 2.255423000 -0.919288000

6 -1.439983000 5.227605000 0.262214000

1 -0.285179000 5.483441000 2.063116000

1 -2.586585000 4.653719000 -1.466742000

1 -1.436047000 6.283750000 0.011017000

6 -2.231371000 0.572582000 2.357451000

8 -2.863142000 1.324430000 3.070361000

8 -2.198384000 -0.746011000 2.518283000

6 -3.019305000 -1.278377000 3.567408000

1 -4.068540000 -1.041728000 3.377391000

1 -2.863683000 -2.355411000 3.540700000

1 -2.713908000 -0.872562000 4.533879000

1 -0.321525000 0.912470000 1.692892000

1 -0.774037000 0.804710000 -1.056306000

1 -0.139232000 0.833799000 -1.794958000

**16** ***E(S)***

Electronic energy -3717.919781

EE+ Zero-point Energy -3717.191752

EE + Thermal Free Energy Correction -3717.264712

15 1.263209000 -1.191454000 1.008944000

6 3.594391000 -0.797901000 -0.439099000

6 3.087263000 -1.202981000 0.804543000

15 2.406005000 0.053709000 -1.538074000

6 2.941514000 1.852654000 -1.502400000

6 2.935472000 2.305146000 -0.034806000

1 1.949479000 2.167215000 0.423344000

1 3.672516000 1.762916000 0.565570000

6 4.336855000 2.050447000 -2.104746000

1 4.582210000 3.118217000 -2.079495000

1 5.108971000 1.524583000 -1.537910000

1 4.384386000 1.724422000 -3.148428000

6 1.926810000 2.689249000 -2.295970000

1 0.942583000 2.689059000 -1.819180000

1 2.280832000 3.725112000 -2.341725000

1 1.811097000 2.327668000 -3.323038000

6 2.726127000 -0.593931000 -3.205039000

1 3.788970000 -0.584212000 -3.457701000

1 2.359519000 -1.623485000 -3.242983000

1 2.174404000 -0.000463000 -3.937516000

6 1.031559000 -0.638336000 2.732699000

1 1.426765000 0.377658000 2.824329000

1 -0.032110000 -0.627578000 2.981855000

1 1.549080000 -1.279763000 3.449975000

6 0.797671000 -3.006327000 0.964116000

6 1.621421000 -3.874674000 1.921168000

1 1.220816000 -4.894765000 1.897512000

1 2.672634000 -3.927130000 1.627438000

1 1.564714000 -3.523770000 2.956190000

6 1.020376000 -3.496950000 -0.473430000

1 0.760434000 -4.559263000 -0.540487000

1 0.403097000 -2.954171000 -1.195629000

1 2.067726000 -3.387375000 -0.775118000

6 -0.683884000 -3.110767000 1.353204000

1 -1.323306000 -2.469753000 0.742420000

1 -1.018318000 -4.145353000 1.216657000

1 -0.837744000 -2.847455000 2.404522000

6 3.968400000 -1.668685000 1.785136000

6 4.951709000 -0.967327000 -0.726272000

6 5.325599000 -1.791084000 1.508091000

1 3.599068000 -1.956132000 2.763909000

6 5.813039000 -1.467795000 0.243686000

1 5.344387000 -0.700631000 -1.702131000

1 6.001157000 -2.157065000 2.274754000

1 6.867334000 -1.591926000 0.017590000

6 -1.340384000 1.354686000 1.013128000

7 -1.345153000 0.663555000 -0.326950000

1 3.177271000 3.372694000 0.015321000

28 0.465365000 -0.066693000 -0.743020000

6 -2.500527000 -0.138630000 -0.718193000

6 -3.811958000 0.305795000 -0.577871000

6 -2.267044000 -1.364100000 -1.338071000

6 -4.880942000 -0.482171000 -0.998103000

1 -4.028944000 1.270272000 -0.130923000

6 -3.318931000 -2.151754000 -1.774312000

1 -1.246742000 -1.700396000 -1.484816000

6 -4.636397000 -1.720709000 -1.594792000

1 -5.889423000 -0.110747000 -0.863095000

1 -3.135733000 -3.108318000 -2.252422000

8 -5.602147000 -2.565677000 -2.035156000

6 -6.958775000 -2.174799000 -1.874777000

1 -7.554644000 -2.992110000 -2.281629000

1 -7.209992000 -2.034131000 -0.817662000

1 -7.177189000 -1.256023000 -2.430268000

6 -1.235979000 2.856233000 0.873803000

6 -0.257015000 3.525988000 1.609460000

6 -2.067547000 3.588506000 0.022343000

6 -0.106189000 4.905230000 1.497729000

1 0.395389000 2.962252000 2.271192000

6 -1.914929000 4.966883000 -0.092345000

1 -2.835450000 3.091769000 -0.564191000

6 -0.934645000 5.628123000 0.644148000

1 0.663643000 5.411527000 2.071495000

1 -2.564875000 5.524841000 -0.759010000

1 -0.815350000 6.702929000 0.549424000

6 -2.479881000 0.914028000 1.925823000

8 -3.330022000 1.641471000 2.390325000

8 -2.386978000 -0.385368000 2.179296000

6 -3.448686000 -0.970553000 2.947834000

1 -4.402855000 -0.820102000 2.437716000

1 -3.213757000 -2.031786000 3.007415000

1 -3.481959000 -0.528832000 3.945443000

1 -0.440738000 1.024948000 1.532549000

1 -1.321120000 1.427456000 -1.001149000

1 0.255623000 0.431525000 -2.115774000

**Catalytic cycle *Z(R)***

**13** ***Z(R)***

Electronic energy -3716.712163

EE+ Zero-point Energy -3716.007713

EE + Thermal Free Energy Correction -3716.080825

15 1.267879000 -0.081523000 1.404201000

6 3.155470000 -1.448486000 -0.083146000

6 2.870370000 -0.946879000 1.195758000

15 2.111320000 -0.801783000 -1.440690000

6 3.231582000 0.375593000 -2.369622000

6 3.735879000 1.412693000 -1.356885000

1 2.907684000 1.891187000 -0.824499000

1 4.406520000 0.967549000 -0.615390000

6 4.421118000 -0.339516000 -3.020864000

1 5.023437000 0.400954000 -3.559125000

1 5.073122000 -0.814566000 -2.284266000

1 4.100660000 -1.094144000 -3.745607000

6 2.411024000 1.071746000 -3.465053000

1 1.641740000 1.726372000 -3.048628000

1 3.081760000 1.690151000 -4.071768000

1 1.925678000 0.353441000 -4.133780000

6 1.785107000 -2.230546000 -2.518088000

1 2.697011000 -2.782284000 -2.759470000

1 1.092258000 -2.898875000 -1.999623000

1 1.316273000 -1.890143000 -3.444142000

6 1.706263000 1.474028000 2.244977000

1 2.385354000 2.034767000 1.597774000

1 0.807733000 2.073842000 2.408331000

1 2.197511000 1.293558000 3.205322000

6 0.289459000 -1.104546000 2.625455000

6 1.071665000 -1.532919000 3.870991000

1 0.372727000 -1.993013000 4.579328000

1 1.840386000 -2.273807000 3.638788000

1 1.541613000 -0.684181000 4.378492000

6 -0.183183000 -2.353586000 1.868225000

1 -0.742055000 -3.007418000 2.547087000

1 -0.838887000 -2.100117000 1.030130000

1 0.667124000 -2.922222000 1.474695000

6 -0.900180000 -0.239251000 3.068907000

1 -1.397702000 0.252151000 2.230938000

1 -1.638802000 -0.868990000 3.576677000

1 -0.582227000 0.536100000 3.772851000

6 3.776459000 -1.169244000 2.237452000

6 4.266584000 -2.274895000 -0.273689000

6 4.904704000 -1.954808000 2.028948000

1 3.602787000 -0.736750000 3.216535000

6 5.130714000 -2.536881000 0.783323000

1 4.467512000 -2.709009000 -1.247685000

1 5.601308000 -2.125564000 2.843599000

1 5.993362000 -3.177289000 0.629504000

6 -1.884571000 1.759952000 -0.057139000

7 -1.447082000 0.579138000 -0.324952000

1 4.292373000 2.195050000 -1.884976000

28 0.385313000 0.077805000 -0.616299000

1 0.127106000 0.118160000 -2.067593000

6 -2.364306000 -0.516464000 -0.459409000

6 -3.340566000 -0.760881000 0.504632000

6 -2.219343000 -1.411135000 -1.525330000

6 -4.186497000 -1.861944000 0.401376000

1 -3.440418000 -0.110917000 1.366769000

6 -3.063393000 -2.499456000 -1.639767000

1 -1.453312000 -1.239771000 -2.271698000

6 -4.051943000 -2.736798000 -0.676394000

1 -4.929746000 -2.024950000 1.172073000

1 -2.965431000 -3.188922000 -2.471761000

8 -4.814845000 -3.838840000 -0.873011000

6 -5.830228000 -4.130604000 0.077595000

1 -6.307412000 -5.047077000 -0.269836000

1 -5.405245000 -4.297225000 1.073377000

1 -6.575070000 -3.328565000 0.121868000

6 -0.954440000 2.883451000 0.167077000

6 -1.204386000 3.851139000 1.147453000

6 0.205773000 2.966574000 -0.609887000

6 -0.279296000 4.866702000 1.363744000

1 -2.103799000 3.797004000 1.750194000

6 1.111373000 3.999307000 -0.406804000

1 0.373321000 2.232502000 -1.393095000

6 0.876370000 4.943839000 0.589284000

1 -0.465118000 5.602912000 2.138868000

1 2.000515000 4.065528000 -1.025428000

1 1.589790000 5.744324000 0.757249000

6 -3.364968000 2.074312000 -0.012785000

8 -3.947116000 2.456968000 0.978963000

8 -3.909837000 1.911860000 -1.205318000

6 -5.330518000 2.132333000 -1.290278000

1 -5.851454000 1.455798000 -0.609923000

1 -5.562970000 3.170204000 -1.045648000

1 -5.592395000 1.914441000 -2.323526000

**TS1 *Z(R)***

Electronic energy -3716.684431

EE+ Zero-point Energy -3715.981778

EE + Thermal Free Energy Correction -3716.056581

Imaginary frequency *i*39.1

15 1.223196000 -1.461823000 1.074774000

6 3.148798000 -1.186099000 -0.897779000

6 2.865739000 -1.805123000 0.330429000

15 2.090252000 0.237522000 -1.345066000

6 3.197454000 1.722509000 -1.077964000

6 3.658292000 1.683017000 0.385394000

1 2.809463000 1.621277000 1.072900000

1 4.324265000 0.836810000 0.580647000

6 4.417664000 1.736363000 -2.005199000

1 4.981343000 2.658685000 -1.824564000

1 5.092688000 0.899475000 -1.811192000

1 4.136777000 1.723881000 -3.062855000

6 2.360610000 2.983484000 -1.324767000

1 1.531063000 3.052417000 -0.620753000

1 2.993648000 3.867832000 -1.191804000

1 1.952387000 3.013104000 -2.340695000

6 1.787812000 0.077611000 -3.131116000

1 2.713698000 -0.059289000 -3.694588000

1 1.142212000 -0.789666000 -3.293991000

1 1.272015000 0.969081000 -3.495366000

6 1.606393000 -1.043261000 2.807638000

1 2.189255000 -0.118224000 2.818449000

1 0.678933000 -0.878194000 3.362078000

1 2.180916000 -1.828868000 3.305416000

6 0.348487000 -3.115648000 1.094428000

6 1.111515000 -4.237131000 1.808241000

1 0.458885000 -5.115811000 1.871122000

1 2.008550000 -4.538739000 1.262602000

1 1.394811000 -3.966076000 2.830200000

6 0.124612000 -3.509506000 -0.371451000

1 -0.418869000 -4.459926000 -0.416545000

1 -0.464821000 -2.762024000 -0.908847000

1 1.075175000 -3.641372000 -0.899410000

6 -0.993581000 -2.904747000 1.810164000

1 -1.572541000 -2.087939000 1.376268000

1 -1.592672000 -3.818224000 1.725326000

1 -0.850812000 -2.699662000 2.875971000

6 3.798262000 -2.691358000 0.878528000

6 4.283055000 -1.563479000 -1.622057000

6 4.948537000 -3.027395000 0.172478000

1 3.632003000 -3.126304000 1.857862000

6 5.172336000 -2.492691000 -1.094297000

1 4.483972000 -1.123866000 -2.593677000

1 5.663231000 -3.720662000 0.604642000

1 6.052996000 -2.780546000 -1.659794000

6 -1.629213000 1.810307000 0.421809000

7 -1.458827000 0.536322000 0.383823000

1 4.206094000 2.603200000 0.617344000

28 0.353779000 0.194955000 -0.137438000

1 -0.061136000 1.363846000 -0.902435000

6 -2.557425000 -0.295681000 0.019808000

6 -3.691781000 -0.351846000 0.821635000

6 -2.478192000 -1.079165000 -1.133637000

6 -4.756032000 -1.185099000 0.480753000

1 -3.742278000 0.239148000 1.730793000

6 -3.533893000 -1.900305000 -1.481700000

1 -1.598694000 -1.022134000 -1.767762000

6 -4.679256000 -1.960878000 -0.675711000

1 -5.625160000 -1.217488000 1.126219000

1 -3.492691000 -2.504406000 -2.381901000

8 -5.653139000 -2.802742000 -1.102891000

6 -6.849220000 -2.884911000 -0.340481000

1 -7.493027000 -3.586291000 -0.871391000

1 -6.653031000 -3.265402000 0.668002000

1 -7.348277000 -1.911858000 -0.275414000

6 -0.649753000 2.745630000 0.987301000

6 -0.600333000 4.067698000 0.528971000

6 0.217937000 2.326827000 2.003673000

6 0.338821000 4.947825000 1.050797000

1 -1.272031000 4.402468000 -0.256113000

6 1.147003000 3.216137000 2.528462000

1 0.138373000 1.321489000 2.403607000

6 1.217555000 4.520962000 2.044817000

1 0.388186000 5.965493000 0.678327000

1 1.814780000 2.891152000 3.319504000

1 1.948863000 5.211480000 2.452679000

6 -2.957143000 2.426747000 0.011890000

8 -3.684120000 3.007913000 0.785674000

8 -3.171000000 2.276092000 -1.283592000

6 -4.419286000 2.794023000 -1.781740000

1 -5.250450000 2.265674000 -1.310600000

1 -4.489303000 3.864469000 -1.580891000

1 -4.403132000 2.604535000 -2.852896000

**14** ***Z(R)***

Electronic energy -3716.714761

EE+ Zero-point Energy -3716.009085

EE + Thermal Free Energy Correction -3716.081166

15 0.733600000 -1.736740000 0.869266000

6 3.158366000 -1.354391000 -0.399103000

6 2.536518000 -1.975642000 0.696231000

15 2.209541000 -0.041229000 -1.241250000

6 3.191731000 1.540489000 -1.067377000

6 3.148431000 1.931159000 0.416395000

1 2.123501000 2.067187000 0.771995000

1 3.628062000 1.170573000 1.042099000

6 4.651641000 1.421052000 -1.520124000

1 5.096421000 2.422497000 -1.519753000

1 5.241691000 0.802118000 -0.840528000

1 4.745524000 1.019543000 -2.534113000

6 2.499637000 2.601940000 -1.936495000

1 1.422382000 2.671323000 -1.759893000

1 2.932445000 3.582539000 -1.712292000

1 2.654214000 2.405318000 -3.001768000

6 2.173131000 -0.473717000 -3.005391000

1 3.180946000 -0.532306000 -3.424588000

1 1.680864000 -1.442222000 -3.119376000

1 1.595961000 0.276853000 -3.550994000

6 0.407082000 -1.655536000 2.653075000

1 1.016678000 -0.854243000 3.078214000

1 -0.647171000 -1.421004000 2.816385000

1 0.647013000 -2.597671000 3.153040000

6 -0.023291000 -3.319369000 0.210705000

6 0.695643000 -4.564312000 0.743432000

1 0.143680000 -5.450115000 0.409894000

1 1.716335000 -4.648064000 0.362512000

1 0.727325000 -4.587849000 1.837410000

6 0.083208000 -3.272426000 -1.318650000

1 -0.315557000 -4.201945000 -1.739477000

1 -0.490161000 -2.438916000 -1.738491000

1 1.124723000 -3.178178000 -1.643772000

6 -1.495341000 -3.372982000 0.637987000

1 -2.059340000 -2.518948000 0.264944000

1 -1.946560000 -4.279632000 0.220171000

1 -1.606483000 -3.419524000 1.725573000

6 3.271334000 -2.840280000 1.511639000

6 4.467398000 -1.706800000 -0.738985000

6 4.595174000 -3.132079000 1.203454000

1 2.807513000 -3.302501000 2.376898000

6 5.180687000 -2.593745000 0.059495000

1 4.937167000 -1.286316000 -1.620856000

1 5.163924000 -3.799459000 1.842832000

1 6.201361000 -2.853263000 -0.202523000

6 -0.849624000 1.813506000 0.011336000

7 -1.249907000 0.623187000 0.683716000

1 3.682883000 2.876204000 0.561531000

28 0.295909000 0.097166000 -0.177063000

1 0.080313000 1.602266000 -0.715366000

6 -1.822735000 2.390350000 -1.014094000

8 -1.604130000 2.454814000 -2.206833000

8 -2.924295000 2.822436000 -0.425714000

6 -3.950109000 3.327445000 -1.297227000

1 -4.774661000 3.604131000 -0.643227000

1 -3.584600000 4.199185000 -1.842882000

1 -4.261289000 2.547691000 -1.995339000

6 -0.309554000 2.905122000 0.929600000

6 0.118147000 4.116265000 0.381073000

6 -0.180803000 2.683575000 2.297872000

6 0.680961000 5.092852000 1.195265000

1 0.021457000 4.296823000 -0.686730000

6 0.385559000 3.662084000 3.111230000

1 -0.519230000 1.741270000 2.713718000

6 0.821673000 4.865080000 2.562732000

1 1.014526000 6.029561000 0.759921000

1 0.487725000 3.481499000 4.176871000

1 1.266114000 5.624454000 3.198604000

6 -2.440481000 -0.033824000 0.253839000

6 -3.372156000 -0.383783000 1.228025000

6 -2.701917000 -0.403543000 -1.076595000

6 -4.529006000 -1.092943000 0.910524000

1 -3.179797000 -0.105792000 2.259645000

6 -3.852107000 -1.100473000 -1.408067000

1 -1.988963000 -0.162598000 -1.860231000

6 -4.771801000 -1.454822000 -0.415156000

1 -5.224576000 -1.353129000 1.699529000

1 -4.047705000 -1.392135000 -2.434959000

8 -5.861589000 -2.149500000 -0.839146000

6 -6.789569000 -2.597183000 0.137177000

1 -7.548516000 -3.160366000 -0.406890000

1 -6.309533000 -3.253042000 0.872275000

1 -7.266694000 -1.755650000 0.652093000

**15** ***Z(R)***

Electronic energy -3717.903906

EE+ Zero-point Energy -3717.178346

EE + Thermal Free Energy Correction -3717.250584

15 1.458289000 0.622962000 1.207888000

6 3.755050000 -0.439956000 0.128684000

6 3.269464000 0.560561000 0.980508000

15 2.518038000 -1.258888000 -0.918900000

6 2.807802000 -0.636118000 -2.658566000

6 2.338916000 0.826885000 -2.704398000

1 1.254474000 0.906864000 -2.568536000

1 2.831013000 1.436187000 -1.938270000

6 4.284335000 -0.725403000 -3.061083000

1 4.373636000 -0.450571000 -4.117905000

1 4.907828000 -0.036804000 -2.485651000

1 4.682214000 -1.738791000 -2.947020000

6 1.969607000 -1.489171000 -3.621961000

1 0.903106000 -1.462478000 -3.384407000

1 2.089800000 -1.088934000 -4.634447000

1 2.301515000 -2.531674000 -3.637264000

6 2.884504000 -3.035914000 -0.874414000

1 3.880423000 -3.252331000 -1.269839000

1 2.827703000 -3.371287000 0.164416000

1 2.136632000 -3.576334000 -1.459076000

6 1.043857000 2.389223000 1.203814000

1 1.273265000 2.801512000 0.217963000

1 -0.017472000 2.528040000 1.413358000

1 1.628244000 2.923305000 1.956936000

6 1.159263000 -0.054235000 2.925647000

6 1.996351000 0.673616000 3.984556000

1 1.709924000 0.290153000 4.970145000

1 3.066806000 0.493921000 3.860259000

1 1.817655000 1.753221000 3.986202000

6 1.543844000 -1.540631000 2.902670000

1 1.388189000 -1.966907000 3.899494000

1 0.933179000 -2.110651000 2.194958000

1 2.597135000 -1.681210000 2.638004000

6 -0.328799000 0.112938000 3.262177000

1 -0.972695000 -0.390689000 2.540061000

1 -0.512754000 -0.323560000 4.249993000

1 -0.616360000 1.168145000 3.306148000

6 4.170404000 1.373189000 1.674247000

6 5.126632000 -0.695264000 0.057063000

6 5.537142000 1.140856000 1.567148000

1 3.812360000 2.175707000 2.310530000

6 6.013229000 0.090685000 0.783878000

1 5.502616000 -1.494724000 -0.572768000

1 6.233404000 1.771074000 2.110959000

1 7.079020000 -0.105391000 0.727068000

6 -1.399345000 1.267836000 -0.813789000

7 -1.191729000 0.091726000 0.005900000

1 2.584981000 1.253514000 -3.682841000

28 0.516585000 -0.640644000 -0.293474000

6 -1.908994000 0.868673000 -2.212814000

8 -1.411935000 -0.052676000 -2.838524000

8 -2.905277000 1.600866000 -2.685425000

6 -3.401095000 1.239555000 -3.984543000

1 -4.205346000 1.942648000 -4.193577000

1 -2.609784000 1.331059000 -4.731115000

1 -3.782614000 0.216625000 -3.967838000

6 -2.133208000 2.431817000 -0.152596000

6 -2.142644000 3.681986000 -0.779259000

6 -2.696767000 2.316293000 1.117916000

6 -2.728232000 4.782925000 -0.164553000

1 -1.680958000 3.795828000 -1.756421000

6 -3.284761000 3.418461000 1.736549000

1 -2.666441000 1.363250000 1.633418000

6 -3.306829000 4.653882000 1.097140000

1 -2.725440000 5.745188000 -0.667808000

1 -3.721683000 3.307743000 2.724548000

1 -3.762466000 5.512882000 1.580190000

6 -2.255202000 -0.781712000 0.196667000

6 -2.022781000 -2.022189000 0.820281000

6 -3.591770000 -0.516672000 -0.178746000

6 -3.040191000 -2.941317000 1.066249000

1 -1.015530000 -2.276119000 1.139187000

6 -4.608123000 -1.430085000 0.062078000

1 -3.860717000 0.419404000 -0.654491000

6 -4.348107000 -2.649640000 0.686840000

1 -2.790574000 -3.876089000 1.555680000

1 -5.626813000 -1.199306000 -0.235866000

8 -5.424998000 -3.476850000 0.878684000

6 -5.187194000 -4.720063000 1.513592000

1 -6.154818000 -5.220321000 1.573724000

1 -4.495868000 -5.343612000 0.933666000

1 -4.788951000 -4.585848000 2.526732000

1 -0.403358000 1.667375000 -1.056749000

1 -0.286166000 -1.380653000 -1.533838000

1 -0.045981000 -1.981309000 -1.080533000

**TS2** ***Z(R)***

Electronic energy -3717.888711

EE+ Zero-point Energy -3717.166069

EE + Thermal Free Energy Correction -3717.239027

Imaginary frequency *i*1147.5

15 -1.498345000 0.112036000 -1.320081000

6 -3.758324000 -0.606215000 0.091286000

6 -3.308861000 0.007082000 -1.086720000

15 -2.502616000 -0.883333000 1.380110000

6 -2.842509000 0.393908000 2.704431000

6 -2.732557000 1.777245000 2.046981000

1 -1.737301000 1.938026000 1.615725000

1 -3.478242000 1.918611000 1.258444000

6 -4.232954000 0.213662000 3.323862000

1 -4.361932000 0.959533000 4.115924000

1 -5.031111000 0.369343000 2.593733000

1 -4.357196000 -0.775253000 3.775781000

6 -1.772088000 0.259054000 3.798373000

1 -0.774368000 0.504670000 3.423799000

1 -2.007538000 0.958466000 4.607707000

1 -1.743814000 -0.748070000 4.226864000

6 -2.813977000 -2.536438000 2.060504000

1 -3.853108000 -2.656930000 2.375899000

1 -2.587043000 -3.269804000 1.282067000

1 -2.152737000 -2.711157000 2.912131000

6 -1.211739000 1.808474000 -1.901930000

1 -1.411785000 2.495030000 -1.075305000

1 -0.175078000 1.928949000 -2.219283000

1 -1.872000000 2.054822000 -2.737121000

6 -1.100642000 -1.076759000 -2.700550000

6 -1.895114000 -0.793918000 -3.979485000

1 -1.533199000 -1.461785000 -4.769333000

1 -2.963078000 -0.985824000 -3.850046000

1 -1.761863000 0.235100000 -4.328333000

6 -1.427474000 -2.483445000 -2.179179000

1 -1.204246000 -3.219492000 -2.959077000

1 -0.828930000 -2.735480000 -1.296516000

1 -2.485635000 -2.582444000 -1.915162000

6 0.400316000 -0.956725000 -2.998163000

1 1.009699000 -1.145457000 -2.111032000

1 0.669997000 -1.699809000 -3.756724000

1 0.656993000 0.030943000 -3.393197000

6 -4.239921000 0.434926000 -2.037080000

6 -5.117552000 -0.879553000 0.263573000

6 -5.595607000 0.193678000 -1.842725000

1 -3.912993000 0.946631000 -2.936224000

6 -6.031649000 -0.486236000 -0.707373000

1 -5.466668000 -1.389030000 1.155974000

1 -6.312689000 0.524906000 -2.586866000

1 -7.087444000 -0.696800000 -0.570866000

6 1.477584000 1.493198000 0.575802000

7 1.280108000 0.077122000 0.334972000

1 -2.894764000 2.548703000 2.807417000

28 -0.553243000 -0.506371000 0.555589000

6 2.262207000 1.699402000 1.883018000

8 2.054944000 1.014032000 2.866885000

8 3.137493000 2.691257000 1.851762000

6 3.867418000 2.933490000 3.065969000

1 4.524169000 3.773317000 2.846695000

1 3.180547000 3.186270000 3.875987000

1 4.451580000 2.050599000 3.333174000

6 1.932211000 2.326606000 -0.614022000

6 1.734970000 3.710412000 -0.572197000

6 2.444224000 1.756122000 -1.778448000

6 2.055026000 4.508231000 -1.664286000

1 1.314969000 4.163662000 0.321977000

6 2.764944000 2.554564000 -2.875470000

1 2.580733000 0.683898000 -1.846212000

6 2.573053000 3.931248000 -2.822893000

1 1.890178000 5.580284000 -1.615040000

1 3.160659000 2.092326000 -3.774821000

1 2.818658000 4.551230000 -3.679624000

6 2.371033000 -0.795825000 0.226396000

6 2.112388000 -2.163973000 0.066779000

6 3.718629000 -0.397195000 0.295966000

6 3.137062000 -3.104005000 -0.015867000

1 1.084005000 -2.505853000 -0.011724000

6 4.741932000 -1.328829000 0.205825000

1 3.989106000 0.648308000 0.398525000

6 4.464670000 -2.688885000 0.053964000

1 2.877849000 -4.148821000 -0.142034000

1 5.777467000 -1.006313000 0.254317000

8 5.548247000 -3.519854000 -0.023424000

6 5.301971000 -4.908783000 -0.161353000

1 6.281453000 -5.387585000 -0.193885000

1 4.736340000 -5.303265000 0.691266000

1 4.761725000 -5.131406000 -1.089232000

1 0.487757000 1.889531000 0.846173000

1 0.681339000 -0.467031000 1.517424000

1 0.006480000 -1.048152000 1.907230000

**16** ***Z(R)***

Electronic energy -3717.918143

EE+ Zero-point Energy -3717.189839

EE + Thermal Free Energy Correction -3717.263919

15 -1.351174000 0.313816000 1.252035000

6 -3.699684000 0.535841000 -0.218807000

6 -3.181350000 0.419859000 1.078009000

15 -2.515415000 0.220733000 -1.574074000

6 -3.026576000 -1.434018000 -2.288387000

6 -2.863356000 -2.479125000 -1.174056000

1 -1.824235000 -2.544380000 -0.830115000

1 -3.495133000 -2.255050000 -0.308491000

6 -4.476523000 -1.426945000 -2.786124000

1 -4.692449000 -2.394808000 -3.252434000

1 -5.190140000 -1.290438000 -1.970112000

1 -4.650390000 -0.650972000 -3.538318000

6 -2.093960000 -1.777981000 -3.458580000

1 -1.055866000 -1.880856000 -3.130958000

1 -2.409175000 -2.732594000 -3.894163000

1 -2.131340000 -1.021113000 -4.248790000

6 -2.873330000 1.512409000 -2.802170000

1 -3.940194000 1.588373000 -3.024717000

1 -2.526707000 2.466023000 -2.393707000

1 -2.325145000 1.309170000 -3.724473000

6 -1.157694000 -0.929440000 2.569695000

1 -1.403433000 -1.908390000 2.151015000

1 -0.127170000 -0.946017000 2.923854000

1 -1.814138000 -0.732772000 3.420321000

6 -0.888999000 1.978269000 1.982537000

6 -1.625019000 2.306551000 3.285329000

1 -1.218793000 3.240141000 3.691726000

1 -2.695464000 2.457487000 3.127074000

1 -1.488737000 1.529695000 4.044411000

6 -1.216678000 3.040346000 0.923419000

1 -0.941832000 4.030454000 1.304243000

1 -0.659467000 2.870410000 -0.003741000

1 -2.284747000 3.057032000 0.682536000

6 0.620490000 1.960821000 2.256571000

1 1.197097000 1.717903000 1.360470000

1 0.935009000 2.954207000 2.596564000

1 0.878790000 1.242697000 3.040579000

6 -4.057566000 0.463058000 2.167594000

6 -5.058701000 0.803783000 -0.408192000

6 -5.415236000 0.689499000 1.971129000

1 -3.687281000 0.336997000 3.179103000

6 -5.912829000 0.887091000 0.685066000

1 -5.458102000 0.936878000 -1.408387000

1 -6.083675000 0.726822000 2.825524000

1 -6.968004000 1.090990000 0.533068000

6 1.667857000 -1.781774000 -0.348342000

7 1.375579000 -0.440869000 -0.903501000

1 -3.152668000 -3.464257000 -1.556457000

28 -0.569221000 0.085369000 -0.818715000

6 2.803273000 -2.413936000 -1.168477000

8 2.943283000 -2.172250000 -2.351595000

8 3.548187000 -3.264249000 -0.487089000

6 4.581558000 -3.936830000 -1.228605000

1 5.076762000 -4.586316000 -0.509350000

1 4.142275000 -4.524140000 -2.037078000

1 5.284798000 -3.207023000 -1.634193000

6 1.784795000 -1.957607000 1.152049000

6 1.325860000 -3.174433000 1.669540000

6 2.347082000 -1.030624000 2.030466000

6 1.432171000 -3.464630000 3.024125000

1 0.879643000 -3.904329000 0.999450000

6 2.449172000 -1.317769000 3.390697000

1 2.698899000 -0.071387000 1.677484000

6 1.997123000 -2.533561000 3.892817000

1 1.067301000 -4.414811000 3.401241000

1 2.883972000 -0.579472000 4.057344000

1 2.079425000 -2.752854000 4.952766000

6 2.378263000 0.614161000 -0.808683000

6 1.968236000 1.896045000 -1.159192000

6 3.715757000 0.403745000 -0.470231000

6 2.855975000 2.966921000 -1.157461000

1 0.934851000 2.065224000 -1.446455000

6 4.606011000 1.466634000 -0.448672000

1 4.087944000 -0.575033000 -0.188013000

6 4.185131000 2.754539000 -0.790873000

1 2.494742000 3.950200000 -1.433177000

1 5.642272000 1.308301000 -0.169075000

8 5.134142000 3.724199000 -0.735839000

6 4.752524000 5.049767000 -1.074454000

1 5.652017000 5.656396000 -0.966917000

1 4.396502000 5.110269000 -2.108878000

1 3.978196000 5.426705000 -0.396918000

1 0.801121000 -2.381686000 -0.655528000

1 1.335856000 -0.636833000 -1.905605000

1 -0.462774000 0.215047000 -2.283821000

**Catalytic cycle *Z(S)***

**13** ***Z(S)***

Electronic energy -3716.712857

EE+ Zero-point Energy -3716.009517

EE + Thermal Free Energy Correction -3716.083323

15 -1.090729000 -0.035029000 1.432864000

6 -3.427413000 -0.554903000 0.044909000

6 -2.861382000 -0.499781000 1.327590000

15 -2.244809000 -0.552265000 -1.353384000

6 -2.321679000 -2.279837000 -2.067219000

6 -1.684481000 -3.218062000 -1.031106000

1 -0.628595000 -2.978519000 -0.861939000

1 -2.206304000 -3.171628000 -0.069179000

6 -3.756929000 -2.735789000 -2.358246000

1 -3.719457000 -3.709088000 -2.859661000

1 -4.339845000 -2.860172000 -1.442903000

1 -4.287099000 -2.043391000 -3.019634000

6 -1.515095000 -2.312440000 -3.373055000

1 -0.460791000 -2.074122000 -3.211451000

1 -1.571010000 -3.321128000 -3.796890000

1 -1.915563000 -1.618410000 -4.119035000

6 -2.958639000 0.606893000 -2.559292000

1 -3.968880000 0.312670000 -2.856831000

1 -3.002191000 1.595015000 -2.092947000

1 -2.324040000 0.663142000 -3.446089000

6 -0.368950000 -1.327840000 2.495116000

1 -0.427430000 -2.278529000 1.957544000

1 0.682780000 -1.111477000 2.695119000

1 -0.902880000 -1.421162000 3.444757000

6 -1.039022000 1.573652000 2.384708000

6 -1.802813000 1.552285000 3.713336000

1 -1.602256000 2.489141000 4.246172000

1 -2.883018000 1.486633000 3.564004000

1 -1.487290000 0.729301000 4.362809000

6 -1.644314000 2.643480000 1.465406000

1 -1.610397000 3.617976000 1.964609000

1 -1.100273000 2.727722000 0.521027000

1 -2.692048000 2.420920000 1.234026000

6 0.438550000 1.876274000 2.677259000

1 1.059386000 1.855862000 1.780923000

1 0.521348000 2.878995000 3.110944000

1 0.855475000 1.167854000 3.400067000

6 -3.668668000 -0.737030000 2.444937000

6 -4.810181000 -0.704332000 -0.097599000

6 -5.036472000 -0.932871000 2.291727000

1 -3.233855000 -0.765866000 3.437945000

6 -5.612684000 -0.879483000 1.024062000

1 -5.263087000 -0.697931000 -1.083582000

1 -5.655805000 -1.113046000 3.164637000

1 -6.684890000 -0.999498000 0.906617000

6 2.045568000 1.580007000 -0.519316000

7 1.492948000 0.424946000 -0.413071000

1 -1.740275000 -4.250529000 -1.393893000

28 -0.341982000 -0.012161000 -0.643188000

1 -0.105761000 -0.235748000 -2.081404000

6 2.264733000 -0.718091000 -0.023425000

6 2.250202000 -1.854267000 -0.826314000

6 2.958429000 -0.730922000 1.189478000

6 2.953850000 -2.992770000 -0.448231000

1 1.706559000 -1.844266000 -1.765066000

6 3.653109000 -1.865861000 1.574694000

1 2.937932000 0.135978000 1.842282000

6 3.659897000 -3.001409000 0.757702000

1 2.942043000 -3.857884000 -1.100009000

1 4.191106000 -1.890983000 2.516356000

8 4.371708000 -4.057634000 1.221696000

6 4.395779000 -5.246981000 0.444522000

1 4.848452000 -5.073321000 -0.537911000

1 5.010031000 -5.953110000 1.003608000

1 3.389884000 -5.662883000 0.319683000

6 3.529469000 1.751927000 -0.276358000

8 3.983804000 2.348613000 0.676061000

8 4.235571000 1.182457000 -1.235130000

6 5.666798000 1.214856000 -1.071963000

1 6.016998000 2.247149000 -1.023591000

1 5.943956000 0.677176000 -0.162814000

1 6.068069000 0.714293000 -1.950452000

6 1.260868000 2.788710000 -0.854837000

6 0.225202000 2.707744000 -1.793353000

6 1.519355000 4.006508000 -0.214887000

6 -0.548688000 3.824595000 -2.079340000

1 0.025240000 1.766687000 -2.292571000

6 0.723507000 5.114094000 -0.487673000

1 2.317890000 4.080238000 0.514458000

6 -0.308673000 5.027499000 -1.418626000

1 -1.341878000 3.751841000 -2.816726000

1 0.913129000 6.049197000 0.029112000

1 -0.921480000 5.897585000 -1.632211000

**TS1 *Z(S)***

Electronic energy -3716.683949

EE+ Zero-point Energy -3715.981239

EE + Thermal Free Energy Correction -3716.054694

Imaginary frequency *i*59.0

15 1.037413000 -1.674226000 -0.620723000

6 3.397018000 -0.630599000 0.396895000

6 2.814624000 -1.776839000 -0.169313000

15 2.250726000 0.691607000 0.937139000

6 2.256206000 0.622659000 2.804349000

6 1.532490000 -0.673073000 3.198417000

1 0.487094000 -0.665250000 2.869216000

1 2.021179000 -1.554496000 2.768945000

6 3.667851000 0.629444000 3.400671000

1 3.587512000 0.680388000 4.492402000

1 4.222051000 -0.279378000 3.152635000

1 4.250983000 1.495694000 3.072320000

6 1.482129000 1.839929000 3.328607000

1 0.471874000 1.893551000 2.912572000

1 1.395303000 1.763733000 4.418184000

1 1.999503000 2.777874000 3.101817000

6 3.049877000 2.241634000 0.420134000

1 4.031011000 2.374992000 0.883803000

1 3.170668000 2.215088000 -0.666088000

1 2.410967000 3.088767000 0.679073000

6 0.311284000 -3.176518000 0.112282000

1 0.291954000 -3.059954000 1.199037000

1 -0.714559000 -3.306127000 -0.240865000

1 0.889037000 -4.070737000 -0.135874000

6 0.968539000 -1.882771000 -2.477089000

6 1.373024000 -3.274723000 -2.971208000

1 1.229734000 -3.320913000 -4.057025000

1 2.425347000 -3.491721000 -2.773880000

1 0.761559000 -4.064947000 -2.525144000

6 1.902658000 -0.825520000 -3.081613000

1 1.766852000 -0.794654000 -4.168363000

1 1.702706000 0.176981000 -2.688944000

1 2.953278000 -1.057857000 -2.880808000

6 -0.487922000 -1.609478000 -2.887824000

1 -0.797370000 -0.590258000 -2.641694000

1 -0.590150000 -1.741143000 -3.970698000

1 -1.184178000 -2.302239000 -2.402535000

6 3.614958000 -2.895228000 -0.423713000

6 4.780449000 -0.577886000 0.590866000

6 4.986013000 -2.845861000 -0.196888000

1 3.173436000 -3.810651000 -0.802368000

6 5.573595000 -1.677205000 0.282529000

1 5.241215000 0.317166000 0.996440000

1 5.597974000 -3.717425000 -0.406959000

1 6.646987000 -1.628646000 0.435874000

6 -1.707306000 1.799290000 -0.409268000

7 -1.462557000 0.534357000 -0.359569000

1 1.541856000 -0.781973000 4.288610000

28 0.354523000 0.302557000 0.104192000

1 -0.024812000 1.578203000 0.675934000

6 -2.496414000 -0.410518000 -0.126163000

6 -2.364313000 -1.320707000 0.916628000

6 -3.613947000 -0.465595000 -0.963303000

6 -3.356665000 -2.263030000 1.160287000

1 -1.487536000 -1.280355000 1.556293000

6 -4.599352000 -1.411722000 -0.734882000

1 -3.700813000 0.217661000 -1.802603000

6 -4.480754000 -2.310681000 0.330790000

1 -3.238390000 -2.953032000 1.986842000

1 -5.469000000 -1.471873000 -1.380595000

8 -5.497692000 -3.197037000 0.471503000

6 -5.478847000 -4.067059000 1.594757000

1 -5.459684000 -3.503297000 2.533966000

1 -6.403013000 -4.642920000 1.540301000

1 -4.624734000 -4.752197000 1.558293000

6 -3.083346000 2.343379000 -0.055130000

8 -3.738023000 3.026370000 -0.810980000

8 -3.429687000 2.011650000 1.176748000

6 -4.740970000 2.429620000 1.598022000

1 -4.817887000 3.517905000 1.568235000

1 -5.495715000 1.980816000 0.948989000

1 -4.848257000 2.064827000 2.617291000

6 -0.772307000 2.786760000 -0.976316000

6 0.020180000 2.441788000 -2.076594000

6 -0.680273000 4.068274000 -0.423411000

6 0.933419000 3.356751000 -2.586022000

1 -0.094825000 1.468470000 -2.540615000

6 0.246370000 4.972195000 -0.926791000

1 -1.304620000 4.343367000 0.422089000

6 1.058457000 4.615828000 -2.003247000

1 1.543457000 3.087187000 -3.442230000

1 0.335162000 5.956873000 -0.479849000

1 1.778852000 5.326584000 -2.395563000

**14** ***Z(S)***

Electronic energy -3716.720466

EE+ Zero-point Energy -3716.013915

EE + Thermal Free Energy Correction -3716.086534

15 -1.215333000 -1.365683000 0.953680000

6 -3.452541000 -0.443671000 -0.378033000

6 -2.913701000 -1.542830000 0.307650000

15 -2.298360000 0.897765000 -0.825624000

6 -2.204271000 0.957344000 -2.689309000

6 -1.372008000 -0.257884000 -3.125101000

1 -0.333519000 -0.163952000 -2.791944000

1 -1.781115000 -1.193669000 -2.727395000

6 -3.577330000 0.921425000 -3.367114000

1 -3.440044000 1.085630000 -4.441660000

1 -4.069908000 -0.045915000 -3.240486000

1 -4.244115000 1.705665000 -2.994698000

6 -1.483890000 2.257499000 -3.074855000

1 -0.507672000 2.356321000 -2.590137000

1 -1.311662000 2.258028000 -4.156663000

1 -2.083827000 3.140764000 -2.834450000

6 -3.098959000 2.423017000 -0.246612000

1 -4.050828000 2.594416000 -0.756398000

1 -3.277949000 2.330707000 0.827782000

1 -2.437870000 3.275708000 -0.417687000

6 -0.413088000 -2.963643000 0.639010000

1 -0.397988000 -3.140300000 -0.439047000

1 0.613952000 -2.948551000 1.008499000

1 -0.956801000 -3.775977000 1.128016000

6 -1.381002000 -1.144738000 2.799846000

6 -2.240401000 -2.235024000 3.448216000

1 -2.229245000 -2.085746000 4.533709000

1 -3.281768000 -2.188956000 3.119409000

1 -1.852784000 -3.239005000 3.248974000

6 -2.019046000 0.232546000 3.032467000

1 -2.158706000 0.391187000 4.107233000

1 -1.383219000 1.039849000 2.651699000

1 -3.000226000 0.309979000 2.551770000

6 0.033416000 -1.180655000 3.397748000

1 0.685390000 -0.433409000 2.937592000

1 -0.033514000 -0.968564000 4.470517000

1 0.497459000 -2.165653000 3.283550000

6 -3.710544000 -2.664431000 0.554998000

6 -4.810663000 -0.433662000 -0.709094000

6 -5.048508000 -2.664585000 0.177730000

1 -3.295856000 -3.533958000 1.054359000

6 -5.604603000 -1.539545000 -0.427471000

1 -5.251719000 0.432462000 -1.190796000

1 -5.662860000 -3.537198000 0.374890000

1 -6.657564000 -1.527363000 -0.689893000

6 1.379171000 1.485572000 -0.101053000

7 1.361186000 0.276573000 0.666094000

1 -1.368570000 -0.327875000 -4.218284000

28 -0.360841000 0.387224000 0.047618000

6 1.479413000 2.751762000 0.729976000

6 0.769862000 3.893193000 0.356747000

6 2.311101000 2.785989000 1.848124000

6 0.885537000 5.063458000 1.101755000

1 0.122282000 3.865707000 -0.517063000

6 2.426894000 3.956581000 2.591295000

1 2.858347000 1.892920000 2.132413000

6 1.714588000 5.095840000 2.220423000

1 0.326276000 5.947156000 0.810667000

1 3.074367000 3.979164000 3.462554000

1 1.804445000 6.007314000 2.803223000

1 0.358747000 1.630863000 -0.696956000

6 2.197919000 -0.812544000 0.278804000

6 2.117327000 -1.467652000 -0.952862000

6 3.121774000 -1.286681000 1.216956000

6 2.934009000 -2.555228000 -1.254770000

1 1.392215000 -1.142607000 -1.692571000

6 3.938032000 -2.370081000 0.933260000

1 3.191917000 -0.793275000 2.181381000

6 3.851575000 -3.010314000 -0.307646000

1 2.832965000 -3.036739000 -2.220137000

1 4.654145000 -2.733827000 1.663314000

8 4.696336000 -4.060563000 -0.494325000

6 2.353755000 1.485725000 -1.275991000

8 2.033259000 1.380215000 -2.441740000

8 3.600038000 1.576676000 -0.843946000

6 4.628163000 1.422313000 -1.835826000

1 4.558318000 2.213628000 -2.584324000

1 5.568224000 1.494545000 -1.292399000

1 4.535813000 0.443898000 -2.312461000

6 4.639887000 -4.747210000 -1.735256000

1 4.871830000 -4.079221000 -2.572380000

1 5.397690000 -5.529378000 -1.680779000

1 3.657310000 -5.206217000 -1.893732000

**15** ***Z(S)***

Electronic energy -3717.900557

EE+ Zero-point Energy -3717.175577

EE + Thermal Free Energy Correction -3717.248786

15 0.670034000 -1.251964000 0.994832000

6 3.158729000 -1.437513000 -0.136122000

6 2.467034000 -1.553830000 1.076416000

15 2.329650000 -0.445020000 -1.413612000

6 3.371007000 1.100269000 -1.578074000

6 3.510756000 1.695265000 -0.170344000

1 2.539290000 1.849085000 0.309750000

1 4.115930000 1.053847000 0.477446000

6 4.757169000 0.816272000 -2.167712000

1 5.280147000 1.771500000 -2.289832000

1 5.368557000 0.193898000 -1.511087000

1 4.699236000 0.342644000 -3.152357000

6 2.629792000 2.074148000 -2.505772000

1 1.664578000 2.373156000 -2.095408000

1 3.236434000 2.978037000 -2.625950000

1 2.467698000 1.645501000 -3.500204000

6 2.433644000 -1.351509000 -2.983900000

1 3.437892000 -1.752755000 -3.140777000

1 1.716471000 -2.175082000 -2.969671000

1 2.180517000 -0.681725000 -3.809599000

6 0.188183000 -0.728473000 2.661456000

1 0.759636000 0.161297000 2.930606000

1 -0.878693000 -0.501794000 2.686575000

1 0.389813000 -1.522138000 3.385830000

6 -0.047812000 -2.979498000 0.755928000

6 0.713126000 -4.011913000 1.598566000

1 0.184461000 -4.968447000 1.522073000

1 1.734474000 -4.165582000 1.242099000

1 0.750864000 -3.739170000 2.657947000

6 0.066364000 -3.357117000 -0.725172000

1 -0.315388000 -4.374580000 -0.864020000

1 -0.519423000 -2.688520000 -1.363116000

1 1.108377000 -3.347158000 -1.061107000

6 -1.517347000 -2.977742000 1.196100000

1 -2.113101000 -2.240298000 0.660003000

1 -1.940595000 -3.966418000 0.987602000

1 -1.618112000 -2.795750000 2.270229000

6 3.124768000 -2.047092000 2.205140000

6 4.459821000 -1.934004000 -0.247195000

6 4.443039000 -2.476875000 2.104346000

1 2.608497000 -2.122315000 3.156219000

6 5.096204000 -2.456519000 0.873029000

1 4.980443000 -1.905535000 -1.198299000

1 4.953817000 -2.856351000 2.983400000

1 6.110301000 -2.833508000 0.789307000

6 -1.451239000 1.714114000 0.925591000

7 -1.377035000 0.727399000 -0.120497000

1 4.007125000 2.668760000 -0.239507000

28 0.301283000 0.070184000 -0.695865000

6 -0.241143000 2.641687000 0.949346000

6 0.450916000 2.860978000 2.141347000

6 0.109132000 3.385096000 -0.183212000

6 1.484650000 3.793371000 2.200362000

1 0.164556000 2.314263000 3.034946000

6 1.132969000 4.324932000 -0.121809000

1 -0.439792000 3.242920000 -1.109480000

6 1.823318000 4.532196000 1.070383000

1 2.015192000 3.950199000 3.134485000

1 1.390624000 4.897511000 -1.007452000

1 2.622084000 5.265911000 1.117452000

6 -2.507932000 -0.049038000 -0.329382000

6 -3.604167000 -0.110641000 0.548158000

6 -2.592274000 -0.839482000 -1.499212000

6 -4.710748000 -0.925736000 0.291370000

1 -3.607375000 0.455416000 1.473447000

6 -3.677746000 -1.657713000 -1.749256000

1 -1.786605000 -0.810887000 -2.225804000

6 -4.749952000 -1.714557000 -0.852310000

1 -5.524012000 -0.936624000 1.008470000

1 -3.714002000 -2.263173000 -2.650276000

8 -5.774574000 -2.559033000 -1.190878000

6 -2.658495000 2.656998000 0.798075000

8 -3.211900000 3.151409000 1.761872000

8 -2.996485000 2.925561000 -0.458337000

6 -4.096463000 3.828914000 -0.635551000

1 -3.866751000 4.800712000 -0.193453000

1 -4.227071000 3.923033000 -1.712302000

1 -4.998793000 3.416515000 -0.178651000

6 -6.858729000 -2.664924000 -0.285705000

1 -7.373972000 -1.705016000 -0.159414000

1 -7.549997000 -3.386835000 -0.722855000

1 -6.529411000 -3.029579000 0.694895000

1 -1.561985000 1.295257000 1.937344000

1 0.134128000 0.471309000 -2.347989000

1 -0.223765000 1.052390000 -1.972684000

**TS2** ***Z(S)***

Electronic energy -3717.892909

EE+ Zero-point Energy -3717.169463

EE + Thermal Free Energy Correction -3717.242106

Imaginary frequency *i*1039.9

15 -0.959809000 -1.152904000 -1.153496000

6 -3.238492000 -1.340363000 0.373369000

6 -2.734156000 -1.513805000 -0.922226000

15 -2.234674000 -0.306704000 1.485859000

6 -3.211319000 1.270180000 1.698230000

6 -3.384834000 1.859993000 0.291238000

1 -2.424107000 1.989439000 -0.217299000

1 -4.021196000 1.225896000 -0.333937000

6 -4.582311000 1.037859000 2.343102000

1 -5.060454000 2.011577000 2.497588000

1 -5.243802000 0.447634000 1.705254000

1 -4.503065000 0.548935000 3.319113000

6 -2.397935000 2.221929000 2.587004000

1 -1.427801000 2.463687000 2.148575000

1 -2.955061000 3.157752000 2.703775000

1 -2.235260000 1.803291000 3.585616000

6 -2.155685000 -1.179302000 3.075640000

1 -3.152432000 -1.456789000 3.427642000

1 -1.557205000 -2.084051000 2.940497000

1 -1.670645000 -0.545012000 3.821311000

6 -0.828873000 -0.456258000 -2.824347000

1 -1.483671000 0.415473000 -2.885021000

1 0.200326000 -0.146612000 -3.015826000

1 -1.128078000 -1.185575000 -3.581970000

6 -0.154036000 -2.843639000 -1.184822000

6 -0.915851000 -3.844367000 -2.061117000

1 -0.325616000 -4.765787000 -2.120227000

1 -1.891582000 -4.101418000 -1.641827000

1 -1.059175000 -3.478874000 -3.082805000

6 -0.114398000 -3.358232000 0.260390000

1 0.308526000 -4.368740000 0.274019000

1 0.509035000 -2.723270000 0.896466000

1 -1.119442000 -3.408272000 0.694199000

6 1.264853000 -2.675366000 -1.742871000

1 1.834692000 -1.914196000 -1.208648000

1 1.801315000 -3.625293000 -1.642251000

1 1.247969000 -2.413641000 -2.805565000

6 -3.549706000 -2.068385000 -1.911997000

6 -4.503314000 -1.835369000 0.701854000

6 -4.830494000 -2.504840000 -1.592845000

1 -3.182561000 -2.180803000 -2.926770000

6 -5.292998000 -2.421672000 -0.280565000

1 -4.877229000 -1.756002000 1.717174000

1 -5.461009000 -2.934145000 -2.364797000

1 -6.277963000 -2.799432000 -0.025982000

6 1.639713000 1.660841000 -0.960995000

7 1.470375000 0.648979000 0.054511000

1 -3.859163000 2.844108000 0.367329000

28 -0.314257000 0.076149000 0.549144000

6 0.468686000 2.636458000 -1.019275000

6 -0.211612000 2.841018000 -2.219731000

6 0.132797000 3.417718000 0.092299000

6 -1.228307000 3.789880000 -2.304903000

1 0.064765000 2.264889000 -3.096930000

6 -0.876253000 4.370807000 0.005363000

1 0.674301000 3.293499000 1.025448000

6 -1.561084000 4.558363000 -1.193247000

1 -1.750813000 3.934722000 -3.245403000

1 -1.126692000 4.968250000 0.876396000

1 -2.348989000 5.302130000 -1.260266000

6 2.544726000 -0.201349000 0.331926000

6 3.696185000 -0.282204000 -0.461520000

6 2.480340000 -1.045732000 1.459241000

6 4.736453000 -1.163852000 -0.154651000

1 3.799335000 0.320137000 -1.357320000

6 3.504954000 -1.922731000 1.762661000

1 1.609858000 -1.019576000 2.107999000

6 4.645535000 -1.993891000 0.956481000

1 5.602299000 -1.188947000 -0.806349000

1 3.434848000 -2.569253000 2.632208000

8 5.597423000 -2.898180000 1.340220000

6 2.885254000 2.534785000 -0.759597000

8 3.508312000 3.019622000 -1.683833000

8 3.173289000 2.748807000 0.518791000

6 4.309790000 3.585129000 0.779759000

1 4.155037000 4.579905000 0.356925000

1 4.388362000 3.640922000 1.864055000

1 5.210686000 3.135872000 0.356194000

6 6.754461000 -3.014021000 0.530177000

1 7.316609000 -2.073183000 0.494780000

1 7.372968000 -3.783293000 0.994405000

1 6.500593000 -3.323049000 -0.490893000

1 1.762943000 1.222305000 -1.961519000

1 0.045049000 0.825988000 1.896367000

1 0.742591000 0.988439000 1.299649000

**16** ***Z(S)***

Electronic energy -3717.935732

EE+ Zero-point Energy -3717.206992

EE + Thermal Free Energy Correction -3717.280519

15 0.946339000 -1.011303000 1.223041000

6 3.170806000 -1.460545000 -0.372275000

6 2.665269000 -1.589807000 0.928321000

15 2.222620000 -0.360817000 -1.481881000

6 3.275932000 1.182151000 -1.613263000

6 3.342697000 1.791044000 -0.204441000

1 2.343836000 2.002975000 0.191038000

1 3.855381000 1.125942000 0.498154000

6 4.692110000 0.888109000 -2.120660000

1 5.226925000 1.837927000 -2.234142000

1 5.261756000 0.273394000 -1.419758000

1 4.686772000 0.393124000 -3.096995000

6 2.595078000 2.161178000 -2.580006000

1 1.598868000 2.446121000 -2.235985000

1 3.202237000 3.070753000 -2.649541000

1 2.506133000 1.740976000 -3.587271000

6 2.239267000 -1.200601000 -3.094511000

1 3.249673000 -1.480616000 -3.402518000

1 1.628955000 -2.104237000 -3.010526000

1 1.798846000 -0.551934000 -3.854454000

6 1.075470000 -0.160599000 2.828907000

1 1.753999000 0.688437000 2.715618000

1 0.095418000 0.210024000 3.134494000

1 1.458039000 -0.823335000 3.610147000

6 -0.026128000 -2.581134000 1.544915000

6 0.693657000 -3.614648000 2.415528000

1 -0.003941000 -4.433688000 2.626884000

1 1.566217000 -4.042565000 1.915819000

1 1.009631000 -3.196237000 3.376393000

6 -0.336494000 -3.199913000 0.175400000

1 -0.883730000 -4.139758000 0.310903000

1 -0.950765000 -2.536603000 -0.441048000

1 0.584605000 -3.423519000 -0.375818000

6 -1.315308000 -2.155804000 2.259566000

1 -1.774446000 -1.283689000 1.792477000

1 -2.043800000 -2.973592000 2.223354000

1 -1.125688000 -1.920354000 3.311381000

6 3.451227000 -2.205990000 1.908174000

6 4.389456000 -2.056992000 -0.710019000

6 4.683456000 -2.756969000 1.576400000

1 3.100355000 -2.275295000 2.932019000

6 5.138372000 -2.712266000 0.259977000

1 4.764941000 -1.998196000 -1.726368000

1 5.282204000 -3.237923000 2.343446000

1 6.085641000 -3.170709000 -0.005801000

6 -1.597269000 1.697080000 0.792492000

7 -1.475472000 0.843033000 -0.398432000

1 3.897965000 2.734771000 -0.240794000

28 0.348863000 0.065285000 -0.633525000

6 -0.487683000 2.738657000 0.830865000

6 0.155323000 3.014895000 2.037272000

6 -0.160605000 3.486053000 -0.304675000

6 1.137885000 3.999248000 2.102444000

1 -0.117841000 2.462770000 2.930076000

6 0.821016000 4.469620000 -0.238363000

1 -0.670968000 3.316236000 -1.248725000

6 1.475545000 4.725768000 0.963991000

1 1.636360000 4.198665000 3.045827000

1 1.072739000 5.036100000 -1.129216000

1 2.243055000 5.491743000 1.013722000

6 -2.515112000 -0.148696000 -0.612937000

6 -3.495361000 -0.456764000 0.318390000

6 -2.506283000 -0.821081000 -1.840228000

6 -4.441231000 -1.450692000 0.055449000

1 -3.548206000 0.045947000 1.277570000

6 -3.445825000 -1.796732000 -2.113483000

1 -1.752264000 -0.576225000 -2.582116000

6 -4.416679000 -2.127949000 -1.159843000

1 -5.184916000 -1.673070000 0.810783000

1 -3.439784000 -2.321044000 -3.063382000

8 -5.285296000 -3.110293000 -1.515376000

6 -2.920019000 2.457889000 0.869068000

8 -3.443652000 2.767520000 1.918739000

8 -3.385179000 2.780256000 -0.329543000

6 -4.609430000 3.532675000 -0.357126000

1 -4.475280000 4.486565000 0.156426000

1 -4.828342000 3.693322000 -1.411004000

1 -5.409532000 2.960424000 0.116461000

6 -6.285539000 -3.484401000 -0.579373000

1 -6.948970000 -2.643775000 -0.347085000

1 -6.861705000 -4.276782000 -1.057774000

1 -5.841549000 -3.867730000 0.346324000

1 -1.536561000 1.072612000 1.685864000

1 0.288753000 0.578554000 -2.013379000

1 -1.523861000 1.459516000 -1.207441000
